# Secondary Metabolite Biosynthesis Potential of *Streptomyces* Spp. from the Rhizosphere of *Leontopodium nivale* Subsp. *alpinum*

**DOI:** 10.1021/acsomega.4c10476

**Published:** 2025-02-13

**Authors:** Anna Vignolle, Martin Zehl, Rasmus H. Kirkegaard, Gabriel A. Vignolle, Sergey B. Zotchev

**Affiliations:** 1 Department of Pharmaceutical Sciences, Division of Pharmacognosy, University of Vienna, Vienna 1090, Austria; 2 Department of Analytical Chemistry, Faculty of Chemistry, University of Vienna, Vienna 1090, Austria; 3 Joint Microbiome Facility, Medical University of Vienna and University of Vienna, Vienna 1030, Austria; 4 Division of Microbial Ecology, Centre for Microbiology and Environmental Systems Science, University of Vienna, Vienna 1090, Austria; 5 Center Health & Bioresources, Competence Unit Molecular Diagnostics, AIT Austrian Institute of Technology GmbH, Giefinggasse 4, Vienna 1210, Austria

## Abstract

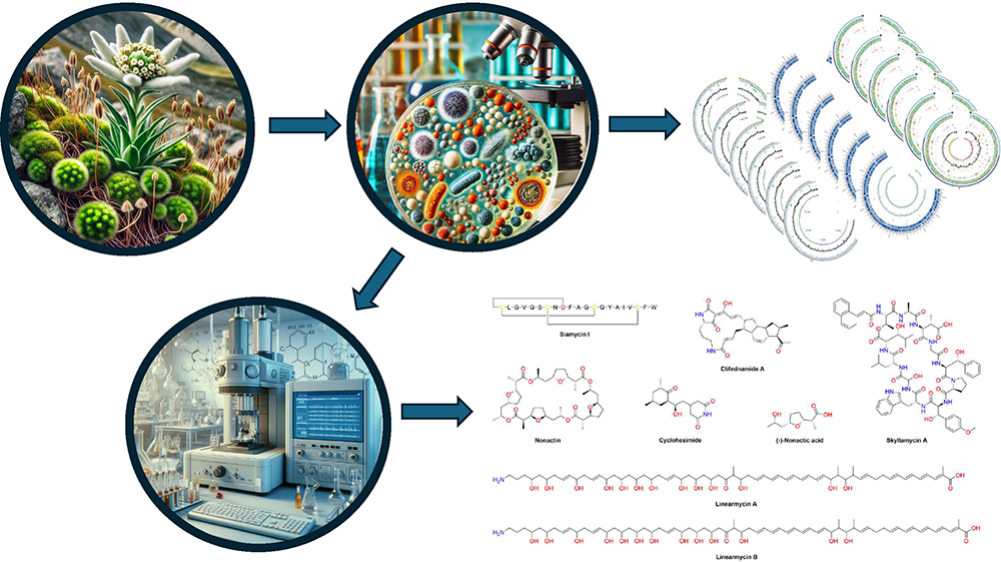

Bacteria of the phylum Actinomycetota, particularly those of the genus *Streptomyces*, are prolific producers of secondary metabolites (SMs), many of which have been developed into antibiotics, immunosuppressants, and cancer therapeutics. With high rediscovery rates, the attention has shifted to *Streptomyces* from unique ecological niches for the discovery of new SMs. The plant rhizosphere is one such niche, characterized by complex chemical interactions between the plant and its rhizobiome, which can elicit the production of SMs in *Streptomyces*. In the present study, 18 S*treptomyces* strains were previously isolated from the rhizosphere of the rare alpine medicinal plant *Leontopodium nivale* subsp. *alpinum* were investigated for their capacity to produce secondary metabolites. Genomes of these strains were analyzed for the presence of SM biosynthetic gene clusters (BGCs). In total, 551 BGCs were detected, of which 217 could not be linked to known SMs. These isolates were cultivated in different media known to support the production of SMs, and 15 out of the 54 methanolic extracts from these cultures exhibited antimicrobial activities. Subsequent liquid chromatography–mass spectrometry analyses of the bioactive extracts led to a putative identification of 69 known SMs as well as 16 potentially new molecules. The results of this study may provide a basis for the discovery of unique molecules with the potential to be developed as drugs against a variety of human diseases.

## Introduction

New natural products (NPs) that can be developed into drugs like antibiotics and chemotherapeutics are urgently needed due to the rise of antimicrobial resistance and virtual lack of breakthroughs in finding new drug leads using high throughput screening of synthetic compound libraries.^1^ Due to their structural diversity and wide range of bioactivities, NPs are excellent candidates for the development of new antibiotics, as well as drugs against other diseases, e.g., cancer. In the past decade, eight of the 19 newly approved antibiotics were derived from NPs and 16 NP-derived compounds were undergoing clinical trials.^2^ Gram-positive bacteria of the phylum Actinomycetota, also known as Actinobacteria, are widely recognized as prolific producers of bioactive secondary metabolites (SMs), some of which have been developed into antibacterial, antifungal, and anticancer drugs. However, the high rate of rediscovery of already known SMs from Actinobacteria has discouraged many pharmaceutical companies from further exploring this bacterial phylum. To minimize the rediscovery rate, screening of Actinobacteria from rare environments and unique ecological niches gained more importance in recent years. One such ecological niche is the plant rhizosphere.

The plant rhizosphere is comprised of the thin cover of soil surrounding plant roots and the microbiota contained therein and has been described as a unique environment for the production of SMs.^3^ Signaling molecules released by the plant via root exudates trigger the production of SMs by rhizosphere microbiota, especially by Actinobacteria. Many of such SMs can be beneficial to the host plant.^4^ SMs, also known as specialized metabolites, are compounds biosynthesized from building blocks originating in primary metabolism utilizing complex enzymatic machinery. In contrast to primary metabolites, such as sugars, lipids, and amino acids, SMs are not vital for the survival of the producing organism under laboratory conditions.^5^ However, secondary metabolites provide an advantage in the natural habitat of the producing organism and fulfill diverse functions ranging from metal scavenging to the defense of nutritional resources.^4^

The rhizosphere, together with the phyllosphere and the endosphere, forms the plant microbiome that includes diverse microorganisms, such as fungi, archaea, and bacteria. Plants actively recruit their endophytes from the rhizosphere.^3^ Filamentous Actinobacteria, including species of the genus *Streptomyces*, arose around 440 million years ago, coinciding with terrestrial colonization by plants. Their filamentous morphology likely conferred an advantage in colonizing plant roots.^1^

Actinobacteria isolated from rare plants, plants growing in extreme environments, or their rhizospheres represent particularly promising candidates for the discovery of novel SMs. Search for novel SMs from such bacteria can be facilitated by a strategy called genome mining, which involves bioinformatics analysis of sequenced genomes to identify novel biosynthetic gene clusters (BGCs) responsible for SM production, followed by experimental validation to harness this genomic potential.^6^*Streptomyces* genomes have been shown to contain up to 50 BGCs, with many of them not yet described and thus potentially encoding the biosynthetic pathways of novel SMs.^7^ Many of these BGCs are not expressed under laboratory conditions while being detected upon genome analysis with bioinformatics tools like antiSMASH.^8^ The latter online software can detect BGCs and predict the types of core biosynthetic enzymes encoded by the cluster, which allows for a prediction of the chemical class of the cognate product.^7^ Several independent bioinformatics tools that embed or use antiSMASH have been developed in recent years, for instance, BiG-SCAPE, which can be used to investigate the range of diverse BGCs across large genomic data sets. This is achieved through the creation of BGC sequence similarity networks, the classification of BGCs into families, and the examination of gene cluster diversity in relation to enzyme phylogenies.^9^

In this study, we investigated the SM production potential of 18 *Streptomyces* spp. isolated from the rhizosphere of the rare alpine medicinal plant *Leontopodium nivale* subsp. *alpinum* (Edelweiss). This was achieved using conventional cultivation and screening of the extracts for antimicrobial activity coupled to liquid chromatography–mass spectrometry (LC-MS) and genome analyses. This combination of approaches allowed for the identification of both known and potentially novel SMs, which can be further explored in follow-up studies.

## Experimental Section

### Growth Conditions of Bacterial Strains

*Streptomyces* spp. isolated previously from the rhizosphere of *Leontopodium nivale* subsp. *alpinum* (RLA) were cultivated on either soy flour (SFM), CP6, or ISP2 agar medium, depending on which one was best suited for the respective strain with respect to growth and sporulation (Table S1, Supporting Information), at 28 °C for 7–10 days, until sporulation occurred.^10^ Spore suspensions were prepared in 20% glycerol and stored at −80 °C.

### Genome Sequencing and Analyses

Genomic DNA was extracted from pure cultures of the 18 rhizosphere isolates using a Power Soil Pro Kit (Qiagen). For nanopore sequencing, extracted DNA was prepared using the rapid barcoding sequencing kit (SQK-RBK004, Oxford Nanopore Technologies) following the manufacturer's protocol. The DNA was sequenced on a MinION Mk1b (Oxford Nanopore Technologies) on an R9.4.1 flowcell (FLO-MIN106D, Oxford Nanopore Technologies). The DNA sequencing was carried out using Minknow (version 20.10.3, Oxford Nanopore Technologies).

For Illumina sequencing, DNA was prepared using the NEBNext Ultra II DNA Library Prep Kit (New England Biolabs) and sequenced on the Illumina MiSeq platform using V3 chemistry and 600 cycles (2 × 300 bp). Nanopore reads were basecalled using Guppy v4.2.2, assembled using flye v2.8.3-b1695,^11^ and then polished twice with minimap2 v2.17 and racon v1.4.3, followed by two rounds of polishing with medaka (v. 1.1.2, github.com/nanoporetech/medaka).^12,13^ The Illumina reads were trimmed using cutadapt v3.1, and then used to polish nanopore genome assemblies using minimap2 v2.17 and racon v1.4.3.^13,14^ Genome quality was checked using QUAST (v. 5.0.2), CheckM (v. 1.1.1), and classified using GTDBtk (v. 1.5.0).^15−17^ Genome assembly features are presented in Table S2 (Supporting Information).

The genomes were compared using fastANI (v. 1.3).^18^ The assembled genomes were analyzed with antiSMASH 7.0 and BiG-SCAPE.^8,9^ The potential novelty of BGCs was determined by a comparison of detected BGCs to known ones in the MIBiG database. If the sequence similarity was below 60% and the gene composition was different, then the BGC in question was considered potentially novel.

We performed two BiG-SCAPE analyses, one on only the detected RLA BGCs in default mode and the second including all MIBiG BGCs encompassed in Version 1.1.5 of BiG-SCAPE. We performed an exploratory analysis of the output focusing on the detection of potentially novel BGCs and inspected the calculated networks manually.

### Preparation of Fermentation Extracts for Bioactivity Testing and Secondary Metabolite Analysis

For the generation of seed cultures, 10 mL of the liquid medium best suited for the respective strain (Table S1, Supporting Information) was inoculated with 100 μL of spore suspension and incubated in 150 mL shake flasks for 2 days at 28 °C and 200 rpm on a rotary shaker. Next, 250 mL baffled flasks containing 50 mL of the respective medium—MYM (4 g/L maltose, 4 g/L oxoid yeast extract, 10 g/L malt extract, 1.9 g/L 3-(*N*-morpholino)propanesulfonic acid, (MOPS)), SM17, or PM4—were inoculated with 3 mL of each seeding culture. Fermentations were carried out for 7–10 days at 28 °C and 200 rpm. The cultures were then harvested, frozen, and lyophilized. The lyophilizates were extracted with 50 mL methanol on a shaker at 120 rpm for 1 h. Next, the extracts were centrifuged to remove solid particles, and the supernatant filtered into pear-shaped flasks. The methanol was evaporated on the rotary evaporator, set to 40 °C and 280 mbar. The residue was dissolved in 3 mL of methanol. These extracts were stored at −20 °C and used for bioactivity tests and LC-MS analyses.

### Bioactivity Testing

The antimicrobial activities of the culture extracts were evaluated by using disc diffusion assays. Test organisms for bioassays were cultivated overnight for utilization on the subsequent day. Details of growth conditions and media can be found in Table S3 (Supporting Information). A sterile test tube containing 2 mL of the suitable growth medium was inoculated with 100 μL of glycerol stock of the respective test organism. The organisms were then incubated overnight at the appropriate temperature (Table S3, Supporting Information). On the following day, 1.5 mL of the culture was aliquoted into a 1.5 mL Eppendorf tube and centrifuged at 4000 rpm for 2 min. After aspiration and discarding of 1 mL of the supernatant, the pellet was resuspended in the remaining 500 μL. This cell suspension was used to inoculate large Petri dishes (150 × 25 mm) containing suitable solid medium (Table S3, Supporting Information). The methanolic fermentation extracts were applied onto 9 mm Whatman antibiotic assay discs. Onto each disc, 50 μL of the extract was applied in a laminar airflow workbench, followed by a 30 min drying period. As controls, methanol, and uninoculated media were used −50 μL of methanol and 50 μL of each uninoculated medium (SM17, PM4, and MYM) were each added to one antibiotic assay disc under sterile conditions in a laminar airflow workbench and dried for 30 min. The discs were placed on the surface of the Petri dishes inoculated with test organisms using flame-sterilized metal tweezers. The bioassays were incubated overnight at the optimal growth temperature of the test organism (Table S3, Supporting Information). Following the incubation, an assessment of the bioassays was conducted. The presence of growth inhibition zones around the paper discs, measured in millimeters, indicated the antibiotic properties of the tested extract.

### Secondary Metabolomics

LC-MS analyses of the culture extracts were performed on a Vanquish Horizon UHPLC system (Thermo Fisher Scientific) coupled to the ESI source of a timsTOF fleX mass spectrometer (Bruker Daltonics). Separation was carried out on an Acquity Premier HSS T3 column, 2.1 mm × 150 mm, 1.8 μm (Waters) using water and acetonitrile/water 9:1, both modified with 0.1% formic acid, as mobile phases A and B, respectively. The sample components were separated and eluted with a gradient starting with a linear increase from 0% to 20% B in 10 min, followed by a linear increase from 20 to 100% B in 25 min, and finally an isocratic column cleaning (3 min at 100% B) and re-equilibration step (5 min at 0% B). The flow rate was 0.5 mL/min, and the column oven temperature was set to 40 °C.

High-resolution ESI-MS and MS/MS spectra were recorded in positive ion mode in the range *m*/*z* 100–2500. CID spectra of the five most intense precursor ions in each MS^1^ spectrum were obtained in automated data-dependent acquisition mode using nitrogen as a collision gas. The sum formulas of the detected ions were determined using Bruker Compass DataAnalysis 5.3 based on the mass accuracy (Δ*m*/*z* ≤ 5 ppm) and isotopic pattern matching (SmartFormula algorithm).

Compounds represented in GNPS were identified by a MOLECULAR-LIBRARYSEARCH-V2 (release_28) workflow, whereby in the case of the very common hydroxamate siderophores only the main congeners present in the extracts are listed here.^19^ Next, Bruker Compass DataAnalysis 5.3 was used for comparing the LC-MS data of all seven investigated strains to find secondary metabolites that are specific to individual strains. Identification of known compounds was accomplished with the aid of The Natural Products Atlas and CAS SciFinder (American Chemical Society) based on the predicted sum formula and plausible match of MS/MS and DAD data.^20^

### Bioactivity-Guided Fractionation

One bioactive crude extract was fractionated into nine fractions via semipreparative HPLC. The semipreparative HPLC was performed on a Shimadzu HPLC system LC-20AT using a Luna C18, 10 mm × 250 mm, 5 μm HPLC semipreparative column (Phenomenex). The mobile phase was a gradient of solvent A (dH_2_O, supplemented with 0.1% formic acid) and solvent B (acetonitrile). The gradient was 5–95% B in 45 min, concluded with a washing step of 10 min at 95% solvent B and a re-equilibration step of 5 min at 10% solvent B. The selected detection wavelength was 190 nm, and the flow rate was 5 mL/min. Following the semipreparative HPLC, acetonitrile was evaporated from the nine fractions on a rotary evaporator, and the water was removed via lyophilization. The nine fractions of the extract were each dissolved in 150 μL of methanol.

## Results and Discussion

### Genomics-Based Phylogeny of the Edelweiss Rhizosphere Isolates

The genomes of the 18 *Streptomyces* spp. isolates were sequenced using a combination of Nanopore and Illumina sequencing technologies (see Experimental Section). The average completeness of the genomes was 99.67%, with five genomes having a completeness of 100% (see Table S2, Supporting Information). The average genome size of the isolates was 9.4 Mb, with the largest one being 11.9 Mb and the smallest one being 8.1 Mb.

**Figure 1:**
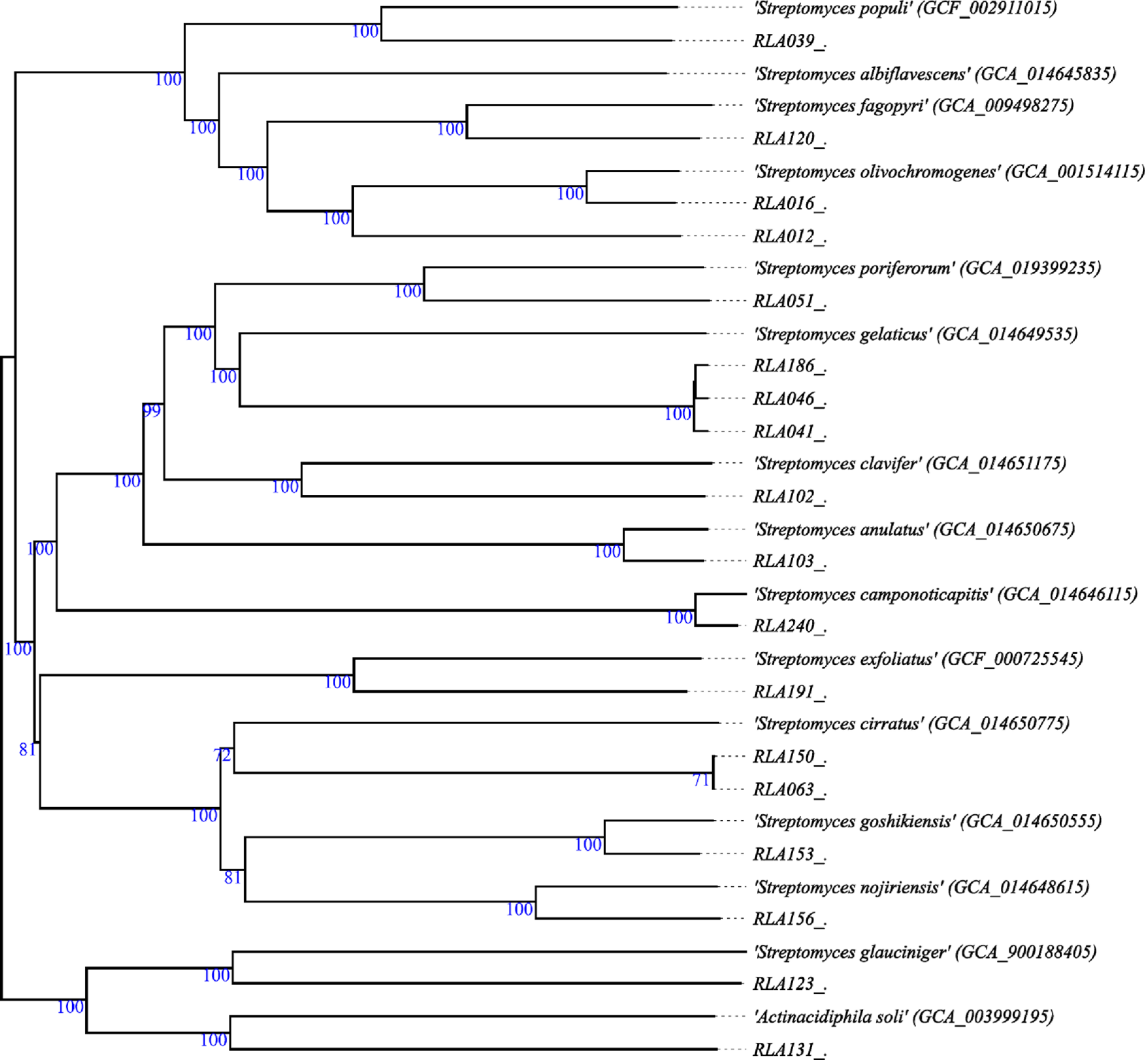
Genome-based phylogenetic tree of rhizosphere *Streptomyces* isolates. The phylogenetic tree was rendered with the phylo.io software; at the nodes, the bootstrap values are indicated; and in the bottom right legend, the respective branch length. The tree was calculated with TYGS-type strain genome server,^21^ providing a whole genome-based taxonomy.

The genome-based phylogeny of the isolates was analyzed with a TYGS-type strain genome server.^21^ This whole-gene-based taxonomy analysis identified four out of the 18 *Streptomyces* spp. as belonging to known species (Figure 1). The remaining 14 strains could, based on the dDDH values compared to those for the type strains in the TYGS database, represent new species. The four known species are RLA016, which was identified as *Streptomyces olivochromogenes*; RLA103, identified as *Streptomyces anulatus*; RLA153, identified as *Streptomyces goshikiensis*; and RLA240, identified as *Streptomyces camponoticapitis*. Interestingly, according to this analysis, isolate RLA131 was found to be most closely related to the type strain of a nonstreptomycete actinomycete, *Actinacidiphila soli* JCM 32822. This bacterium was isolated from soil collected in a birch forest in Xinjiang Uygur Autonomous Region (P.R. China) and initially classified as *Streptomyces soli*.^22^

### Secondary Metabolite Production Potential of the *Streptomyces* Rhizosphere Isolates

For the analysis of the SM production potential of the 18 *Streptomyces* spp. rhizosphere isolates, their genomes were analyzed with antiSMASH 7.0.^8^ With genome sizes ranging from 8.1 Mb for *Streptomyces* sp. RLA191 to 11.9 Mb for *Streptomyces* sp. RLA012, there appears to be no strict correlation between the genome size and the number of detected BGCs (Table 1). A total of 551 BGCs were detected with antiSMASH 7.0 in all of the isolates, with the isolate having the highest number of BGCs being *Streptomyces* sp. RLA012 (45 BGCs detected), which also has the largest genome (Table 1). The lowest detected number of BGCs was 17 in strain *Streptomyces* sp. RLA041. The antiSMASH results were further analyzed using BiG-SCAPE to reveal the BGC diversity.^9^ The BiG-SCAPE analysis facilitated the dereplication of 119 putative BGCs, demonstrating relatedness to known BGCs in the MIBiG database (Table 2).

**Table 1:** Genome Sizes and Numbers of Detected BGCs in the Analyzed *Streptomyces* Sp. Rhizosphere Isolates^a^

species	strain	genome size in Mb	number of BGCs
*Streptomyces* sp.	RLA191	8.106721	31
*Streptomyces* sp.	RLA123	8.286967	23
*Streptomyces* sp.	RLA131	8.570756	30
*Streptomyces anulatus*	RLA103	8.674807	35
*Streptomyces* sp.	RLA186	8.895788	18
*Streptomyces camponoticapitis*	RLA240	8.943973	38
*Streptomyces goshikiensis*	RLA153	8.971571	38
*Streptomyces* sp.	RLA041	8.982861	17
*Streptomyces* sp.	RLA063	9.039871	34
*Streptomyces* sp.	RLA046	9.043344	18
*Streptomyces* sp.	RLA150	9.142704	36
*Streptomyces* sp.	RLA102	9.175665	28
*Streptomyces* sp.	RLA156	9.375778	39
*Streptomyces* sp.	RLA051	9.514153	23
*Streptomyces* sp.	RLA039	9.867097	31
*Streptomyces* sp.	RLA120	10.084162	30
*Streptomyces olivochromogenes*	RLA016	11.928114	37
*Streptomyces* sp.	RLA012	11.981612	45

a The RLA strains are sorted by genome size from the smallest (top) to the largest (bottom). There appears to be no direct correlation between the size of the genome and the observed number of BGCs.

**Table 2:** This Table Is Based on the BiG-SCAPE Output Performed on All Detected BGCs^a^

**BGC type**	**total**	**singletons**	**links**	**families**	**associated MIBiG BGC**	**unknown BGCs**
other	211	116	207	144	19	173
NRPS	129	90	32	106	14	113
PKS I	35	27	4	31	12	31
PKS other	107	77	23	89	50	80
RiPPs	124	81	49	97	9	120
terpene	114	60	114	74	5	88
saccharides	4	4	0	4	1	3
hybrid	33	25	8	28	5	30

a The column “total” indicates the number of BGCs from the RLA strains used for the analysis of the specific BGC type. The column “singletons” indicates how many BGCs did not build any families with other BGCs (based solely on the RLY BGCs). “Links” numerates the connections between the BGCs in the network analysis and represents the surpassing of the similarity threshold in the BiG-SCAPE analysis between the RLA BGCs. “Families” indicates the number of detected BGC families (including singletons). “Unknown BGCs” is the number of BGCs that did not have any connection to a MIBiG BGC in the BiG-SCAPE analysis (some BGCs were grouped in two or more BGC types in the BiG-SCAPE analysis).

These dereplicated BGCs exhibited close similarity in the calculated gene cluster families and subsequently in the SMs they potentially specify. Hence, they were considered known entities and consequently removed from further analysis to prioritize the exploration of potentially novel BGCs. This approach enhances the efficiency of BGC discovery by focusing efforts on unexplored gene cluster families, potentially leading to the identification of new SMs and their associated biosynthetic pathways (see Table 2). Considering solely the BiG-SCAPE analysis, we were able to detect 399 putatively unique BGCs. By further manual curation of the antiSMASH results, the number of potentially “unique” BGCs could be narrowed down to 217. For this purpose, the “uniqueness” of a BGC was assessed via its comparison to known BGCs in the MiBIG database. The BGC at hand was considered potentially unique if its gene composition was different from any BGC with a known cognate product. The detailed antiSMASH-assisted analysis of BGCs in the genomes of 18 isolates is presented as an Excel file in the Supporting Information.

### Antimicrobial Activities and Secondary Metabolomes of the *Streptomyces* Rhizosphere Isolates

For the assessment of their antimicrobial activities, the isolates were cultivated in the fermentation media MYM, SM17, and PM4 (see Experimental Section). The 54 crude methanolic extracts from these cultures were tested against *Bacillus subtilis*, *Staphylococcus carnosus*, *Escherichia coli*, *Saccharomyces cerevisiae*, and *Pseudomonas fluorescens*. As controls, methanol and uninoculated media MYM, SM17, and PM4 were used. Of the 54 extracts tested, 15, derived from strains RLA016, 039, 051, 063, 102, 103, 150, and 153 displayed antimicrobial activity. Out of these, 12 were active against the Gram-positive test organisms *B. subtilis* and *S. carnosus*, 3 were active against the Gram-negative test organisms *E. coli* and *P. fluorescens*, and 8 extracts displayed bioactivity against the yeast *S. cerevisiae* (Table 3). None of the controls, which were used in the disc diffusion assays, displayed any activity against the test organisms. These results suggested that a wide range of SMs with antimicrobial activities can be produced by the *L. nivale* subsp. *alpinum* rhizosphere *Streptomyces* isolates.

**Table 3:** Antimicrobial activity of crude methanolic extracts from fermentation broths of the 18 RLA isolates in the media MYM, SM17, and PM4^a^

	*B. subtilis*			*E. coli*			*S. carnosus*			*P. fluorescens*			*S. cerevisiae*		
strains	MYM	SM17	PM4	MYM	SM17	PM4	MYM	SM17	PM4	MYM	SM17	PM4	MYM	SM17	PM4
RLA012	–	NA	–	–	–	–	–	–	–	–	–	–	–	–	–
RLA016	++	–	–	–	–	–	–	–	–	–	–	–	–	–	–
RLA039	–	–	–	–	–	–	–	–	–	–	–	–	–	+	–
RLA041	–	–	–	–	–	–	–	–	–	–	–	–	–	–	–
RLA046	–	–	–	–	–	–	–	–	–	–	–	–	–	–	–
RLA051	–	–	–	–	+	–	+	++	–	–	+	–	–	–	–
RLA063	++	++	–	–	–	–	–	–	–	–	–	–	++	–	–
RLA102	++	+++	–	–	–	–	–	–	–	–	–	–	–	–	–
RLA103	–	–	–	–	–	–	–	+	–	–	–	–	++	+++	+
RLA120	–	–	–	–	–	–	–	–	–	–	–	–	–	–	–
RLA123	–	–	–	–	–	–	–	–	–	–	–	–	–	–	–
RLA131	–	NA	–	–	–	–	–	–	–	–	–	–	–	–	–
RLA150	–	–	–	–	+	–	–	+	–	–	–	–	–	–	–
RLA153	–	–	–	–	–	–	+	++	+	–	–	–	++	++	++
RLA156	–	–	–	–	–	–	–	–	–	–	–	–	–	–	–
RLA186	–	–	–	–	–	–	–	–	–	–	–	–	–	–	–
RLA191	–	NA	–	–	–	–	–	–	–	–	–	–	–	–	–
RLA240	–	NA	–	–	–	–	–	–	–	–	–	–	–	–	–
uninoculated media															
methanol															

a Within the table, a “–“ represents the absence of an inhibition zone, a “+” represents an observed inhibition zone of ≤0.5 cm, a “++” represents an inhibition zone of 0.5-1.0 cm, and a “+++” represents an inhibition zone of >1.0 cm. NA – not assessed. Methanol and uninoculated media MYM, SM17, and PM4 were used as controls.

The extract from the *S. anulatus* RLA103 culture grown in medium SM17 displayed relatively strong bioactivity against *S. cerevisiae* and weak bioactivity against *S. carnosus* (Table 3). In order to identify the compounds potentially responsible for these activities, bioactivity-guided fractionation of the extract was performed. The fractions were retested against the same two test organisms and the bioactive fractions were analyzed with LC-MS. The latter was shown to contain cycloheximide and nonactins, which are likely responsible for the observed bioactivities.

### LC-MS Analysis of Bioactive Extracts

All crude methanolic extracts of isolates that exhibited antimicrobial activity were subjected to LC-MS analysis for dereplication of known secondary metabolites and putative identification of the active compounds. For each isolate, extracts from three fermentation media were analyzed—MYM, PM4, and SM17. A total of 85 SMs were detected, 16 of which are potentially novel (Table S4 and Figures S1–S85, Supporting Information). The known SMs gaburedin A, B, C, and D—a family of γ-aminobutyrate-derived ureas first discovered in *Streptomyces venezuelae* ATCC 10712—were identified in the extracts from several isolates, demonstrating them to be rather common in *Streptomyces* spp.^23^

The secondary metabolomes of *Streptomyces* sp. RLA063 and *Streptomyces* sp. RLA150 is virtually the same, and their genomes are highly similar, although there are some differences concerning the nature and number of BGCs in these two strains (Table 1). Thus, only the data for *Streptomyces* sp. RLA063 is listed in Table S4 (Supporting Information). It is worth noting that these two isolates have genomes, including BGC composition, that are very similar to those of *Streptomyces* sp. LN699, which was isolated from the Edelweiss plant itself.^24^ The extract of the LN699 culture grown in MYM medium showed activity against *S. cerevisiae* and *B. subtilis*, same as for RLA063 but not RLA150 isolates (Table 3). In general, the *Streptomyces* isolates from the Edelweiss rhizosphere analyzed in this study were more bioactive compared with those originating from the plant itself. This may reflect a more challenging environment that rhizosphere isolates are exposed to compared to a better-protected endosphere of the plant.

**Figure 2:**
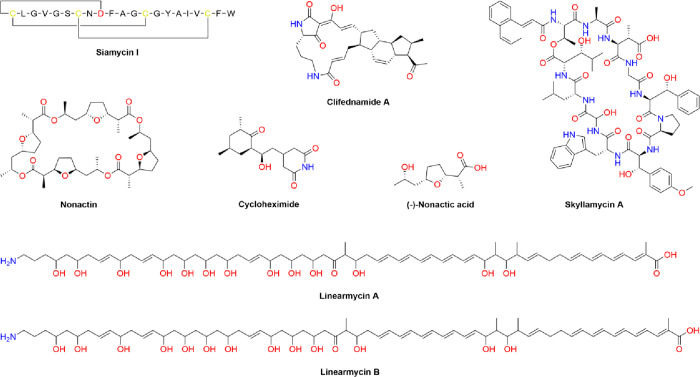
Chemical structures of several known bioactive compounds detected with LC-MS in the extracts of *Streptomyces* spp. from the rhizosphere of Edelweiss.

The analysis of the three extracts from *S. olivochromogenes* RLA016 resulted in the detection of 11 SMs, among them a potentially novel one. The known compounds include the lasso peptide siamycin I (see Figure 2), which has been shown to inhibit Gram-positive bacteria by targeting cell wall biosynthesis.^25^ Siamycin I biosynthesis is specified by BGC 1.11 in the genome of *S. olivochromogenes* RLA016. The observed bioactivity of the MYM-derived extract containing siamycin I against Gram-positive test organism *B. subtilis* is likely to be caused by this antibiotic. An abundant, potentially novel compound has been detected in the extract from the RLA016 cultures grown in PM4 and SM17 media. Another very abundant compound detected in all three media was tentatively identified as surugapyrone A, also known as germicidin D, which acts as a DPPH radical-scavenger and inhibits the germination of spores.^26,27^ However, this metabolite could also be a structurally very similar isomer of surugapyrone A, namely, 6-ethyl-4-hydroxy-3,5-dimethylpyran-2-one, which was isolated from *Streptomyces* sp. Tü 6077 and has been suggested to be a precursor in the biosynthesis of the spiro[4.5]decene spirodionic acid.^28^ A compound fitting the latter was also found in *S. olivochromogenes* RLA016 extracts in small quantities.

The crude methanolic extract of the isolate *Streptomyces* sp. RLA051 cultivated in SM17 was active against Gram-negative test organisms *E. coli* and *P. fluorescens* and Gram-positive test organism *S. carnosus*. The known secondary metabolites putatively identified in this extract are *N*-(2,3-dihydroxybenzoyl)serine monomer and dimer, presumably shunt products from incomplete enterobactin biosynthesis by BGC 1.4, as well as *N*-heptanoyl-arginine. Enterobactin is a known catecholate siderophore produced by several *Streptomyces* species, for which no antibacterial activity has been reported.^29^ We therefore hypothesize that the two unidentified secondary metabolites found in this extract (Table S4, Supporting Information) may be responsible for the observed antibiotic activity. The analysis of the extract of *Streptomyces* sp. RLA051 culture grown in MYM medium led to the detection of the secondary metabolites piceamycin, phevalin, and the analogous pyrazinone originating from the Ile-Phe aldehyde, all in rather low amounts.^30^ Piceamycin is a macrolactam antibiotic with a reported activity against Gram-positive bacteria, which would explain the observed activity of this extract against *S. carnosus*.^31^ The biosynthesis of piceamycin is apparently specified by BGC 1.13 in *Streptomyces* sp. RLA051. The compound phevalin has been reported to inhibit the protease calpain.^32^

Two of the three extracts of *Streptomyces* sp. RLA063 were bioactive—the extract from the MYM-grown culture was active against *B. subtilis* and *S. cerevisiae*; the SM17-grown culture extract displayed bioactivity against *B. subtilis*. The MYM- and SM17-grown culture extracts contain the compounds KF77-AG6, phevalin, *N*-acetylphenylalaninol, and an unknown nucleoside, among other compounds (Table S4, Supporting Information). KF77-AG6 is a *Streptomyces* metabolite related to the protease inhibitor antipain.^33^ In the extract from the fermentation in SM17 the compounds antipain, leupeptin, MAPI, and many congeners of these reactive aldehydes were detected, as well as coelimycin P1. MAPI is an inhibitor of microbial alkaline proteinases.^34^ The compound Mer-N5075-A is related to MAPI and was reported to inhibit HIV-1 protease.^35^ Leupeptin and its congeners are produced by numerous species of *Streptomyces* and inhibit proteases like trypsin and papain.^36^ The polyketide alkaloid coelimycin P1, a yellow pigment, was previously discovered in *Streptomyces coelicolor* M145.^37^ Since none of the known detected compounds explain the observed bioactivity, we hypothesize that it is caused by the unknown molecule with the sum formula C_16_H_19_N_5_O_9,_ which was tentatively identified as a nucleoside due to an MS/MS (Figure S21, Supporting Information) and UV-spectrum typical of adenine-glycosides and may have antibiotic activity.

The fermentation extracts of strain *Streptomyces* sp. RLA102 grown in the media MYM and SM17 was bioactive against *B. subtilis*. In both extracts, only one prominent peak of a potentially novel secondary metabolite with the sum formula C_9_H_16_N_4_O_3_ was detected with LC-MS. However, since this compound is also present in the antibiotically inactive PM4 extract, it is unlikely to possess relevant antibiotic activity.

The bioactivity exhibited by the extract of *Streptomyces anulatus* RLA103 cultivated in SM17 against the test organism *S. cerevisiae* is likely caused by the detected cycloheximide isomers. The biosynthetic pathway for cycloheximide is encoded by BGC 1.3 in the genome of *S. anulatus* RLA103. Cycloheximide (see Figure 2) is a known inhibitor of eukaryotic protein biosynthesis that targets the elongation phase.^38^ The bioactivity against *S. carnosus*, on the other hand, can be explained by the presence of the macrotetrolide antibiotic nonactic acid (see Figure 2) and its congeners, which reportedly have activity against *Staphylococcus aureus* and other Gram-positive bacteria.^39^ The BGC that specifies nonactic acid biosynthesis in *S. anulatus* RLA103 has not been identified yet and is the subject of further studies. The SM17 extract further contains the siderophore dehydroxynocardamine, the biosynthesis of which is specified by BGC 1.24 in the genome of *S. anulatus* RLA103, and the siderophore schizokinen, which is specified by BGC 1.15.^40,41^ Cycloheximide and nonactin congeners (Figure 2) were identified in all three extracts of *Streptomyces anulatus* RLA103 in various concentrations.

Accordingly, the extract of the culture from the MYM medium was also bioactive against *S. cerevisiae*. Furthermore, this extract contains three groups of potentially new secondary metabolites. It was not possible to predict their sum formulas with certainty, owing to their high molecular weight. The group of compounds that elute after 22–23 min most likely represents small peptides linked to an alkenyl chain via a pyrophosphate group, with the latter two matching to geranylgeranyl pyrophosphate. These three congeners presumably differ by an alanine and a glycine residue (Figures S60, S61, and S64, Supporting Information). The potentially new compound eluting after 13.2 min gave only a few fragment ions of low intensity, suggesting a stabilized peptide with a monoisotopic mass of 1669.76 Da (Figure S54, Supporting Information). The last group of compounds was more polar, had a molecular weight of around 500 Da, and yielded intense [M+2H]^2+^ ions, which indicates two fixed charges or groups with very high proton affinity per molecule (Figures S42, S43, and S45–S48, Supporting Information). In addition, the compounds AmfS, phenatic acid A, le-pyrrolopyrazine A, and melanostatin were detected. The class III lanthipeptide AmfS is involved in the regulation of aerial mycelium formation in *Streptomyces griseus*.^42^ The phenol phenatic acid A, which is structurally related to cycloheximide, has been reported to increase the effectiveness of miconazole against *Candida albicans*.^43^ The compound melanostatin is a known inhibitor of melanin synthesis.^44^

The extract of the fermentation of *S. anulatus* RLA103 in the medium PM4 was bioactive against *S. cerevisiae* as well, which is explained by the presence of cycloheximide isomers at low levels but did not yield any additional secondary metabolites compared to the other two media. Overall, *S. anulatus* RLA103 was found to be the most bioactive strain in this study. In total, 18 known compounds were detected in three extracts from the fermentation media MYM, SM17, and PM4, which could be assigned to six BGCs. Among the known compounds were skyllamycin A (see Figure 2), detected in the extract from MYM medium and specified by BGC 1.22, a strong inhibitor of the PDGF signaling pathway, and clifednamide A (see Figure 2), which was also found in the extract from MYM medium and has a cytotoxic effect *in vitro* by triggering apoptosis through cell cycle arrest in the S phase.^45,46^ The biosynthesis of clifednamide A is specified by BGC 1.9. Furthermore, over 10 (including congeners) potentially new natural products were found in the extracts from this strain’s cultures.

The observed bioactivity against *S. carnosus* and *S. cerevisiae* by *Streptomyces goshikiensis* RLA153 is probably the effect of linearmycins A and B (see Figure 2) that were detected in all three extracts. Linearmycins have antifungal activity and have also been shown to inhibit the growth of Gram-positive bacteria.^47^ However, the extracts did not exhibit any activity against *B. subtilis*, which might be due to the low abundance of linearmycins and hence failure to reach the minimum inhibitory concentration. The MYM and SM17 culture extracts also contain an abundant, unidentified aminodisaccharide with the sum formula of C_14_H_27_NO_10_. Two other unidentified peptide-like compounds, whose sum formulas could not be determined with certainty, were detected in the MYM culture. Among the known compounds detected in the extract of *S. goshikiensis* RLA153 were the peptidic siderophores salinichelin C and desferri-peucechelin.^48,49^

## Conclusions

Our study of 18 *Streptomyces* spp. isolates from the rhizosphere of the rare alpine medicinal plant *Leontopodium nivale* subsp. *alpinum* revealed a substantial reservoir of BGCs, totaling 551 with 217 presumably not yet represented in the MIBiG database, which indicates a rich potential for secondary metabolite production by these bacteria. Furthermore, our antimicrobial activity assays demonstrated that 28% of the extracts derived from these isolates exhibited notable inhibitory effects, underscoring the pharmacological relevance of their secondary metabolites. Through LC-MS analyses of the secondary metabolomes of the bioactive strains, we identified 69 known compounds, connecting most of them to their cognate BGCs. Importantly, we also uncovered 16 potentially novel compounds produced under laboratory conditions, revealing as yet uncharacterized biosynthetic potential within this group of isolates. These findings highlight the significance of the *Streptomyces* spp. rhizosphere isolates as a valuable source for the discovery of novel secondary metabolites and underscore the importance of continued exploration of their biosynthetic potential.

## Supporting Information Available

(Table S1) Solid and liquid media used for the cultivation of the Streptomyces spp. RLA isolates; (Table S2) genome assembly characteristics; (Table S3) growth conditions and media for the cultivation of bioassay test organisms; (Table S4) secondary metabolites detected in the extracts of Streptomyces spp. from Edelweiss rhizosphere using LC-MS; (Figures S1–S85) HRESI-MS and MS/MS spectra of the detected secondary metabolites; BGCs of the 18 RLA isolates; and (Figure S86) detailed analyses of BGCs in the genomes of 18 *Streptomyces* isolates studied in this work (PDF)

## Notes

The polished assemblies generated in this study have been deposited at NCBI under the BioProject accession number PRJNA1004202.
