# Supplementary material for: Secondary Metabolite Biosynthesis Potential of Streptomyces Spp. from the Rhizosphere of Leontopodium nivale Subsp. alpinum
Source: ACS Omega. 2025 Feb 13;10(7):7163–71. doi: 10.1021/acsomega.4c10476 (PMC11865988; doi:10.1021/acsomega.4c10476)
Supplement: Supplementary file 1 — ao4c10476_si_001.pdf [file ao4c10476_si_001.pdf]

Supporting Information

Secondary metabolite biosynthesis potential of *Streptomyces* spp. from the rhizosphere of *Leontopodium nivale* subsp. *alpinum*

Anna Vignolle[1], Martin Zehl[2], Rasmus H. Kirkegaard[3,4], Gabriel A. Vignolle[5], Sergey B. Zotchev[1*]

[1]Department of Pharmaceutical Sciences, Division of Pharmacognosy, University of Vienna, 1090 Vienna, Austria.

[2]Department of Analytical Chemistry, Faculty of Chemistry, University of Vienna, 1090 Vienna, Austria.

[3] Joint Microbiome Facility, Medical University of Vienna and University of Vienna, 1030 Vienna, Austria.

[4] Division of Microbial Ecology, Centre for Microbiology and Environmental Systems Science, University of Vienna, 1090 Vienna, Austria

[5]AIT Austrian Institute of Technology GmbH, Center Health & Bioresources, Competence Unit Molecular Diagnostics, Giefinggasse 4, 1210 Vienna, Austria.

Table S1. Solid and liquid media used for the cultivation of the *Streptomyces* spp. RLA isolates.

**Strain**	**Best solid medium for sporulation**	**Liquid medium with best growth**[a]
Streptomyces sp. RLA039	SFM	2xYT
Streptomyces sp. RLA120	CP6	2xYT
Streptomyces sp. RLA123	CP6	2xYT
Streptomyces sp. RLA150	CP6	TSB
Streptomyces sp. RLA156	SFM	TSB
Streptomyces sp. RLA186	CP6 /SFM	2xYT
Streptomyces goshikiensis RLA153	CP6	2xYT
Streptomyces anulatus RLA103	SFM	2xYT
Streptomyces sp. RLA012	SFM	2xYT
Streptomyces sp. RLA191	ISP2	2xYT
Streptomyces sp. RLA046	CP6	2xYT
Streptomyces sp. RLA063	CP6	2xYT
Streptomyces camponoticapitis RLA240	CP6/ISP4	2xYT
Streptomyces olivochromogenes RLA016	SFM/ISP4	2xYT
Streptomyces sp. RLA102	SFM	2xYT
Streptomyces sp. RLA131	SFM	2xYT
Streptomyces sp. RLA041A	CP6	2xYT/TSB
Streptomyces sp. RLA051	SFM	2xYT/TSB

[a] Culture turbidity after 16 h of incubation was used as a criterion of growth.

Table S2: Genome assembly characteristics. Raw sequencing results obtained from Nanopore and Illumina sequencing. Contigs were assembled with flye and polished several times with Illumina trimmed reads. Quality metrics were calculated with QUAST and CheckM. N50 – the sequence length that comprises 50% of the Nanopore data.

Sample	N50 (bp)	Nanopore data (Mbp)	trimmed Illumina data (Mbp)	assembled contigs	completeness (%)	contamination (%)
**RLA012**	6534	497	407.6	3	100.00	0.39
**RLA016**	5621	237	570.1	5	100.00	2.14
**RLA039**	6644	523	627.6	4	100.00	1.32
**RLA041A**	6068	448	542.7	2	100.00	1.23
**RLA046**	7275	288	451.8	3	100.00	0.21
**RLA051**	8280	712	675.9	3	99.97	1.01
**RLA063**	6842	571	834.3	8	99.89	0.43
**RLA102**	7661	708	768.1	5	99.82	0.76
**RLA103**	9394	1174	548.3	2	99.71	0.64
**RLA120**	5751	846	401.6	2	99.70	1.11
**RLA123**	5337	606	469.5	5	99.64	0.99
**RLA131**	5462	870	699.6	2	99.64	1.36
**RLA150**	6629	346	478.0	15	99.57	1.32
**RLA153**	6086	280	645.4	3	99.56	0.76
**RLA156**	6122	843	623.0	7	99.29	1.51
**RLA186**	5933	786	423.1	6	99.23	1.51
**RLA191**	7354	794	636.2	1	99.20	0.00

**RLA240**	5632	378	894.7	5	98.84	0.78

Table S3. Growth conditions and media for the cultivation of bioassay test organisms.

**Test Organism**	**Liquid Medium**	**Solid Medium**	**Temperature**
Bacillus subtilis	TSB	LB	28 °C
Staphylococcus carnosus	TSB	LB	37 °C
Escherichia coli	LB	LB	37 °C
Saccharomyces cerevisiae	YPD	YPD	28 °C
Pseudomonas fluorescens	LB	LB	28 °C

Table S4. Secondary metabolites detected in the extracts of *Streptomyces* spp. from Edelweiss rhizosphere using LC-MS.

#	Strain	Rt [min]	*m/z* [M+H]+	[M+2H]2+	[M+Na]+	Sum formula (proposed)	*m/z* calcd.	Δ*m/z* [ppm]	Tentative ID	BGC	GNPS	The Natural Products Atlas	Comment
**1**	many	4.0	247.1290			C10H18N2O5	247.1288	-0.7	Gaburedin D			NPA028620	
**2**	many	6.2	261.1445			C11H20N2O5	261.1445	-0.2	Gaburedin C			NPA028619	
**3**	many	6.6	261.1445			C11H20N2O5	261.1445	0.1	Gaburedin B			NPA028618	
**4**	many	7.5	295.1290			C14H18N2O5	295.1288	-0.6	Gaburedin A			NPA028617	
**5**	RLA016	3.8	248.0919			C13H13NO4	248.0917	-0.7	Potentially new NP				λmax = 294 nm
**6**	RLA016	9.5	561.3604			C25H48N6O8	561.3606	0.5	Desferrioxamine B	1.14	CCMSLIB00000848497	NPA009012	
**7**	RLA016	11.5	587.3398			C26H46N6O9	587.3399	0.1	Desmethylenylnocardamine	1.14	CCMSLIB00000848941	NPA002285	
**8**	RLA016	12.4	601.3560			C27H48N6O9	601.3556	-0.7	Desferrioxamine E	1.14	CCMSLIB00000846839	NPA024674	
**9**	RLA016	13.3	169.0862			C9H12O3	169.0859	-1.4	Surugapyrone A or isomer			NPA011938	
**10**	RLA016	16.0	265.1438			C15H20O4	265.1434	-1.2	Spirodionic acid		CCMSLIB00000855784	NPA012120	
**11**	RLA016	22.8		1081.9337		C97H131N23O26S4	1081.9332	-0.5	Siamycin I/MS-271/NP-06	1.12			CLGVGSCNDFAGCGYAIVCFW
**12**	RLA051	6.4	242.0655			C10H11NO6	242.0659	1.5	*N*-(2,3-Dihydroxybenzoyl)serine	1.4		NPA009062	
**13**	RLA051	10.8	212.0914			C10H13NO4	212.0917	1.7	Potentially new NP				
**14**	RLA051	10.9	287.2074			C13H26N4O3	287.2078	1.2	*N*-Heptanoyl-arginine		CCMSLIB00010011098		
**15**	RLA051	11.0	252.0862			C12H13NO5	252.0866	1.8	Potentially new NP				λmax = 286 nm
**16**	RLA051	12.1	465.1136			C20H20N2O11	465.1140	0.8	*N*-(2,3-Dihydroxybenzoyl)serine dimer	1.4		NPA000504	
**17**	RLA051	18.3	229.1332			C14H16N2O	229.1335	1.3	Phevalin	1.1 (NRPS)		NPA003113	
**18**	RLA051	20.0	243.1489			C15H18N2O	243.1492	1.0	PZN11 (Pheisoleucin)	1.1 (NRPS)			
**19**	RLA051	21.0	655.2758	328.1418		C36H38N4O8	655.2762	0.7	Coproporphyrin		CCMSLIB00003138722		
**20**	RLA051	23.6	432.2167			C27H29NO4	432.2169	0.6	Piceamycin	1.13 (T1PKS)		NPA009950	several isomers detected
**21**	RLA063	5.0	426.1258			C16H19N5O9	426.1256	-0.7	Aureonucleomycin				
**22**	RLA063	7.9-12.0		303.1796		C27H44N10O6	303.1795	-0.3	Antipain (CHO)	2.3 (NRPS)		NPA021186	several congeners detected
**23**	RLA063	8.5	252.1815			C12H21N5O	252.1819	1.4	Arglecin	2.3 (NRPS)		NPA011039	
**24**	RLA063	8.6	366.1775			C16H23N5O5	366.1772	-0.7	Metabolite KF77-AG6	2.3 (NRPS)			
**25**	RLA063	8.8	252.1815			C12H21N5O	252.1819	1.4	Arglecin isomer (Leu-->Ile)	2.3 (NRPS)			
**26**	RLA063	9.1	349.1219			C17H20N2O4S	349.1217	-0.6	Coelimycin P1	2.6 (T1PKS)		NPA020344	
**27**	RLA063	10.0-13.6	427.3025			C20H38N6O4	427.3027	0.5	Leupeptin (CHO)	2.3 (NRPS)	CCMSLIB00003137883	NPA017329	several congeners detected
**28**	RLA063	10.5	194.1180			C11H15NO2	194.1176	-2.1	*N*-Acetylphenylalaninol	2.3 (NRPS)			
**29**	RLA063	10.6-14.4	441.3183			C21H40N6O4	441.3184	0.1	Leupeptin Pr-LL (CHO)	2.3 (NRPS)	CCMSLIB00000840429	NPA013087	
**30**	RLA063	11.6	429.3181			C20H40N6O4	429.3184	0.8	Reduced leupeptin (CH2OH)	2.3 (NRPS)			
**31**	RLA063	11.8	443.2973			C20H38N6O5	443.2976	0.9	Oxidiced leupeptin (COOH)	2.3 (NRPS)			
**32**	RLA063	12.1	464.2612			C21H33N7O5	464.2616	0.8	Unknown congener	2.3 (NRPS)			
**33**	RLA063	12.8	443.3336			C21H42N6O4	443.3340	1.0	Reduced leupeptin Pr-LL (CH2OH)	2.3 (NRPS)			
**34**	RLA063	13.4	429.2706			C20H36N4O6	429.2708	0.3	Unknown congener	2.3 (NRPS)			
**35**	RLA063	14.1-17.2	596.3184			C30H41N7O6	596.3191	1.1	MAPI (CHO)	2.3 (NRPS)			several congeners detected
**36**	RLA063	15.9	598.3342			C30H43N7O6	598.3348	0.9	Mer-N5075-A (CH2OH)	2.3 (NRPS)	CCMSLIB00000577812	NPA019726	
**37**	RLA063	15.9	584.3186			C29H41N7O6	584.3191	0.9	Unknown congener	2.3 (NRPS)			
**38**	RLA063	16.0	612.3136			C30H41N7O7	612.3140	0.6	Oxidized MAPI (COOH)	2.3 (NRPS)			
**39**	RLA063	16.7	584.3185			C29H41N7O6	584.3191	1.0	Unknown congener	2.3 (NRPS)			
**17**	RLA063	18.3	229.1338			C14H16N2O	229.1335	-1.3	Phevalin	2.3 (NRPS)		NPA003113	
**40**	RLA102	2.8	229.1293			C9H16N4O3	229.1295	1.0	Potentially new NP				
**41**	RLA103	2.5	404.1926	202.6001		C19H25N5O5	202.6001	-0.2	Melanostatin/BMY 28566				
**42**	RLA103	5.6-9.7	507.2271	254.1173					Potentially new NP				several isomers detected
**43**	RLA103	5.8	513.2017	257.1050					Potentially new NP				
**44**	RLA103	6.2	421.1932			C16H28N4O9	421.1929	-0.7	Schizokinen	1.14 (?)	CCMSLIB00005435751	NPA027582	
**45**	RLA103	6.6	513.2017	257.1049					Potentially new NP				
**46**	RLA103	8.0-10.3	509.2429	255.1253					Potentially new NP				several isomers detected

Table S4. (continued)

#	Strain	Rt [min]	*m/z* [M+H]+	[M+2H]2+	[M+Na]+	Sum formula (proposed)	*m/z* calcd.	Δ*m/z* [ppm]	Tentative ID	BGC	GNPS	The Natural Products Atlas	Comment
**47**	RLA103	9.2	491.2319	246.1198					Potentially new NP				
**48**	RLA103	9.9	491.2324	246.1199					Potentially new NP				
**49**	RLA103	10.9	201.1124		223.0943	C10H16O4	223.0941	-1.2	Nonactic acid congener				
**50**	RLA103	11.2	203.1282		225.1100	C10H18O4	225.1097	-1.4	Nonactic acid		CCMSLIB00004698285	NPA005190	several isomers/congeners detected
**51**	RLA103	11.4	179.1177			C10H14N2O	179.1179	0.9	Le-pyrrolopyrazine A			NPA028352	
**52**	RLA103	11.7	585.3606			C27H48N6O8	585.3606	0.0	Dehydroxynocardamine	1.23	CCMSLIB00000848996	NPA017033	
**53**	RLA103	12.7-14.2	300.1804			C15H25NO5	300.1805	0.4	Hydrolyzed cycloheximide	1.3			
**54**	RLA103	13.2		835.8863					Potentially new RiPP	1.18 (?)			
**55**	RLA103	13.5	217.1437		239.1256	C11H20O4	239.1254	-0.8	Homononactic acid		CCMSLIB00004692465	NPA013702	
**56**	RLA103	14.0-16.2	282.1702			C15H23NO4	282.1700	-0.6	**Cycloheximide isomers**	1.3	CCMSLIB00005778324	NPA012393	several isomers/congeners detected
**57**	RLA103	16.9	294.1336			C15H19NO5	294.1336	-0.1	Phenatic acid A	1.3		NPA006248	
**58**	RLA103	21.5	401.2535		423.2355	C21H36O7	401.2534	-0.3	Bonactin		CCMSLIB00005722319	NPA014740	
**59**	RLA103	21.6	511.2802			C29H38N2O6	511.2803	0.2	Polycyclic tetramate macrolactam	1.8			several isomers detected
**60**	RLA103	22.3	1296.5768						Potentially new NP				
**61**	RLA103	22.4	1225.5403						Potentially new NP				
**62**	RLA103	22.7		1142.5707		C102H162N24O31S2	1142.5712	0.5	AmfS congener	1.16			ATGDhaQVDhaLLVCEYDhaSLDhaVVLCTP
**63**	RLA103	22.9	495.2851			C29H38N2O5	495.2853	0.6	Polycyclic tetramate macrolactam	1.8			several isomers detected
**64**	RLA103	22.9	1168.5181						Potentially new NP				
**65**	RLA103	23.0	513.2958			C29H40N2O6	513.2959	0.2	Polycyclic tetramate macrolactam	1.8			
**66**	RLA103	23.0	509.2644			C29H36N2O6	509.2646	0.4	Polycyclic tetramate macrolactam	1.8			
**67**	RLA103	23.1		1056.5283		C95H150N22O28S2	1056.5288	0.5	AmfS congener	1.16			GDhaQVDhaLLVCEYDhaSLDhaVVLCTP
**68**	RLA103	23.2		1107.0526		C99H157N23O30S2	1107.0527	0.1	AmfS	1.16			TGDhaQVDhaLLVCEYDhaSLDhaVVLCTP
**69**	RLA103	24.3	493.2694			C29H36N2O5	493.2697	0.5	Clifednamide A	1.8	CCMSLIB00005788083	NPA024740	
**70**	RLA103	24.4	497.3005			C29H40N2O5	497.3010	0.9	10-epi-3-deOH-HSAF	1.8		NPA026678	
**71**	RLA103	25.0	1483.6771			C75H94N12O20	1483.6780	0.6	RP-1776/Skyllamycin A	1.21		NPA003971	
**72**	RLA103	36.7	723.4321		745.4136	C39H62O12	745.4133	-0.3	Nonactin congener				
**73**	RLA103	-	737.4489		759.4292	C40H64O12	759.4290	-0.3	Nonactin		CCMSLIB00010109074		several congeners detected
**74**	RLA103	-	751.4624		773.4443	C41H66O12	773.4446	0.5	Monactin		CCMSLIB00000851863	NPA005516	
**75**	RLA103	-	765.4788		787.4607	C42H68O12	787.4603	-0.5	Isodinactin				
**76**	RLA103	-	779.4939		801.4757	C43H70O12	801.4759	0.3	Trinactin			NPA006480	
**77**	RLA153	1.8	370.1710			C14H27NO10	370.1708	-0.5	Unidentified aminodisaccharide				
**78**	RLA153	4.6	645.3310			C25H44N10O10	645.3315	0.7	Desferri-peucechelin	2.8 (NRPS)		NPA032825	
**79**	RLA153	6.4	673.3623			C27H48N10O10	673.3628	0.6	Salinichelin C	2.8 (NRPS)		NPA028403	several congeners detected
**80**	RLA153	8.0	196.0607			C9H9NO4	196.0604	-1.3	*N*-Acetyl-5-aminosalicylic acid		CCMSLIB00000841943		
**23**	RLA153	8.5	252.1819			C12H21N5O	252.1819	-0.2	Arglecin			NPA011039	
**81**	RLA153	8.7		408.7034					Potentially new NP				
**25**	RLA153	8.8	252.1819			C12H21N5O	252.1819	-0.2	Arglecin isomer (Leu-->Ile)				
**82**	RLA153	8.8		496.7375					Potentially new NP				
**6**	RLA153	9.5	561.3607			C25H48N6O8	561.3606	-0.1	Desferrioxamine B	2.11	CCMSLIB00000848497	NPA009012	
**83**	RLA153	11.8	252.1709			C13H21N3O2	252.1707	-0.8	Streptopyrazinone A			NPA022957	
**19**	RLA153	21.0	655.2767	328.1424		C36H38N4O8	655.2762	-0.7	Coproporphyrin		CCMSLIB00003138722		
**84**	RLA153	21.5	1140.7197			C64H101NO16	1140.7193	-0.3	Linearmycin A	2.8 (T1PKS)		NPA005259	other minor congeners detected
**85**	RLA153	22.4	1166.7353			C66H103NO16	1166.7350	-0.3	Linearmycin B	2.8 (T1PKS)		NPA005208	other minor congeners detected

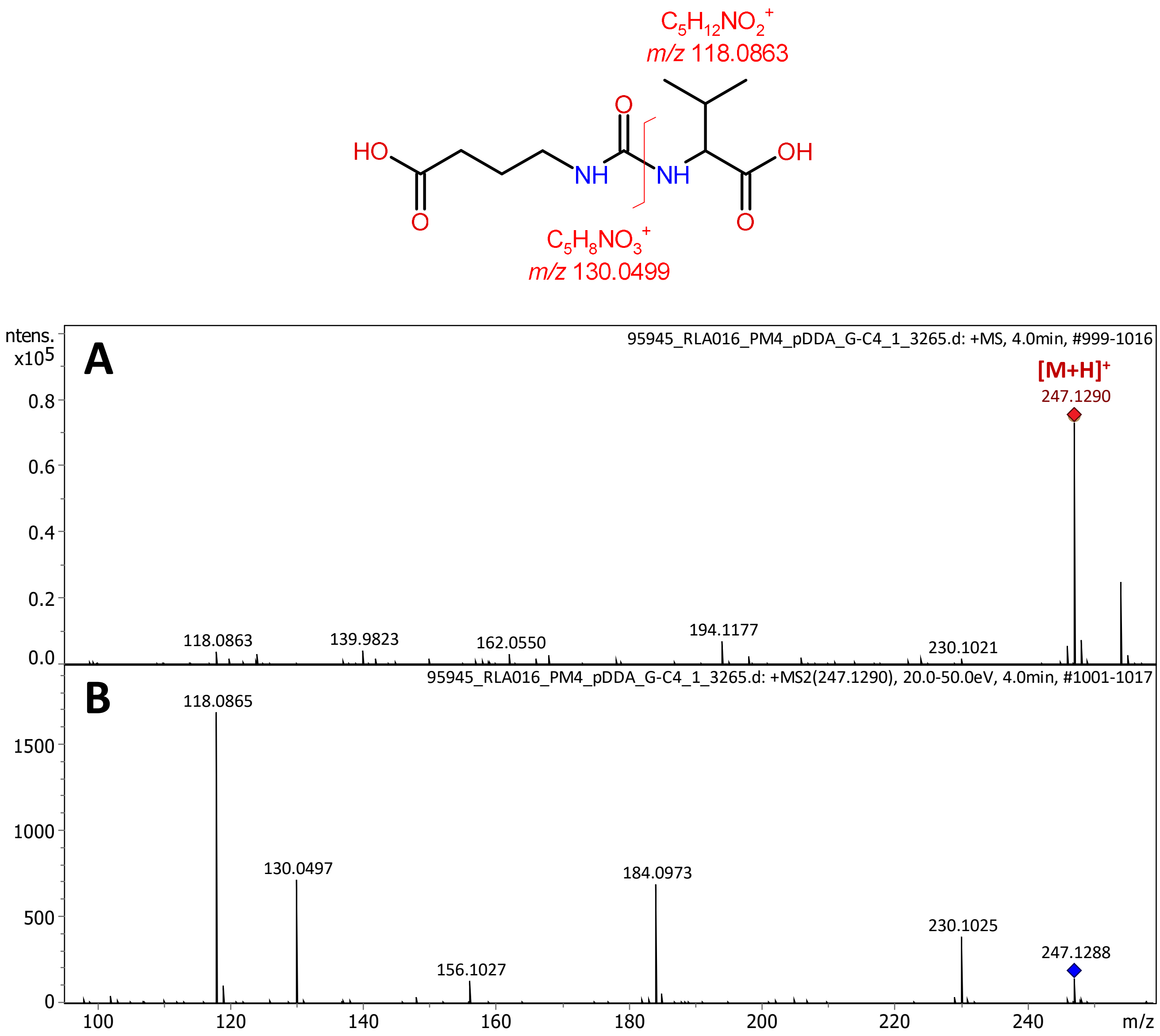

Figure S1. High resolution ESI-Qq-TOF mass spectrum of Gaburedin D (**1**) in strain RLA016 grown in PM4 (A) and high resolution MS/MS spectrum of its $[M+H]^+$ ion (B).

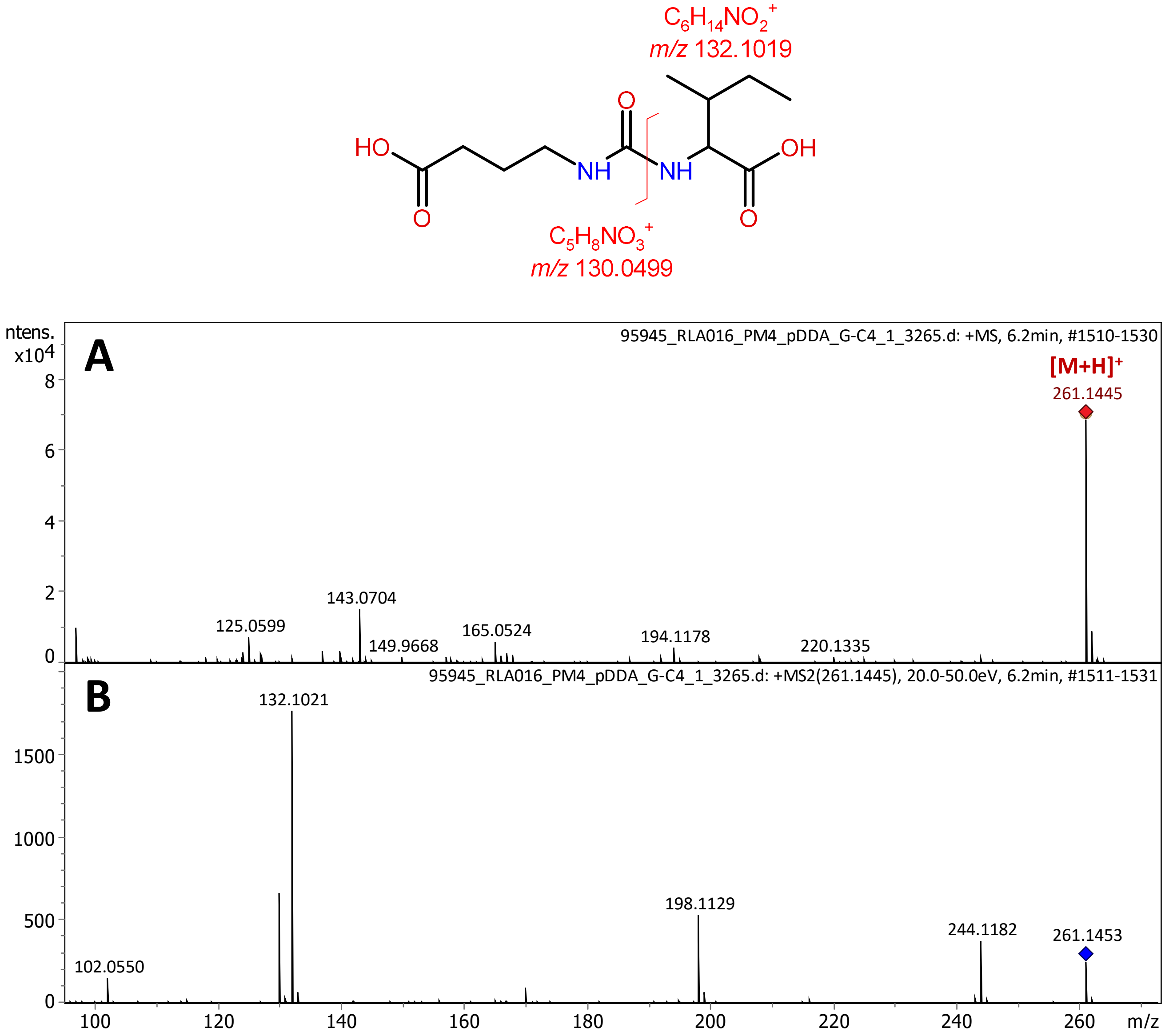

Figure S2. High resolution ESI-Qq-TOF mass spectrum of Gaburedin C (**2**) in strain RLA016 grown in PM4 (A) and high resolution MS/MS spectrum of its $[M+H]^+$ ion (B).

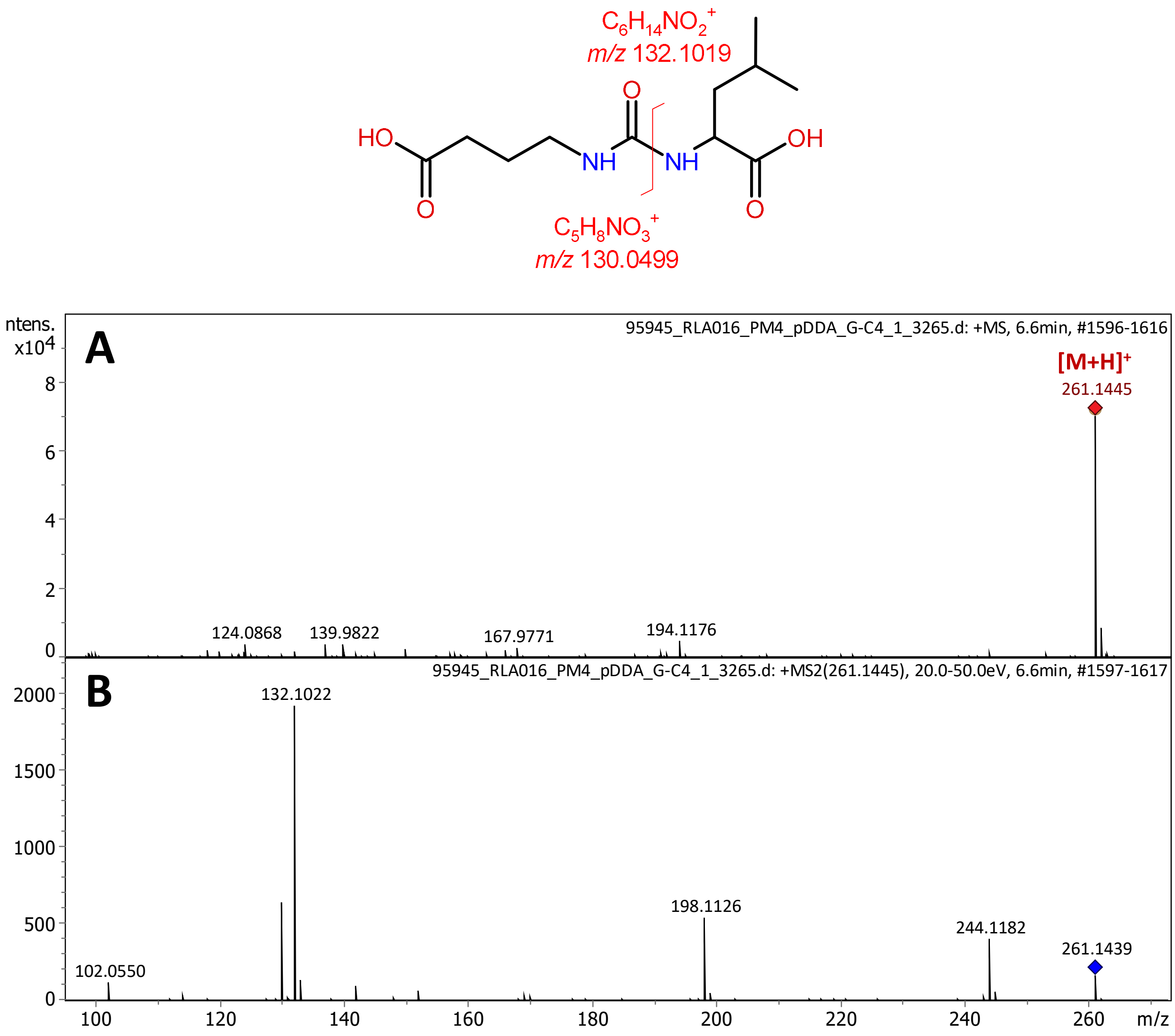

Figure S3. High resolution ESI-Qq-TOF mass spectrum of Gaburedin B (**3**) in strain RLA016 grown in PM4 (A) and high resolution MS/MS spectrum of its $[M+H]^+$ ion (B).

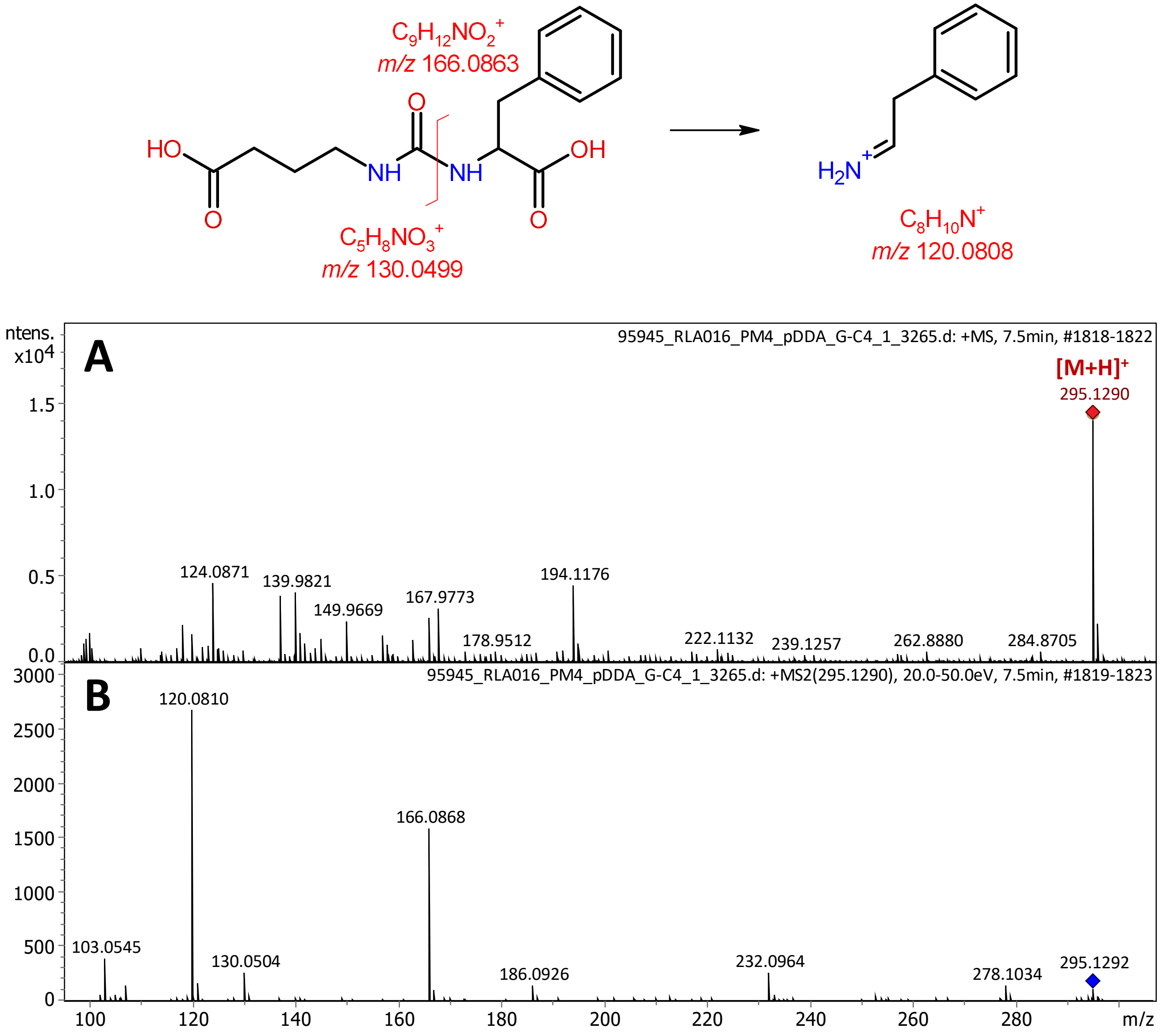

Figure S4. High resolution ESI-Qq-TOF mass spectrum of Gaburedin A (**4**) in strain RLA016 grown in PM4 (A) and high resolution MS/MS spectrum of its $[M+H]^+$ ion (B).

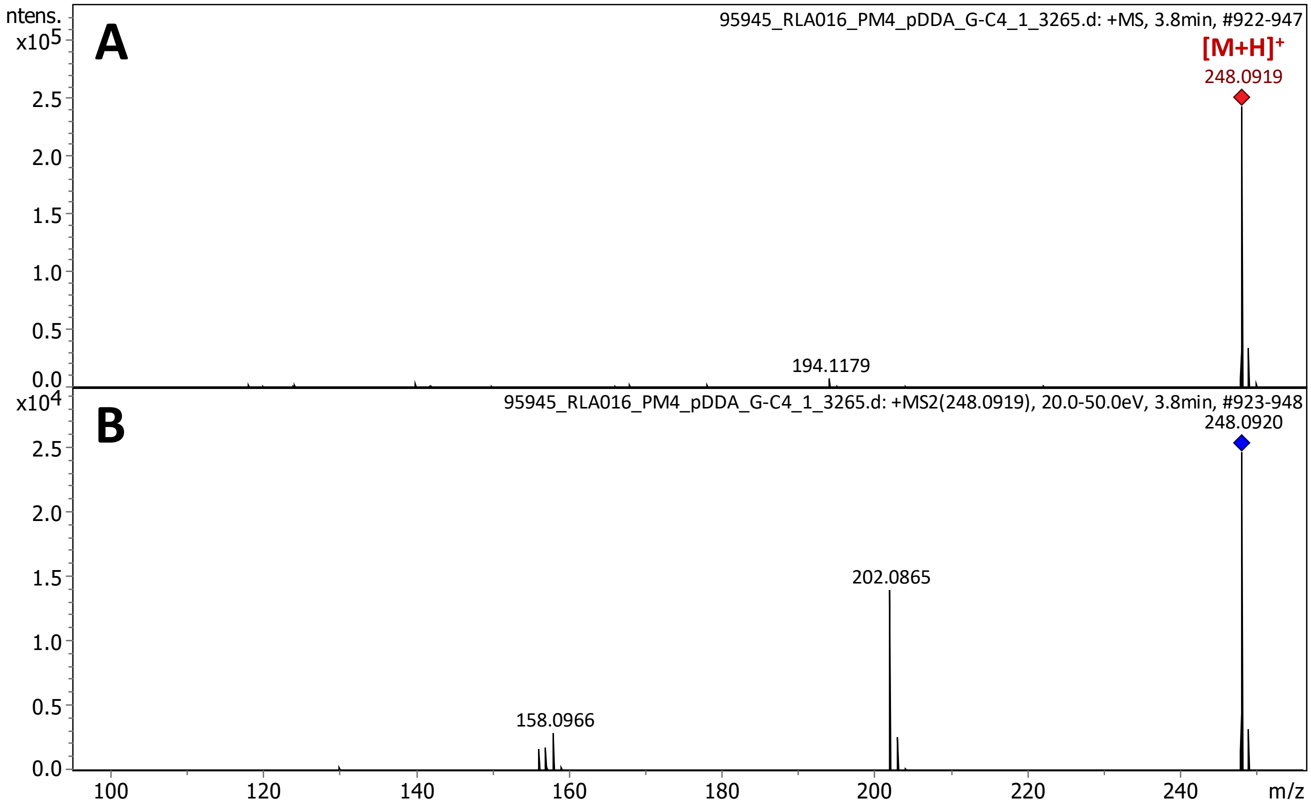

Figure S5. High resolution ESI-Qq-TOF mass spectrum of the potentially new natural product **5** in strain RLA016 grown in PM4 (A) and high resolution MS/MS spectrum of its $[M+H]^+$ ion (B).

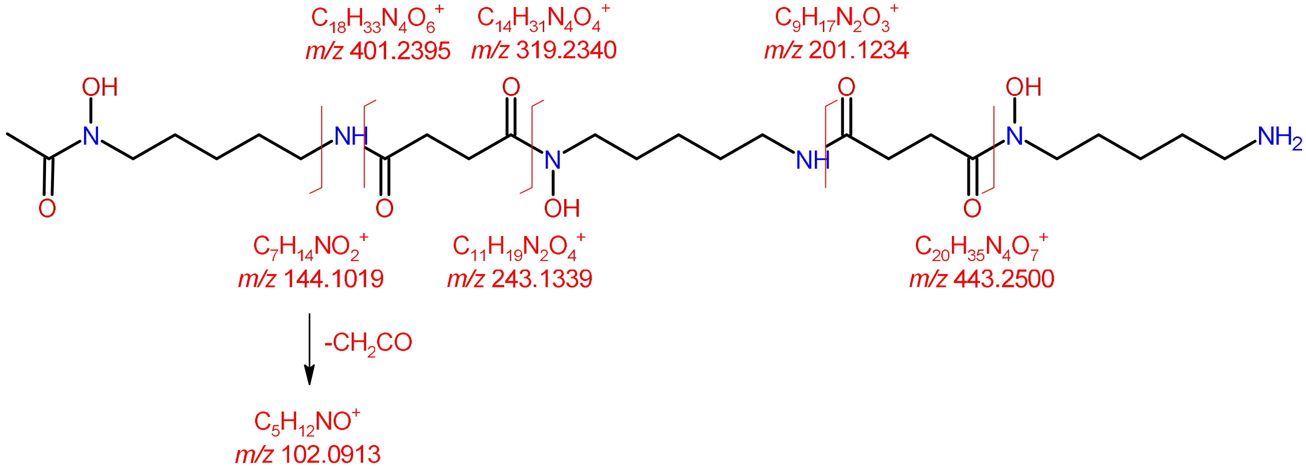

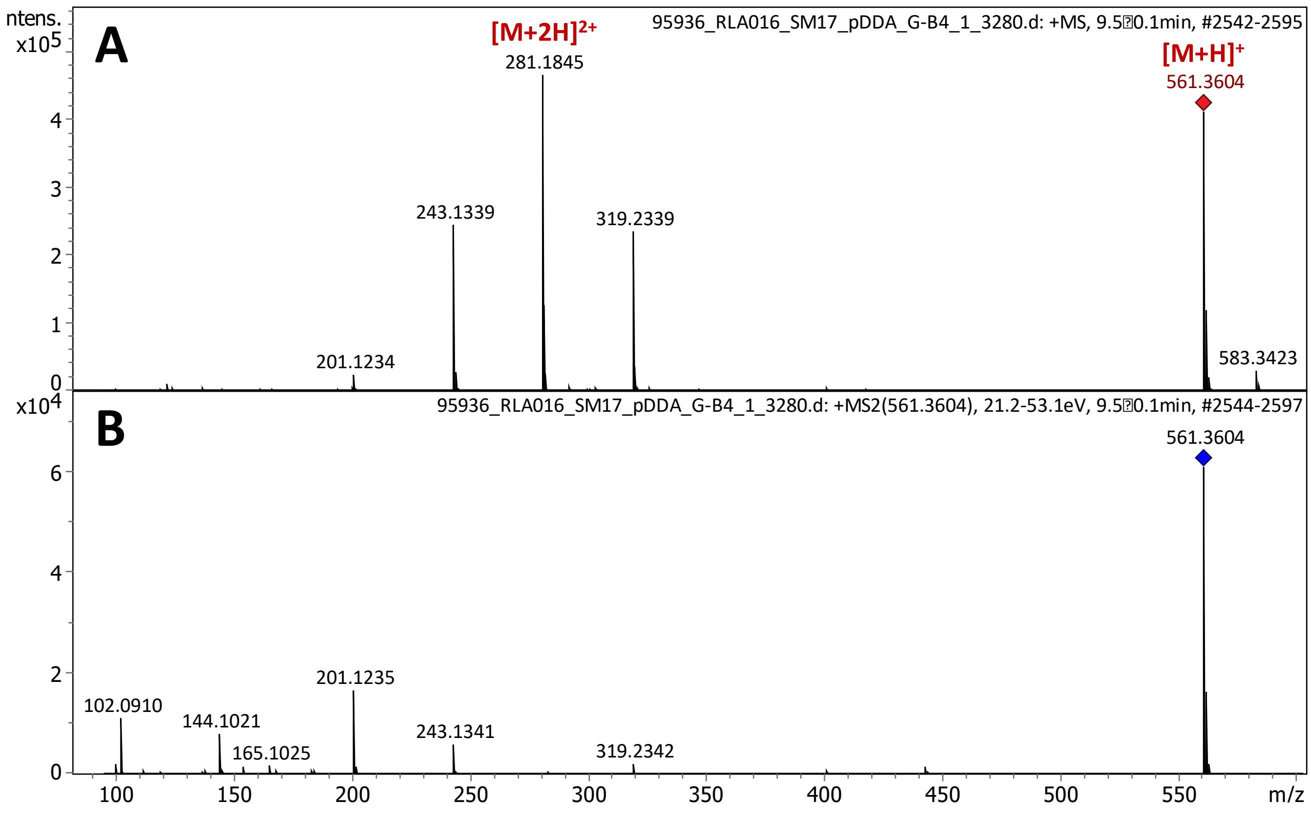

Figure S6. High resolution ESI-Qq-TOF mass spectrum of Desferrioxamine B (**6**) in strain RLA016 grown in SM17 (A) and high resolution MS/MS spectrum of its $[M+H]^+$ ion (B).

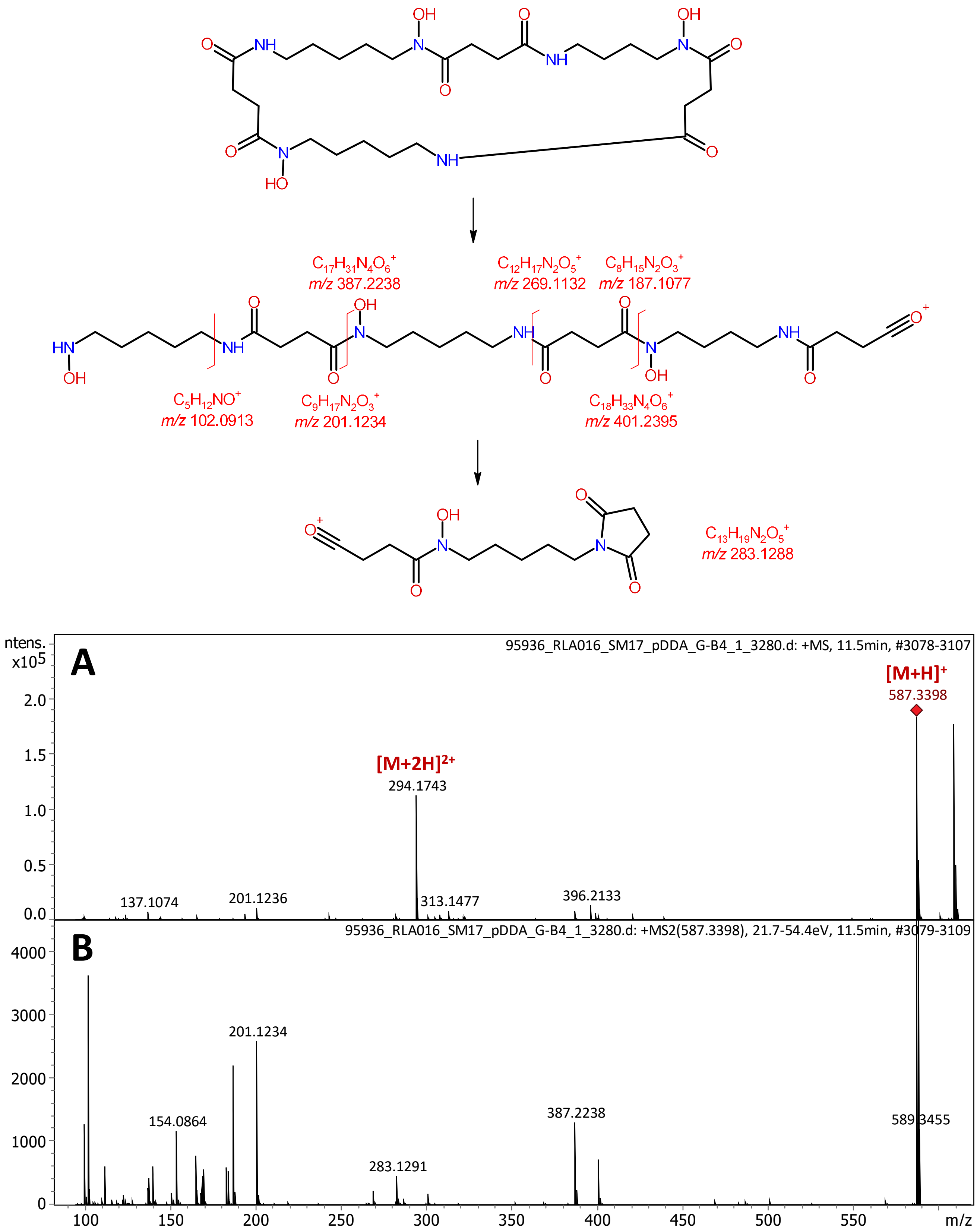

Figure S7. High resolution ESI-Qq-TOF mass spectrum of Desmethylenylnocardamine (**7**) in strain RLA016 grown in SM17 (A) and high resolution MS/MS spectrum of its $[M+H]^+$ ion (B).

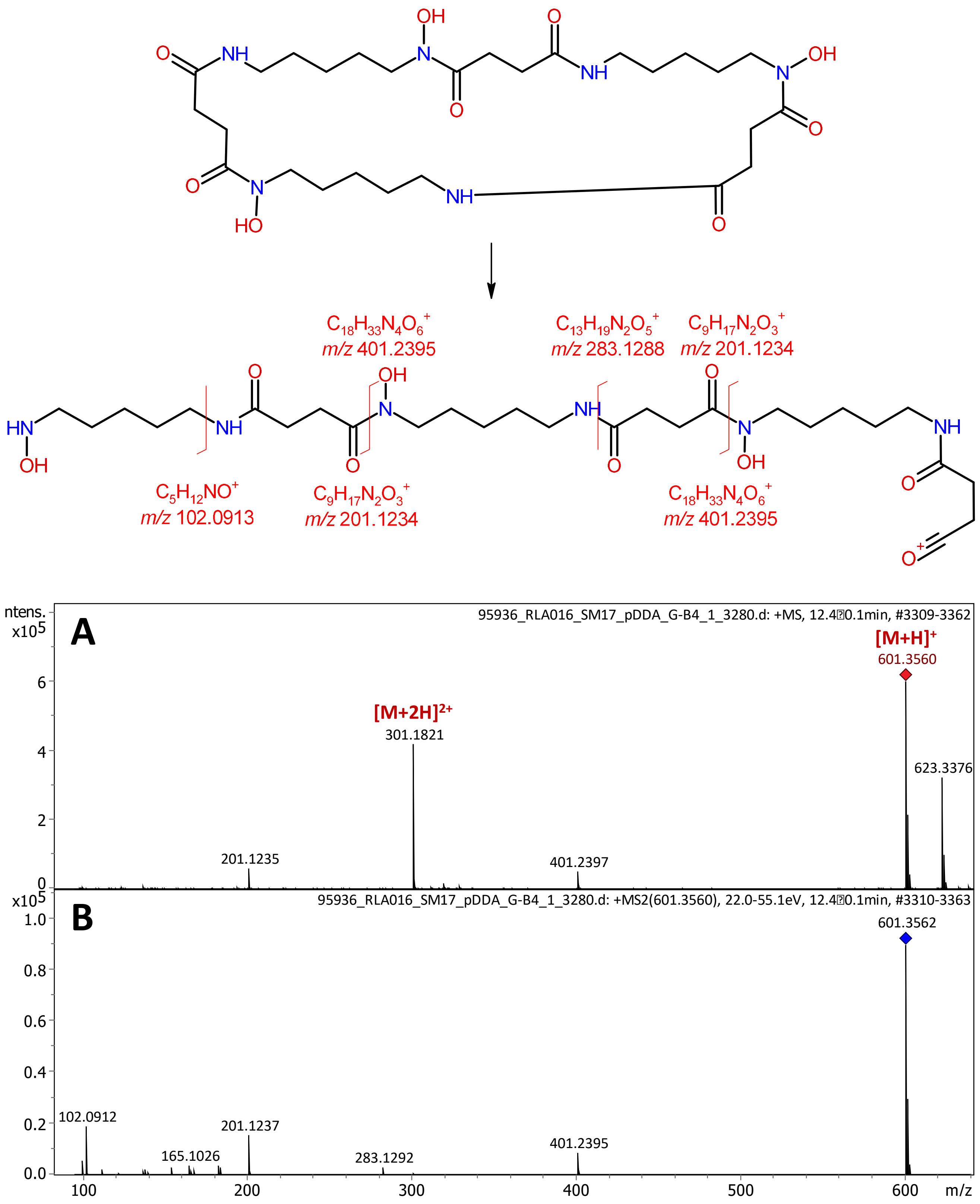

Figure S8. High resolution ESI-Qq-TOF mass spectrum of Desferrioxamine E (**8**) in strain RLA016 grown in SM17 (A) and high resolution MS/MS spectrum of its $[M+H]^+$ ion (B).

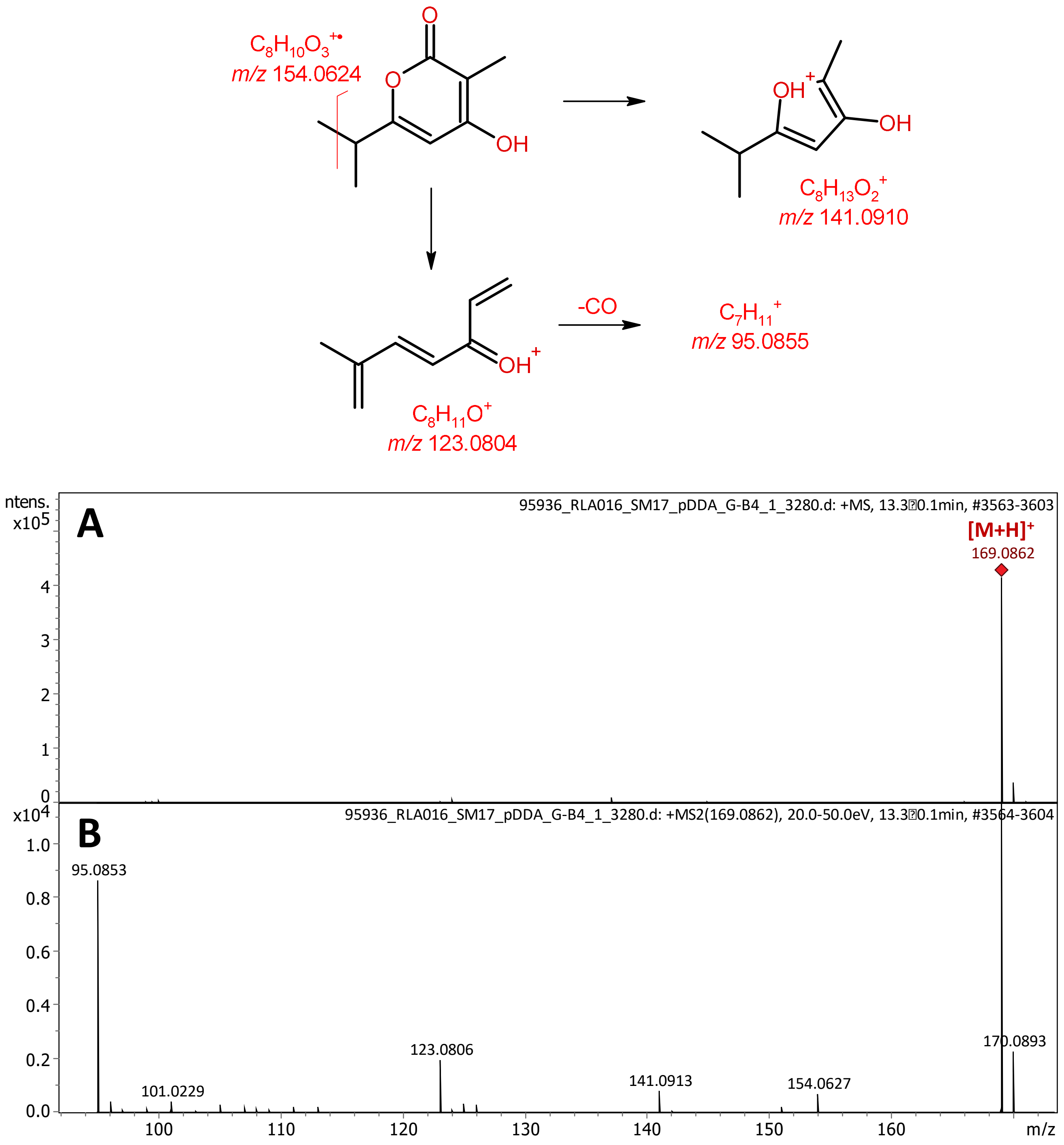

Figure S9. High resolution ESI-Qq-TOF mass spectrum of Surugapyrone A or isomer (**9**) in strain RLA016 grown in SM17 (A) and high resolution MS/MS spectrum of its $[M+H]^+$ ion (B).

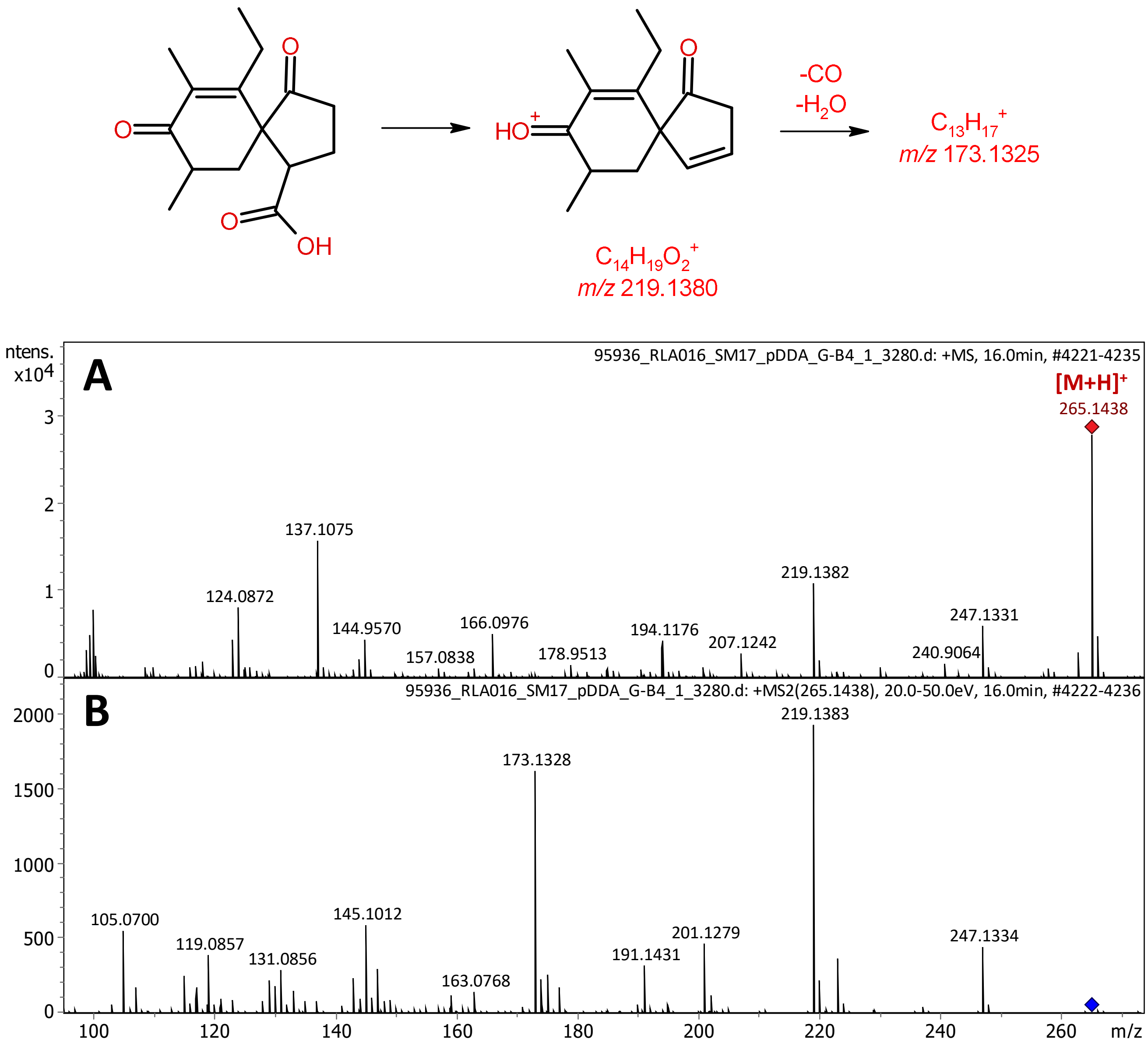

Figure S10. High resolution ESI-Qq-TOF mass spectrum of Spirodionic acid (**10**) in strain RLA016 grown in SM17 (A) and high resolution MS/MS spectrum of its $[M+H]^+$ ion (B).

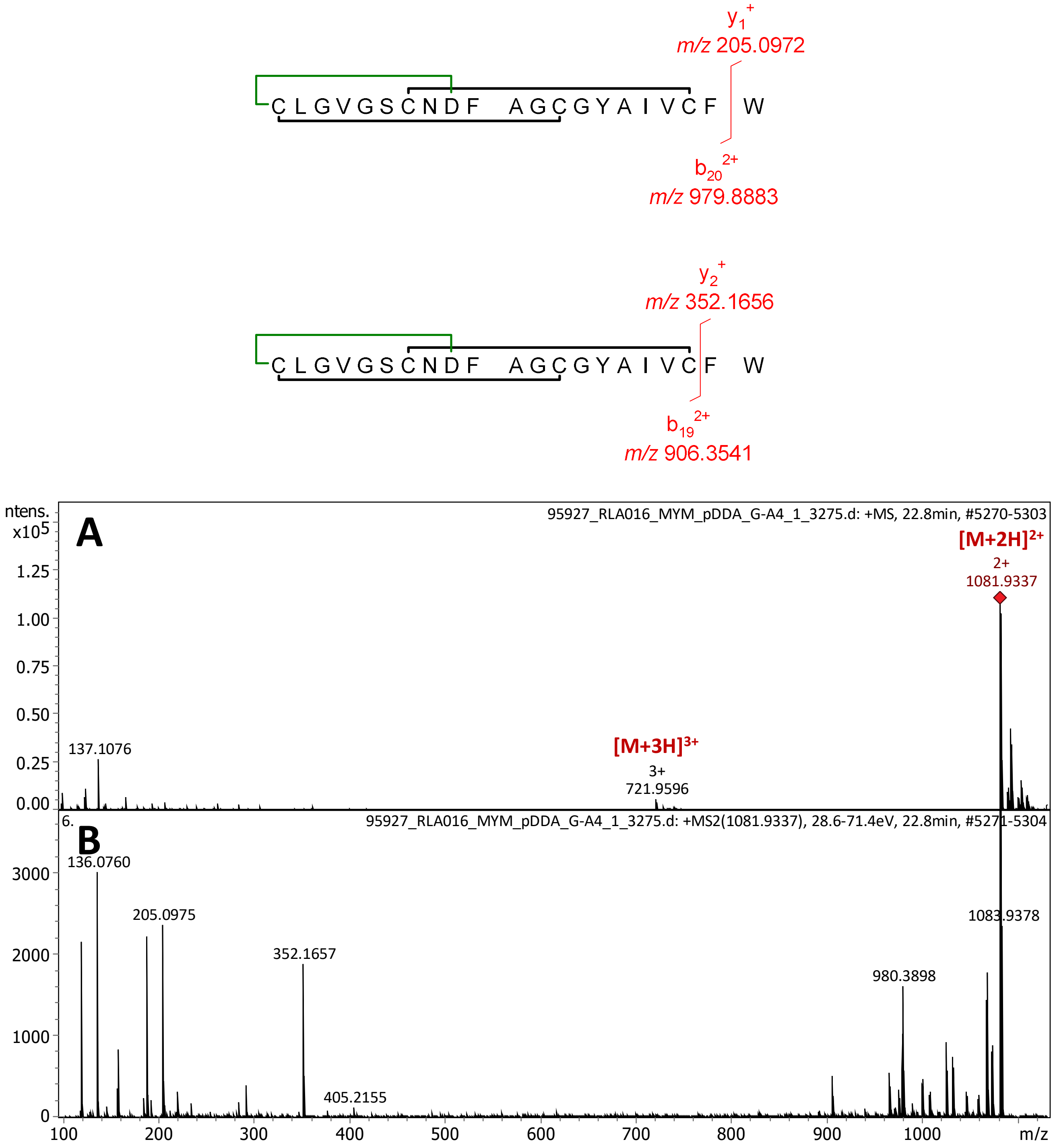

Figure S11. High resolution ESI-Qq-TOF mass spectrum of Siamycin I/MS-271/NP-06 (**11**) in strain RLA016 grown in MYM (A) and high resolution MS/MS spectrum of its $[M+2H]^{2+}$ ion (B).

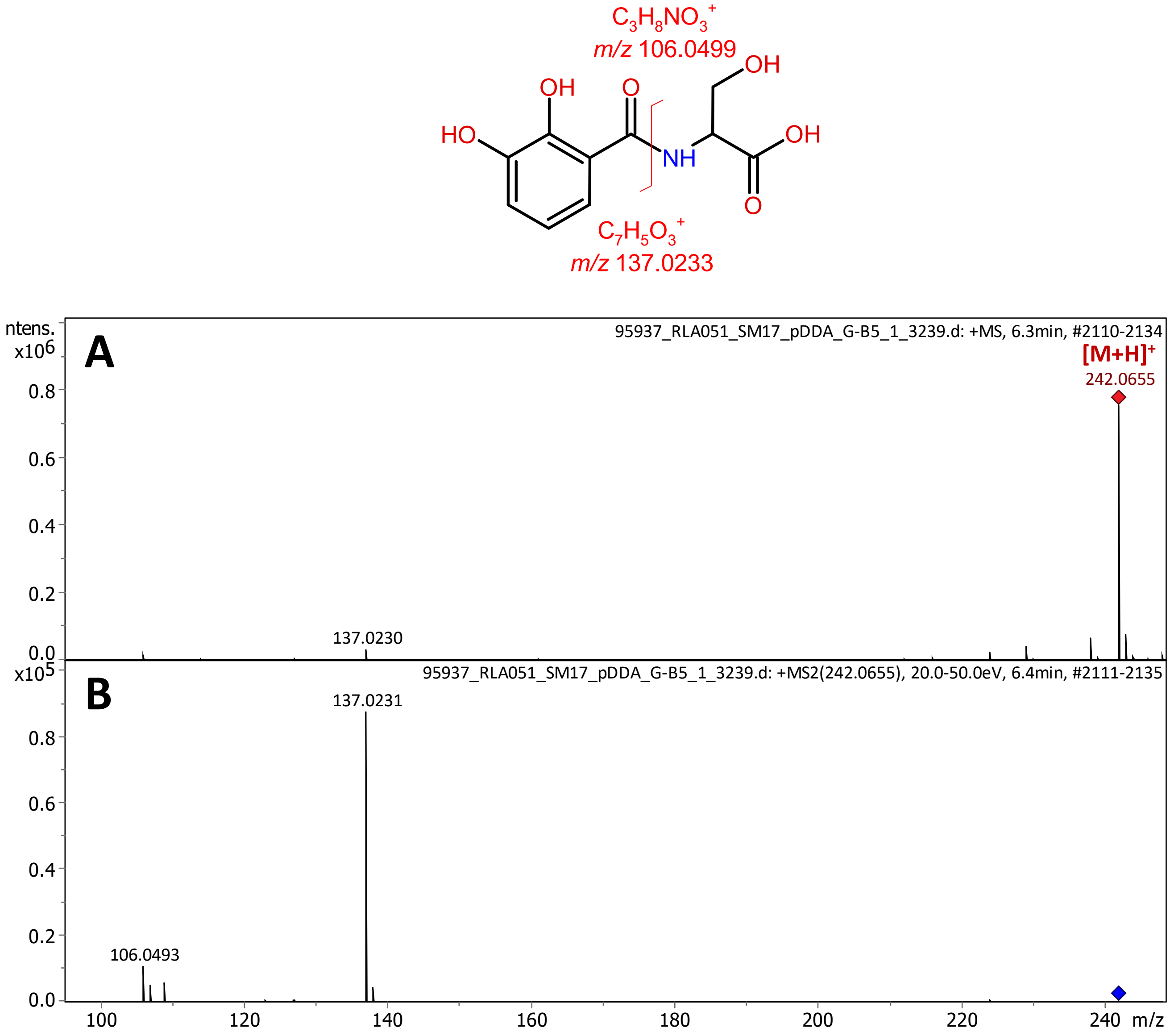

Figure S12. High resolution ESI-Qq-TOF mass spectrum of *N*-(2,3-dihydroxybenzoyl)serine (**12**) in strain RLA051 grown in SM17 (A) and high resolution MS/MS spectrum of its $[M+H]^+$ ion (B).

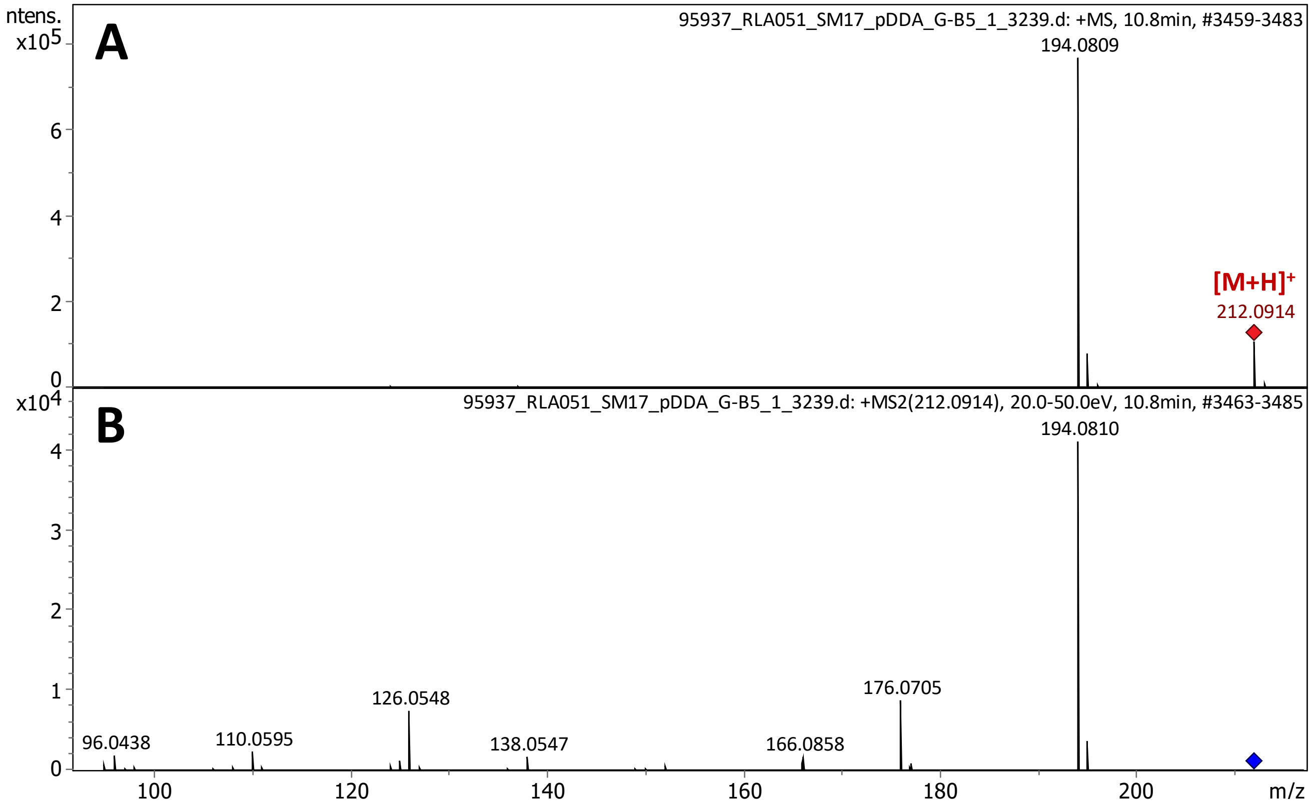

Figure S13. High resolution ESI-Qq-TOF mass spectrum of the potentially new natural product **13** in strain RLA051 grown in SM17 (A) and high resolution MS/MS spectrum of its $[M+H]^+$ ion (B).

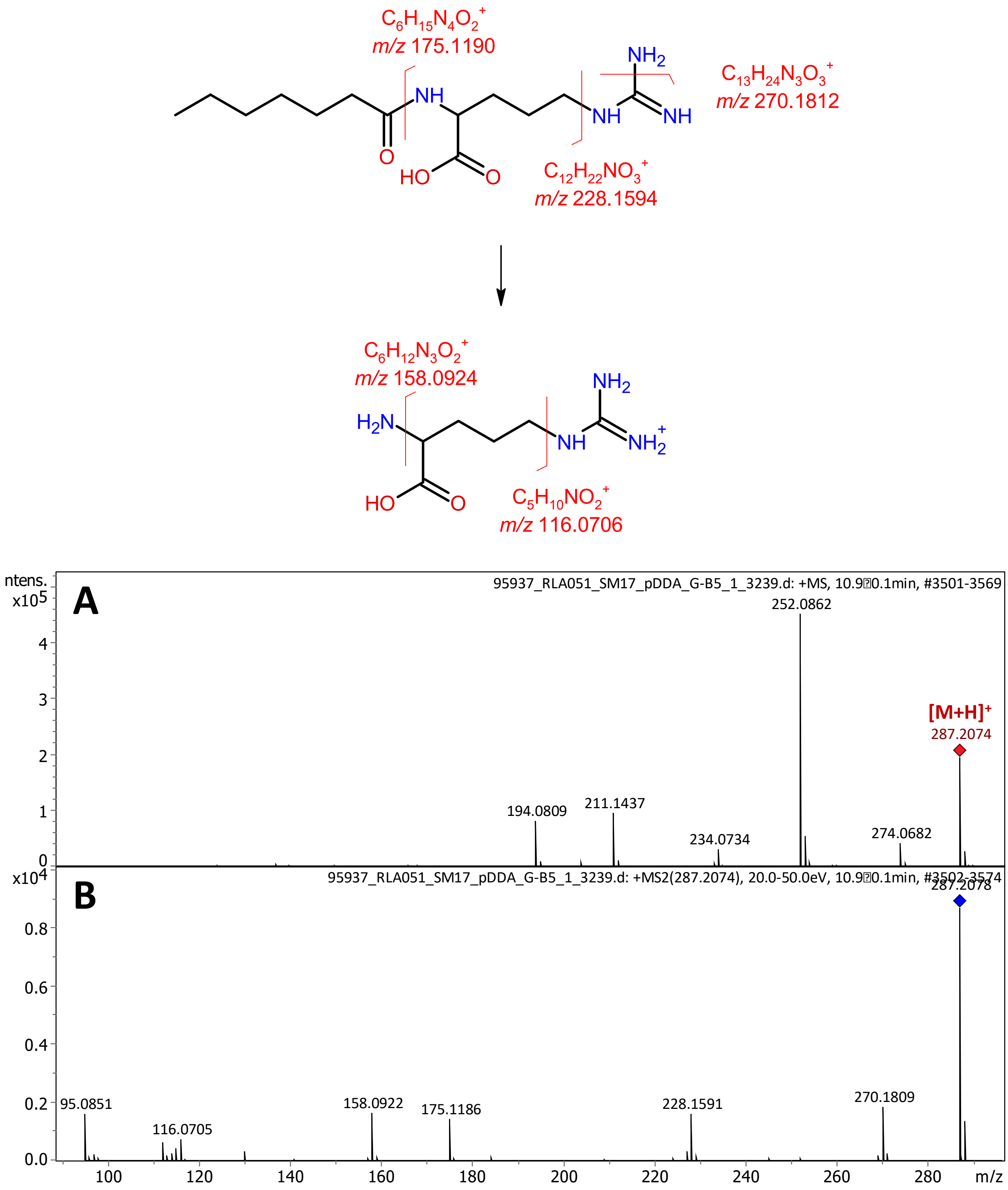

Figure S14. High resolution ESI-Qq-TOF mass spectrum of *N*-heptanoyl-arginine (**14**) in strain RLA051 grown in SM17 (A) and high resolution MS/MS spectrum of its $[M+H]^+$ ion (B).

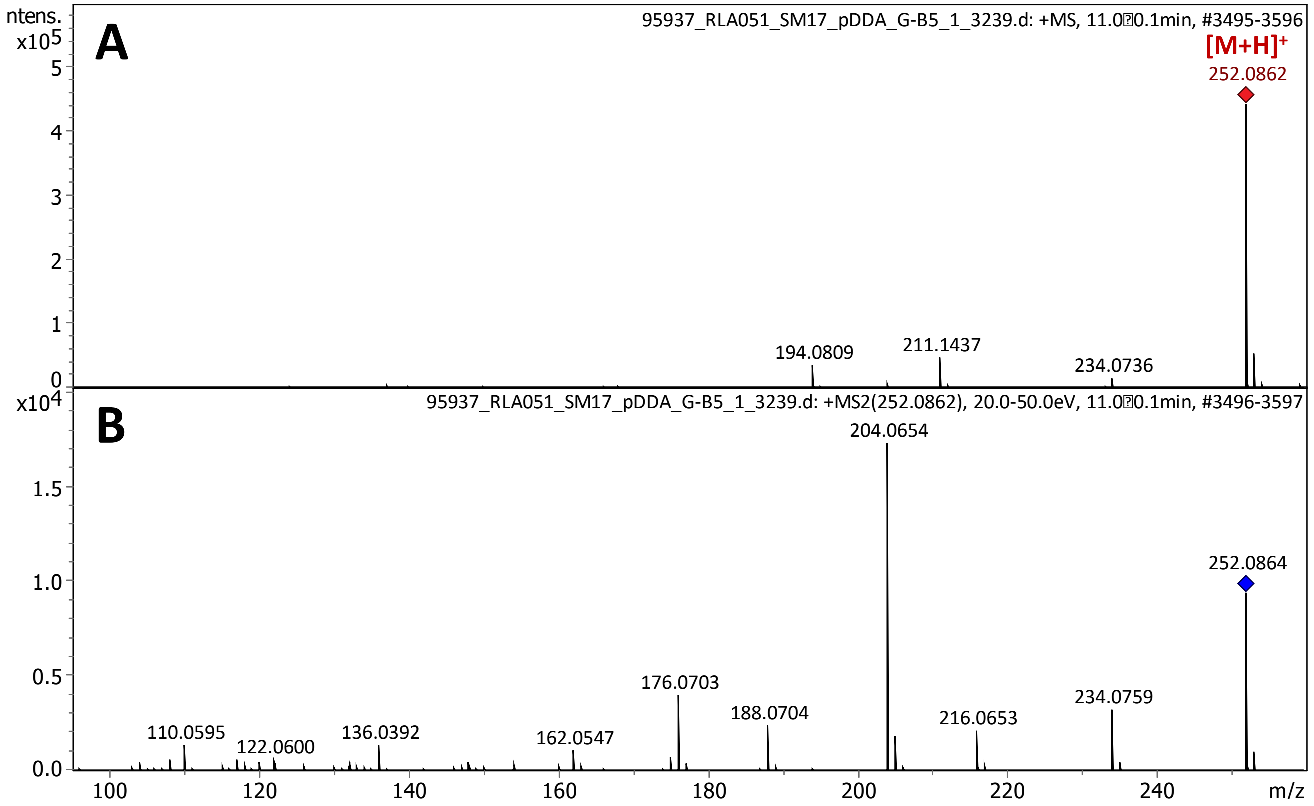

Figure S15. High resolution ESI-Qq-TOF mass spectrum of the potentially new natural product **15** in strain RLA051 grown in SM17 (A) and high resolution MS/MS spectrum of its $[M+H]^+$ ion (B).

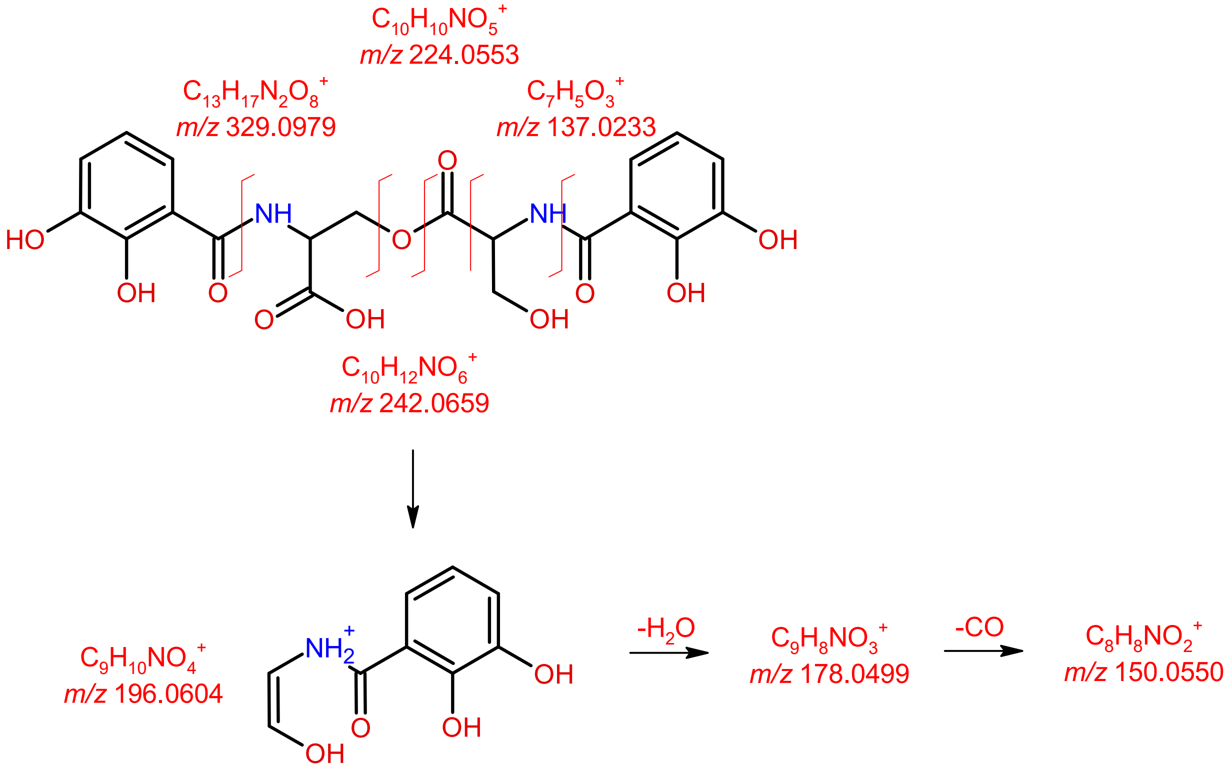

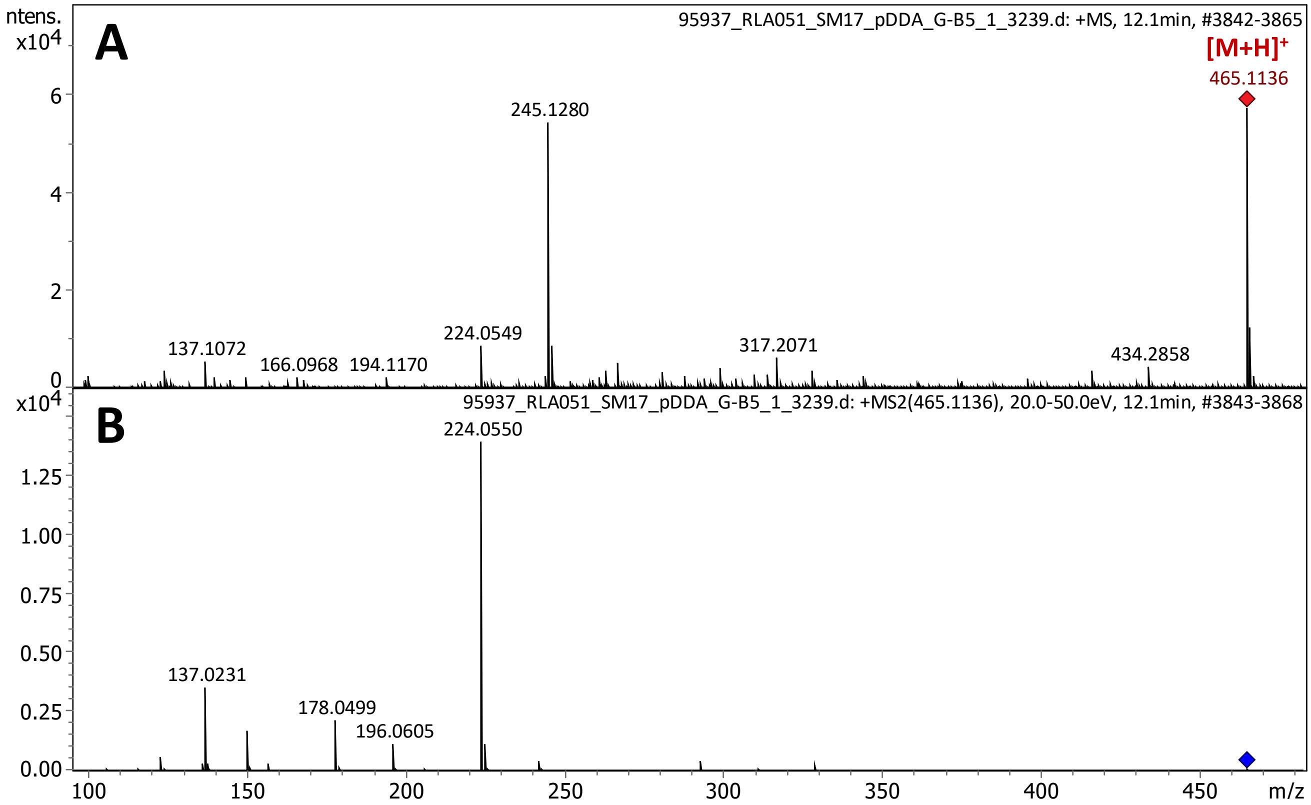

Figure S16. High resolution ESI-Qq-TOF mass spectrum of *N*-(2,3-dihydroxybenzoyl)serine dimer (**16**) in strain RLA051 grown in SM17 (A) and high resolution MS/MS spectrum of its $[M+H]^+$ ion (B).

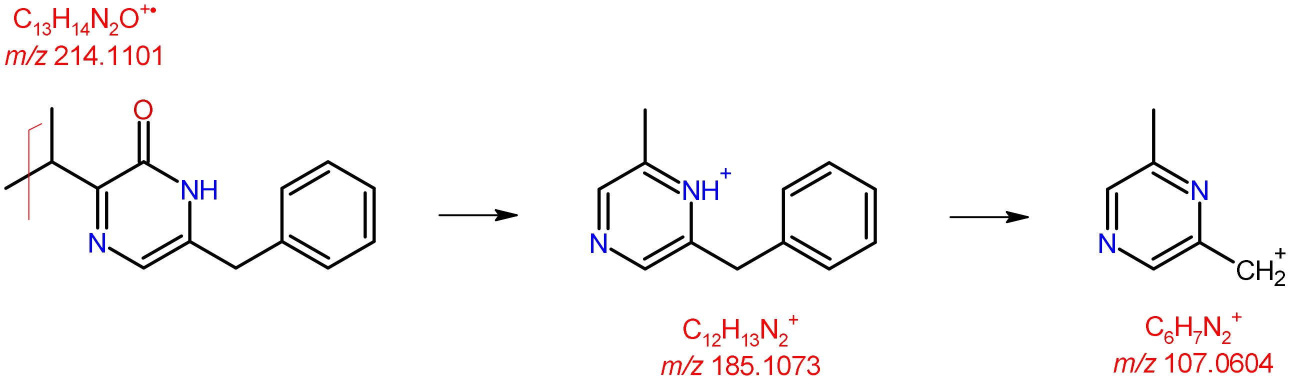

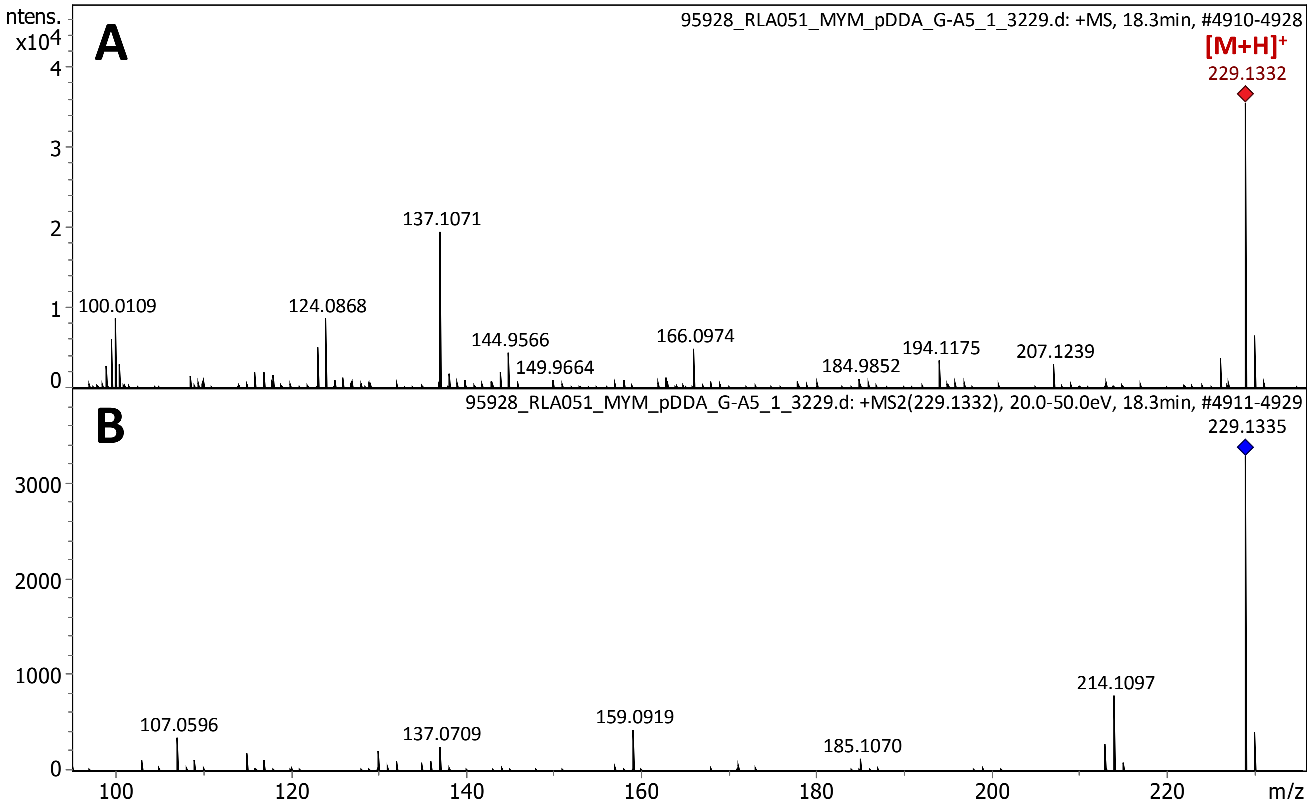

Figure S17. High resolution ESI-Qq-TOF mass spectrum of Phevalin (**17**) in strain RLA051 grown in MYM (A) and high resolution MS/MS spectrum of its $[M+H]^+$ ion (B).

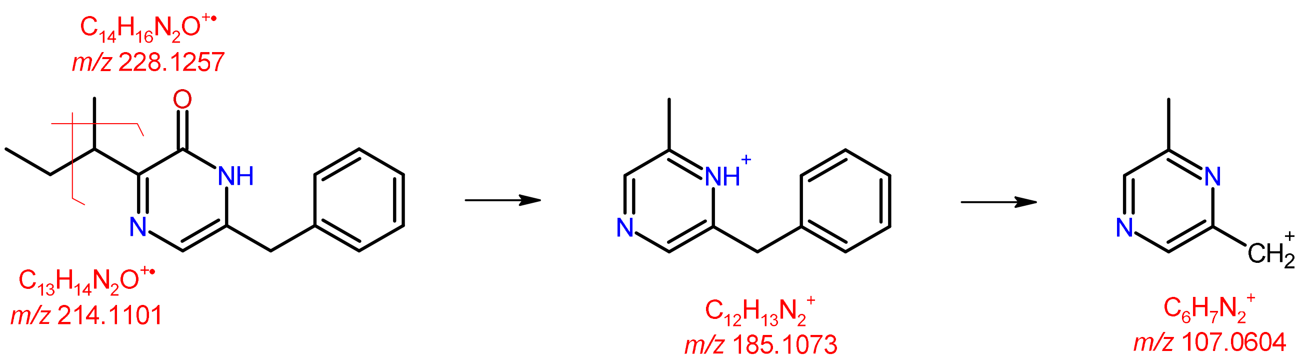

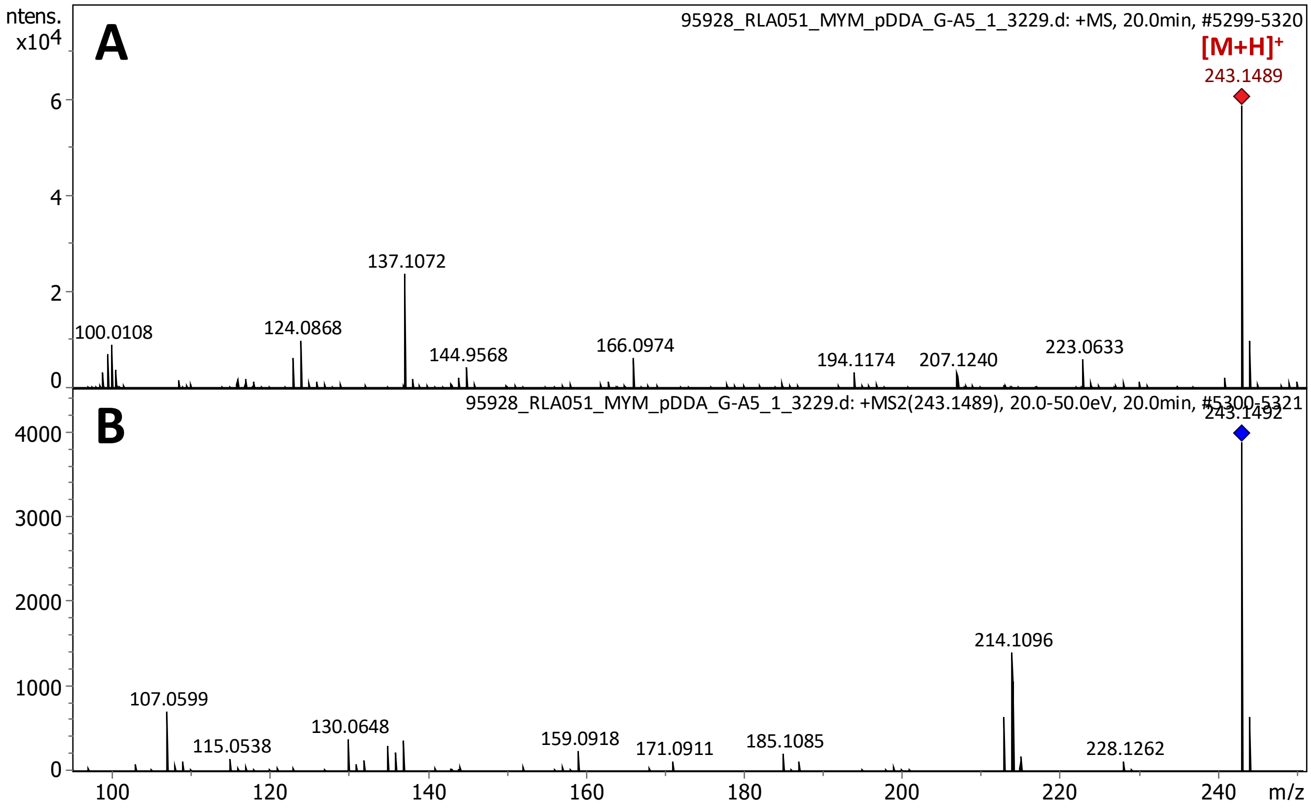

Figure S18. High resolution ESI-Qq-TOF mass spectrum of PZN11/Pheisoleucin (**18**) in strain RLA051 grown in MYM (A) and high resolution MS/MS spectrum of its $[M+H]^+$ ion (B).

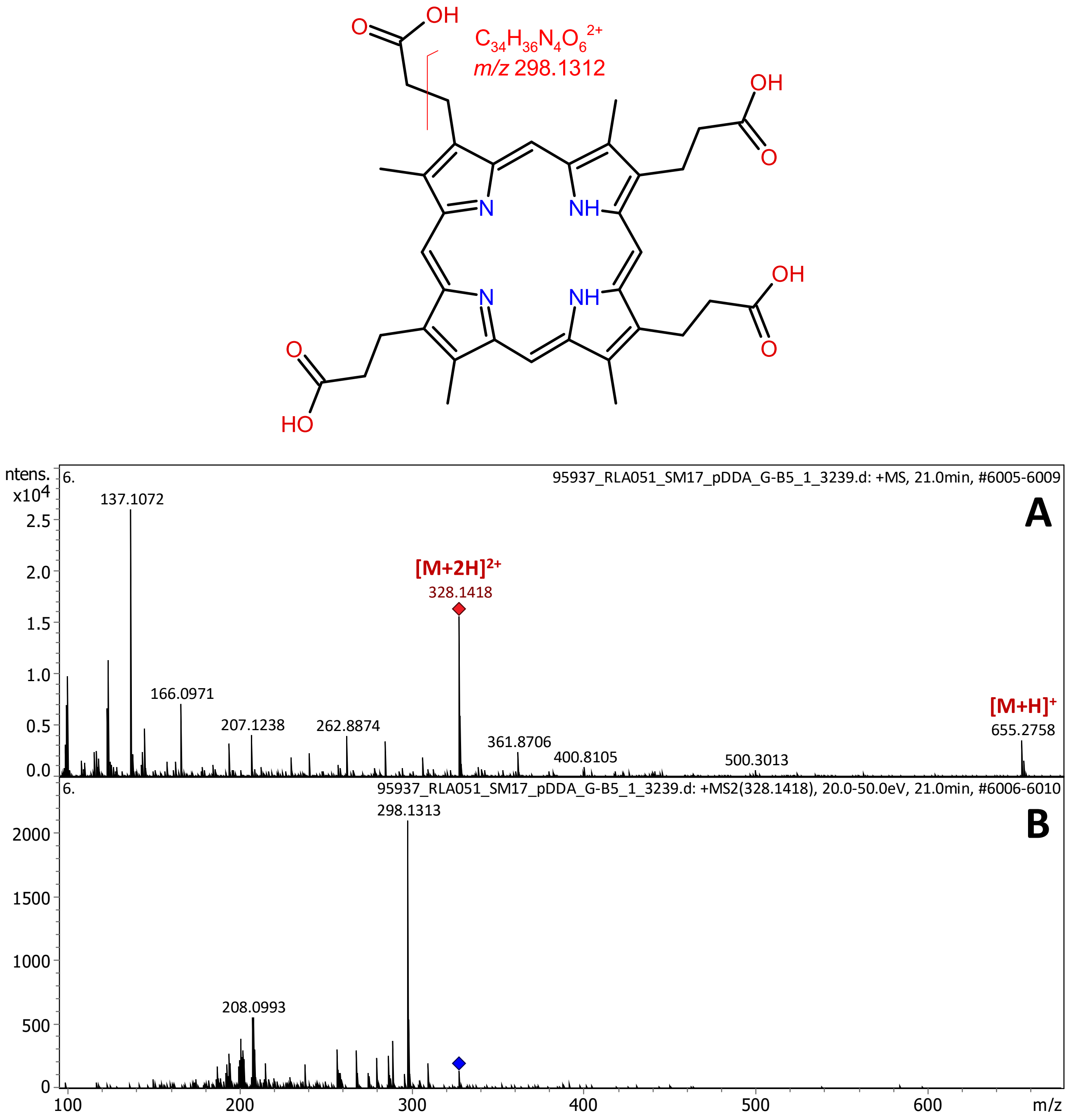

Figure S19. High resolution ESI-Qq-TOF mass spectrum of Coproporphyrin (**19**) in strain RLA051 grown in SM17 (A) and high resolution MS/MS spectrum of its $[M+2H]^{2+}$ ion (B).

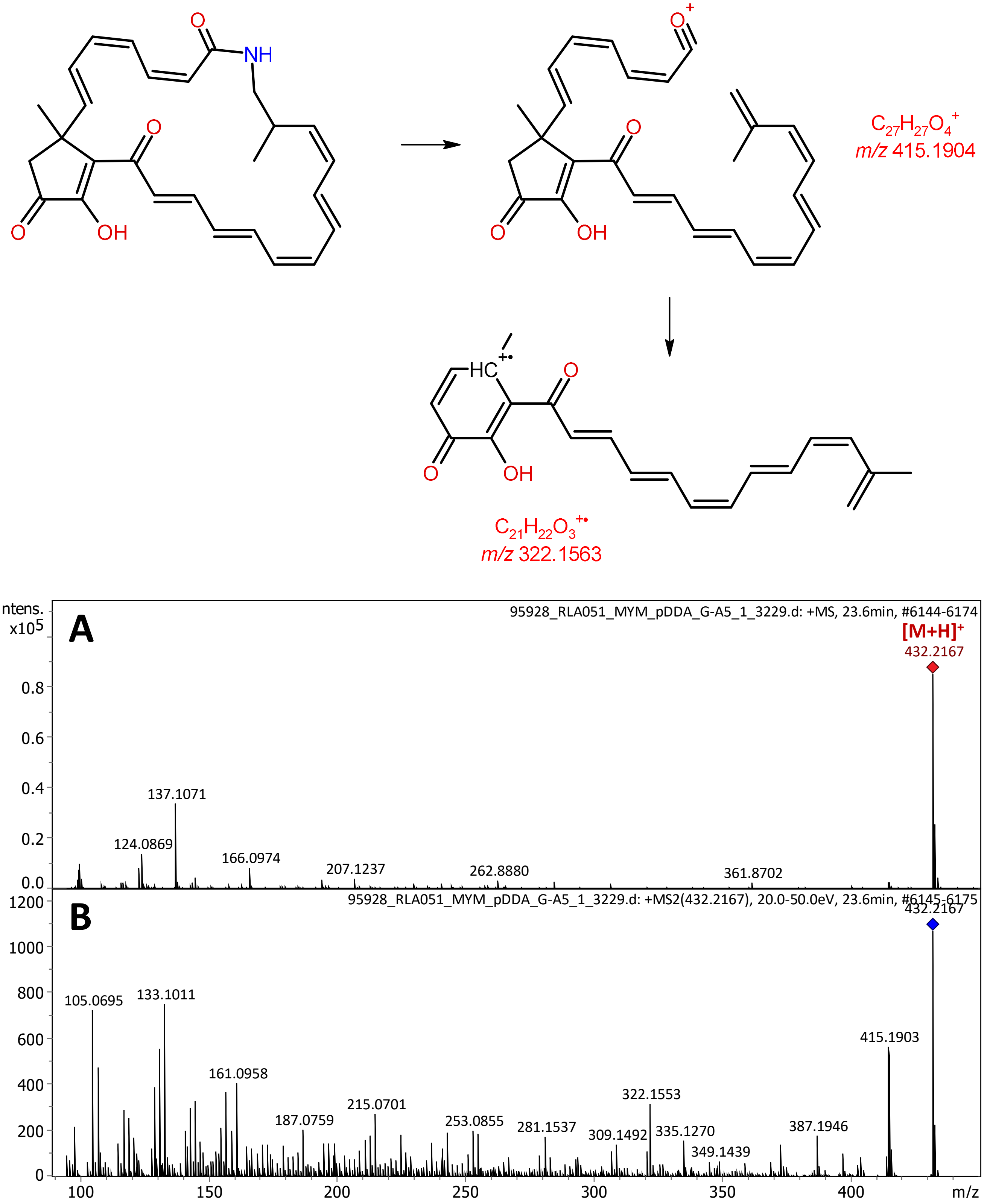

Figure S20. High resolution ESI-Qq-TOF mass spectrum of Piceamycin (**20**) in strain RLA051 grown in MYM (A) and high resolution MS/MS spectrum of its $[M+H]^+$ ion (B).

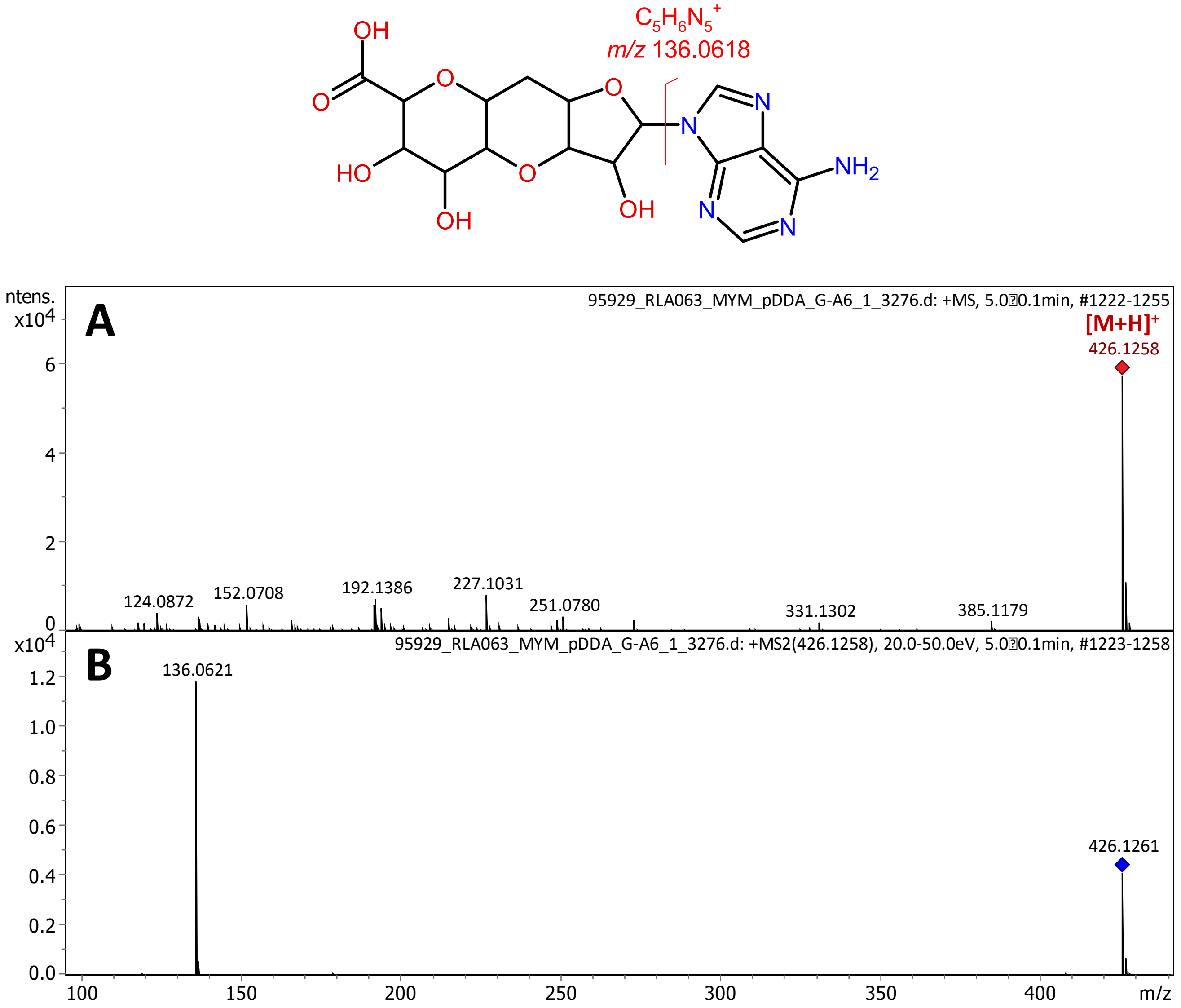

Figure S21. High resolution ESI-Qq-TOF mass spectrum of Aureonucleomycin (**21**) in strain RLA063 grown in MYM (A) and high resolution MS/MS spectrum of its $[M+H]^+$ ion (B).

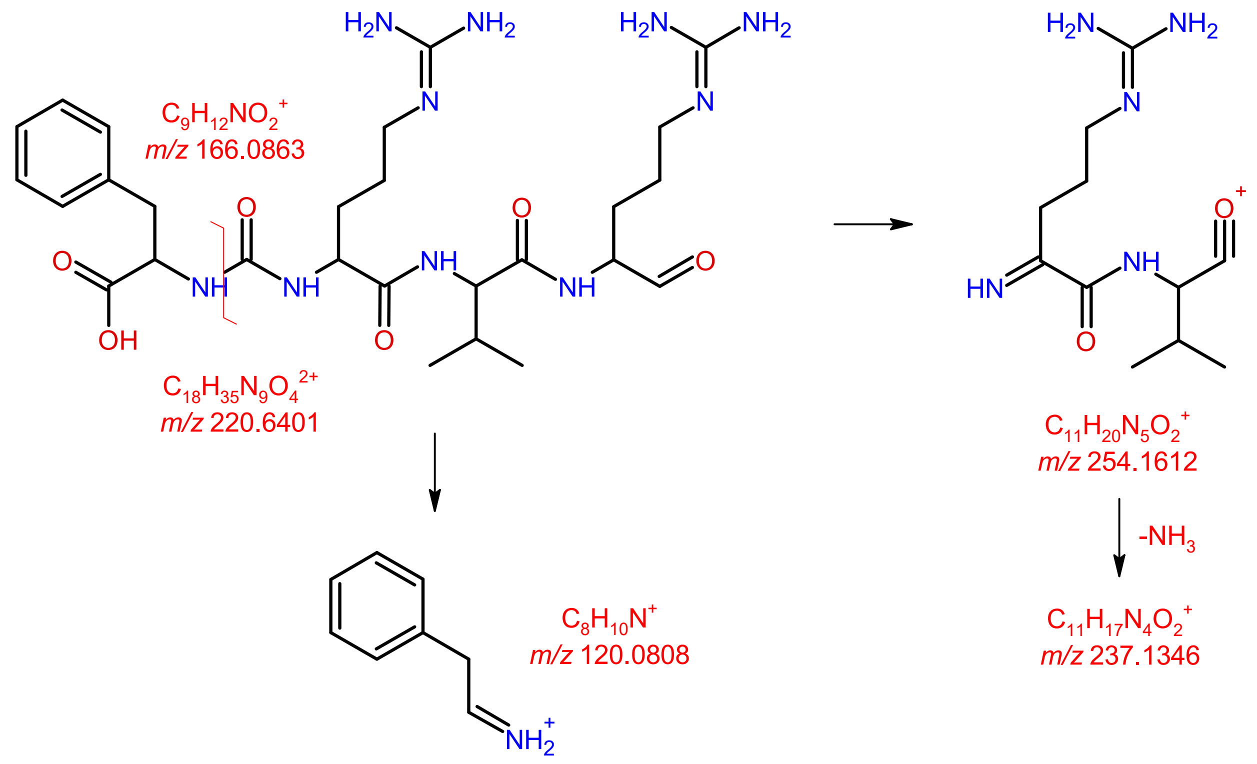

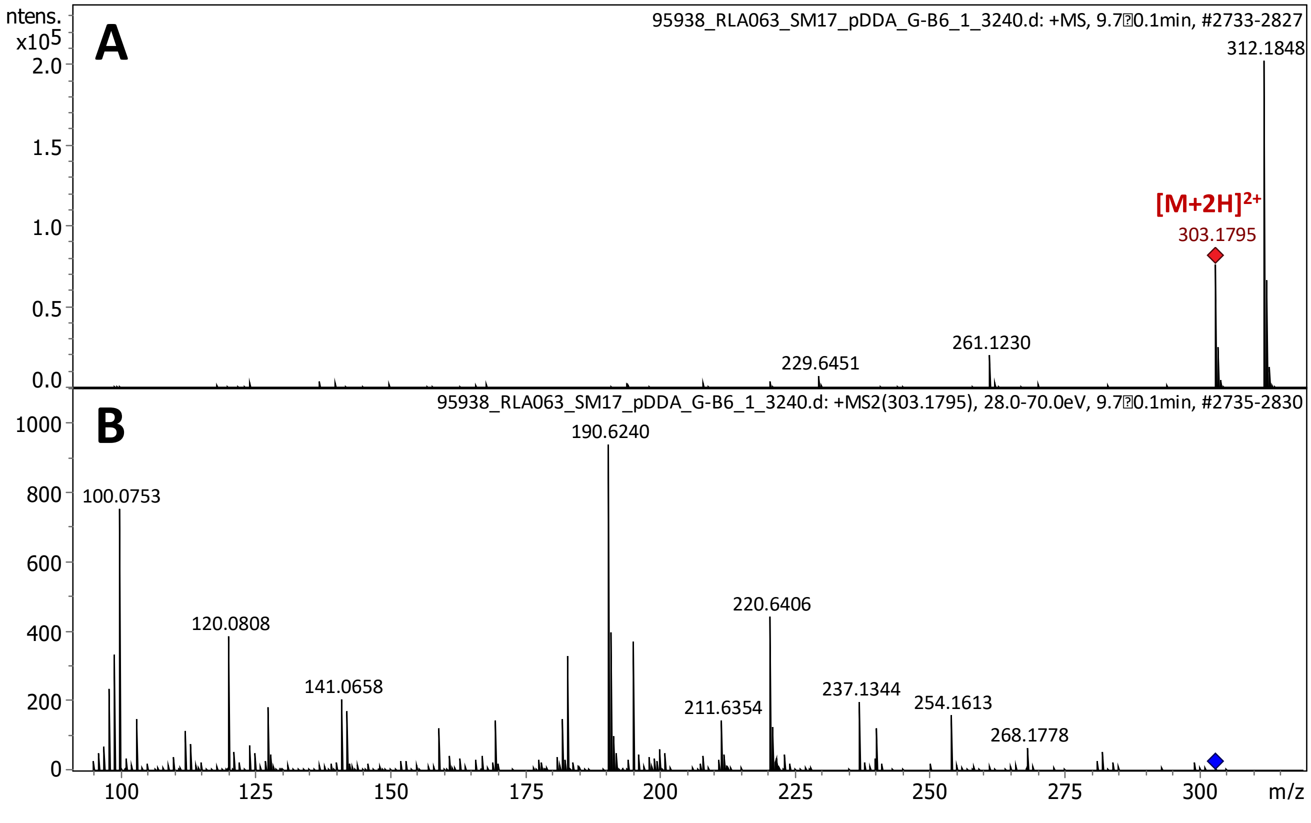

Figure S22. High resolution ESI-Qq-TOF mass spectrum of Antipain (**22**) in strain RLA063 grown in SM17 (A) and high resolution MS/MS spectrum of its $[M+2H]^{2+}$ ion (B).

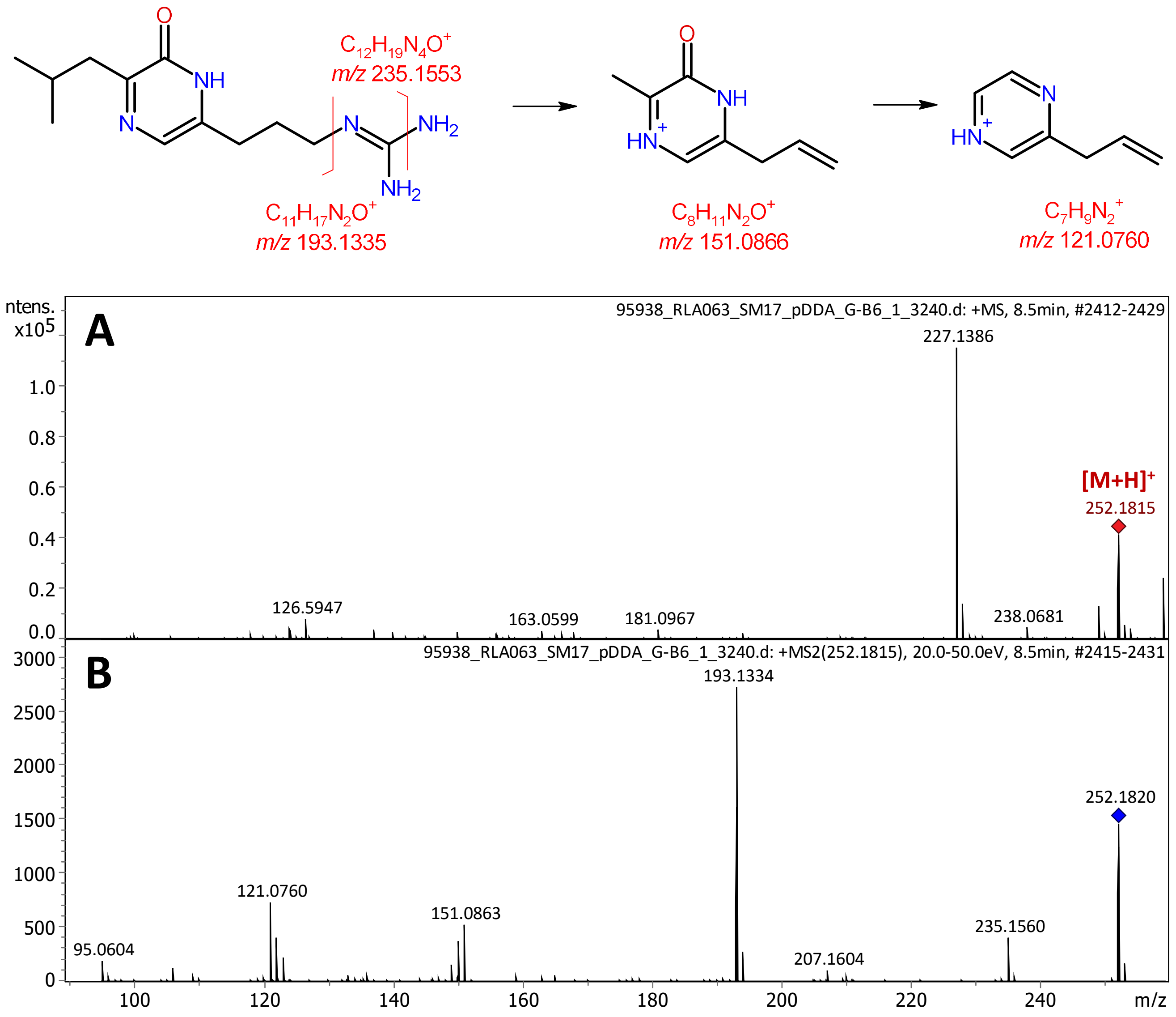

Figure S23. High resolution ESI-Qq-TOF mass spectrum of Arglecin (**23**) in strain RLA063 grown in SM17 (A) and high resolution MS/MS spectrum of its $[M+H]^+$ ion (B).

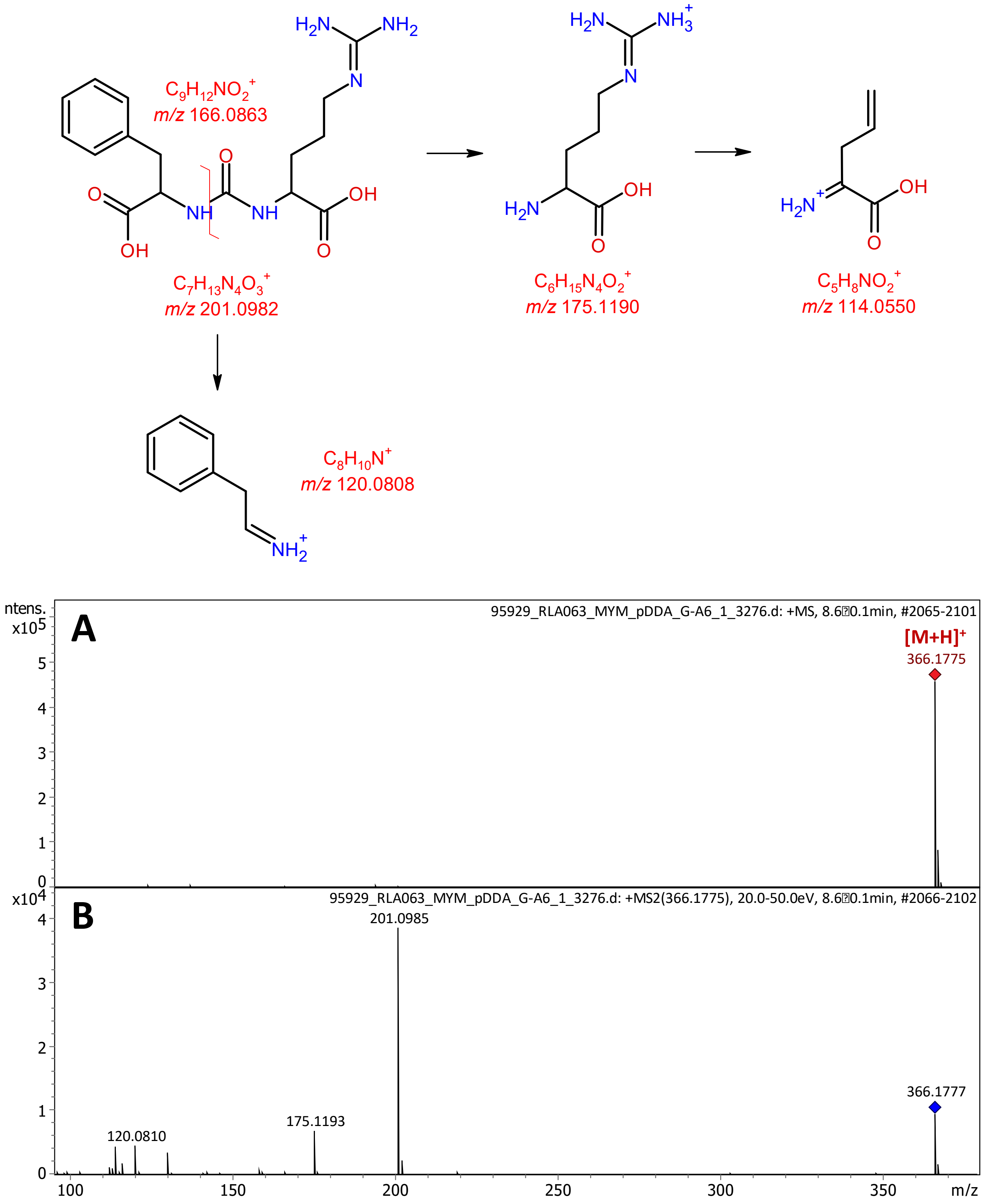

Figure S24. High resolution ESI-Qq-TOF mass spectrum of Metabolite KF77-AG6 (**24**) in strain RLA063 grown in MYM (A) and high resolution MS/MS spectrum of its $[M+H]^+$ ion (B).

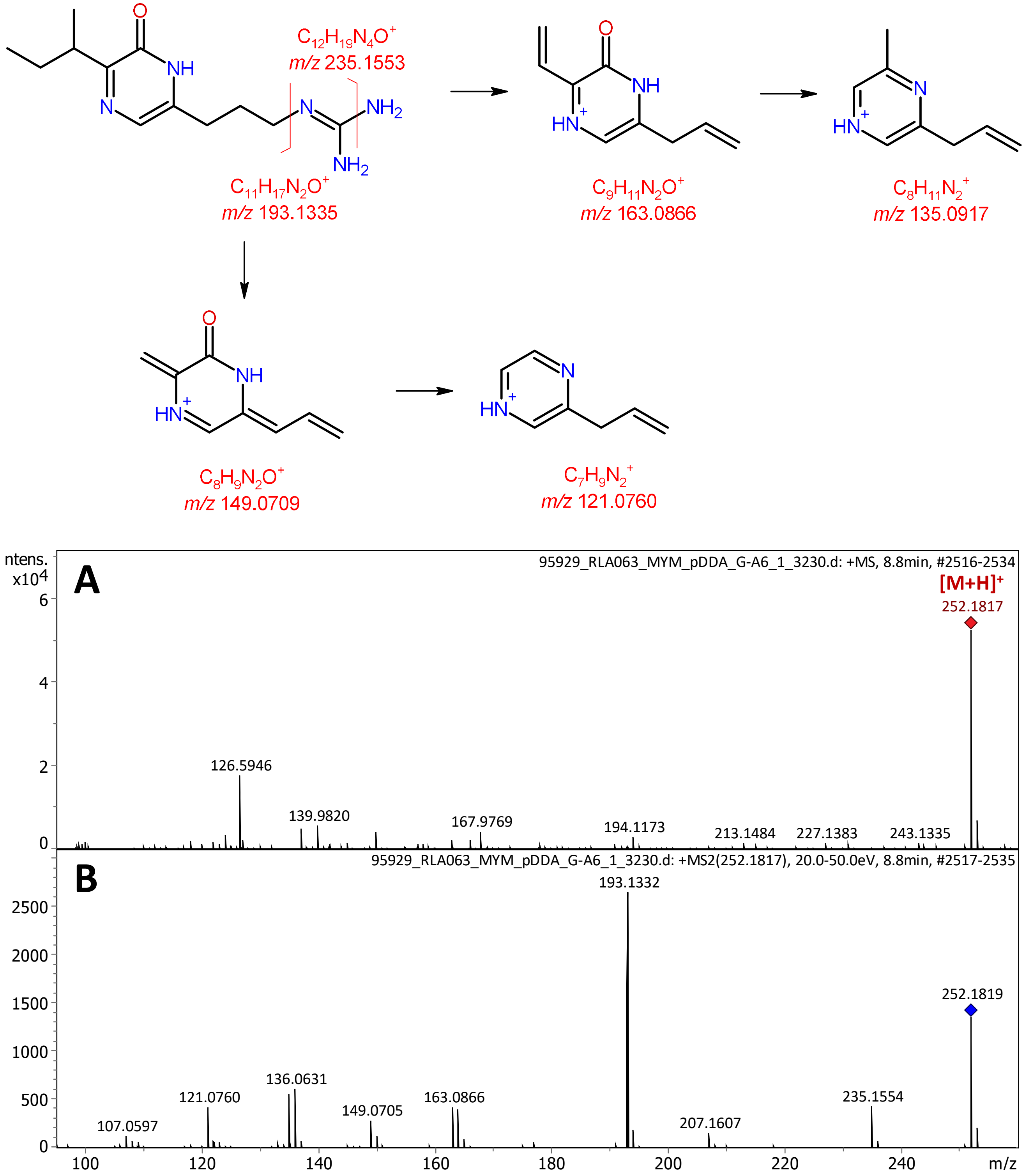

Figure S25. High resolution ESI-Qq-TOF mass spectrum of the Ile-derived Arglecin isomer (**25**) in strain RLA063 grown in MYM (A) and high resolution MS/MS spectrum of its $[M+H]^+$ ion (B).

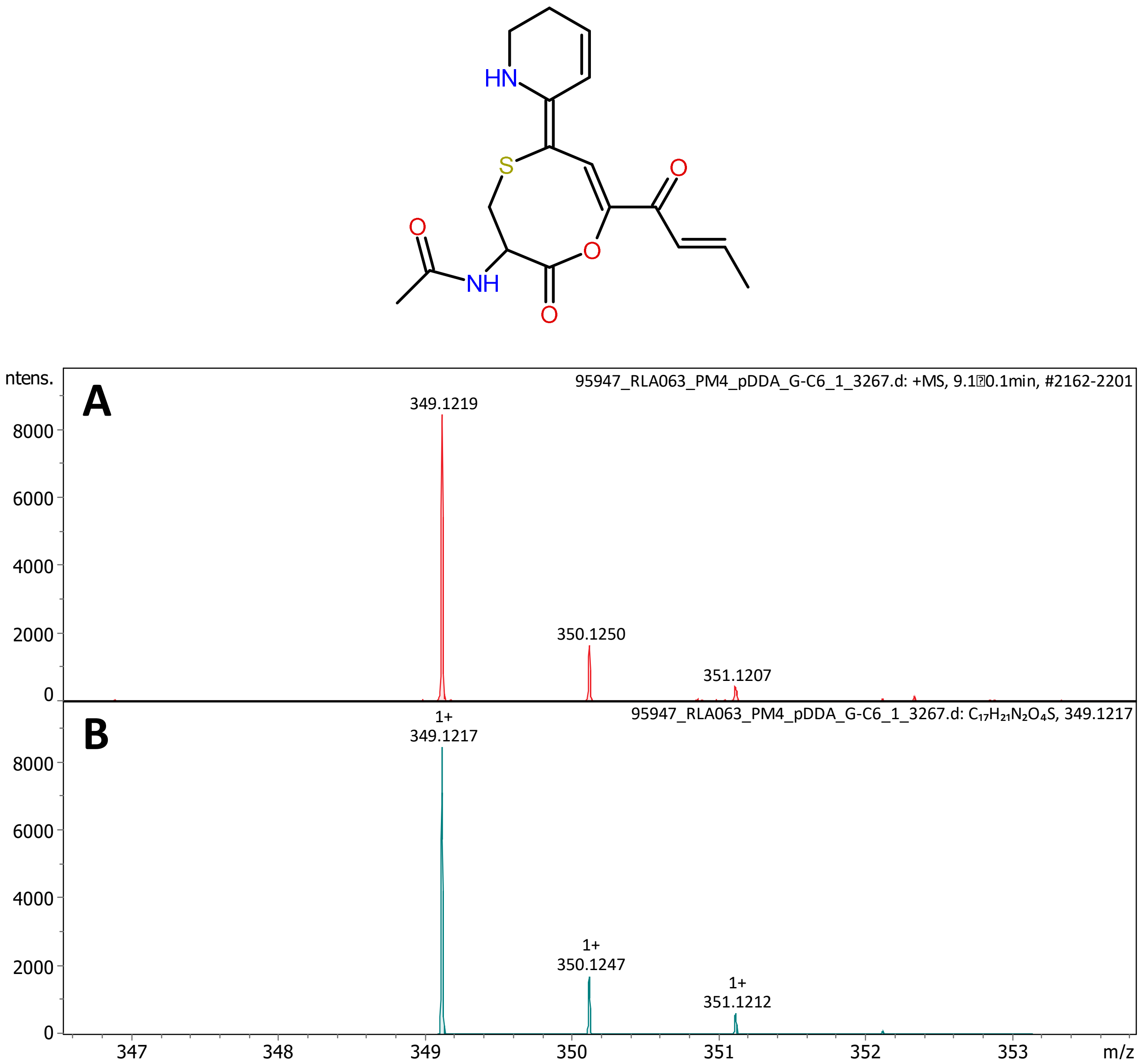

Figure S26. High resolution ESI-Qq-TOF mass spectrum of Coelimycin P1 (**26**) in strain RLA063 grown in PM4 (A) in comparison to the simulated isotopic pattern of the $[M+H]^+$ ion of a compound with the sum formula $C_{17}H_{20}N_2O_4S$ (B).

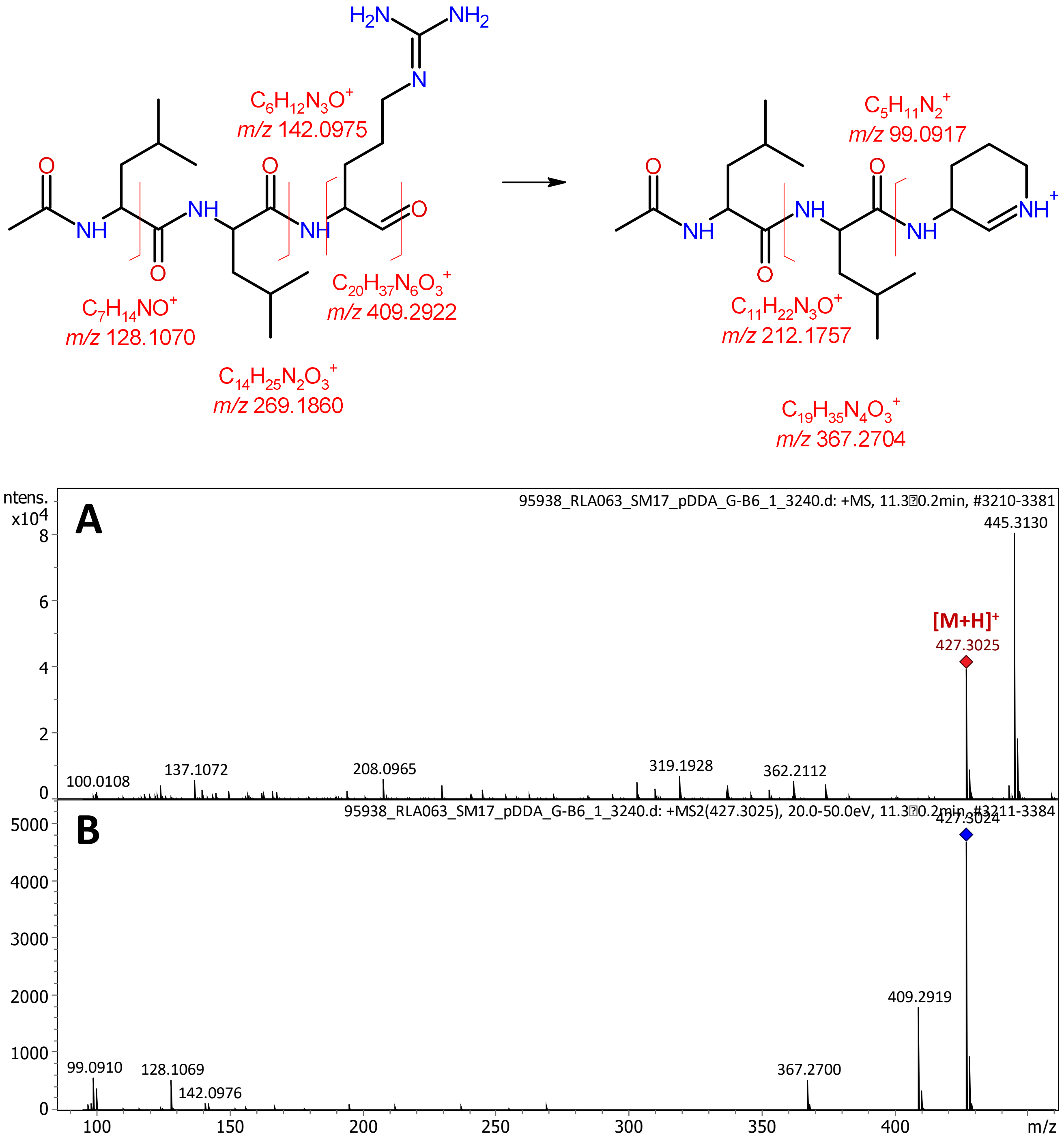

Figure S27. High resolution ESI-Qq-TOF mass spectrum of Leupeptin (**27**) in strain RLA063 grown in SM17 (A) and high resolution MS/MS spectrum of its $[M+H]^+$ ion (B).

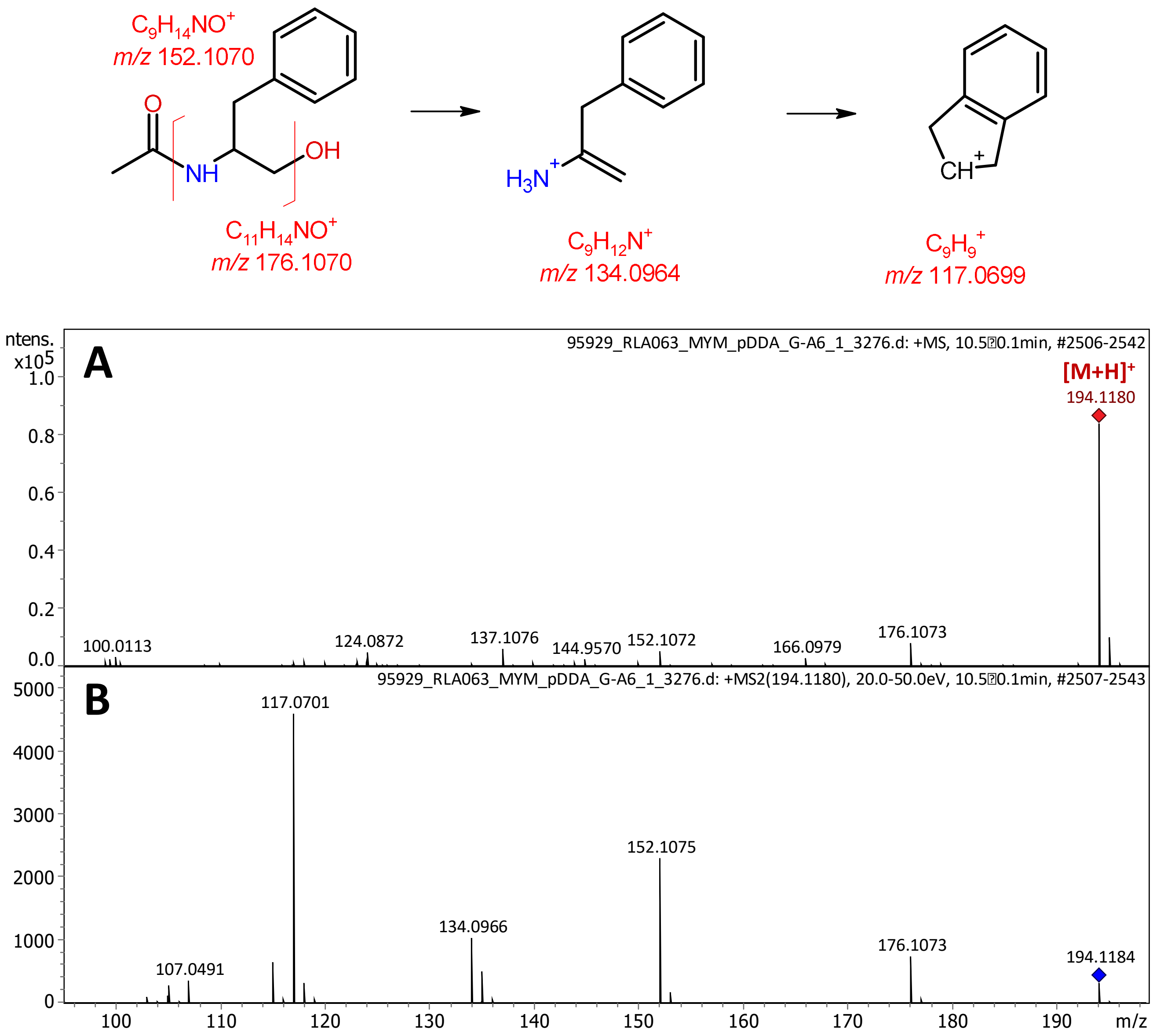

Figure S28. High resolution ESI-Qq-TOF mass spectrum of *N*-Acetylphenylalaninol (**28**) in strain RLA063 grown in MYM (A) and high resolution MS/MS spectrum of its $[M+H]^+$ ion (B).

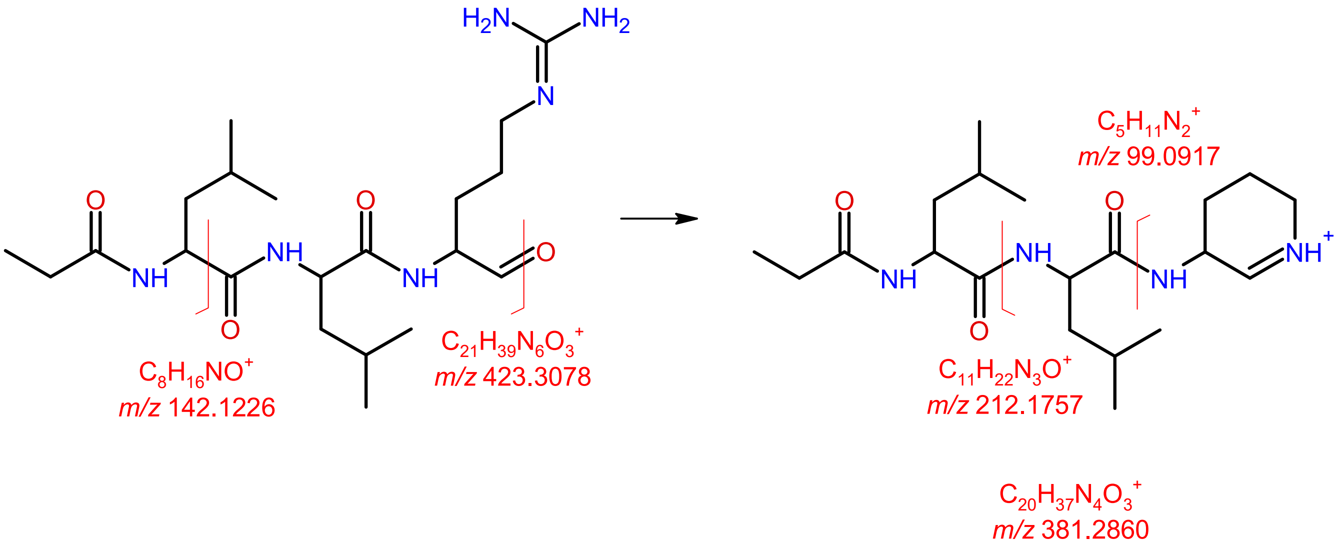

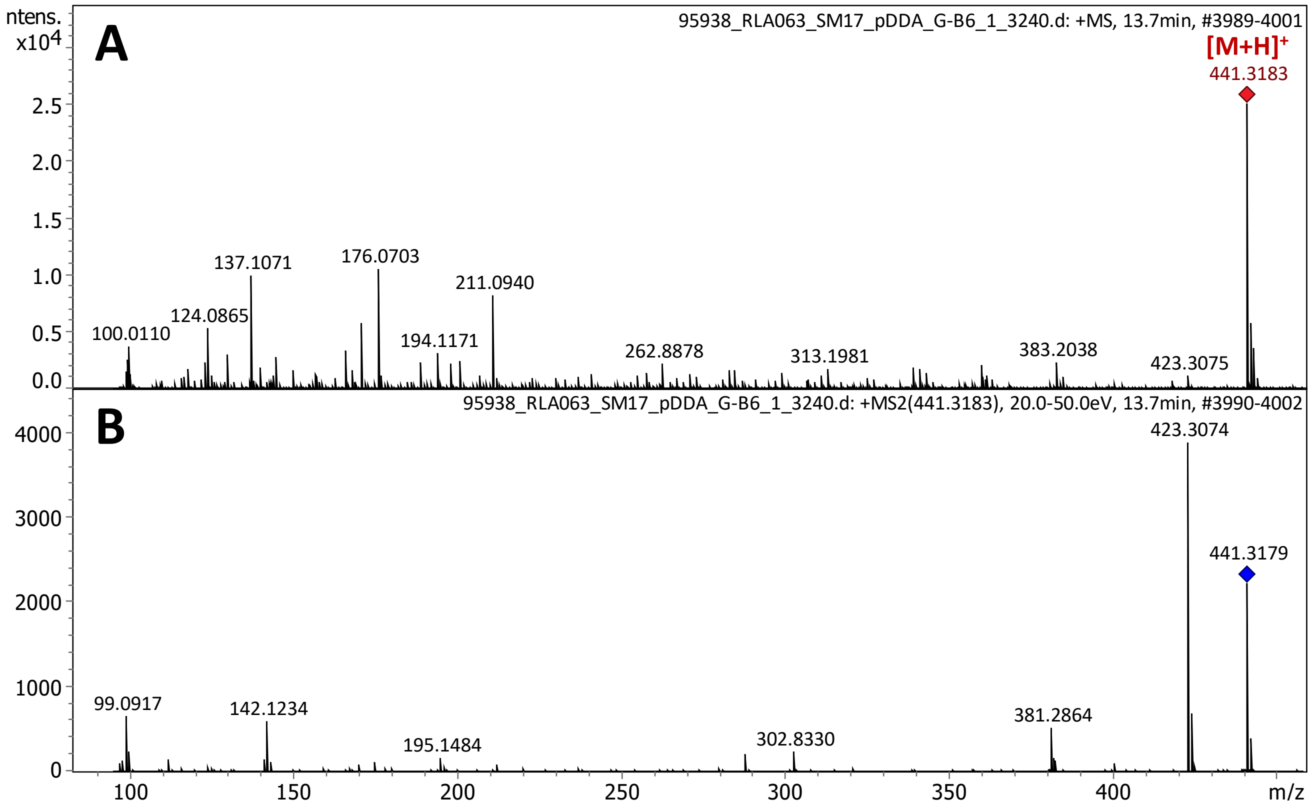

Figure S29. High resolution ESI-Qq-TOF mass spectrum of Leupeptin Pr-LL (**29**) in strain RLA063 grown in SM17 (A) and high resolution MS/MS spectrum of its $[M+H]^+$ ion (B).

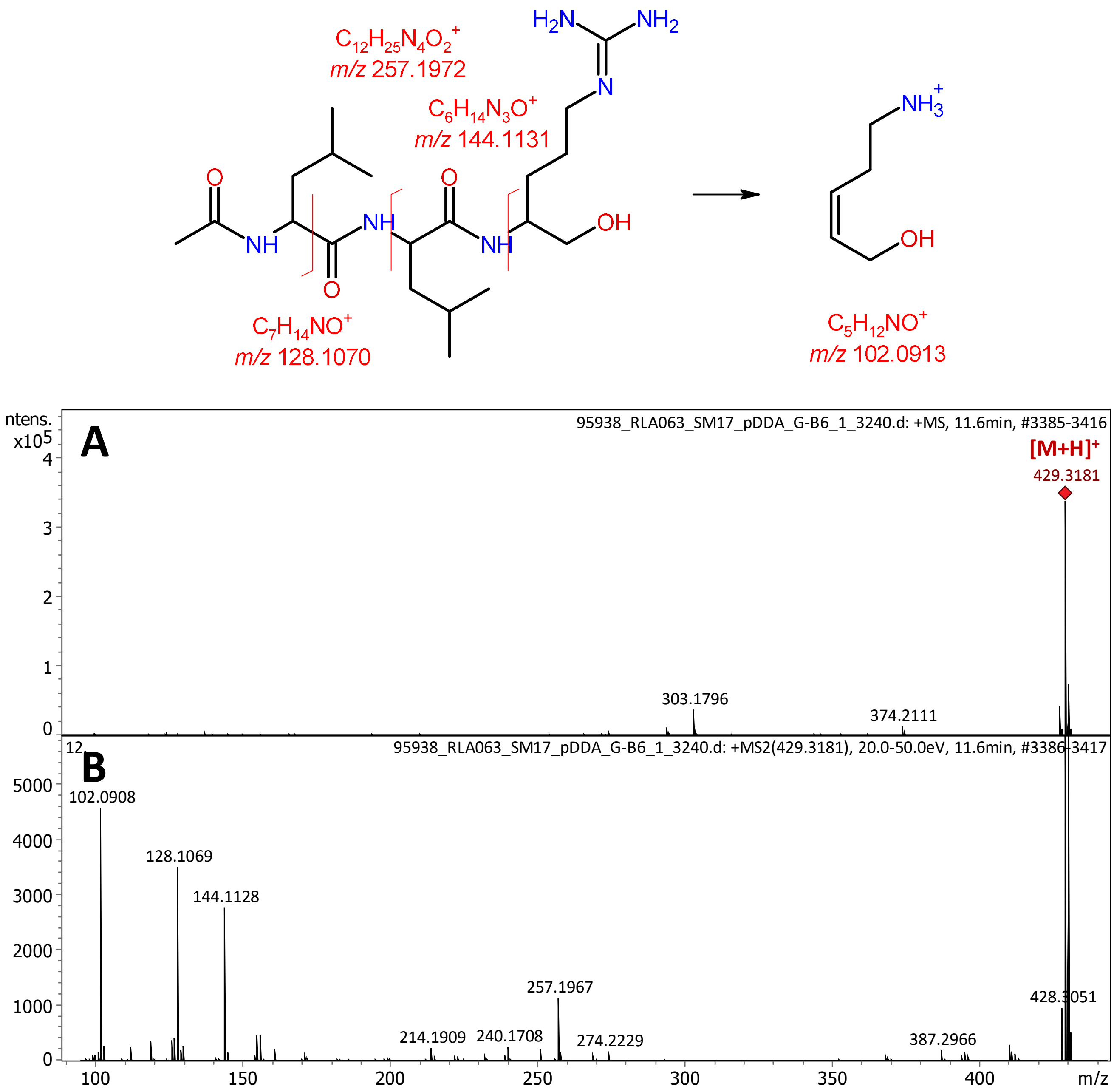

Figure S30. High resolution ESI-Qq-TOF mass spectrum of reduced Leupeptin (**30**) in strain RLA063 grown in SM17 (A) and high resolution MS/MS spectrum of its $[M+H]^+$ ion (B).

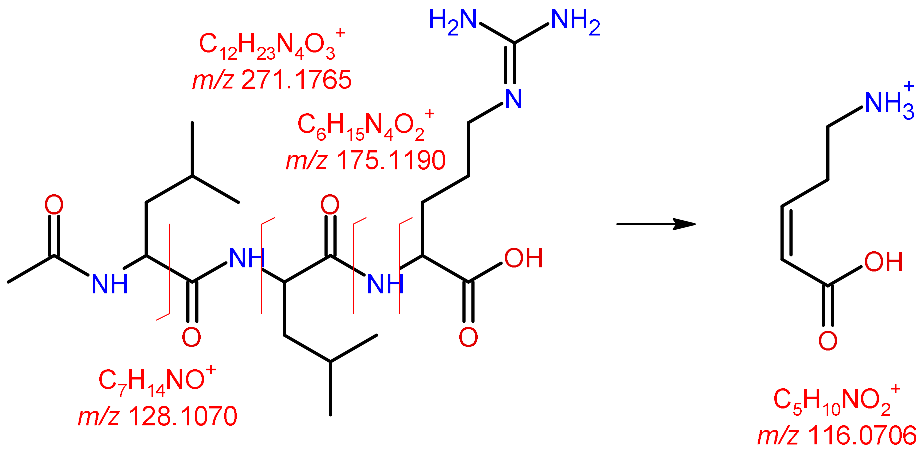

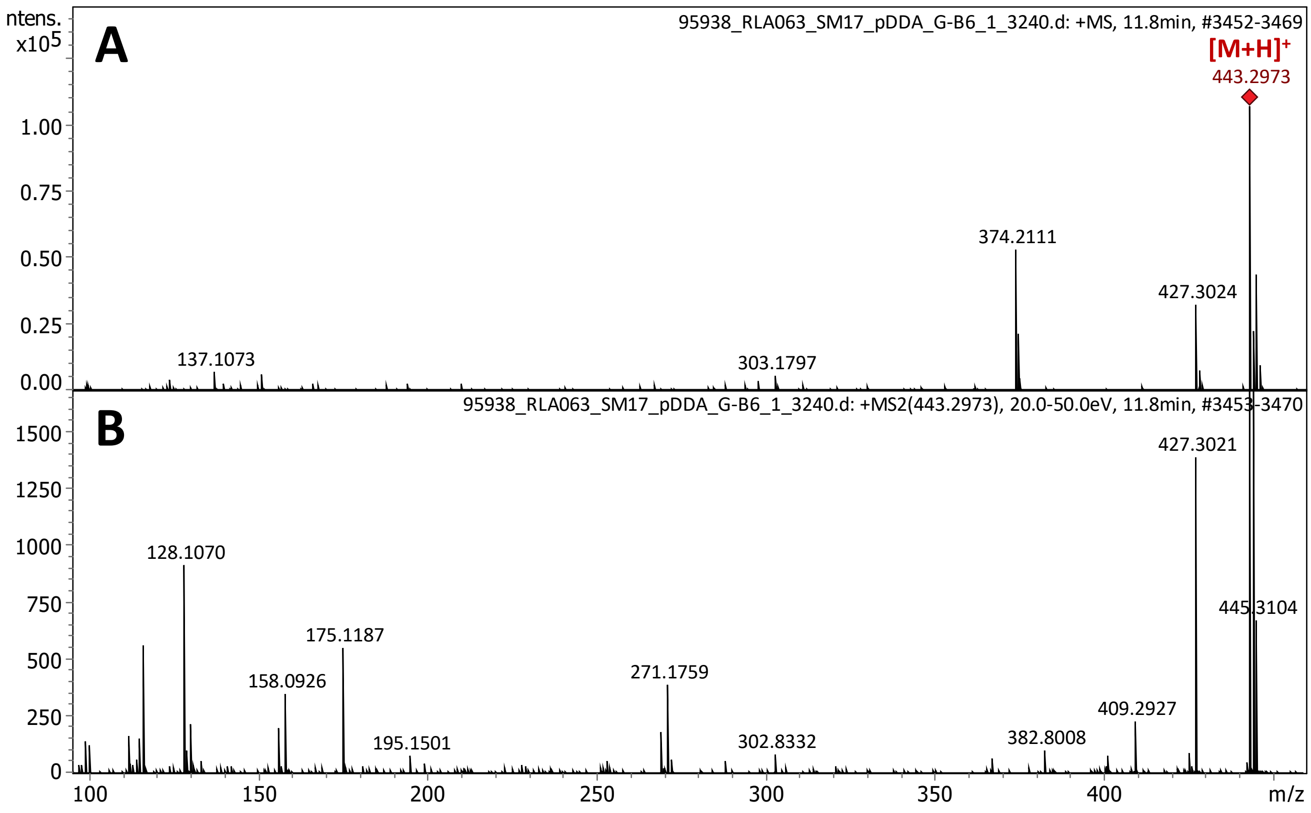

Figure S31. High resolution ESI-Qq-TOF mass spectrum of oxidized Leupeptin (**31**) in strain RLA063 grown in SM17 (A) and high resolution MS/MS spectrum of its $[M+H]^+$ ion (B).

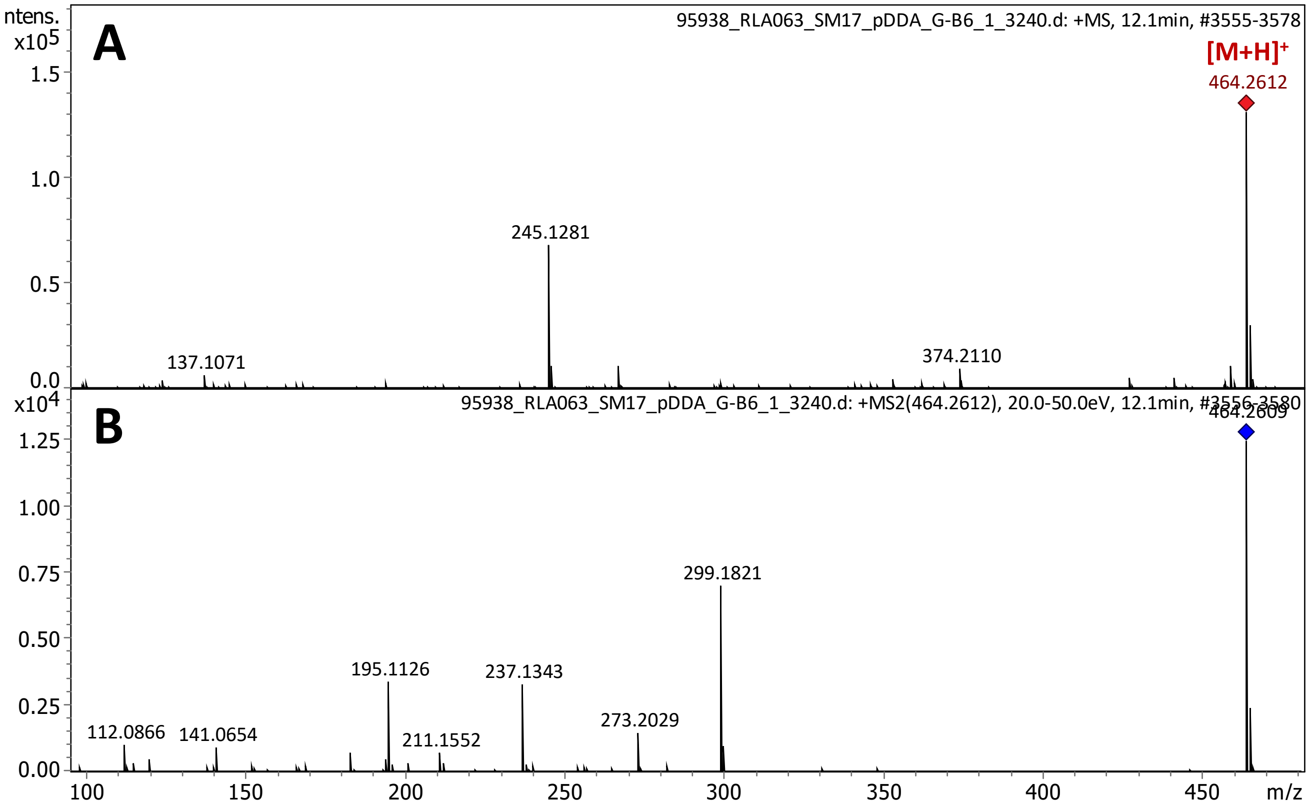

Figure S32. High resolution ESI-Qq-TOF mass spectrum of the unknown Leupeptin congener **32** in strain RLA063 grown in SM17 (A) and high resolution MS/MS spectrum of its $[M+H]^+$ ion (B).

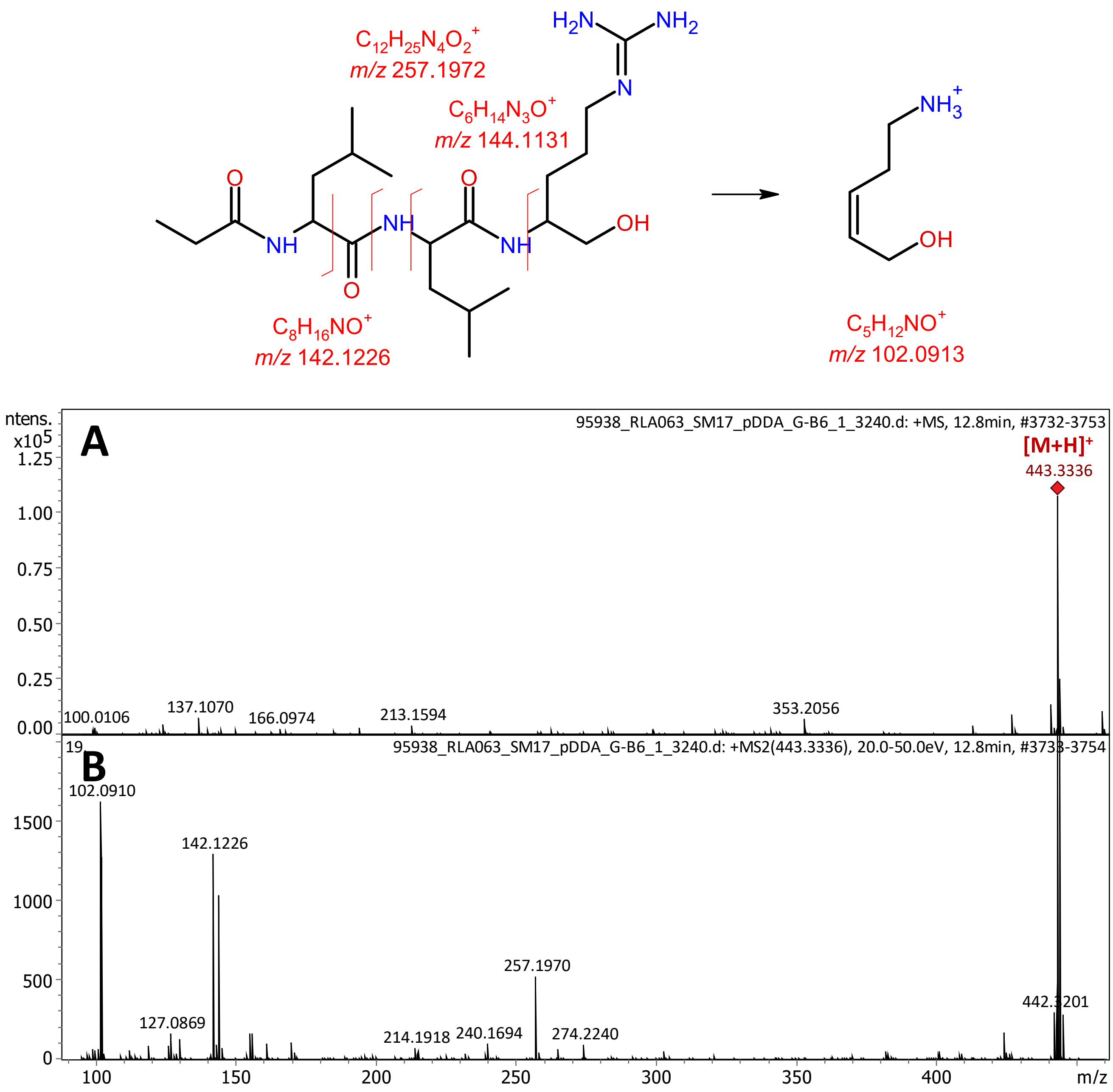

Figure S33. High resolution ESI-Qq-TOF mass spectrum of reduced Leupeptin Pr-LL (**33**) in strain RLA063 grown in SM17 (A) and high resolution MS/MS spectrum of its $[M+H]^+$ ion (B).

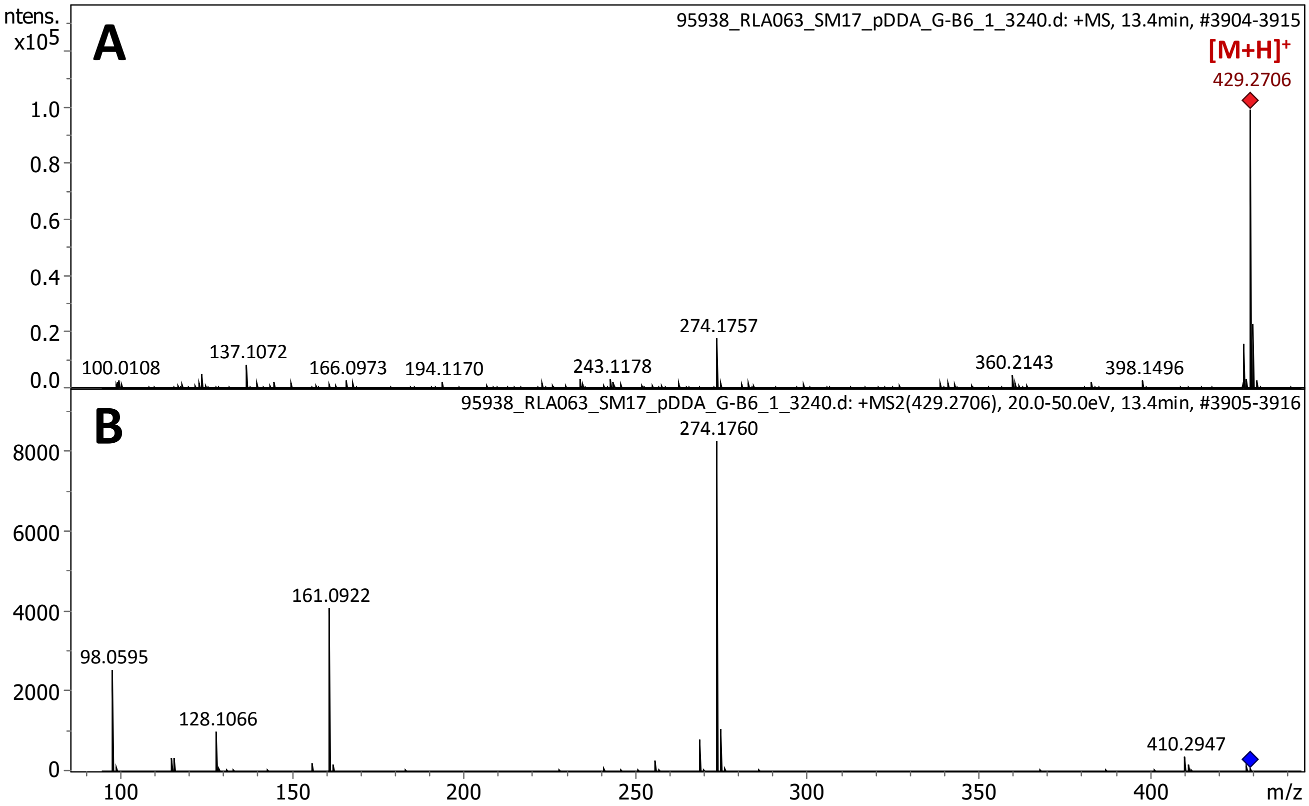

Figure S34. High resolution ESI-Qq-TOF mass spectrum of the unknown Leupeptin congener **34** in strain RLA063 grown in SM17 (A) and high resolution MS/MS spectrum of its $[M+H]^+$ ion (B).

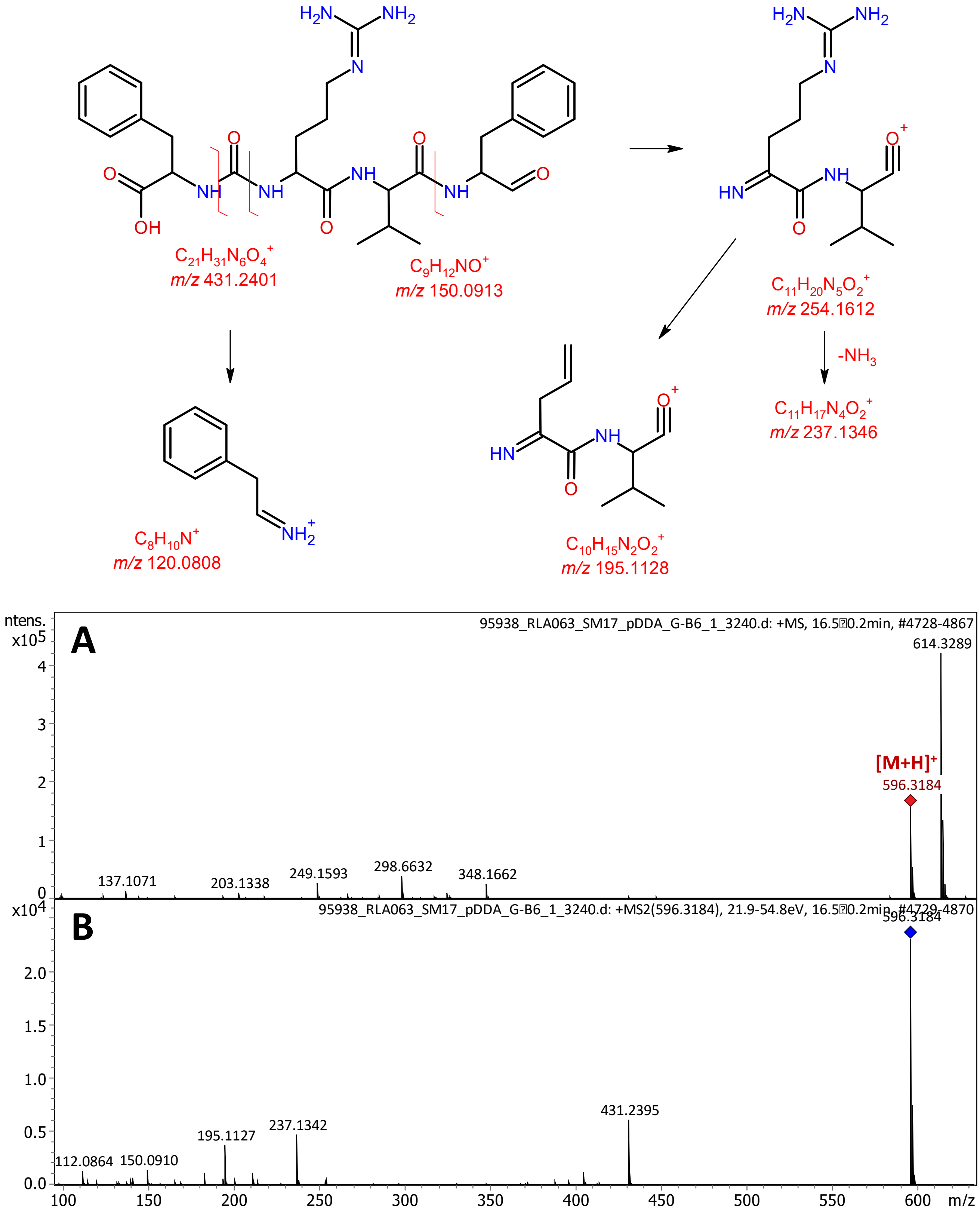

Figure S35. High resolution ESI-Qq-TOF mass spectrum of MAPI (**35**) in strain RLA063 grown in SM17 (A) and high resolution MS/MS spectrum of its $[M+H]^+$ ion (B).

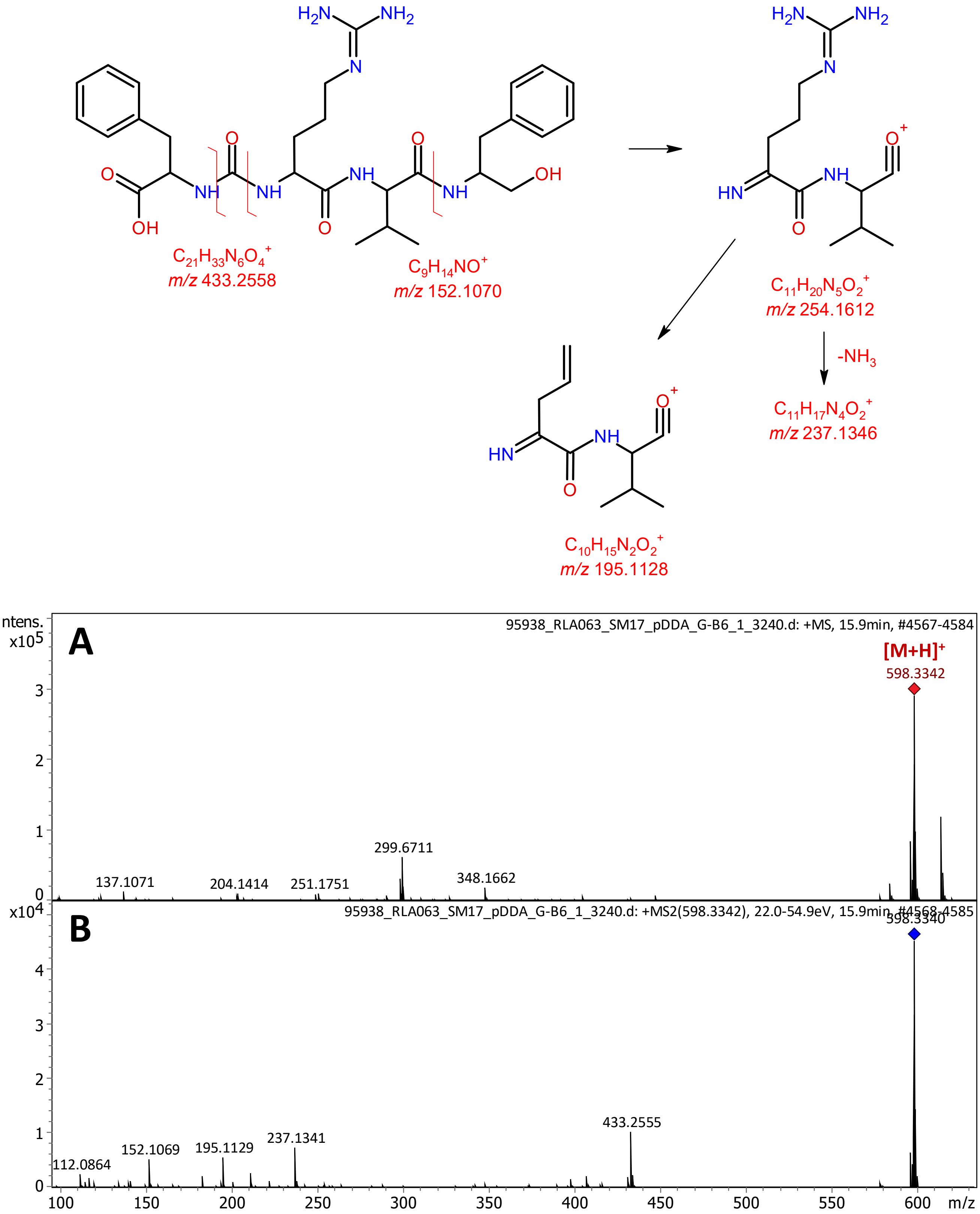

Figure S36. High resolution ESI-Qq-TOF mass spectrum of Mer-N5075-A (**36**) in strain RLA063 grown in SM17 (A) and high resolution MS/MS spectrum of its $[M+H]^+$ ion (B).

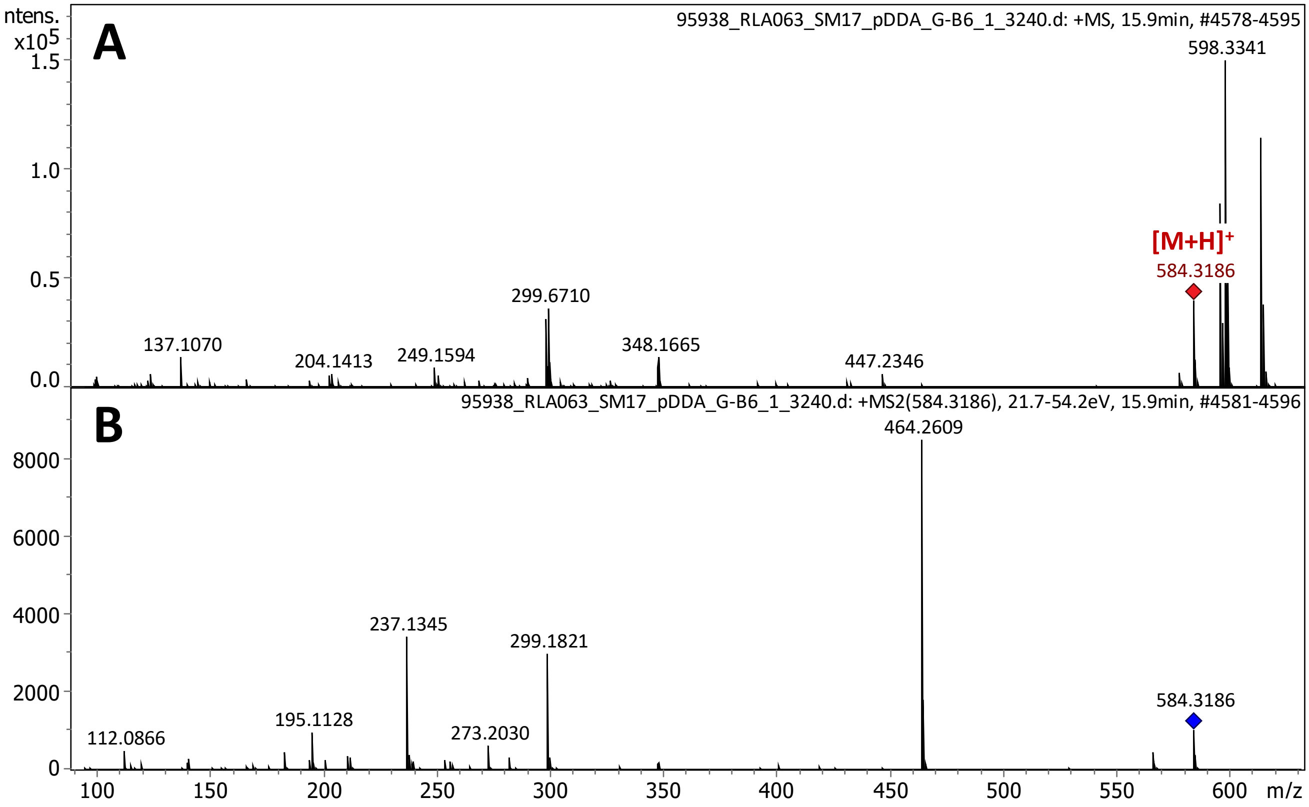

Figure S37. High resolution ESI-Qq-TOF mass spectrum of the unknown MAPI congener **37** in strain RLA063 grown in SM17 (A) and high resolution MS/MS spectrum of its $[M+H]^+$ ion (B).

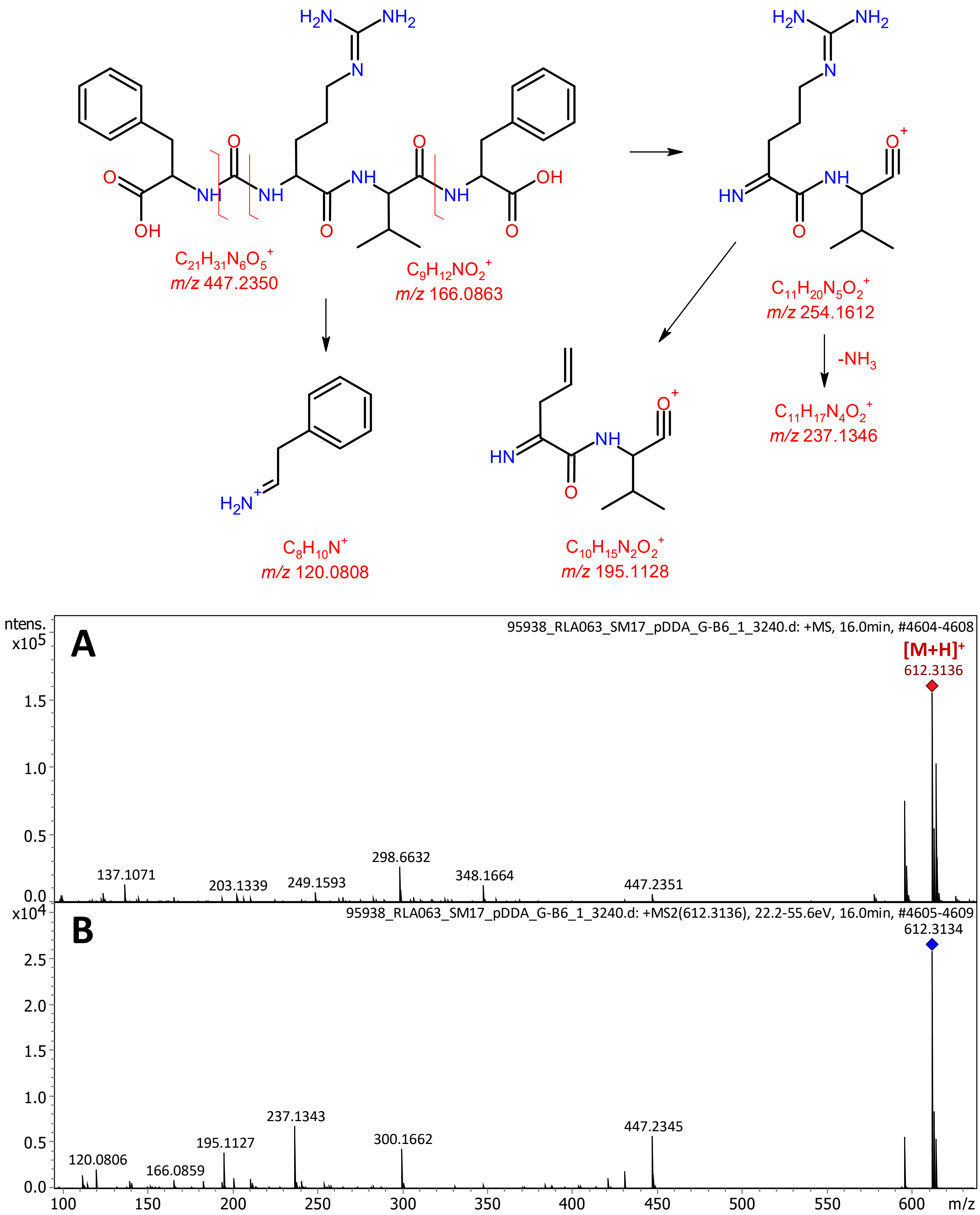

Figure S38. High resolution ESI-Qq-TOF mass spectrum of oxidized MAPI (**38**) in strain RLA063 grown in SM17 (A) and high resolution MS/MS spectrum of its $[M+H]^+$ ion (B).

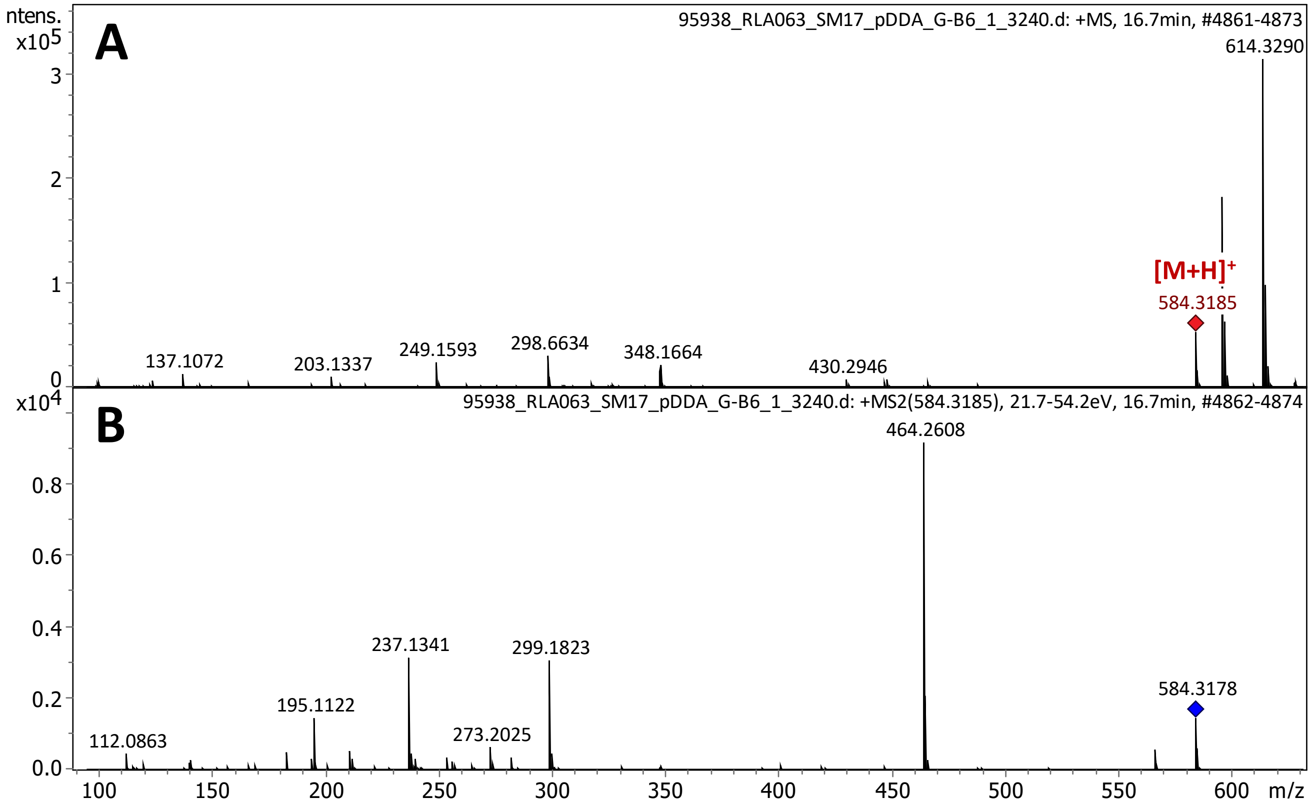

Figure S39. High resolution ESI-Qq-TOF mass spectrum of the unknown MAPI congener **39** in strain RLA063 grown in SM17 (A) and high resolution MS/MS spectrum of its $[M+H]^+$ ion (B).

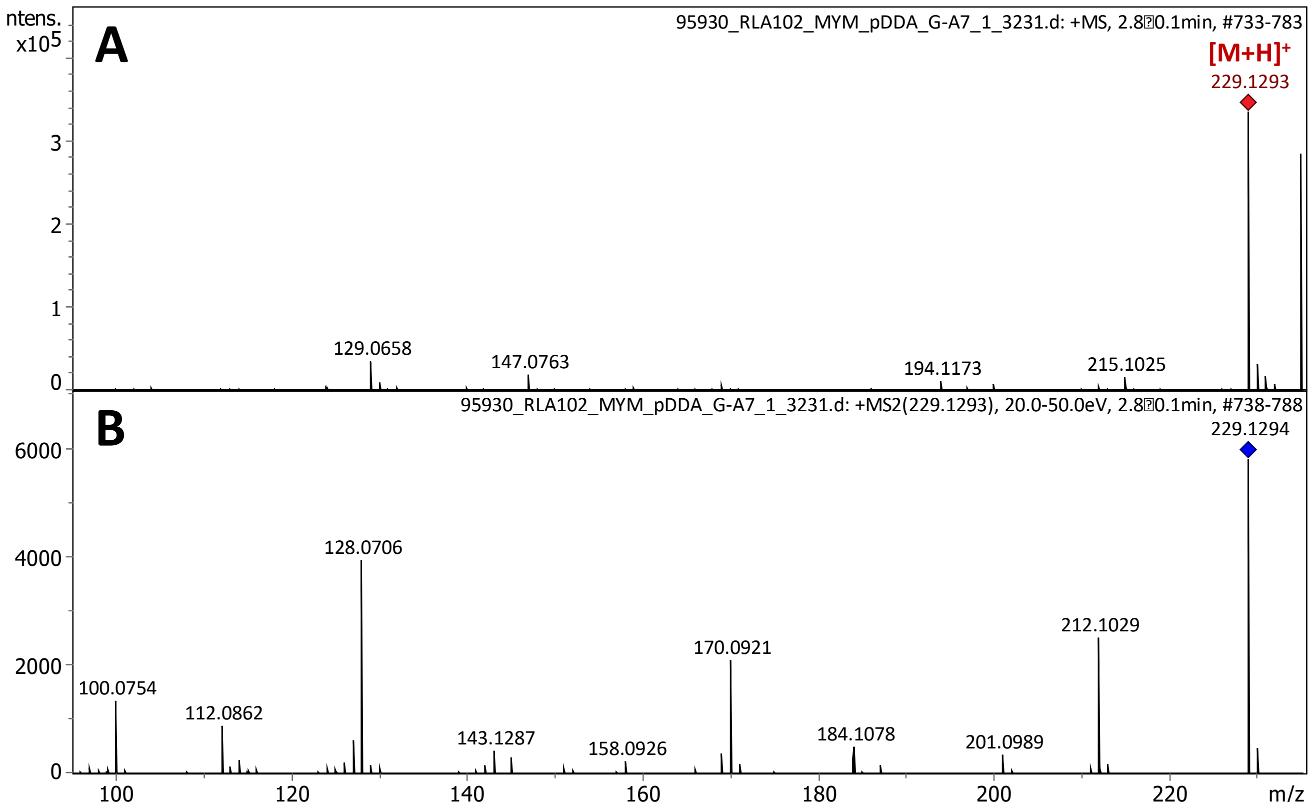

Figure S40. High resolution ESI-Qq-TOF mass spectrum of the potentially new natural product **40** in strain RLA102 grown in MYM (A) and high resolution MS/MS spectrum of its $[M+H]^+$ ion (B).

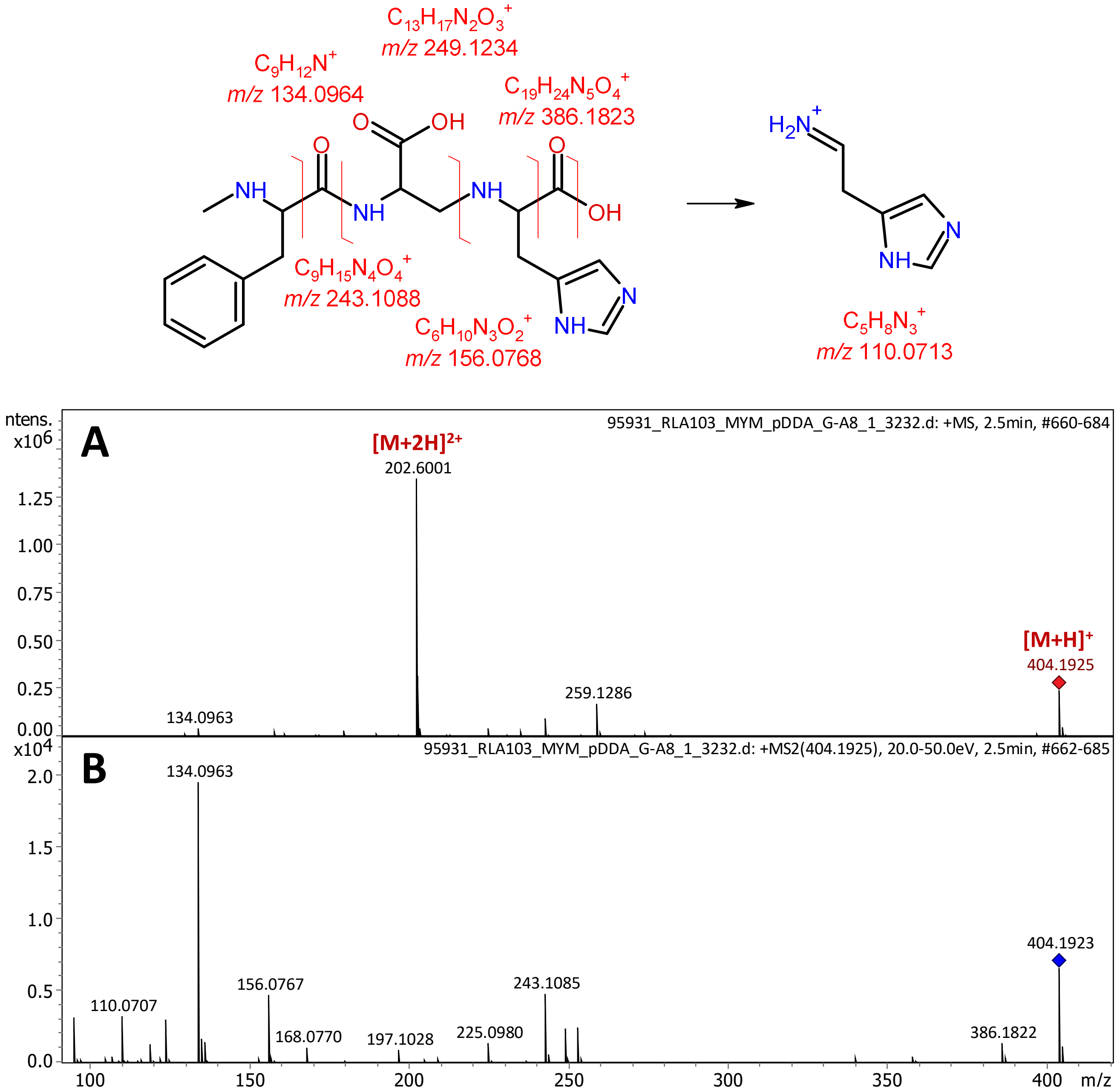

Figure S41. High resolution ESI-Qq-TOF mass spectrum of Melanostatin/BMY 28566 (**41**) in strain RLA103 grown in MYM (A) and high resolution MS/MS spectrum of its $[M+H]^+$ ion (B).

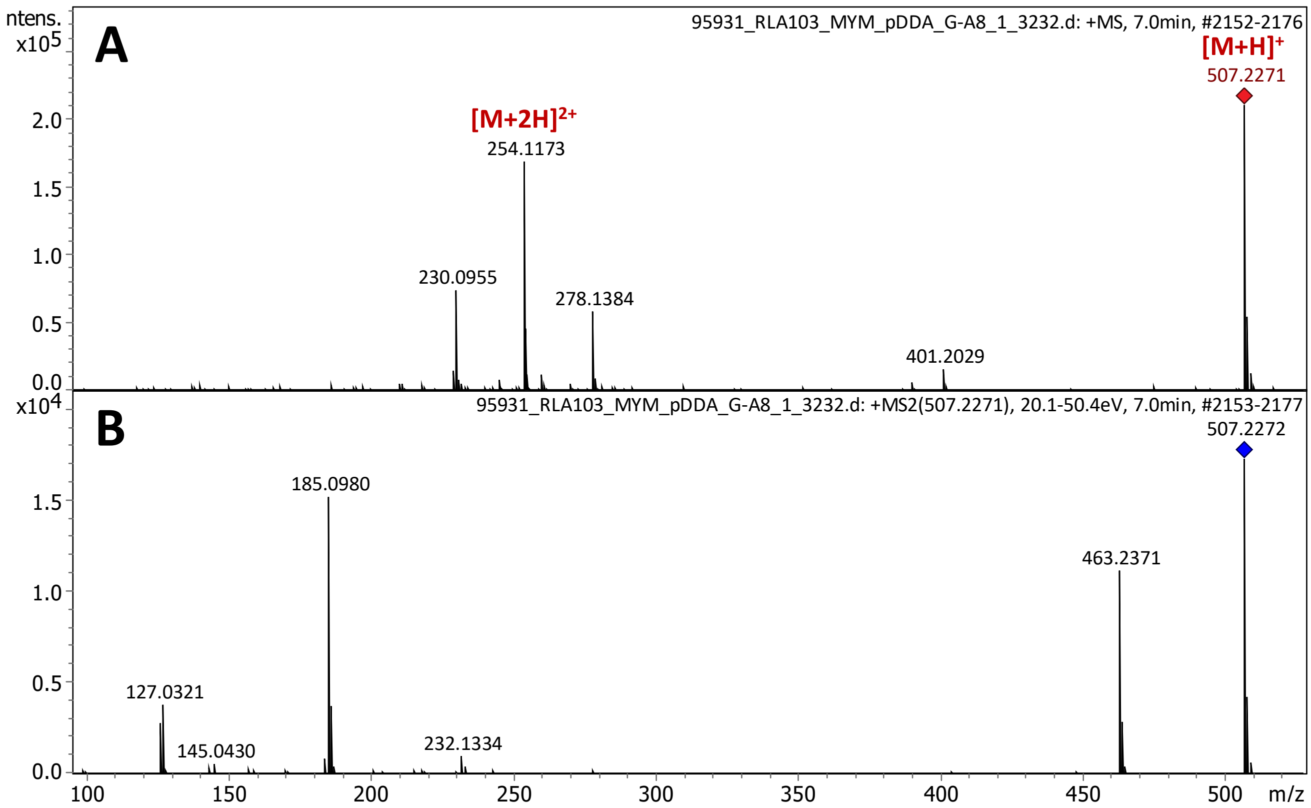

Figure S42. High resolution ESI-Qq-TOF mass spectrum of the potentially new natural product **42** in strain RLA103 grown in MYM (A) and high resolution MS/MS spectrum of its $[M+H]^+$ ion (B).

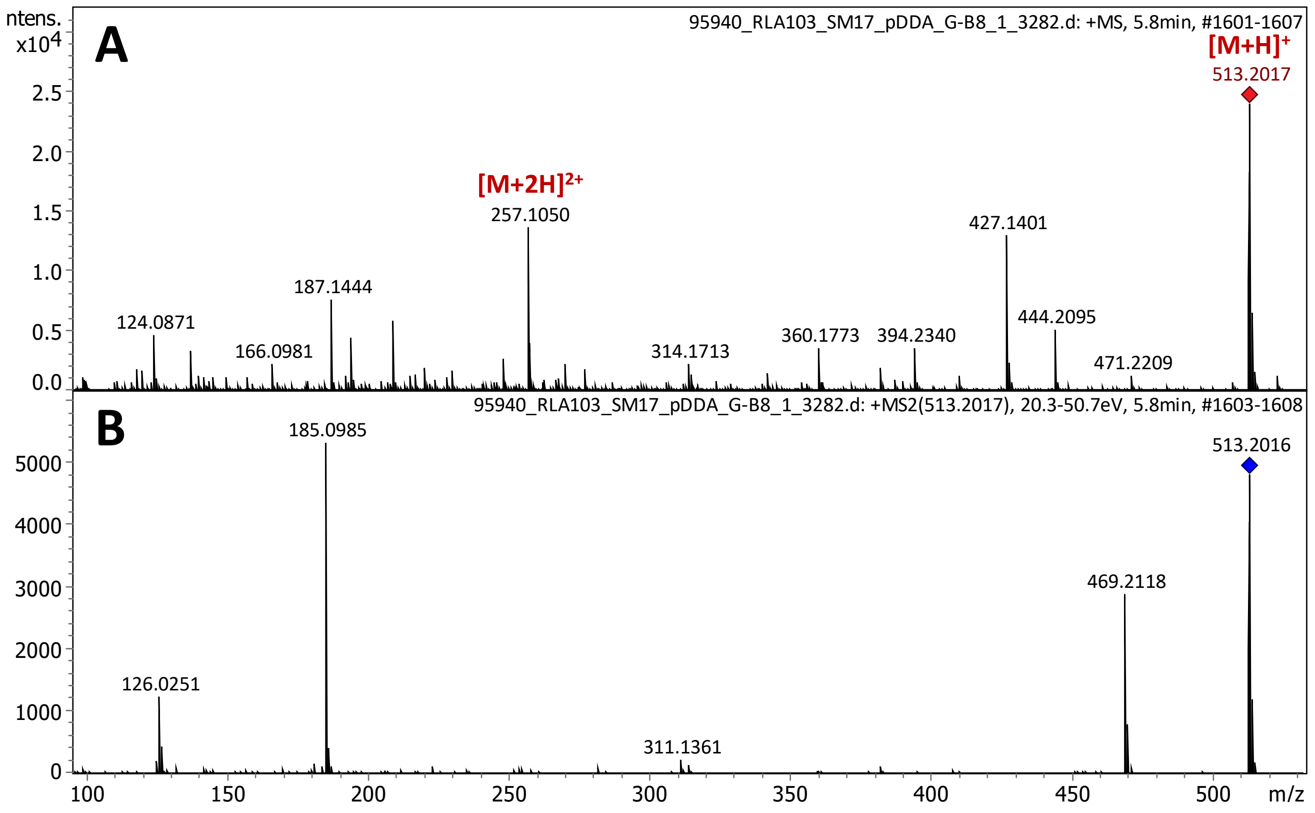

Figure S43. High resolution ESI-Qq-TOF mass spectrum of the potentially new natural product **43** in strain RLA103 grown in SM17 (A) and high resolution MS/MS spectrum of its $[M+H]^+$ ion (B).

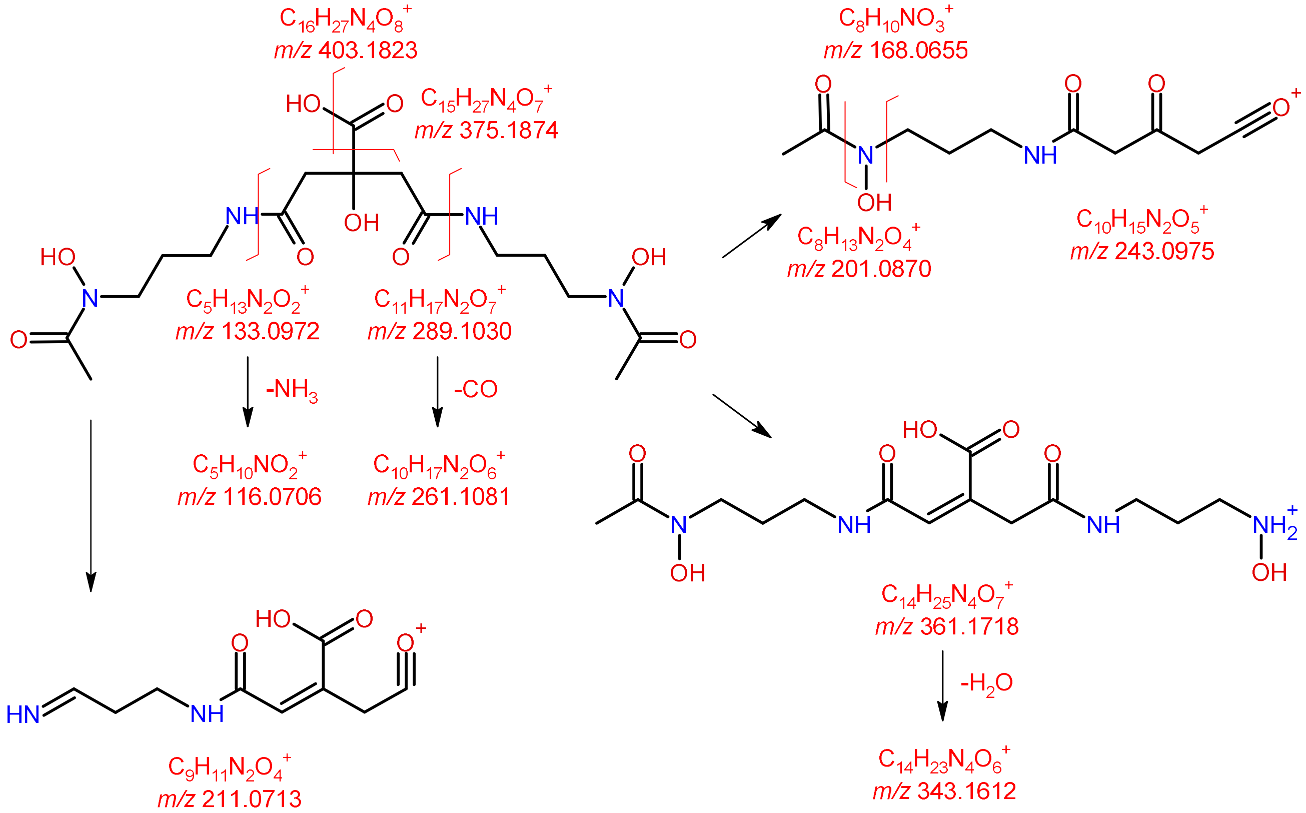

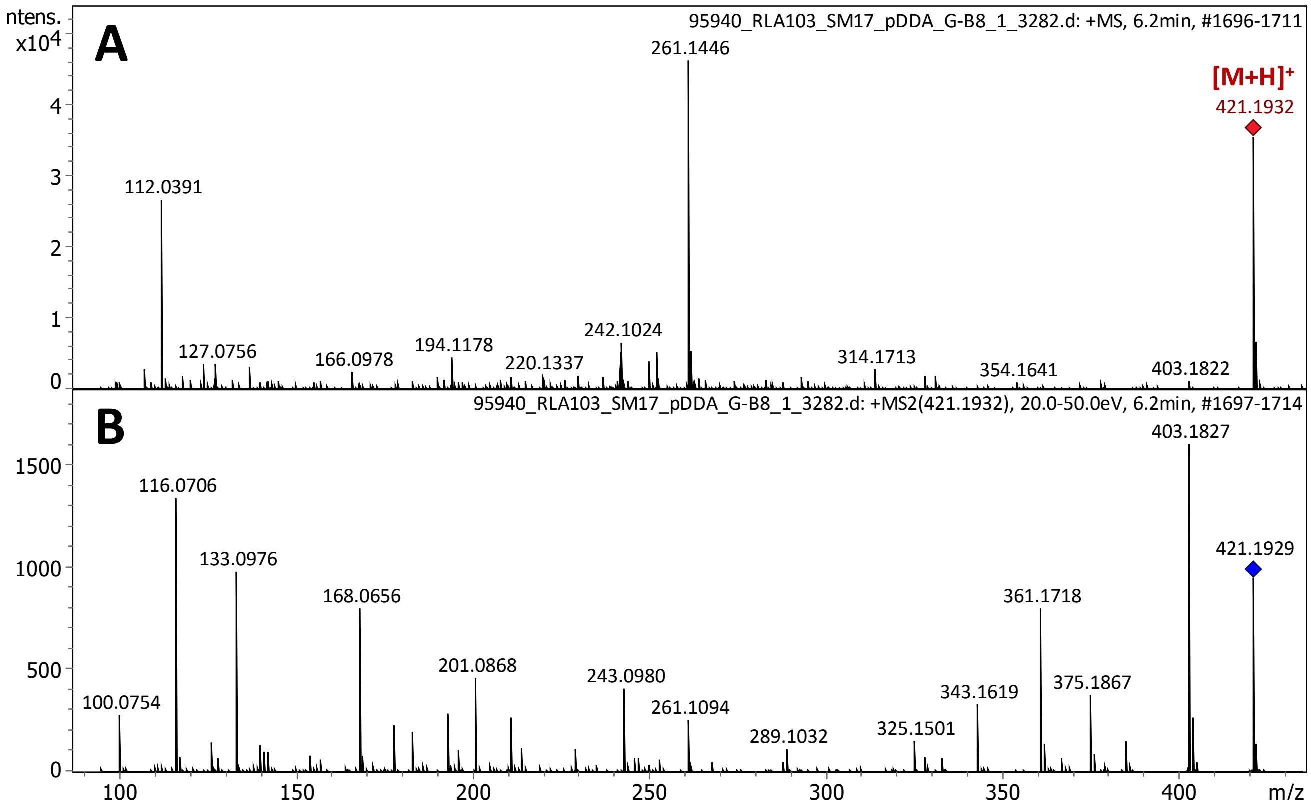

Figure S44. High resolution ESI-Qq-TOF mass spectrum of Schizokinen (**44**) in strain RLA103 grown in SM17 (A) and high resolution MS/MS spectrum of its $[M+H]^+$ ion (B).

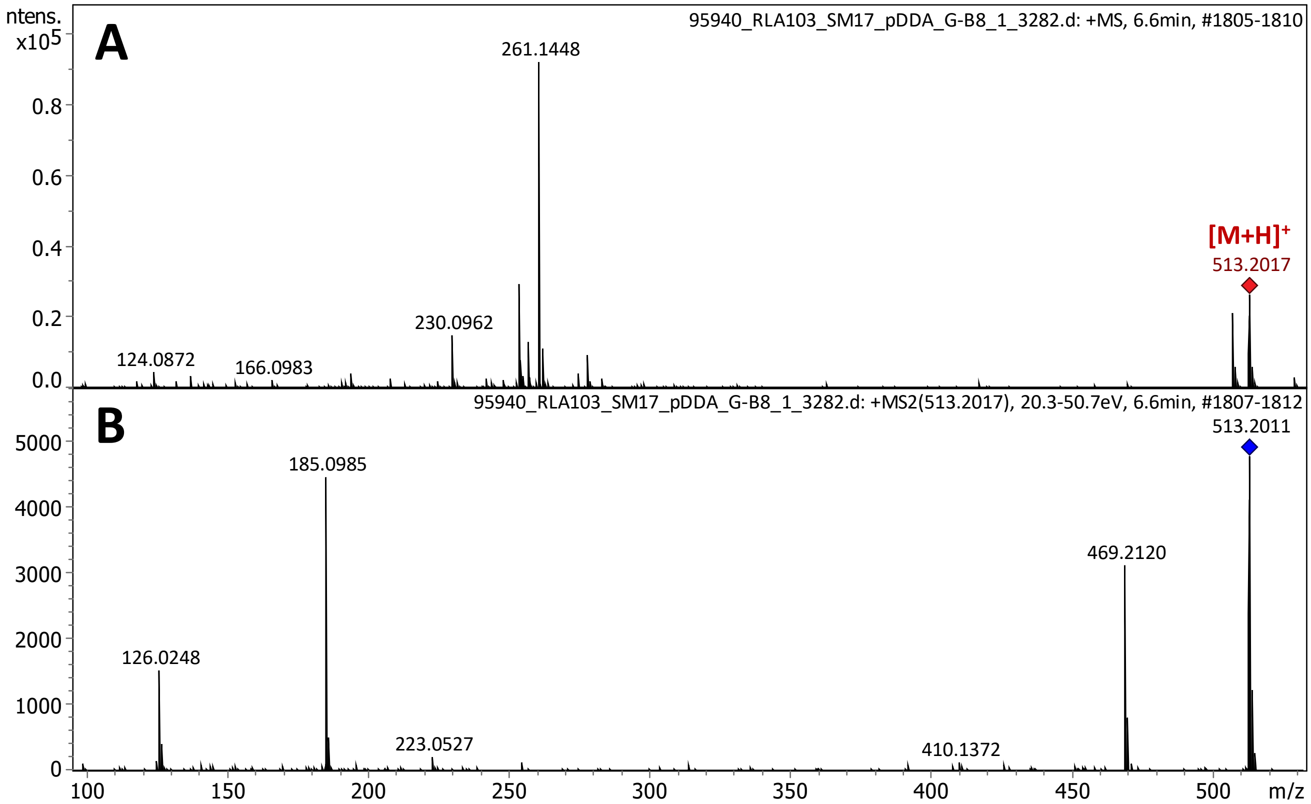

Figure S45. High resolution ESI-Qq-TOF mass spectrum of the potentially new natural product **45** in strain RLA103 grown in SM17 (A) and high resolution MS/MS spectrum of its $[M+H]^+$ ion (B).

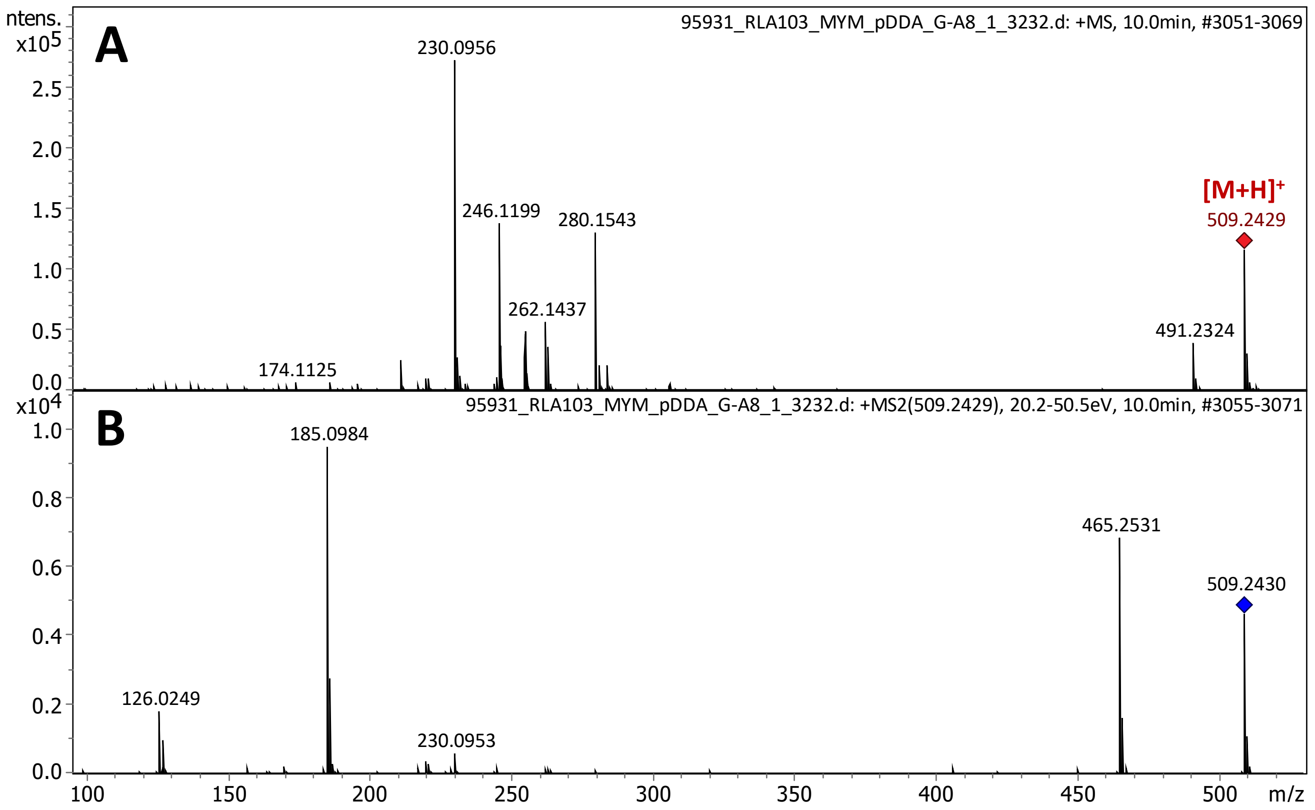

Figure S46. High resolution ESI-Qq-TOF mass spectrum of the potentially new natural product **46** in strain RLA103 grown in MYM (A) and high resolution MS/MS spectrum of its $[M+H]^+$ ion (B).

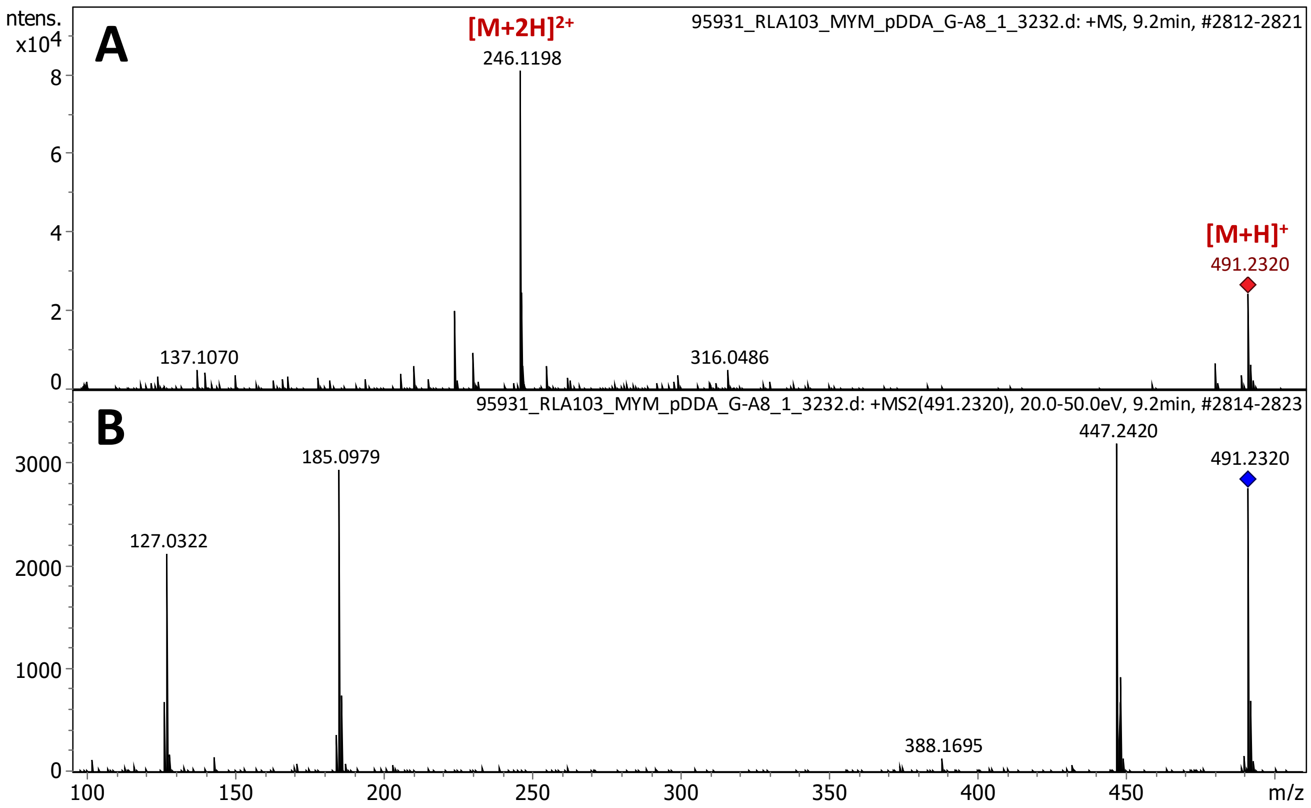

Figure S47. High resolution ESI-Qq-TOF mass spectrum of the potentially new natural product **47** in strain RLA103 grown in MYM (A) and high resolution MS/MS spectrum of its $[M+H]^+$ ion (B).

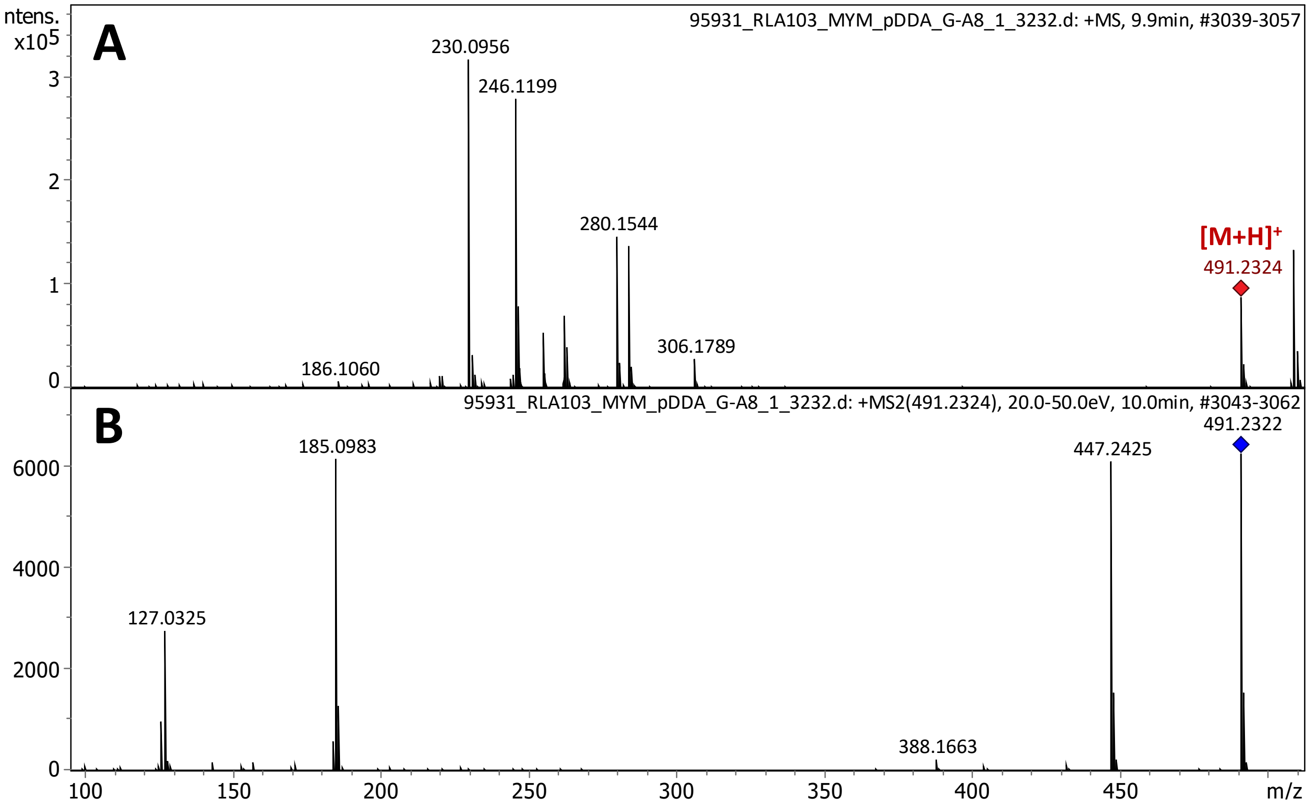

Figure S48. High resolution ESI-Qq-TOF mass spectrum of the potentially new natural product **48** in strain RLA103 grown in MYM (A) and high resolution MS/MS spectrum of its $[M+H]^+$ ion (B).

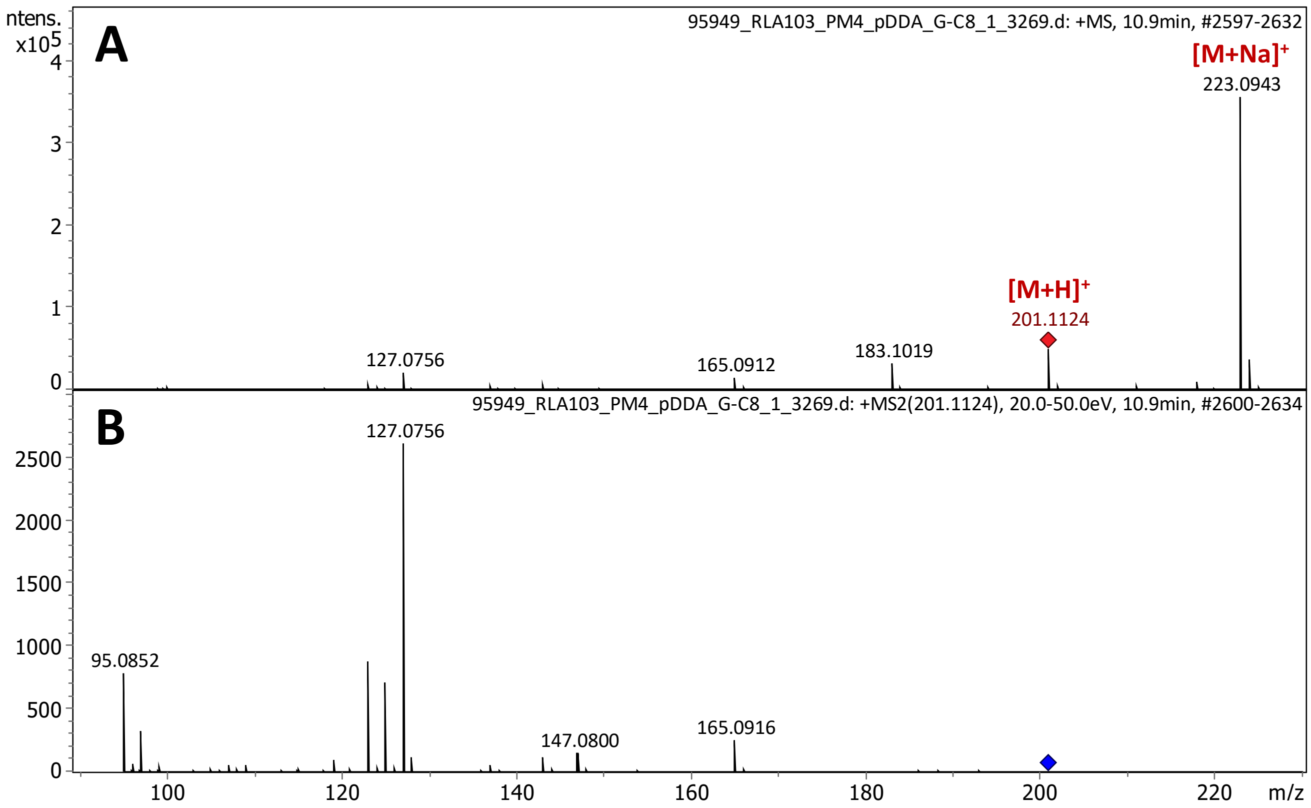

Figure S49. High resolution ESI-Qq-TOF mass spectrum of the Nonactic acid congener **49** in strain RLA103 grown in PM4 (A) and high resolution MS/MS spectrum of its $[M+H]^+$ ion (B).

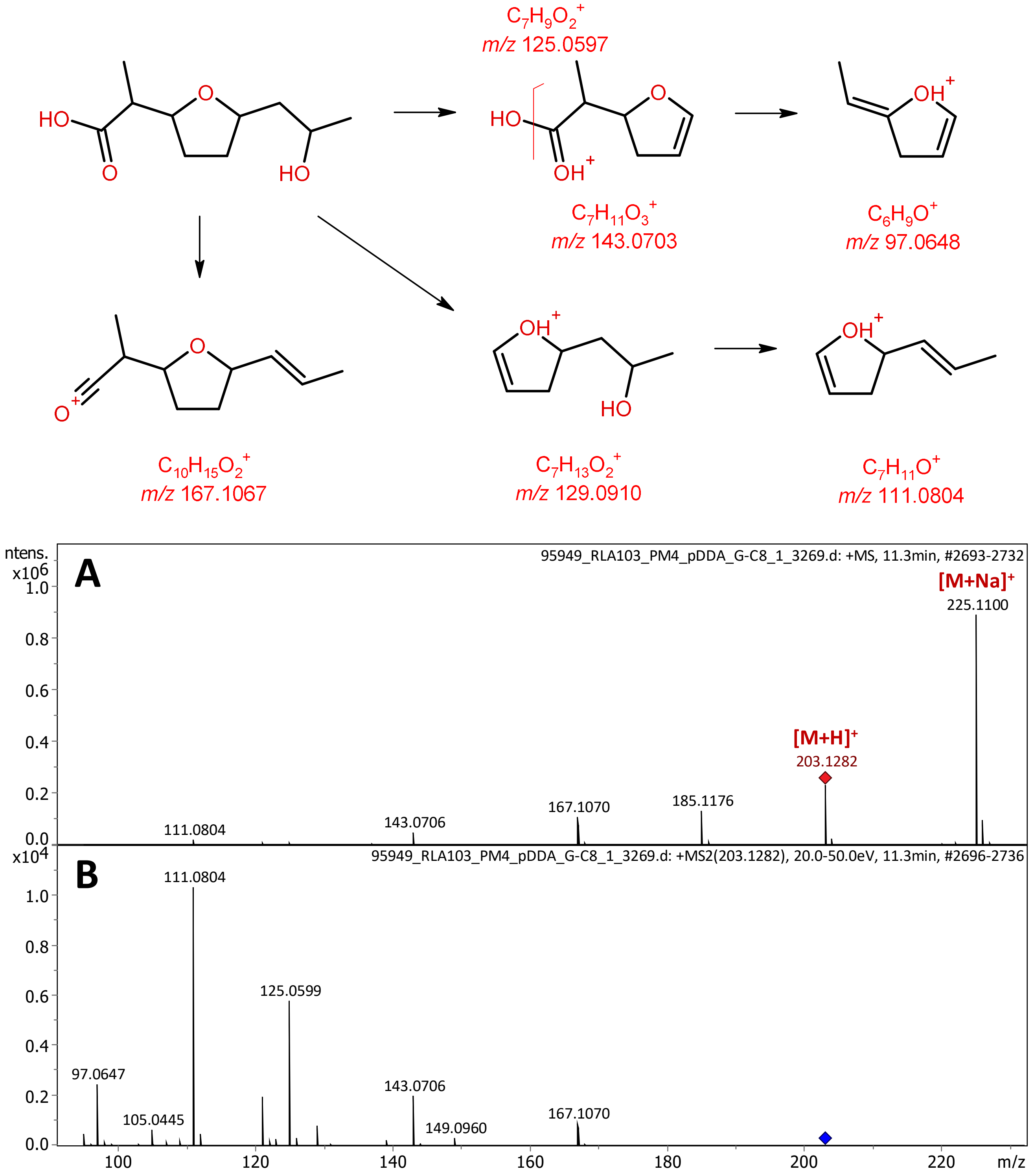

Figure S50. High resolution ESI-Qq-TOF mass spectrum of Nonactic acid (**50**) in strain RLA103 grown in PM4 (A) and high resolution MS/MS spectrum of its $[M+H]^+$ ion (B).

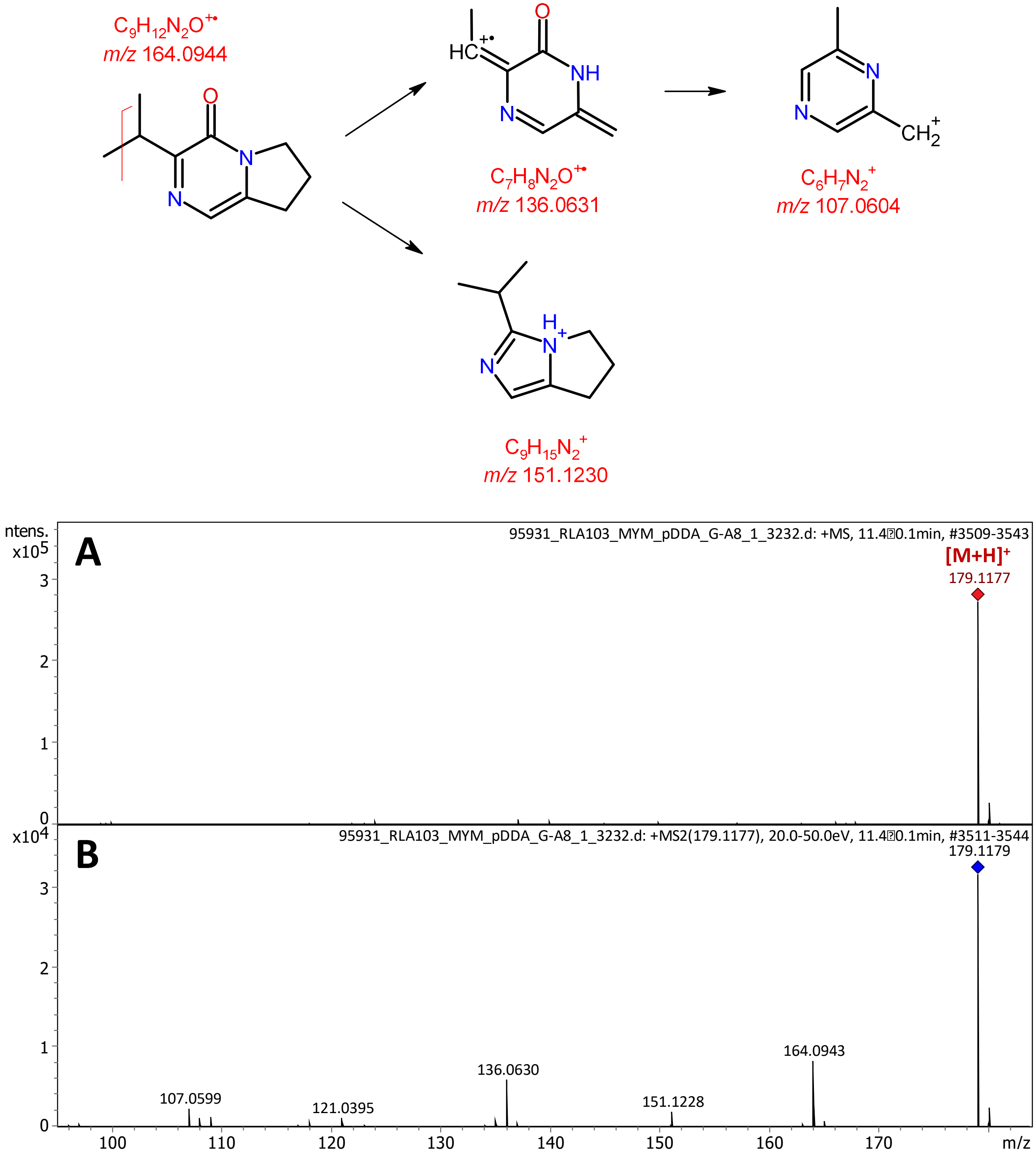

Figure S51. High resolution ESI-Qq-TOF mass spectrum of Le-pyrrolopyrazine A (**51**) in strain RLA103 grown in MYM (A) and high resolution MS/MS spectrum of its $[M+H]^+$ ion (B).

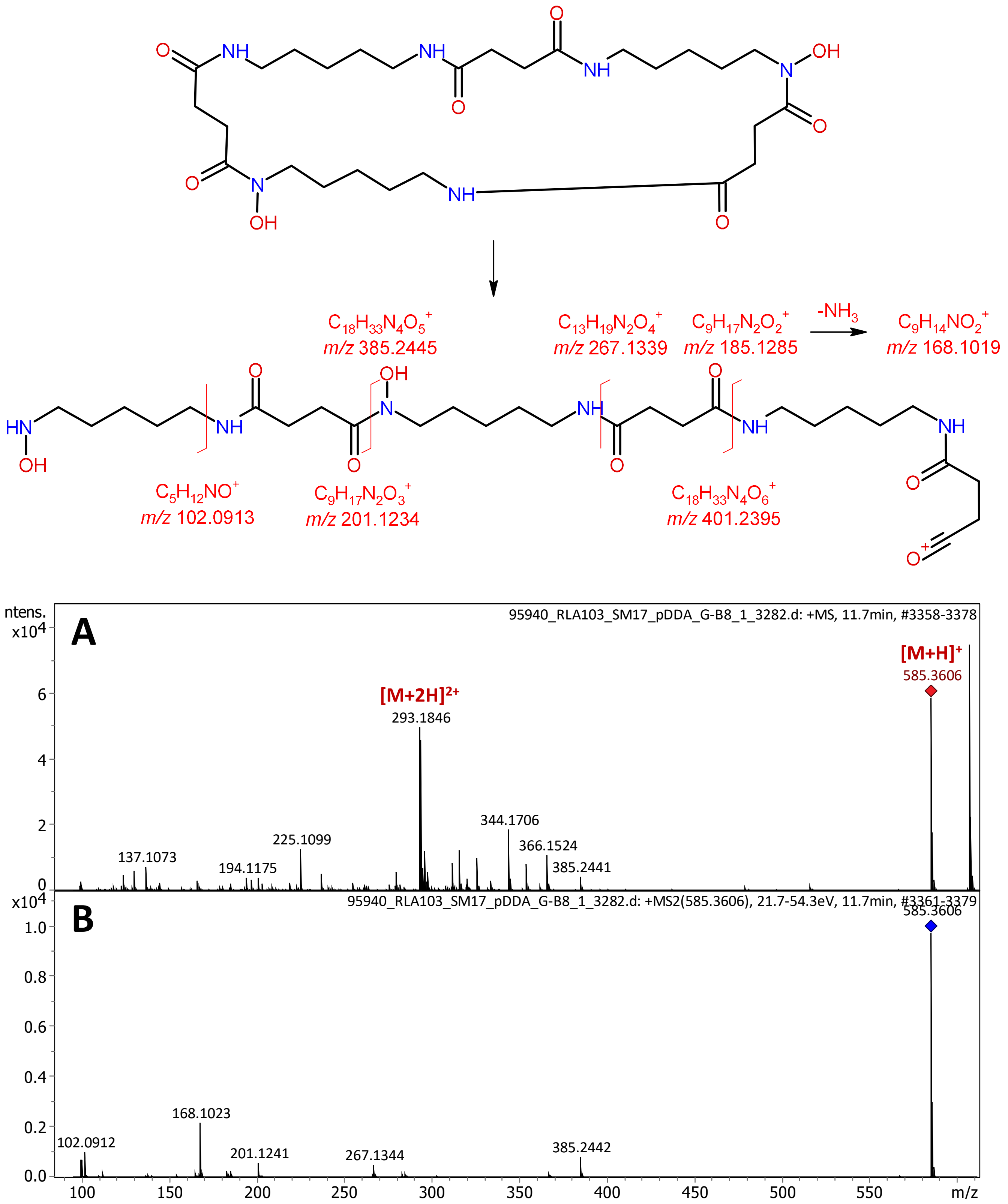

Figure S52. High resolution ESI-Qq-TOF mass spectrum of Dehydroxynocardamine (**52**) in strain RLA103 grown in SM17 (A) and high resolution MS/MS spectrum of its $[M+H]^+$ ion (B).

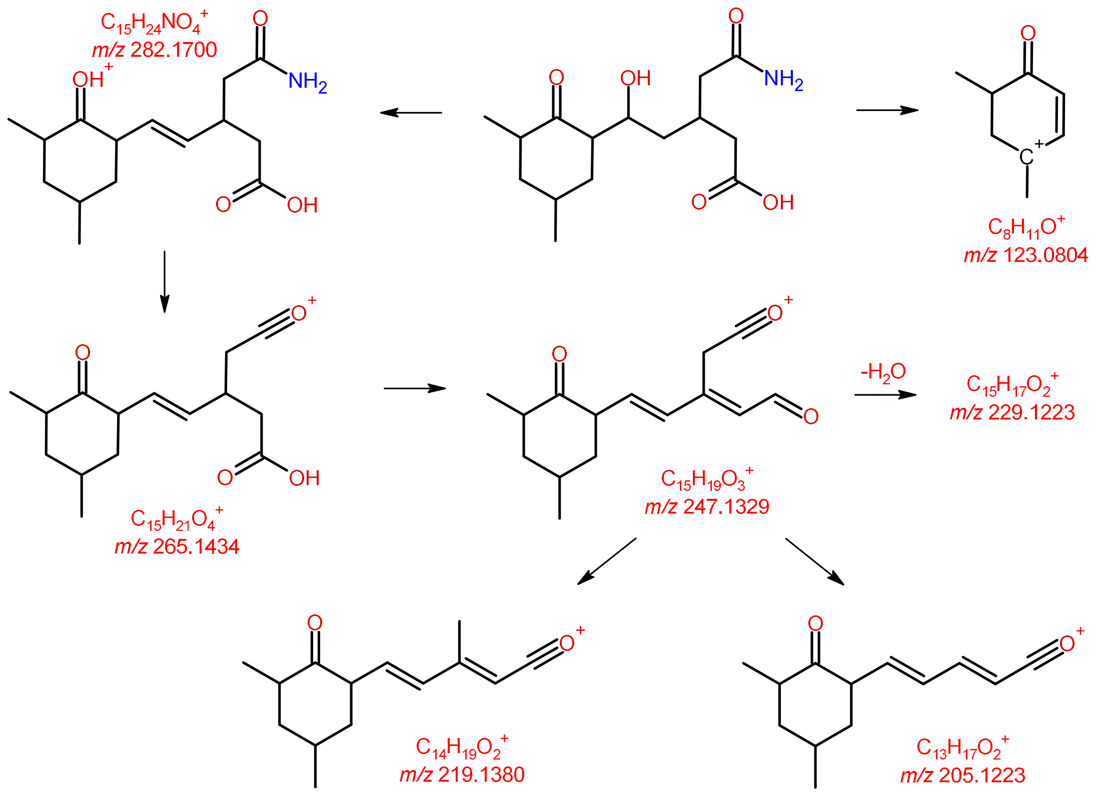

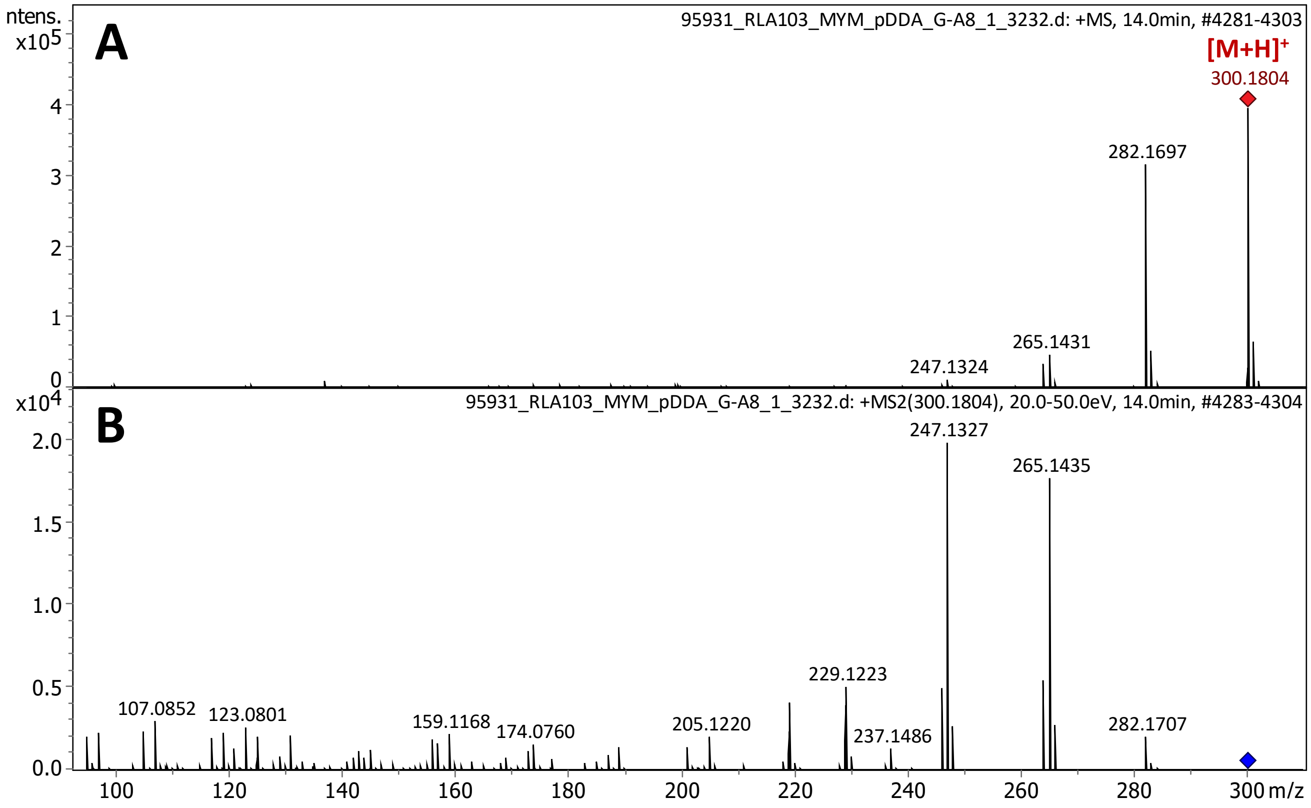

Figure S53. High resolution ESI-Qq-TOF mass spectrum of hydrolyzed Cycloheximide (**53**) in strain RLA103 grown in MYM (A) and high resolution MS/MS spectrum of its $[M+H]^+$ ion (B).

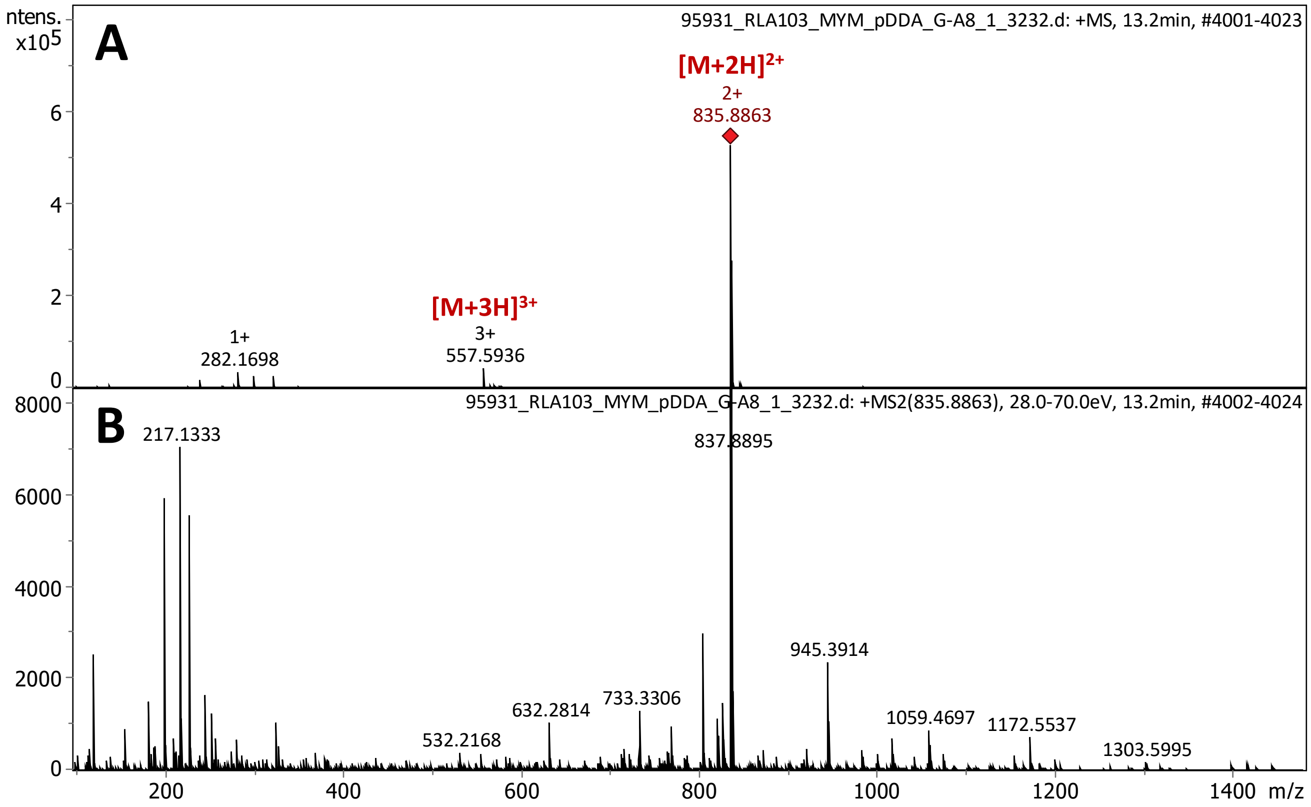

Figure S54. High resolution ESI-Qq-TOF mass spectrum of the potentially new RiPP **54** in strain RLA103 grown in MYM (A) and high resolution MS/MS spectrum of its $[M+2H]^{2+}$ ion (B).

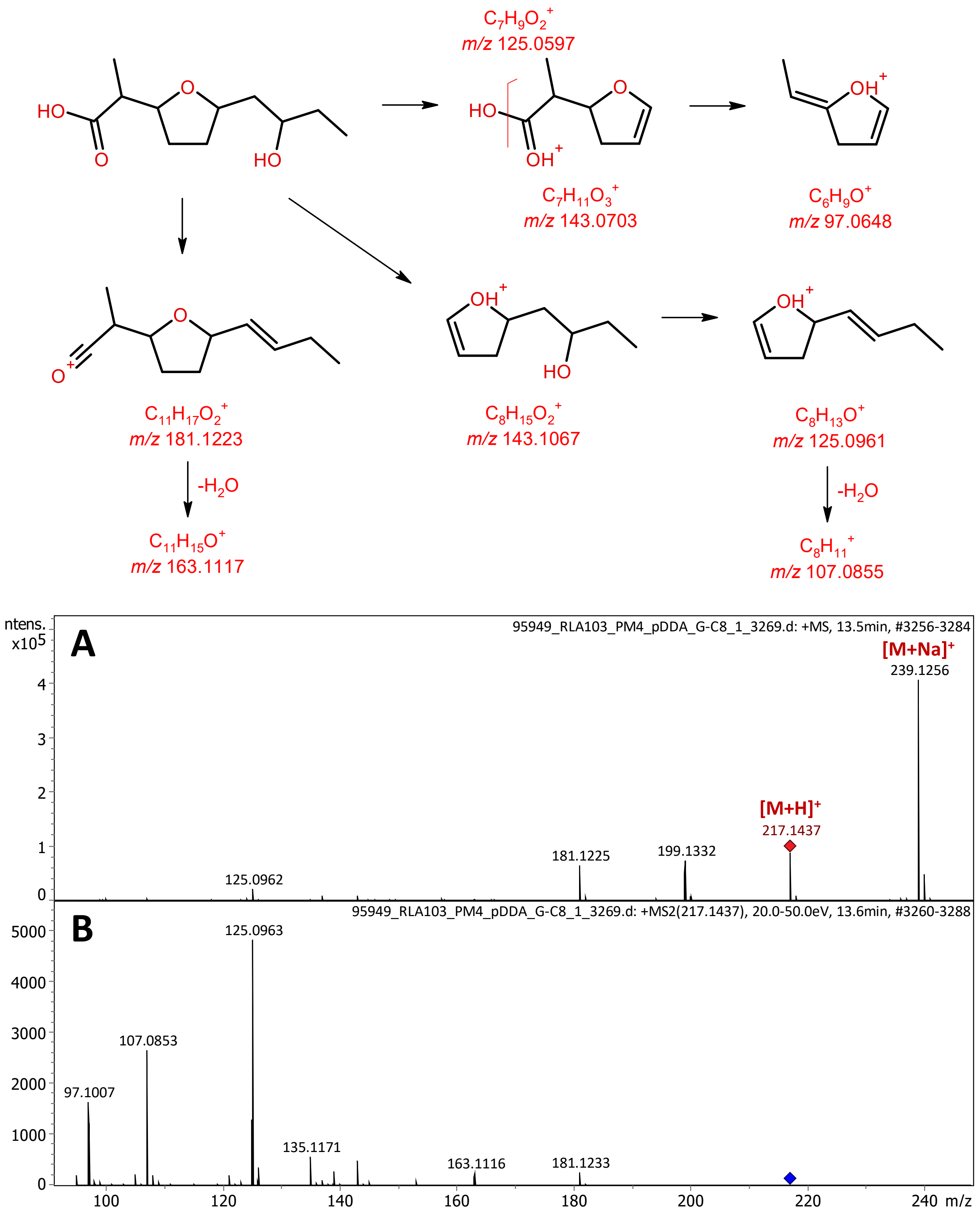

Figure S55. High resolution ESI-Qq-TOF mass spectrum of Homononactic acid (**55**) in strain RLA103 grown in PM4 (A) and high resolution MS/MS spectrum of its $[M+H]^+$ ion (B).

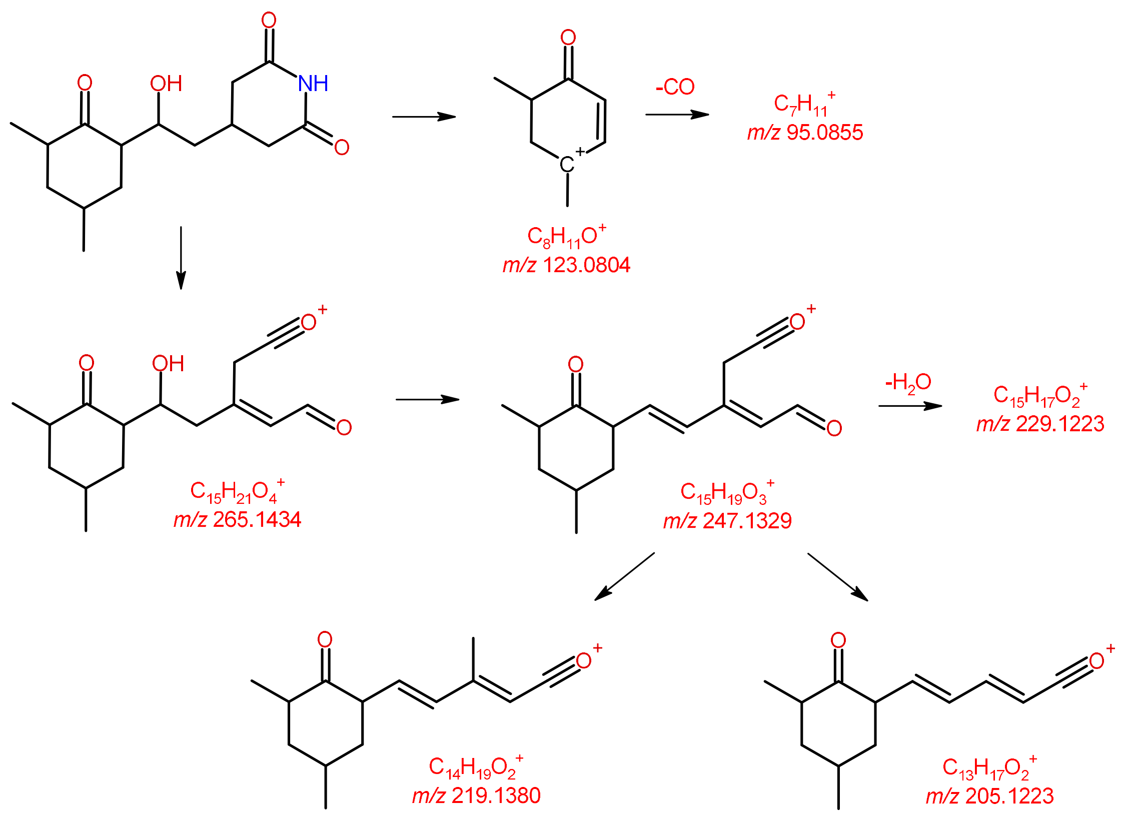

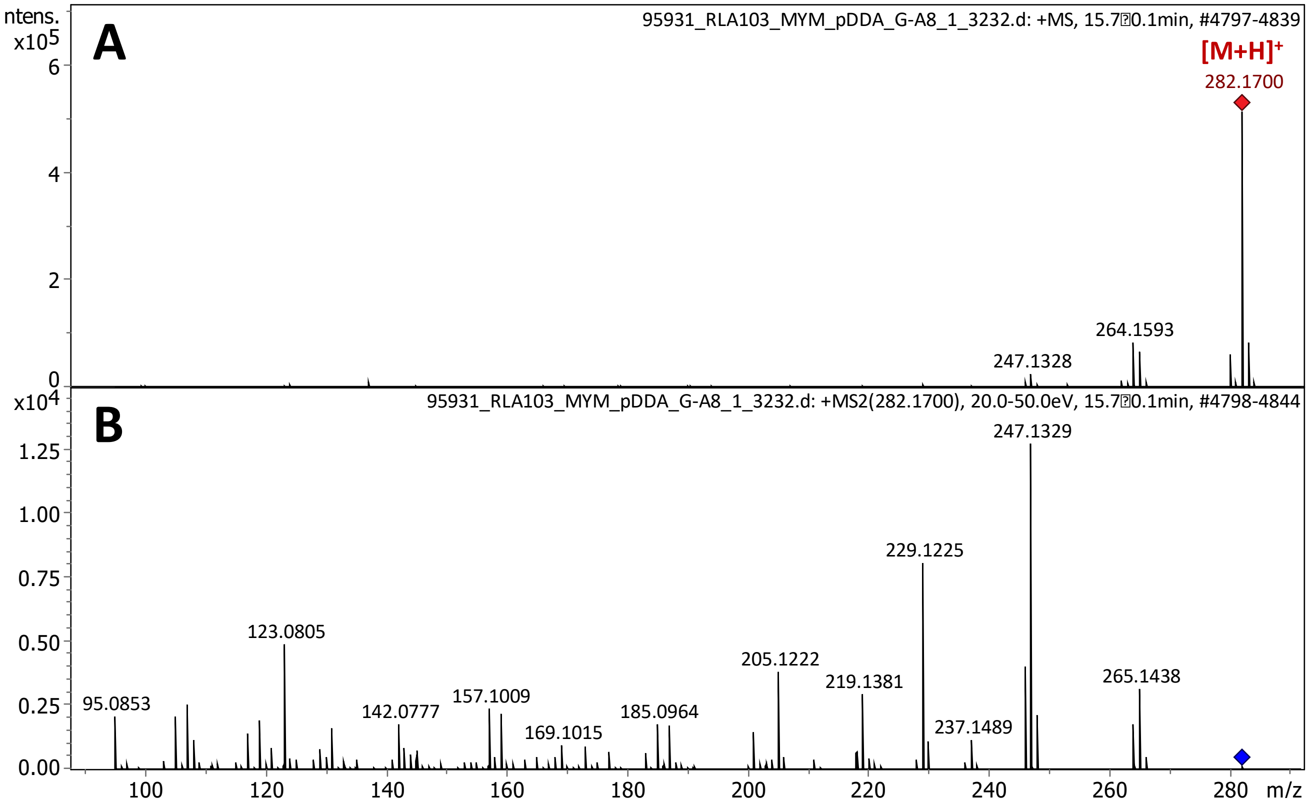

Figure S56. High resolution ESI-Qq-TOF mass spectrum of Cycloheximide (**56**) in strain RLA103 grown in MYM (A) and high resolution MS/MS spectrum of its $[M+H]^+$ ion (B).

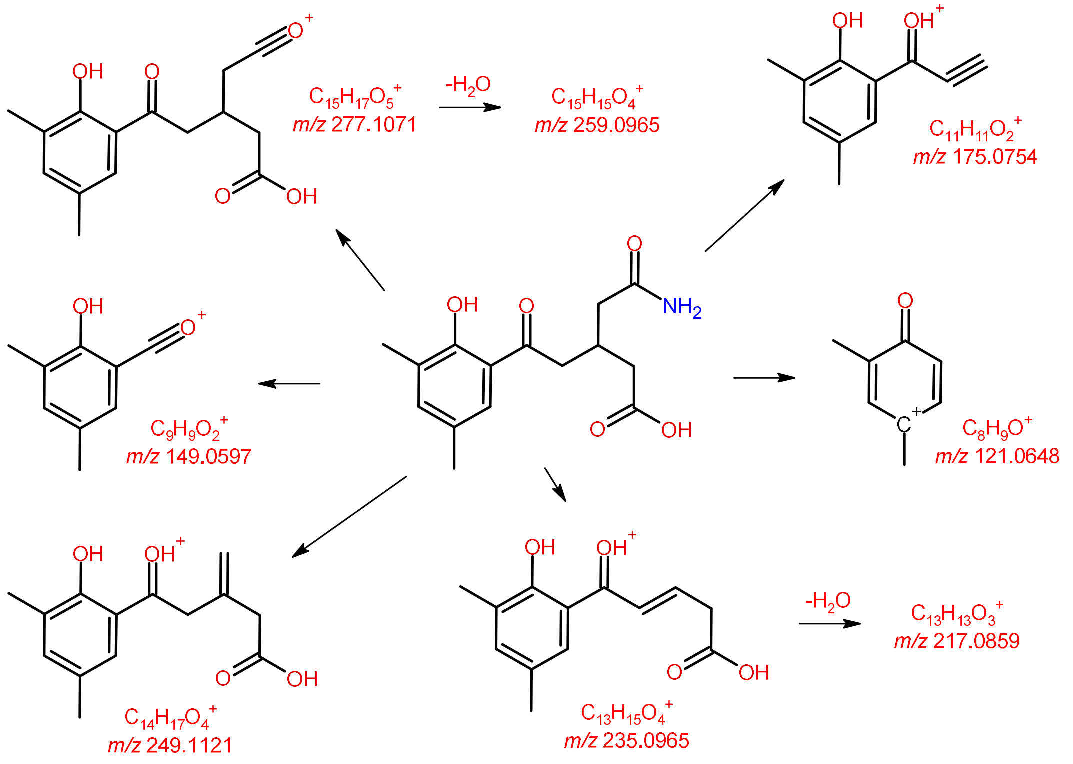

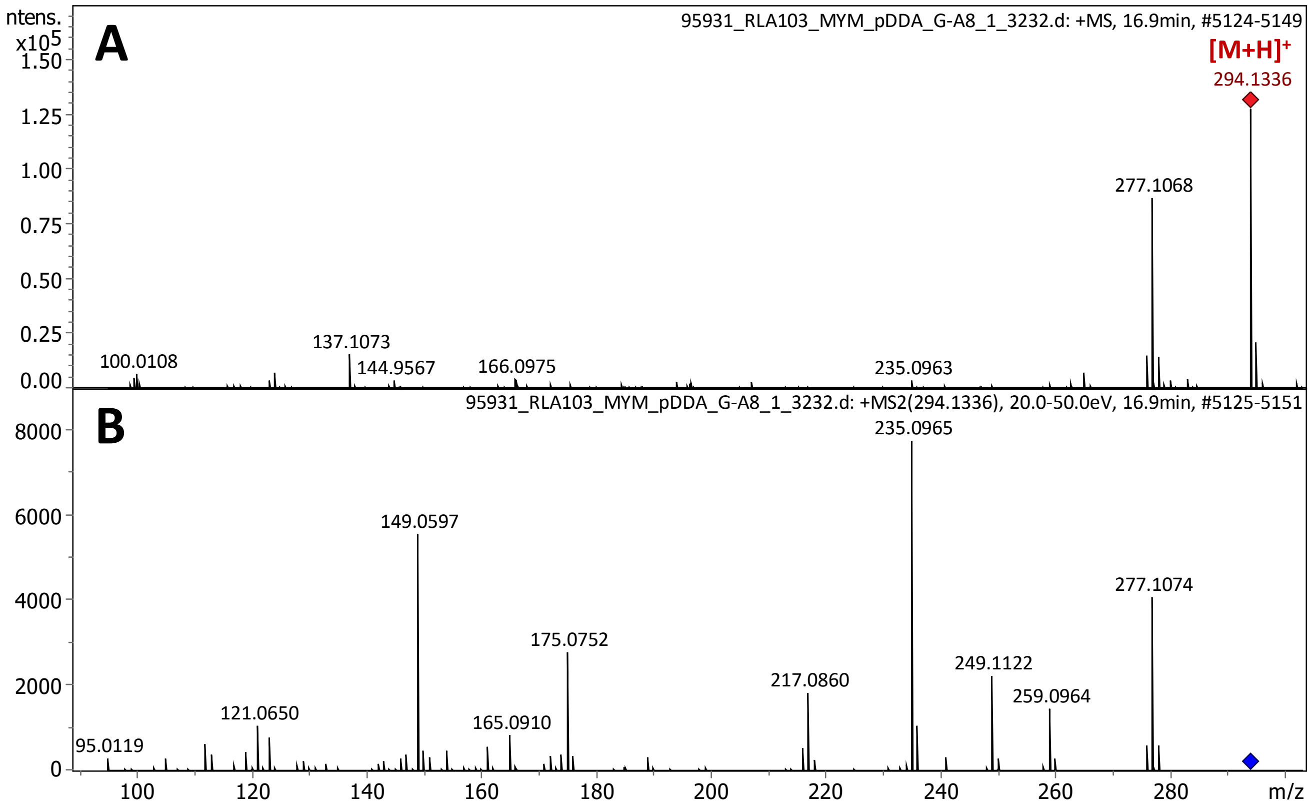

Figure S57. High resolution ESI-Qq-TOF mass spectrum of Phenatic acid A (**57**) in strain RLA103 grown in MYM (A) and high resolution MS/MS spectrum of its $[M+H]^+$ ion (B).

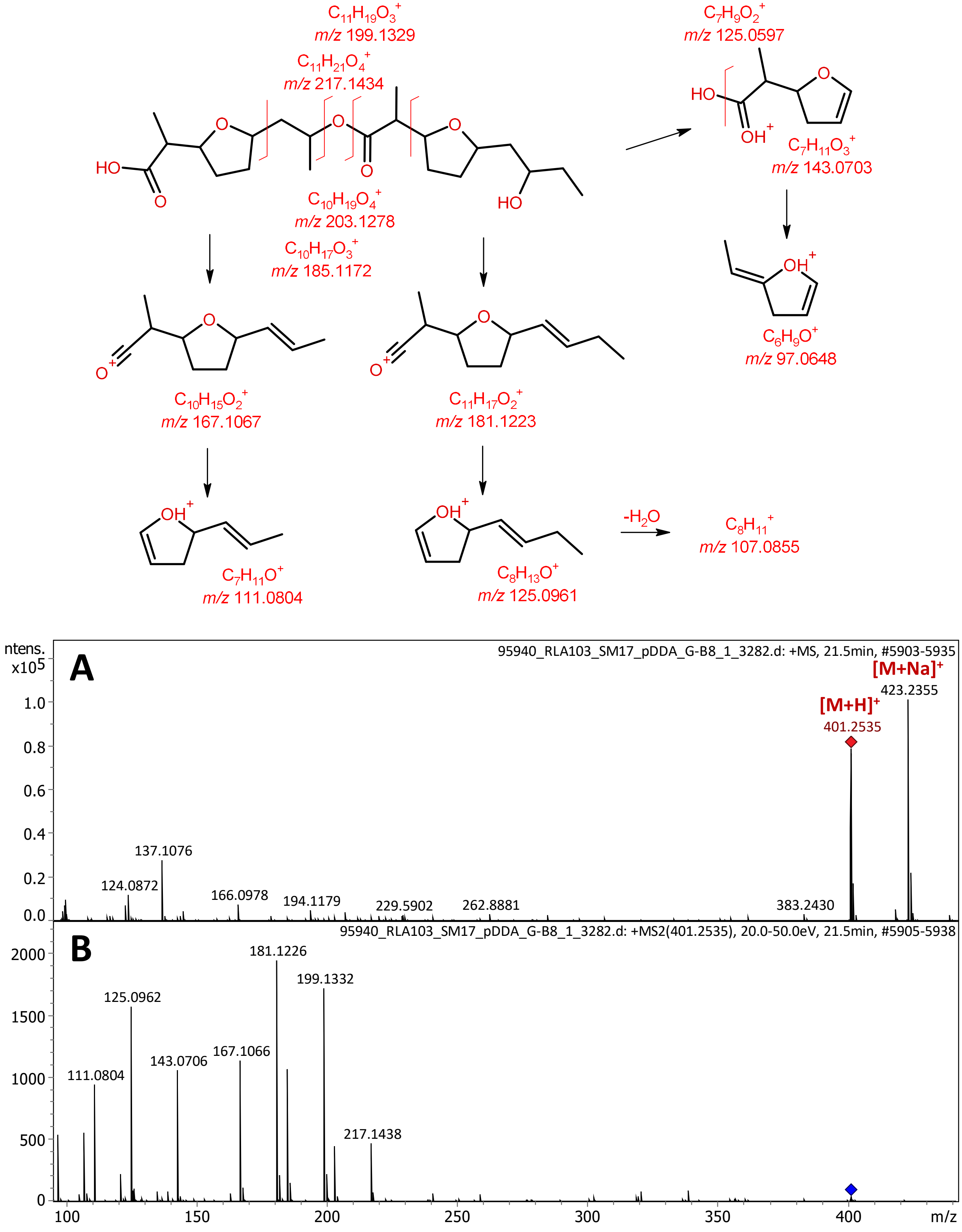

Figure S58. High resolution ESI-Qq-TOF mass spectrum of Bonactin (**58**) in strain RLA103 grown in SM17 (A) and high resolution MS/MS spectrum of its $[M+H]^+$ ion (B).

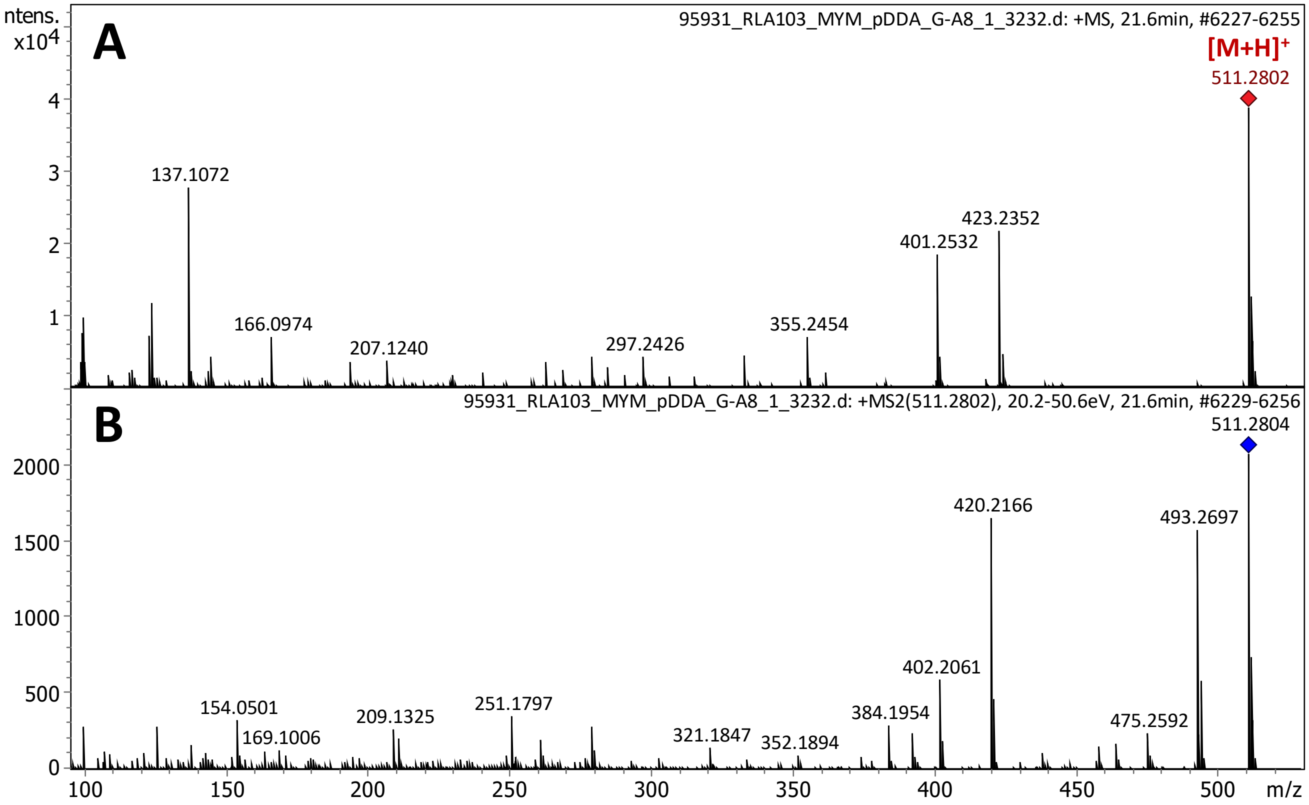

Figure S59. High resolution ESI-Qq-TOF mass spectrum of the polycyclic tetramate macrolactam **59** in strain RLA103 grown in MYM (A) and high resolution MS/MS spectrum of its $[M+H]^+$ ion (B).

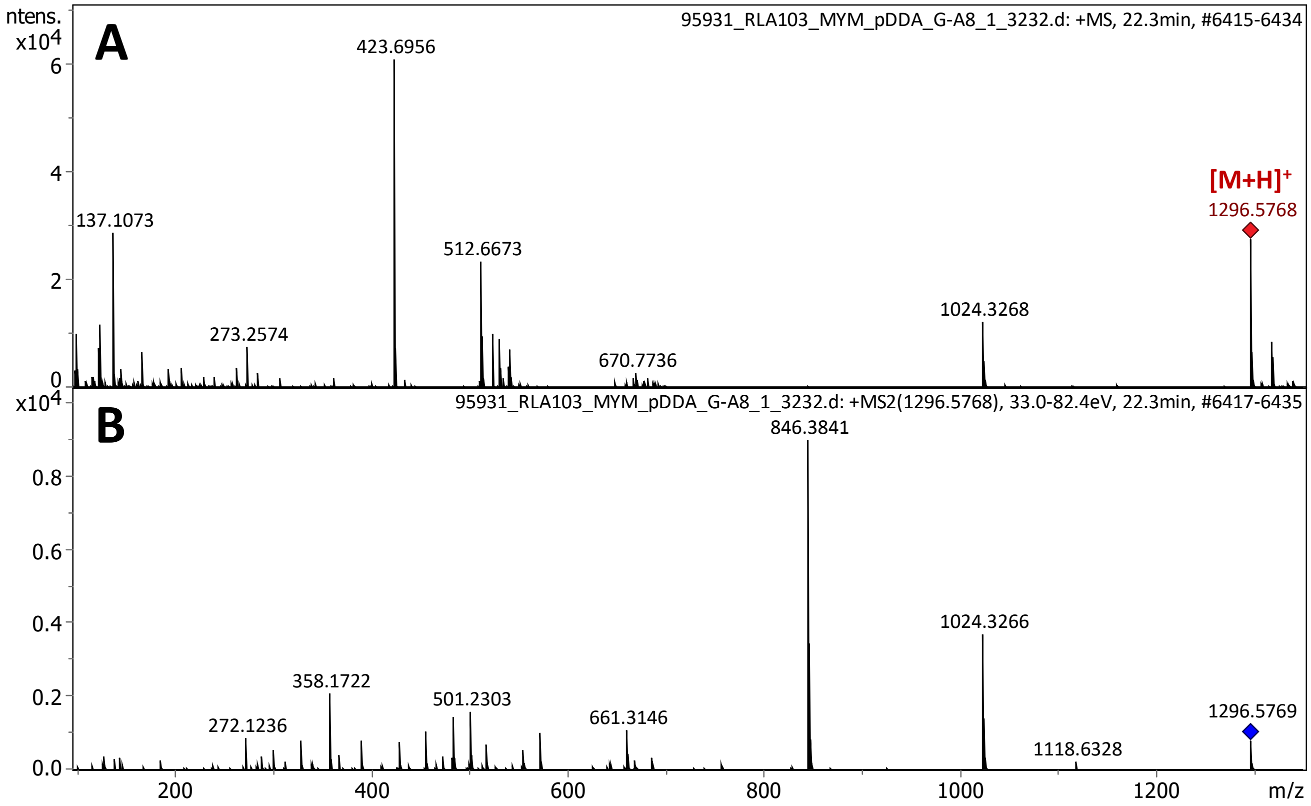

Figure S60. High resolution ESI-Qq-TOF mass spectrum of the potentially new natural product **60** in strain RLA103 grown in MYM (A) and high resolution MS/MS spectrum of its $[M+H]^+$ ion (B).

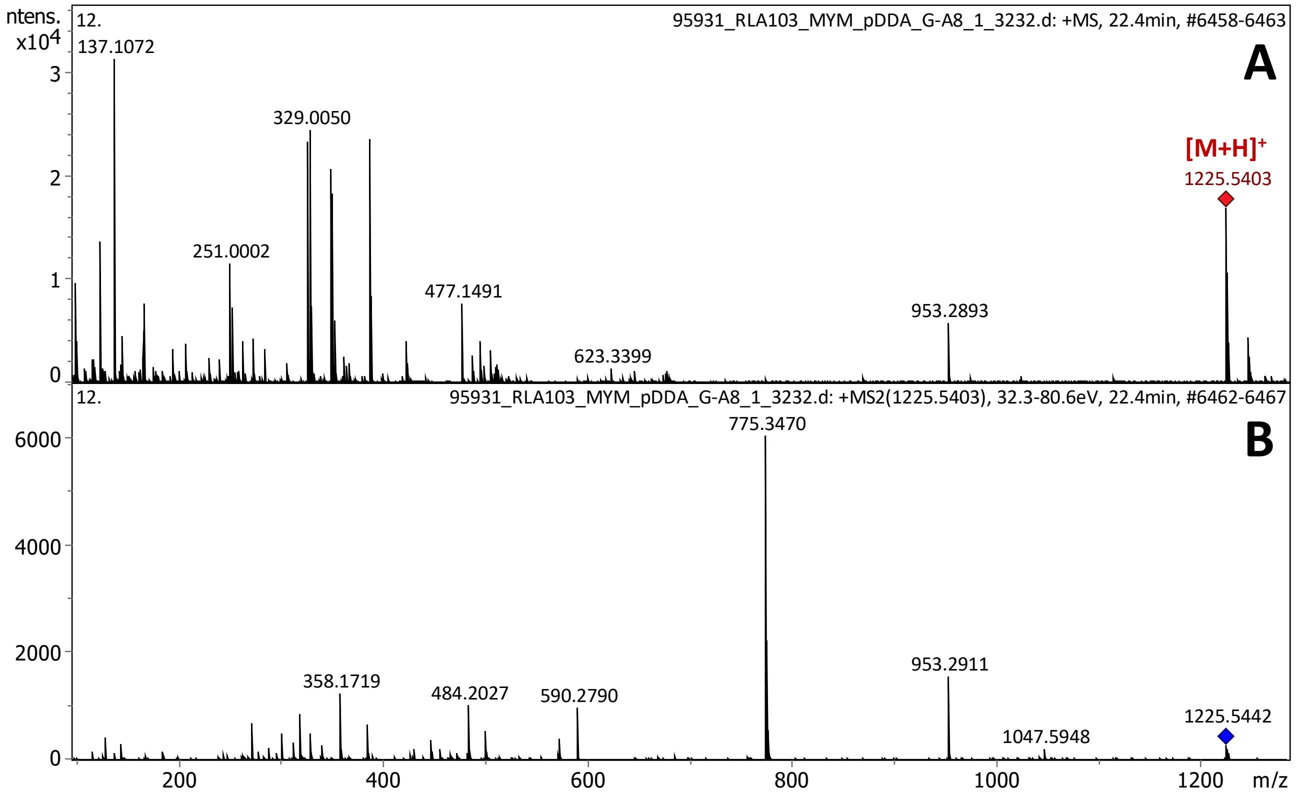

Figure S61. High resolution ESI-Qq-TOF mass spectrum of the potentially new natural product **61** in strain RLA103 grown in MYM (A) and high resolution MS/MS spectrum of its $[M+H]^+$ ion (B).

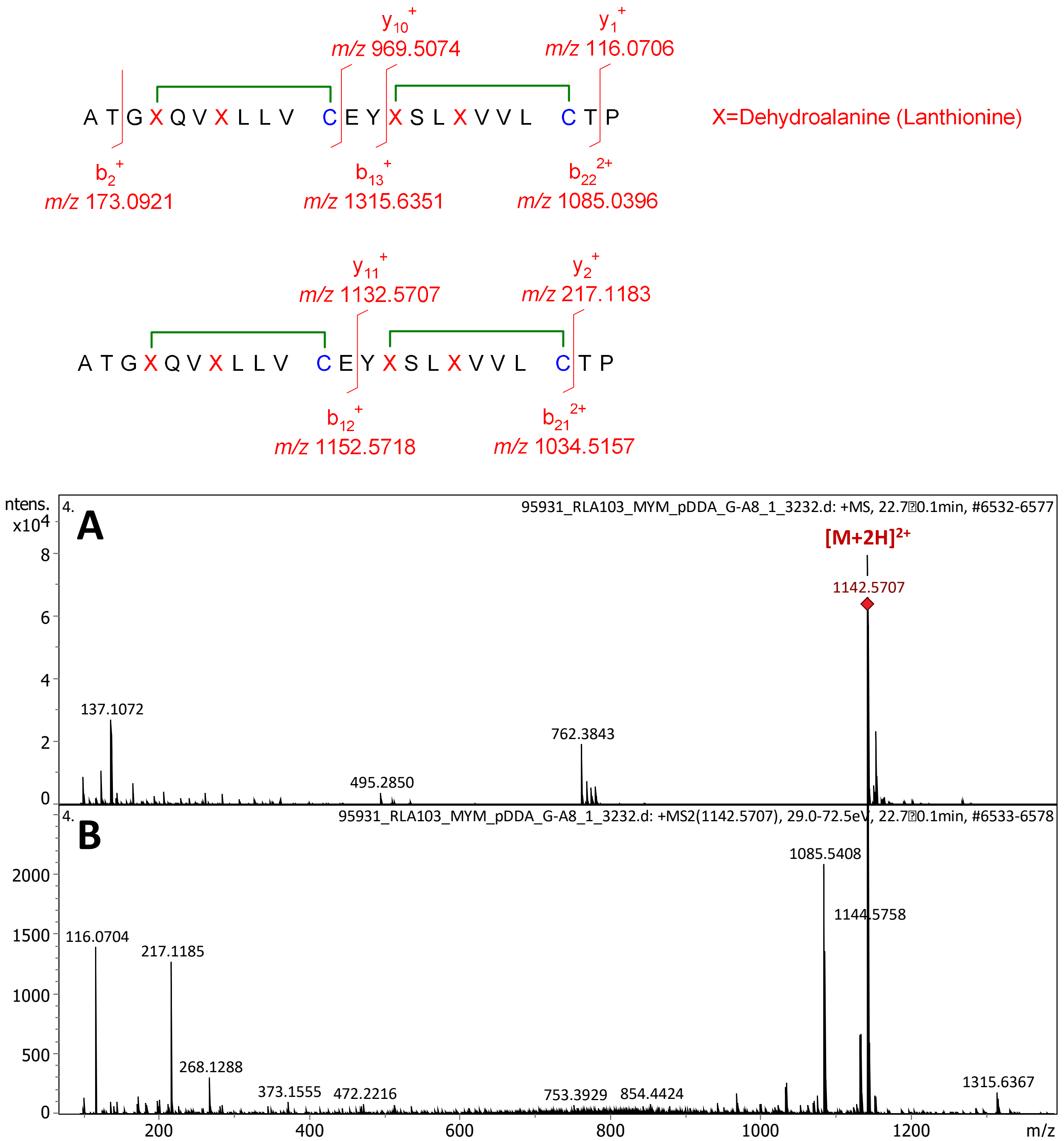

Figure S62. High resolution ESI-Qq-TOF mass spectrum of the AmfS congener **62** in strain RLA103 grown in MYM (A) and high resolution MS/MS spectrum of its $[M+2H]^{2+}$ ion (B).

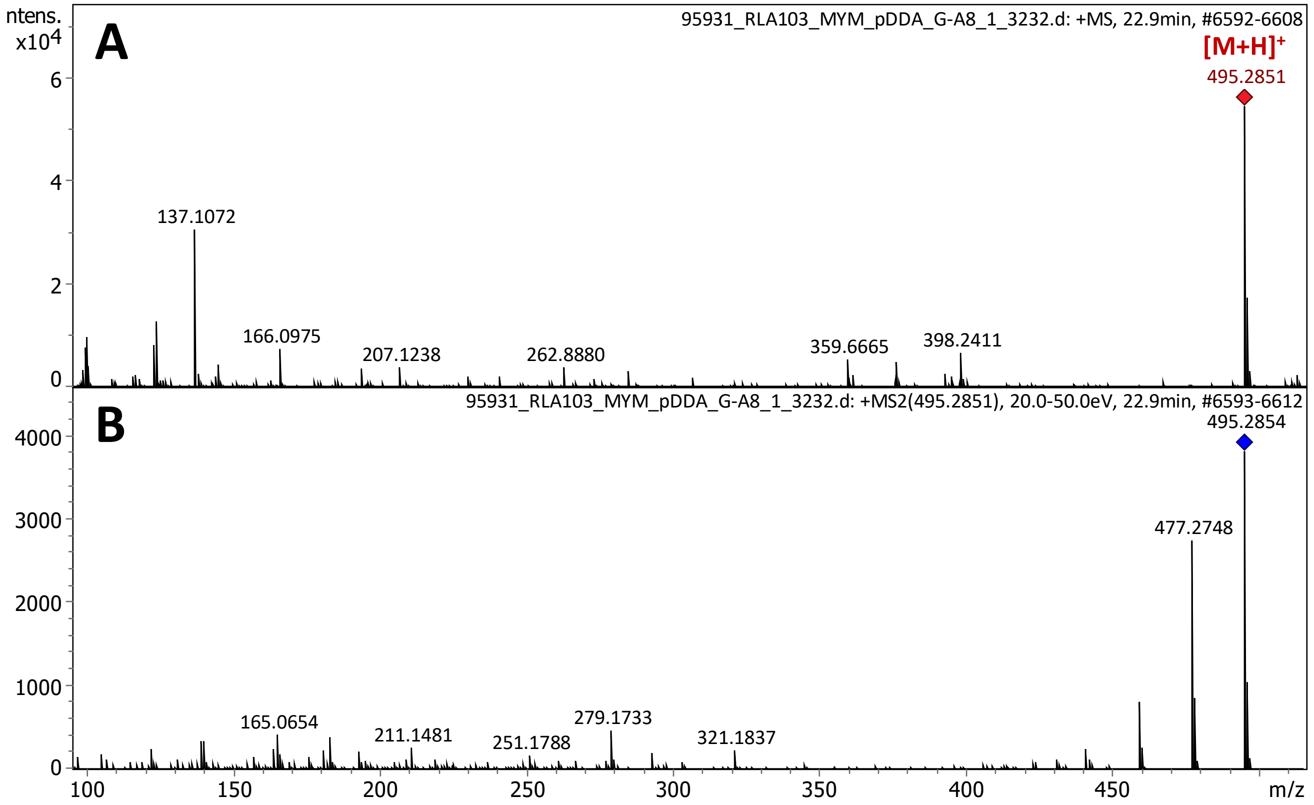

Figure S63. High resolution ESI-Qq-TOF mass spectrum of the polycyclic tetramate macrolactam **63** in strain RLA103 grown in MYM (A) and high resolution MS/MS spectrum of its $[M+H]^+$ ion (B).

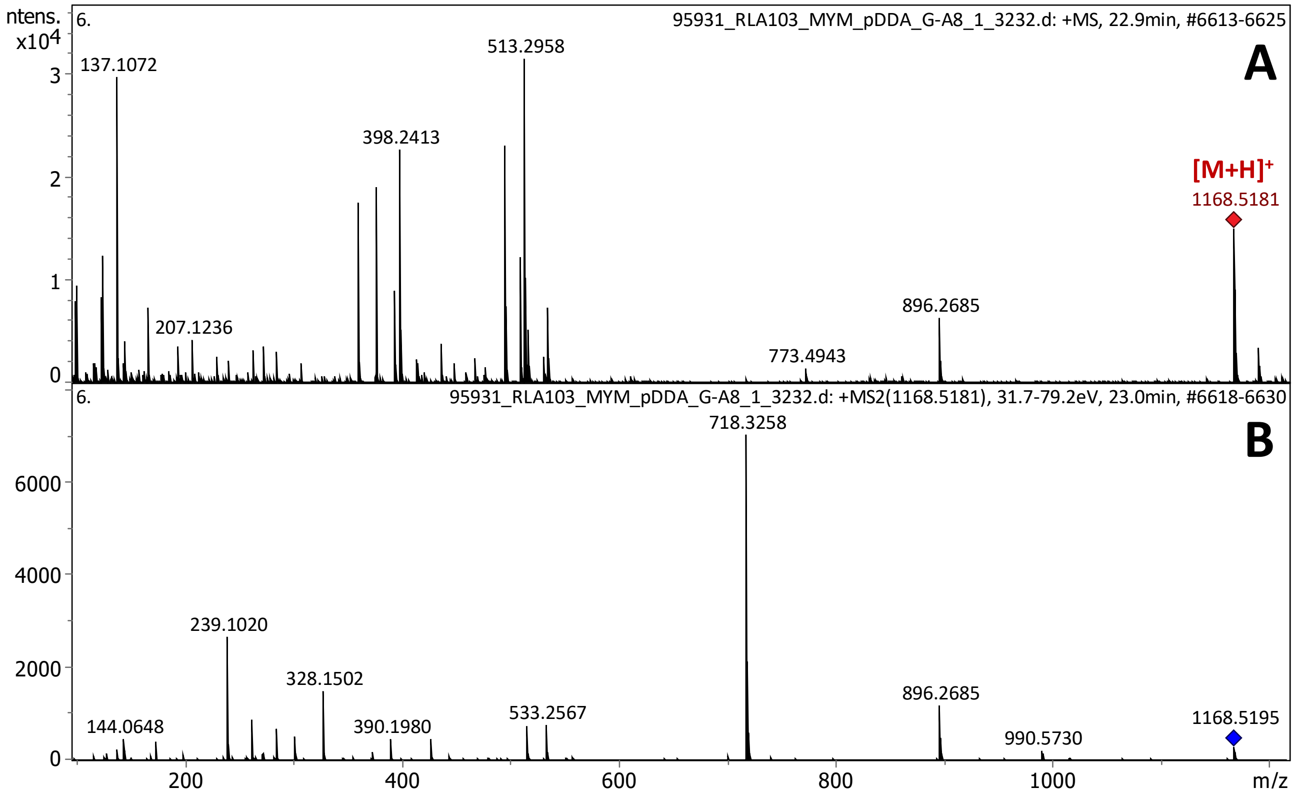

Figure S64. High resolution ESI-Qq-TOF mass spectrum of the potentially new natural product **64** in strain RLA103 grown in MYM (A) and high resolution MS/MS spectrum of its $[M+H]^+$ ion (B).

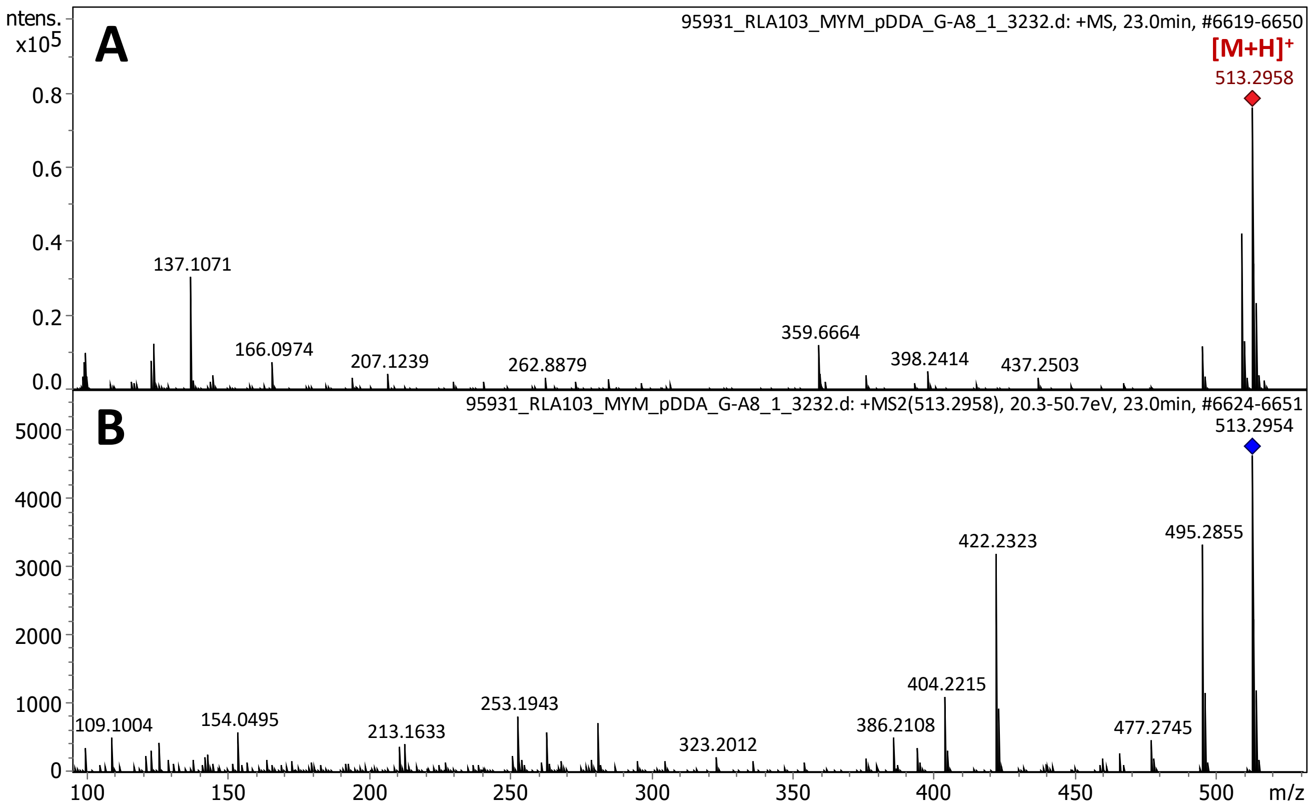

Figure S65. High resolution ESI-Qq-TOF mass spectrum of the polycyclic tetramate macrolactam **65** in strain RLA103 grown in MYM (A) and high resolution MS/MS spectrum of its $[M+H]^+$ ion (B).

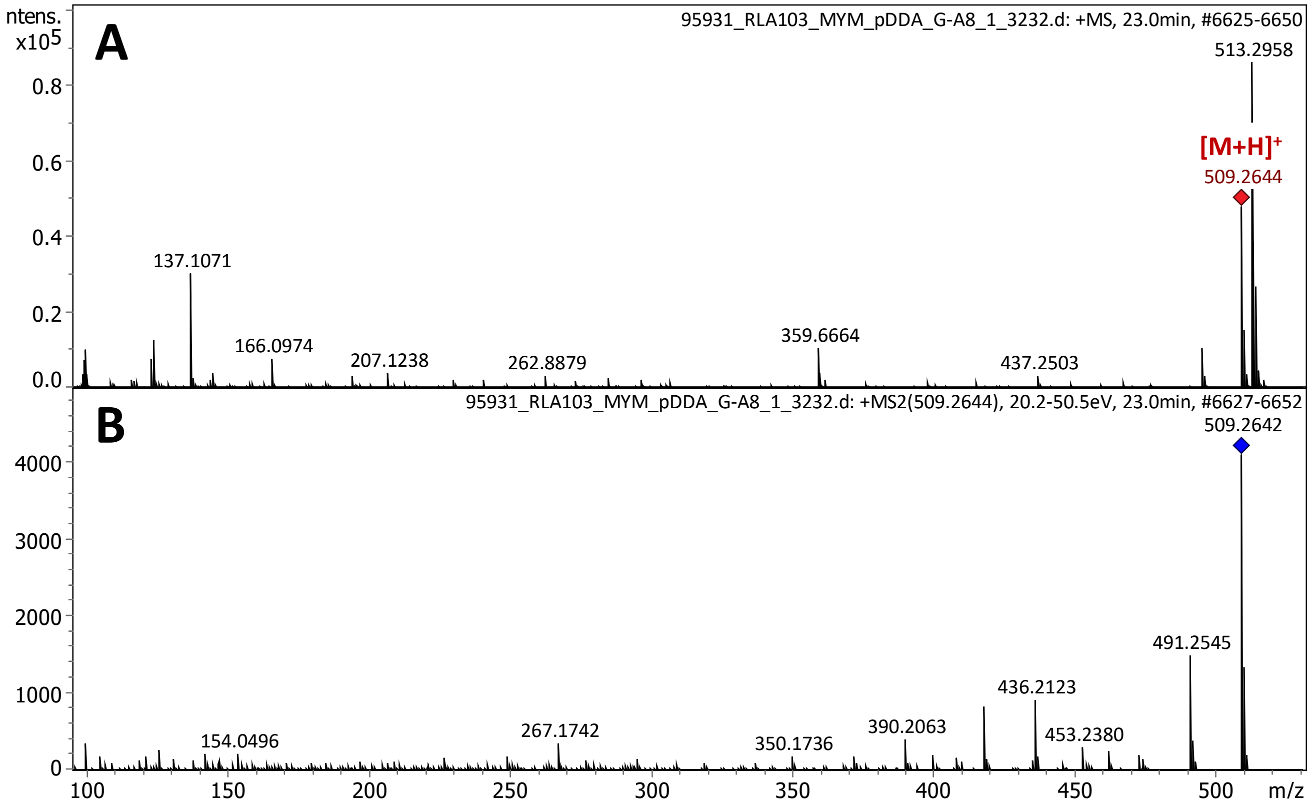

Figure S66. High resolution ESI-Qq-TOF mass spectrum of the polycyclic tetramate macrolactam **66** in strain RLA103 grown in MYM (A) and high resolution MS/MS spectrum of its $[M+H]^+$ ion (B).

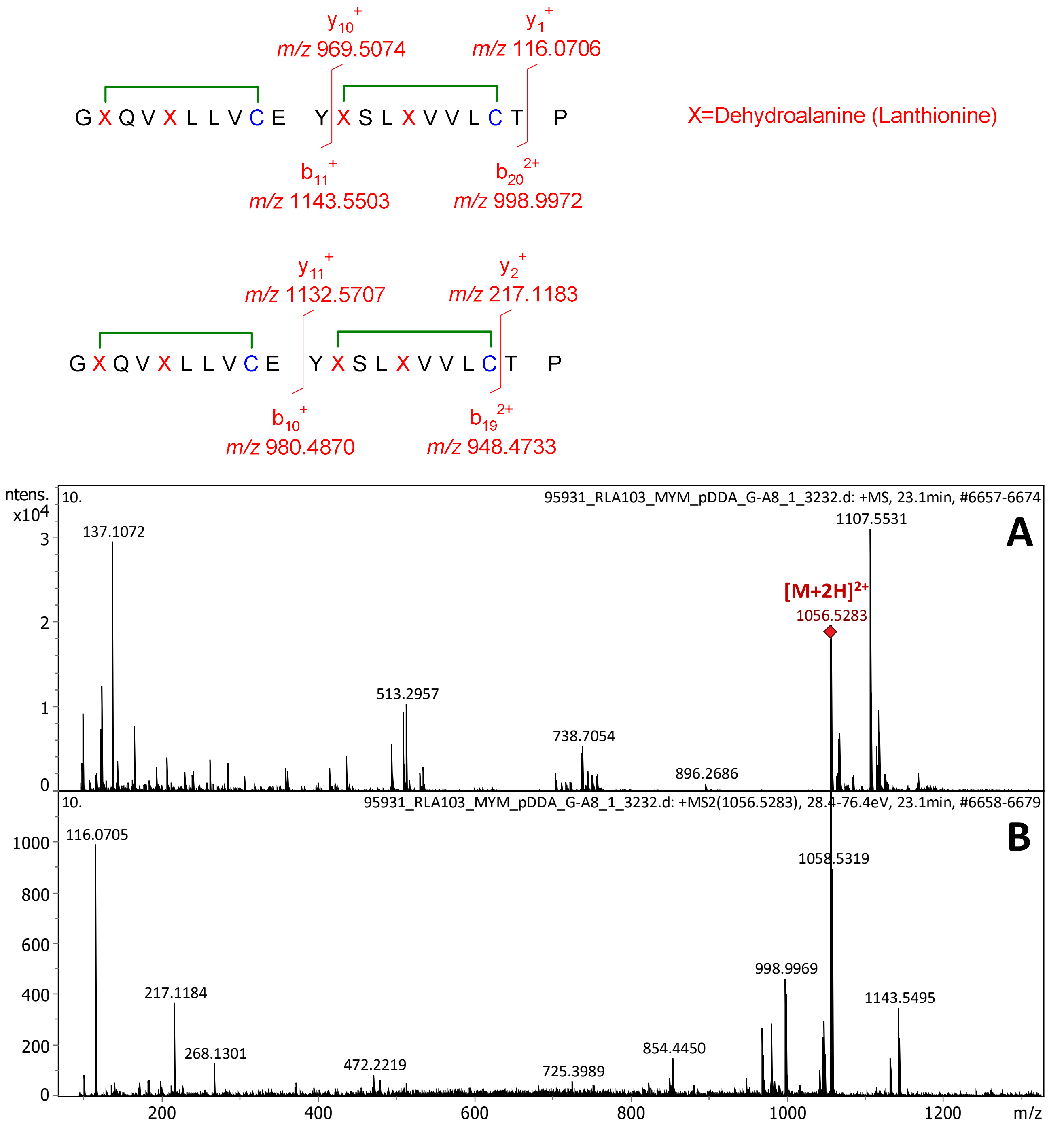

Figure S67. High resolution ESI-Qq-TOF mass spectrum of the AmfS congener **67** in strain RLA103 grown in MYM (A) and high resolution MS/MS spectrum of its $[M+2H]^{2+}$ ion (B).

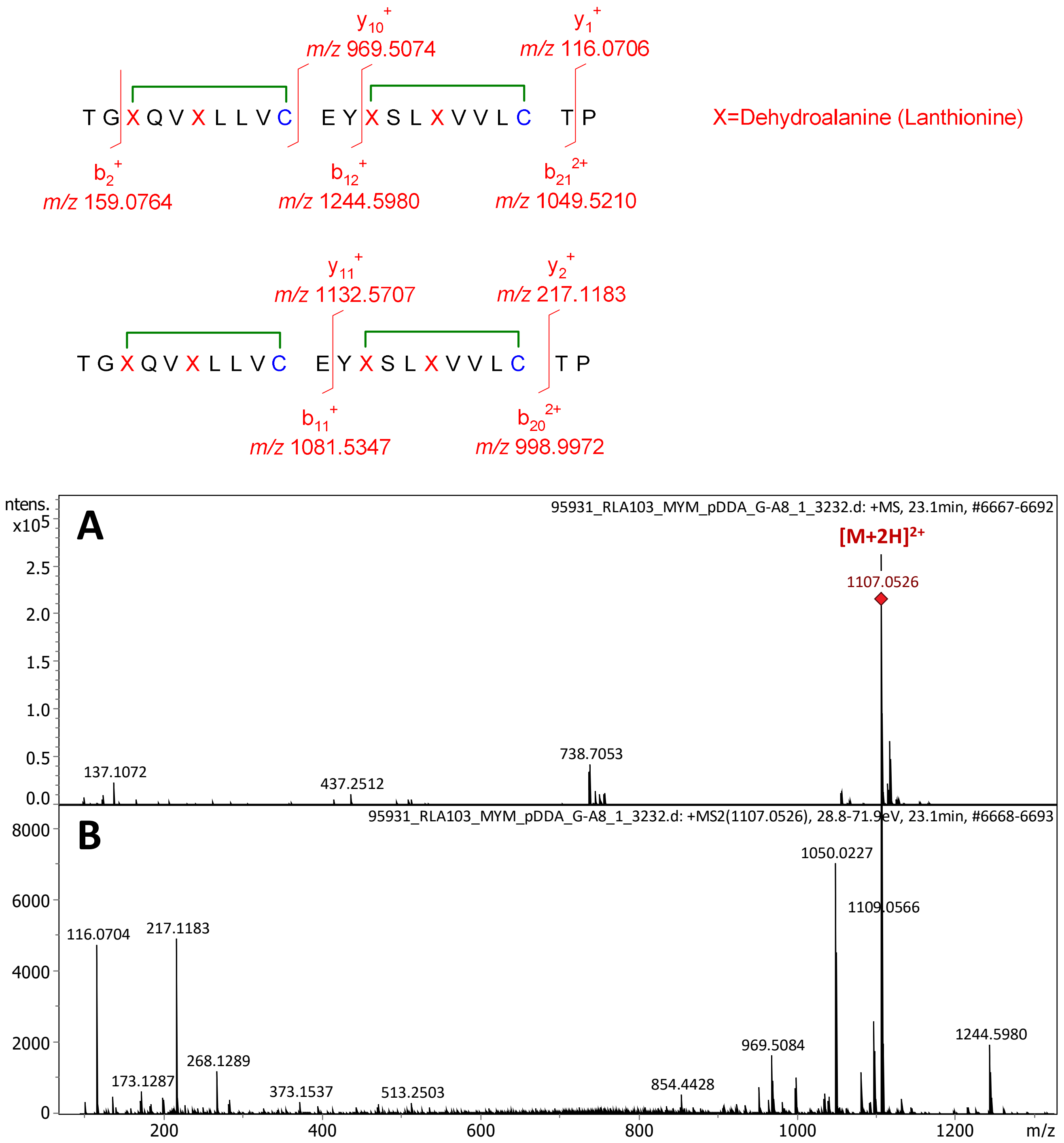

Figure S68. High resolution ESI-Qq-TOF mass spectrum of AmfS (**68**) in strain RLA103 grown in MYM (A) and high resolution MS/MS spectrum of its $[M+2H]^{2+}$ ion (B).

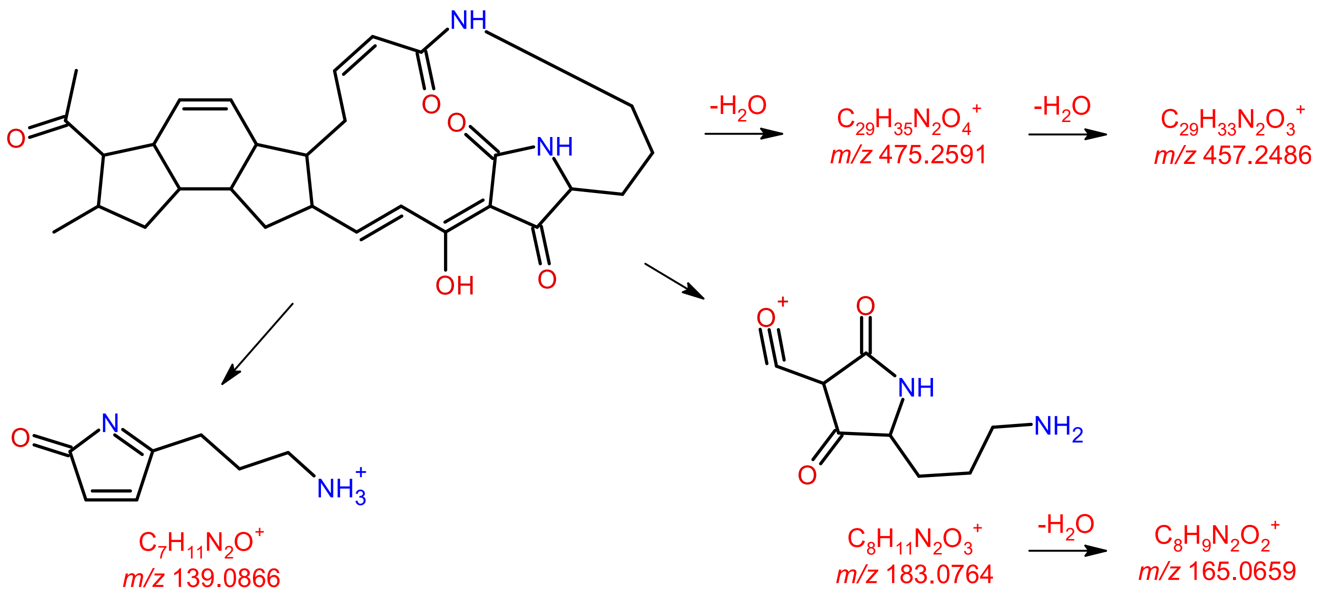

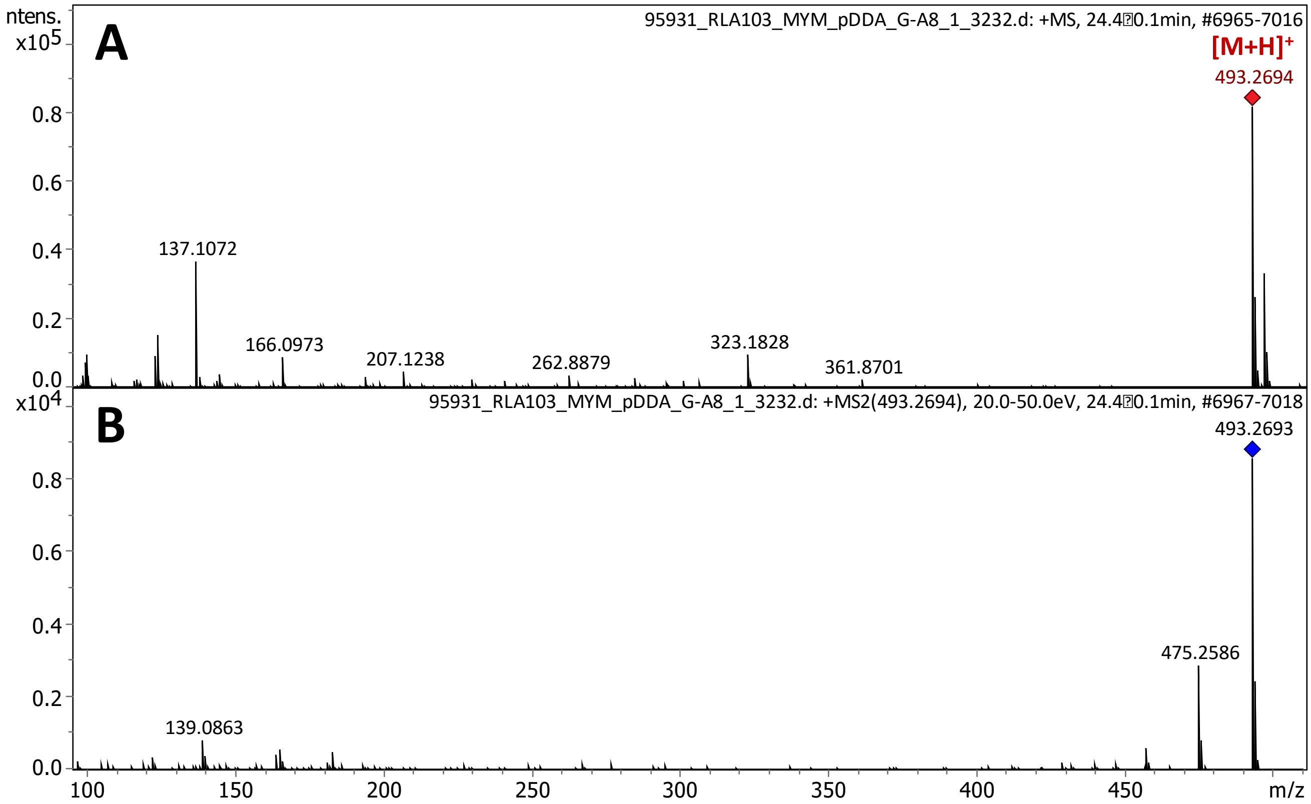

Figure S69. High resolution ESI-Qq-TOF mass spectrum of Clifednamide A (**69**) in strain RLA103 grown in MYM (A) and high resolution MS/MS spectrum of its $[M+H]^+$ ion (B).

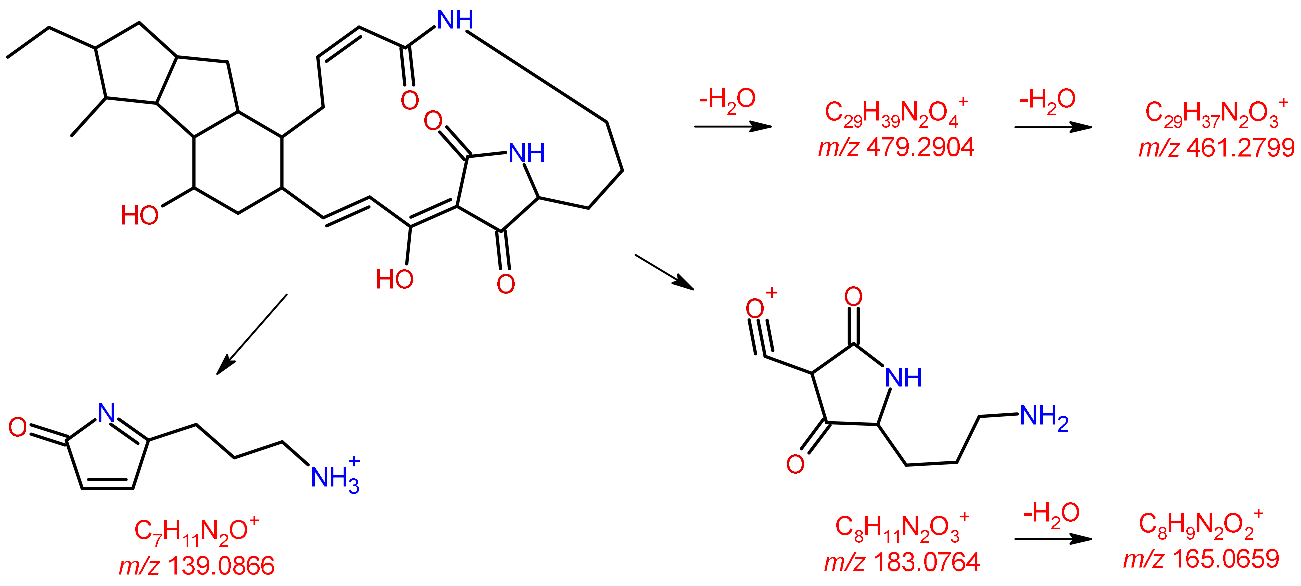

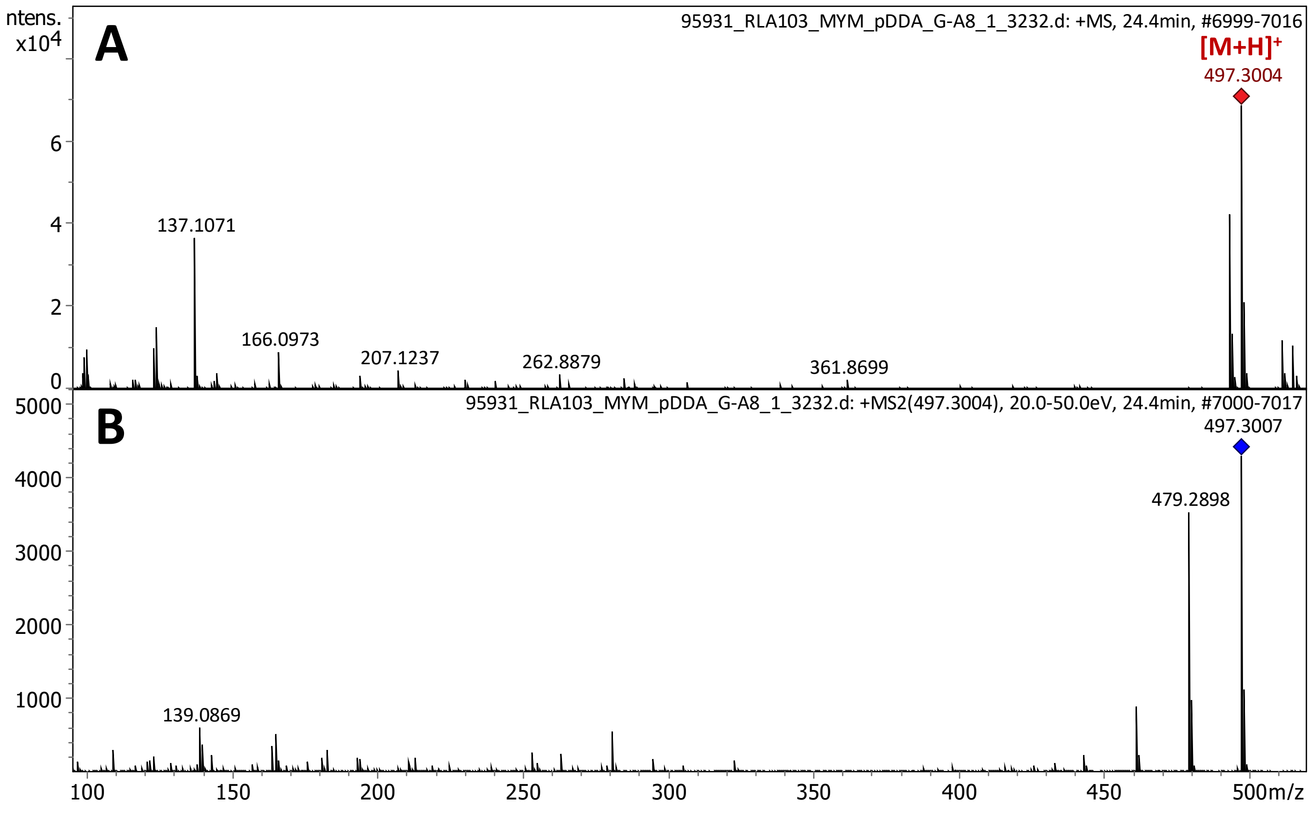

Figure S70. High resolution ESI-Qq-TOF mass spectrum of 10-epi-3-deOH-HSAF (**70**) in strain RLA103 grown in MYM (A) and high resolution MS/MS spectrum of its $[M+H]^+$ ion (B).

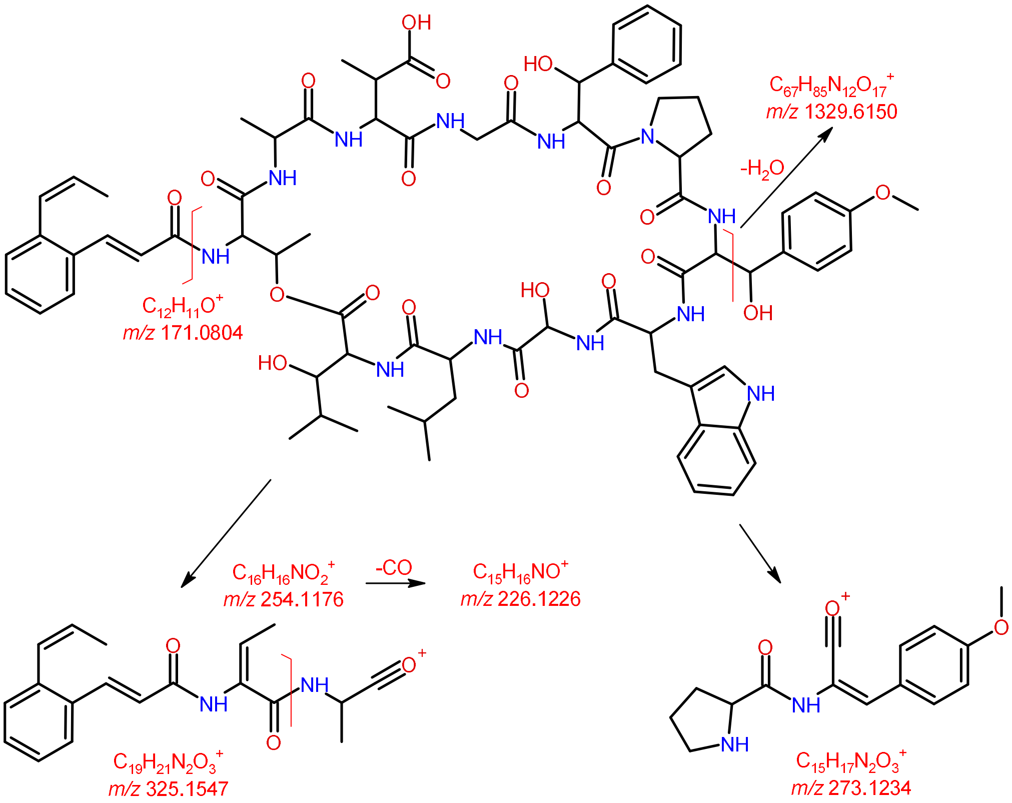

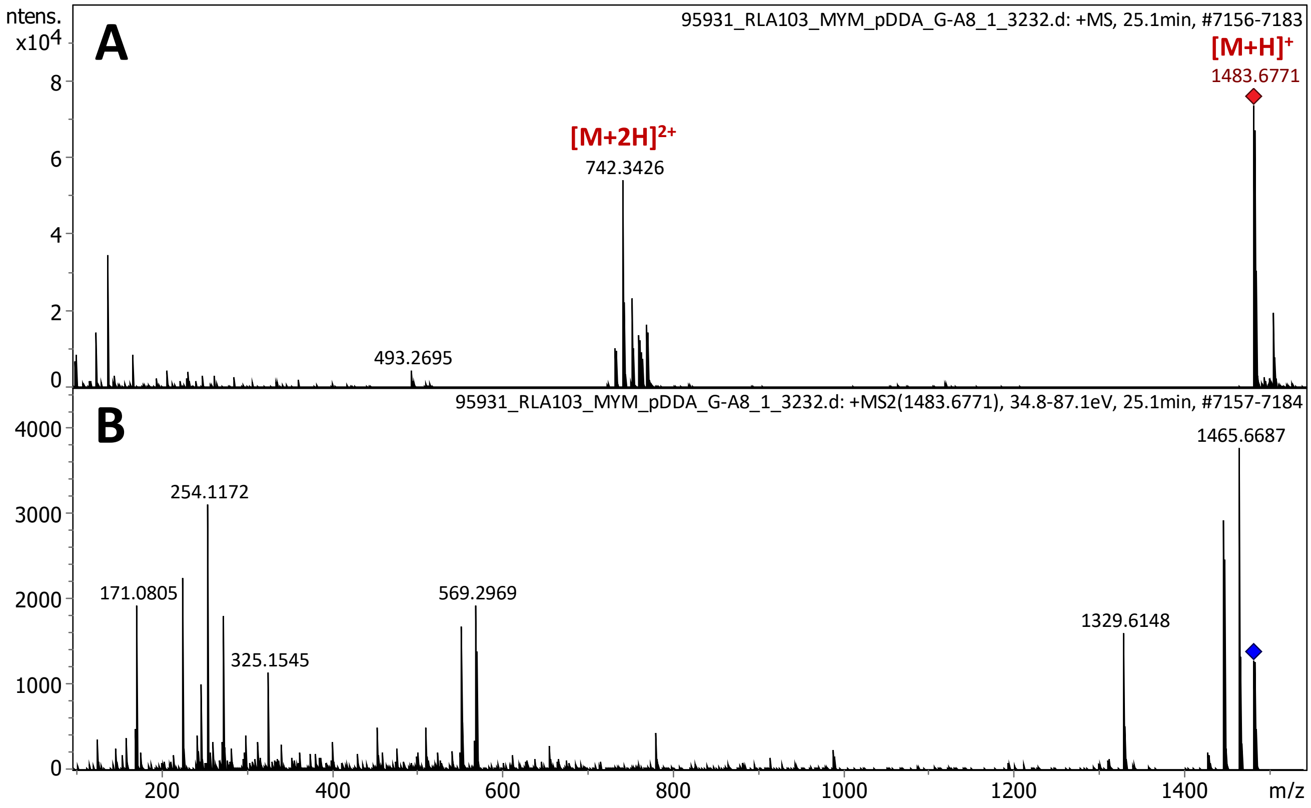

Figure S71. High resolution ESI-Qq-TOF mass spectrum of RP-1776/Skyllamycin A (**71**) in strain RLA103 grown in MYM (A) and high resolution MS/MS spectrum of its $[M+H]^+$ ion (B).

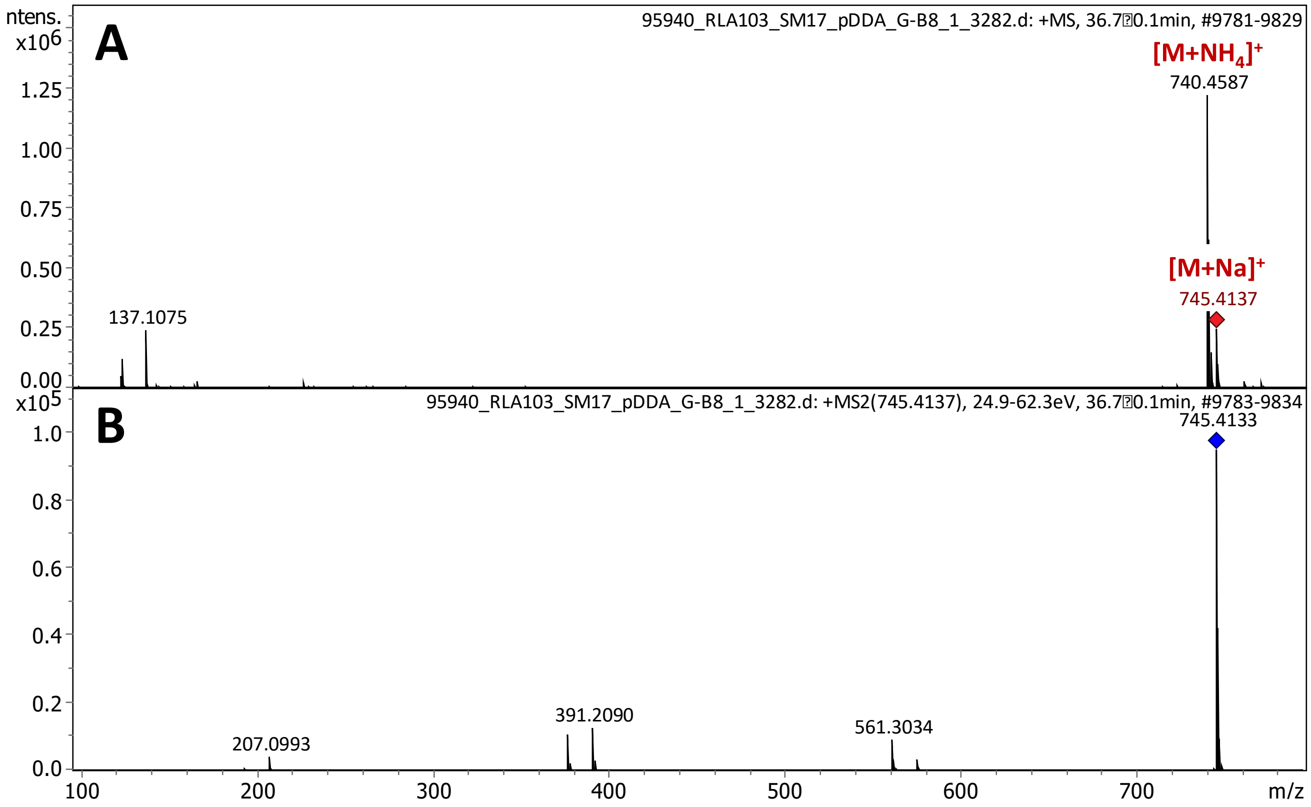

Figure S72. High resolution ESI-Qq-TOF mass spectrum of a Nonactin congener (**72**) in strain RLA103 grown in SM17 (A) and high resolution MS/MS spectrum of its $[M+Na]^+$ ion (B).

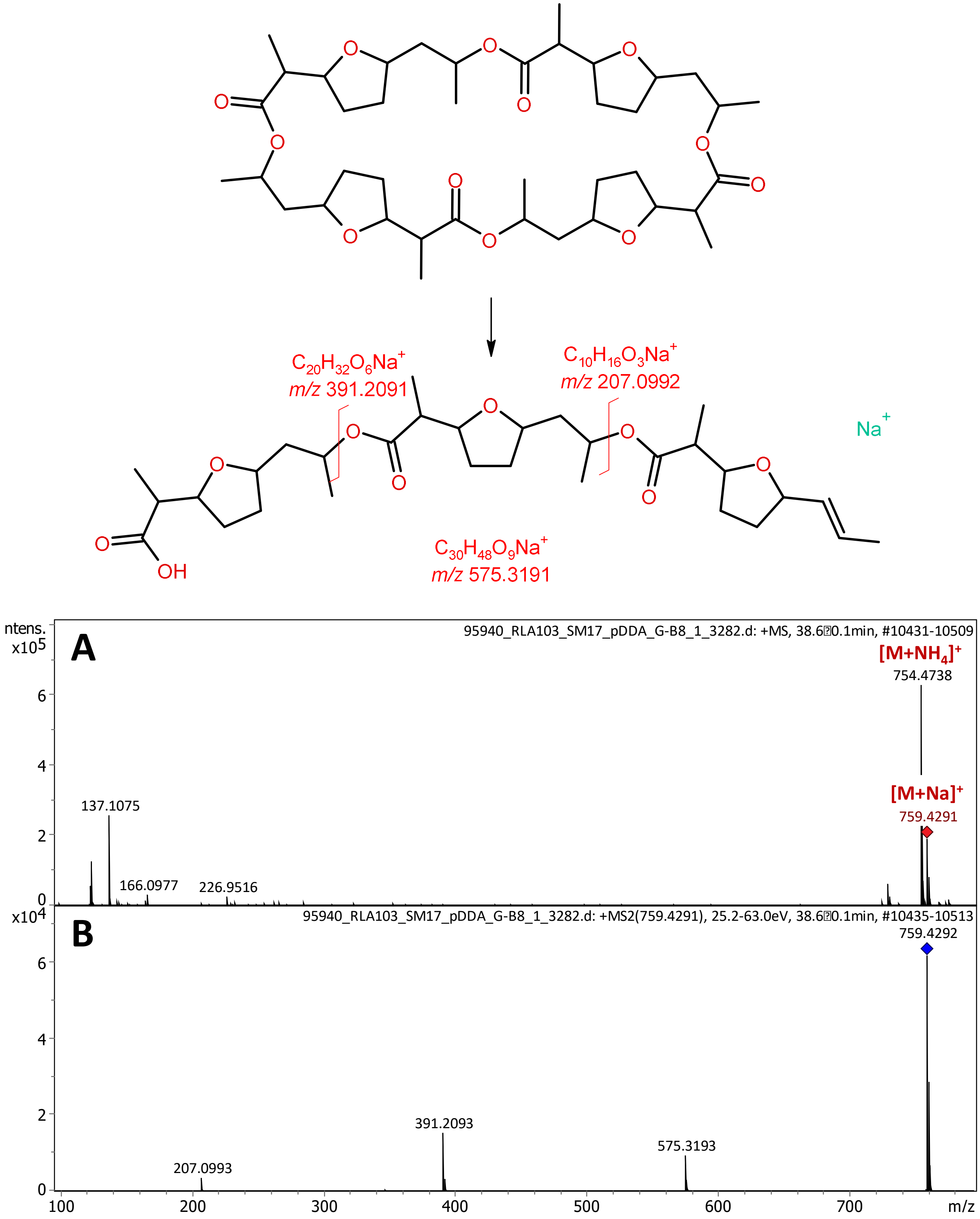

Figure S73. High resolution ESI-Qq-TOF mass spectrum of Nonactin (**73**) in strain RLA103 grown in SM17 (A) and high resolution MS/MS spectrum of its $[M+Na]^+$ ion (B).

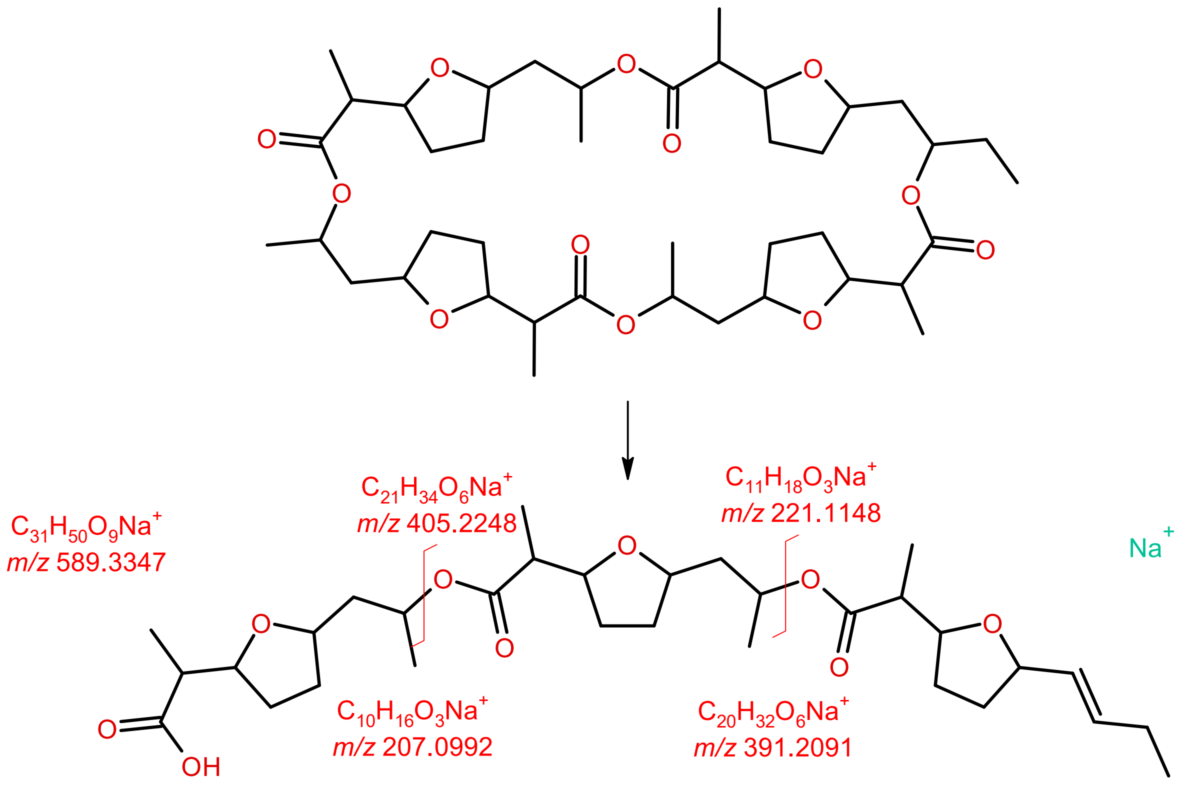

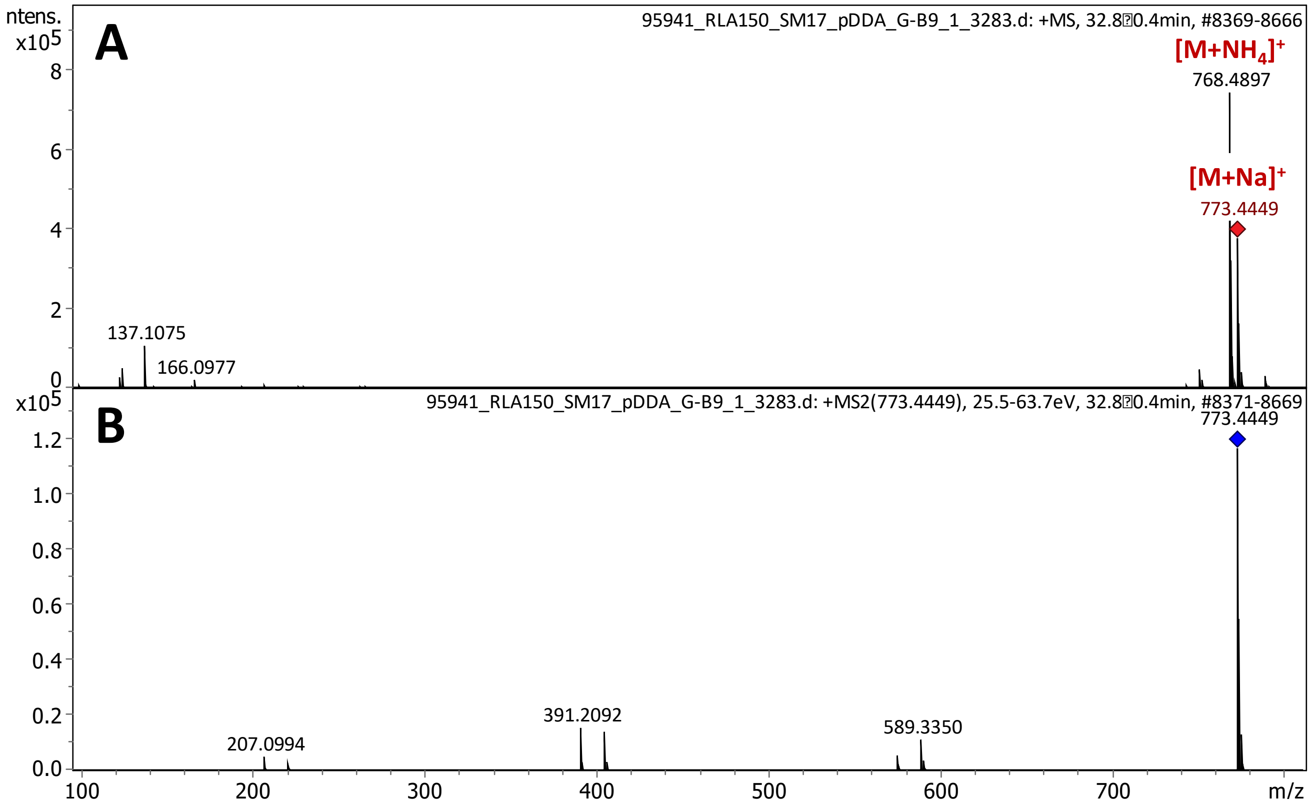

Figure S74. High resolution ESI-Qq-TOF mass spectrum of Monactin (**74**) in strain RLA103 grown in SM17 (A) and high resolution MS/MS spectrum of its $[M+Na]^+$ ion (B).

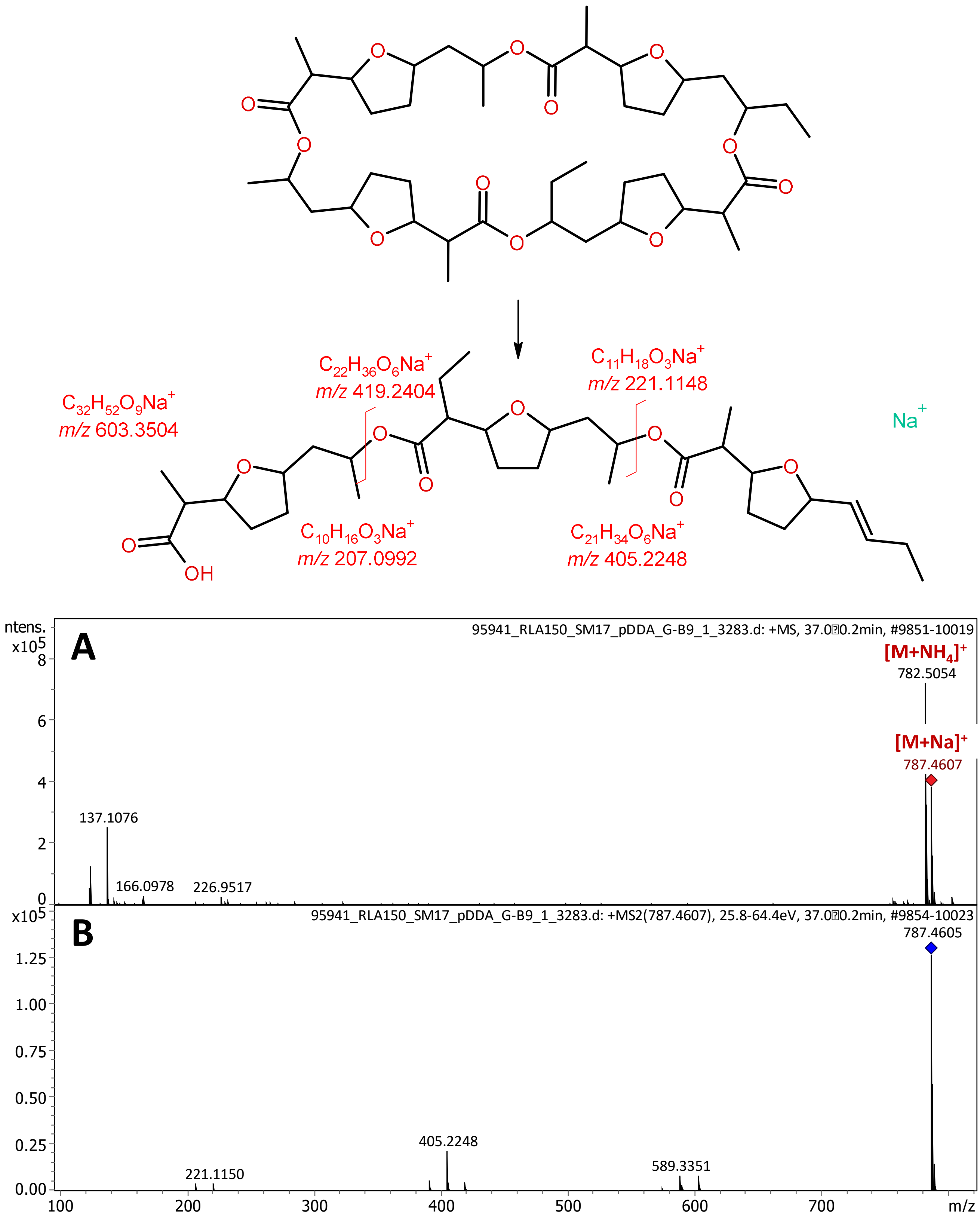

Figure S75. High resolution ESI-Qq-TOF mass spectrum of Isodinactin (**75**) in strain RLA103 grown in SM17 (A) and high resolution MS/MS spectrum of its $[M+Na]^+$ ion (B).

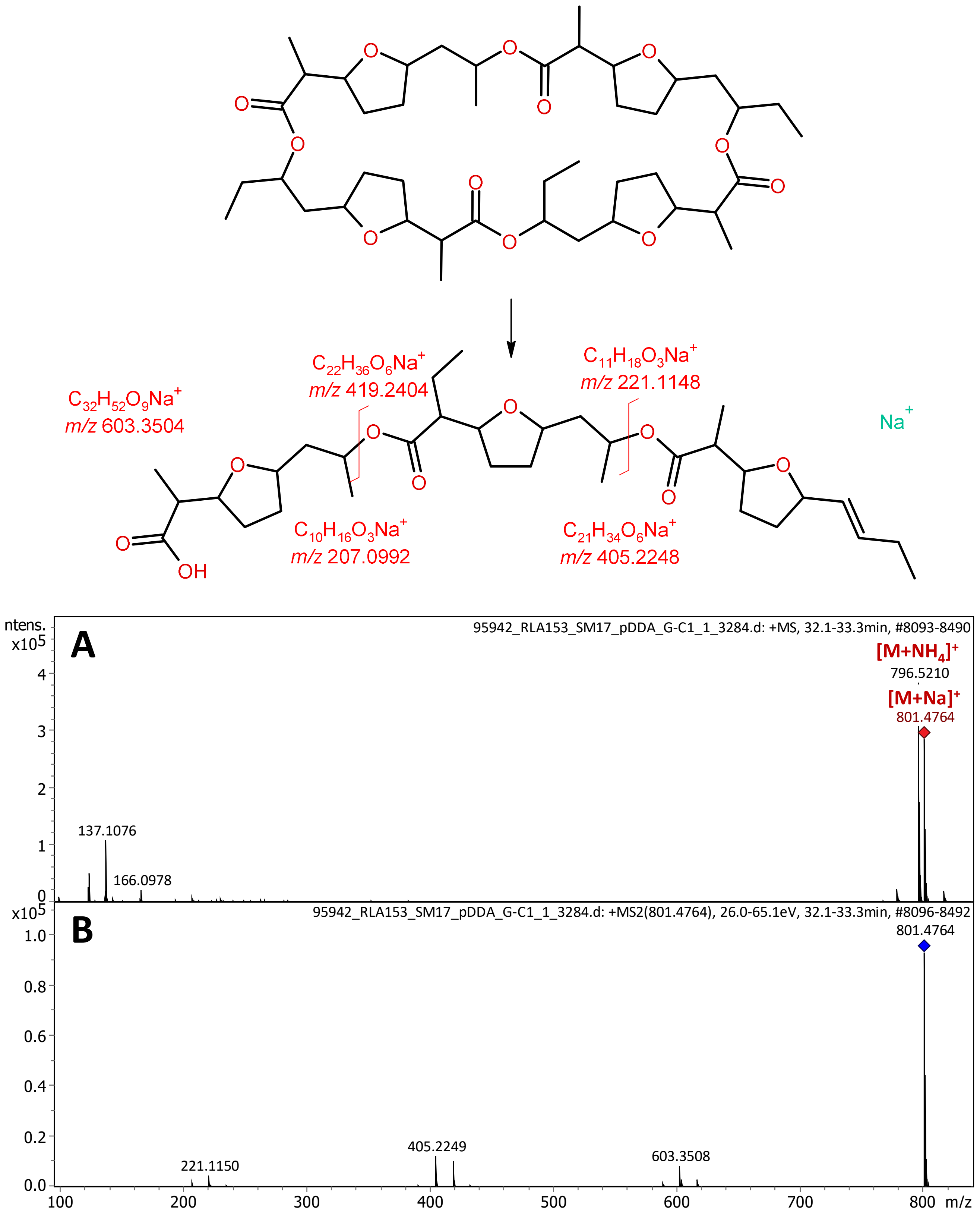

Figure S76. High resolution ESI-Qq-TOF mass spectrum of Trinactin (**76**) in strain RLA103 grown in SM17 (A) and high resolution MS/MS spectrum of its $[M+Na]^+$ ion (B).

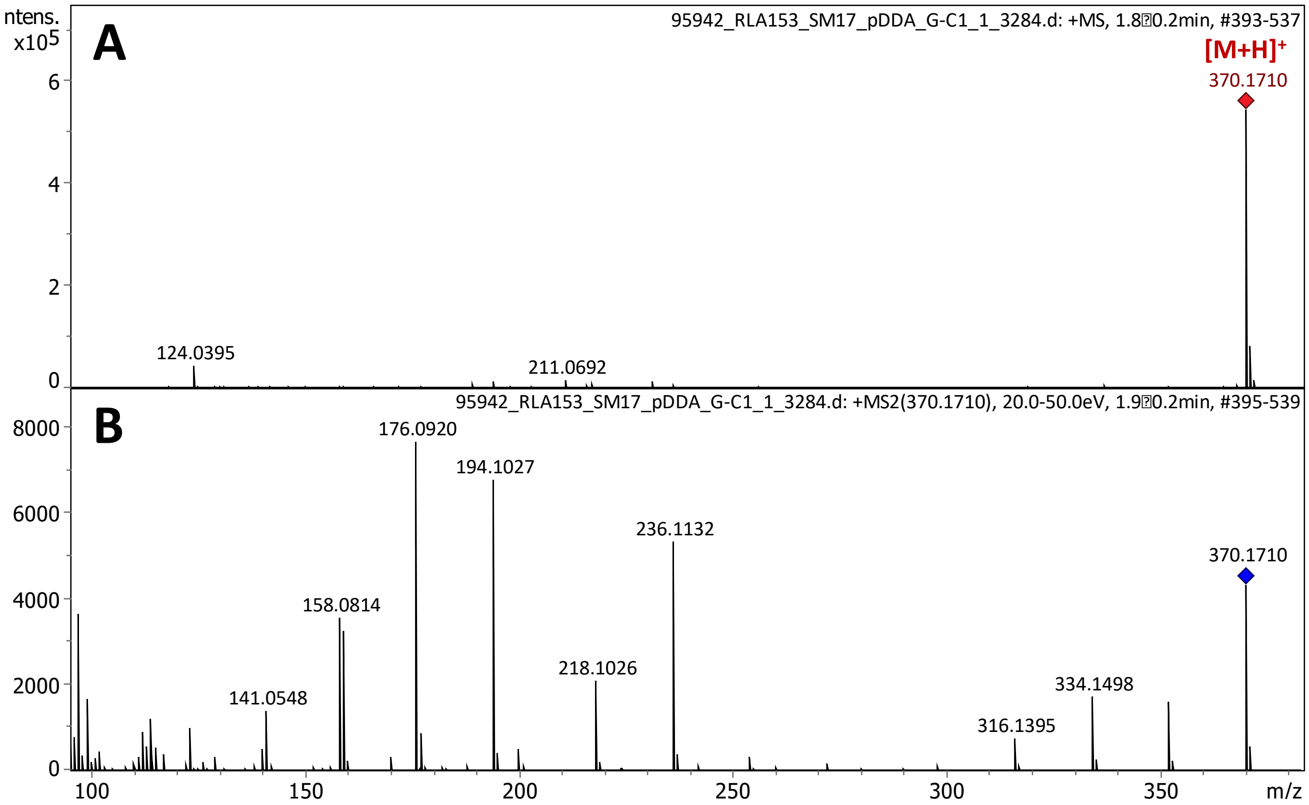

Figure S77. High resolution ESI-Qq-TOF mass spectrum of an unidentified aminodisaccharide (**77**) in strain RLA153 grown in SM17 (A) and high resolution MS/MS spectrum of its $[M+H]^+$ ion (B).

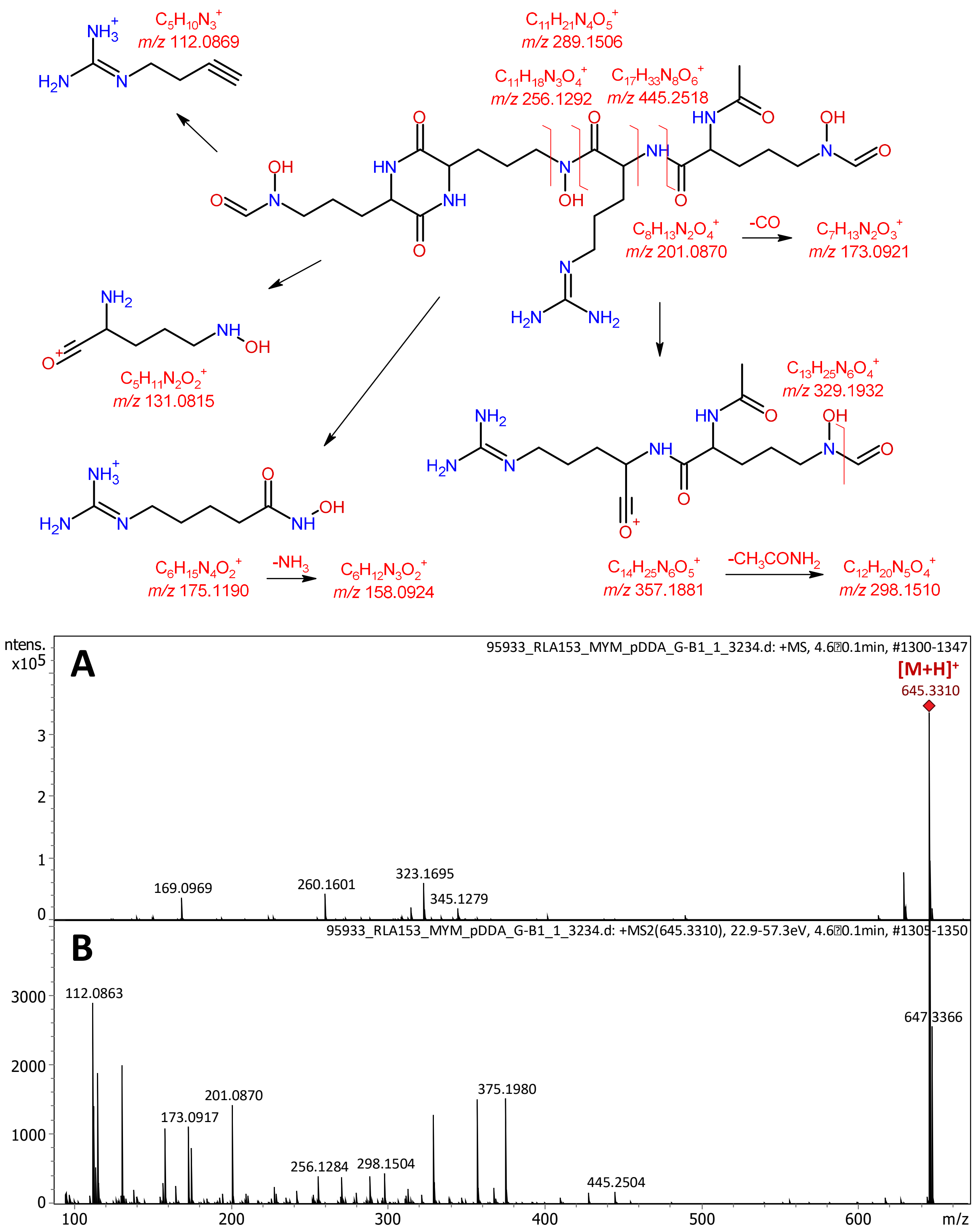

Figure S78. High resolution ESI-Qq-TOF mass spectrum of Desferri-peucechelin (**78**) in strain RLA153 grown in MYM (A) and high resolution MS/MS spectrum of its $[M+H]^+$ ion (B).

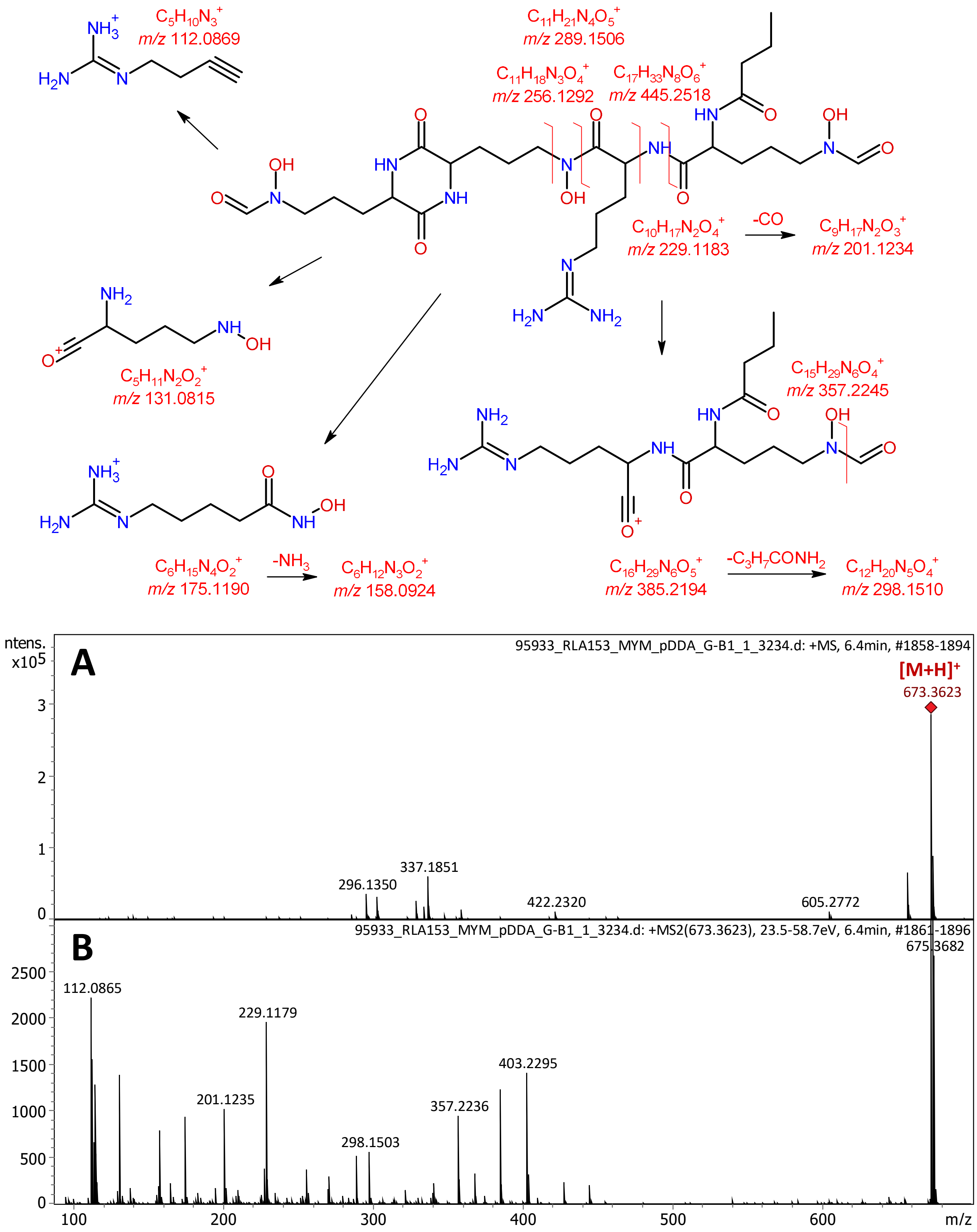

Figure S79. High resolution ESI-Qq-TOF mass spectrum of the Salinichelin C congener (**79**) in strain RLA153 grown in MYM (A) and high resolution MS/MS spectrum of its $[M+H]^+$ ion (B).

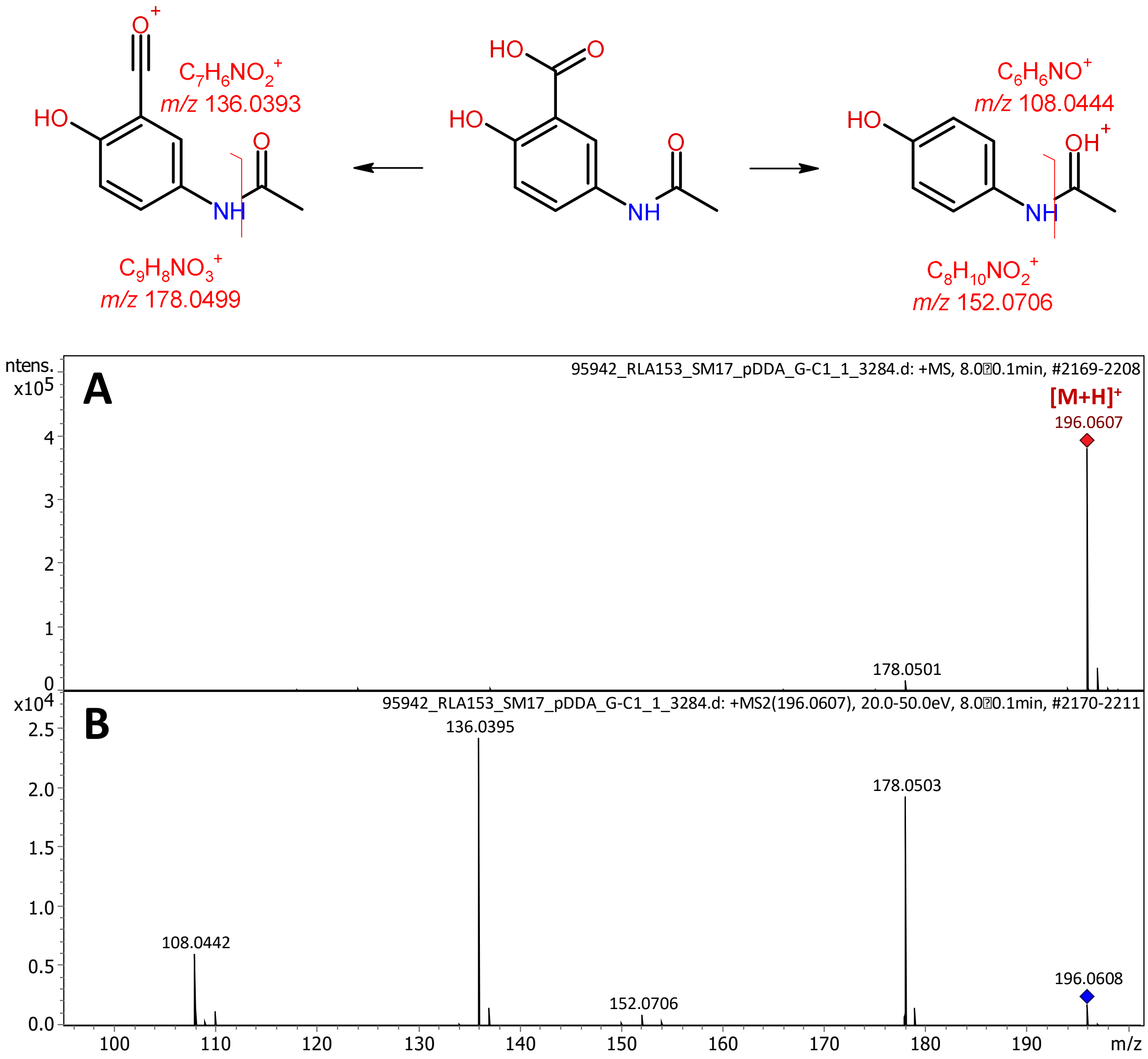

Figure S80. High resolution ESI-Qq-TOF mass spectrum of *N*-acetyl-5-aminosalicylic acid (**80**) in strain RLA153 grown in SM17 (A) and high resolution MS/MS spectrum of its $[M+H]^+$ ion (B).

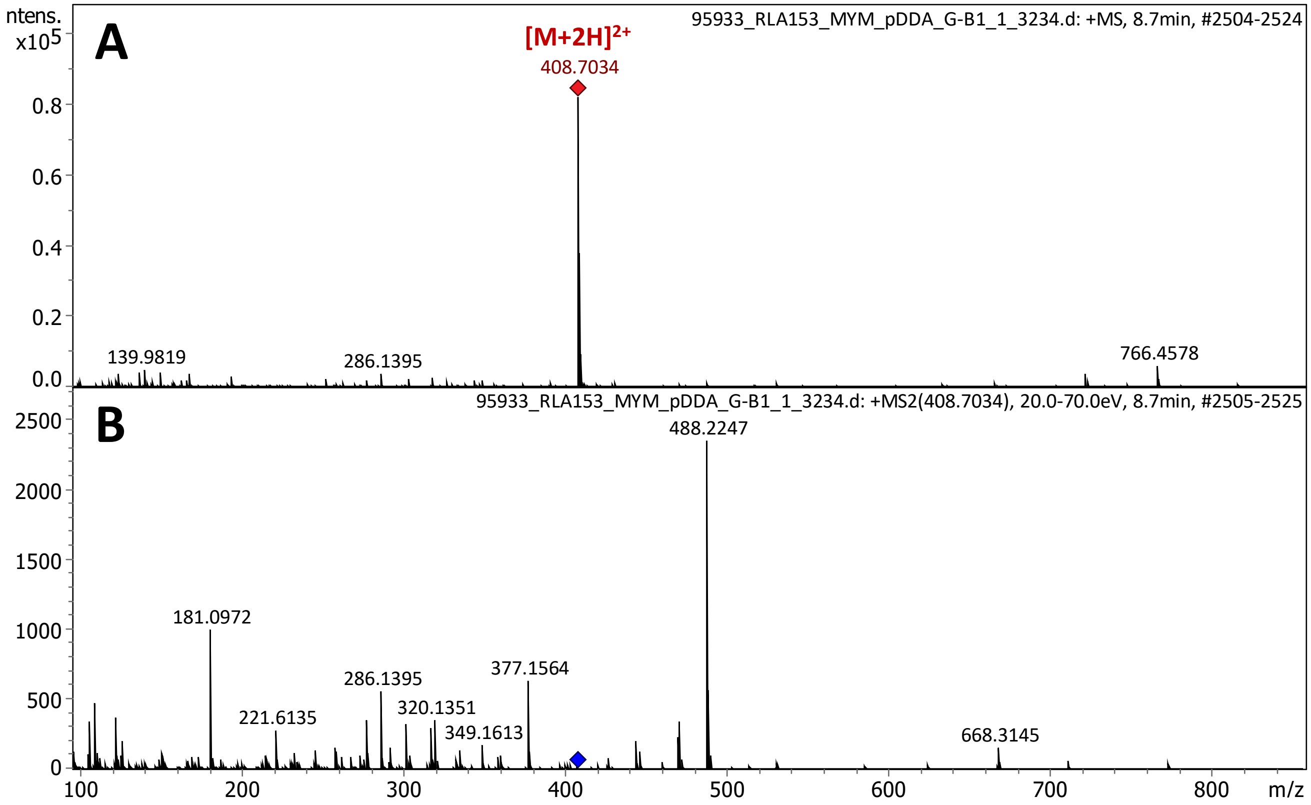

Figure S81. High resolution ESI-Qq-TOF mass spectrum of the potentially new natural product **81** in strain RLA153 grown in MYM (A) and high resolution MS/MS spectrum of its $[M+2H]^{2+}$ ion (B).

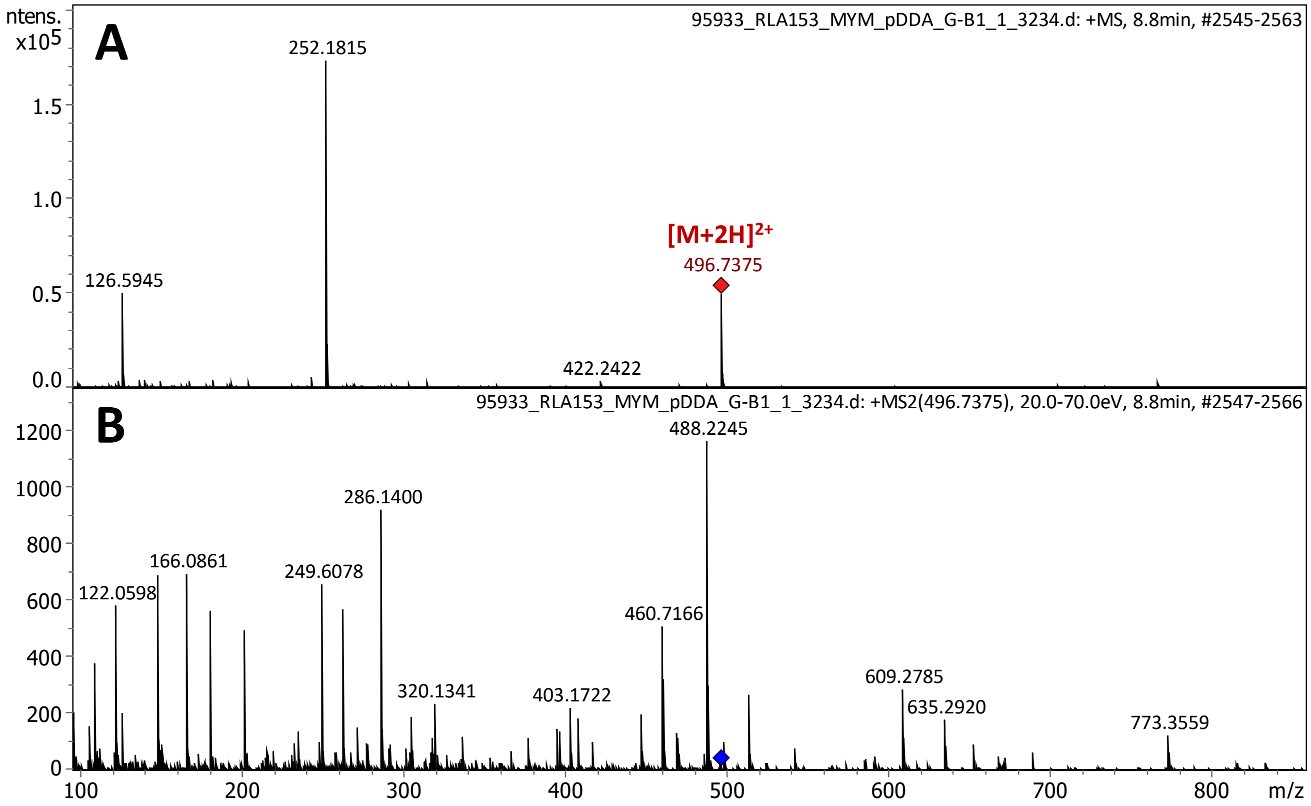

Figure S82. High resolution ESI-Qq-TOF mass spectrum of the potentially new natural product **82** in strain RLA153 grown in MYM (A) and high resolution MS/MS spectrum of its $[M+2H]^{2+}$ ion (B).

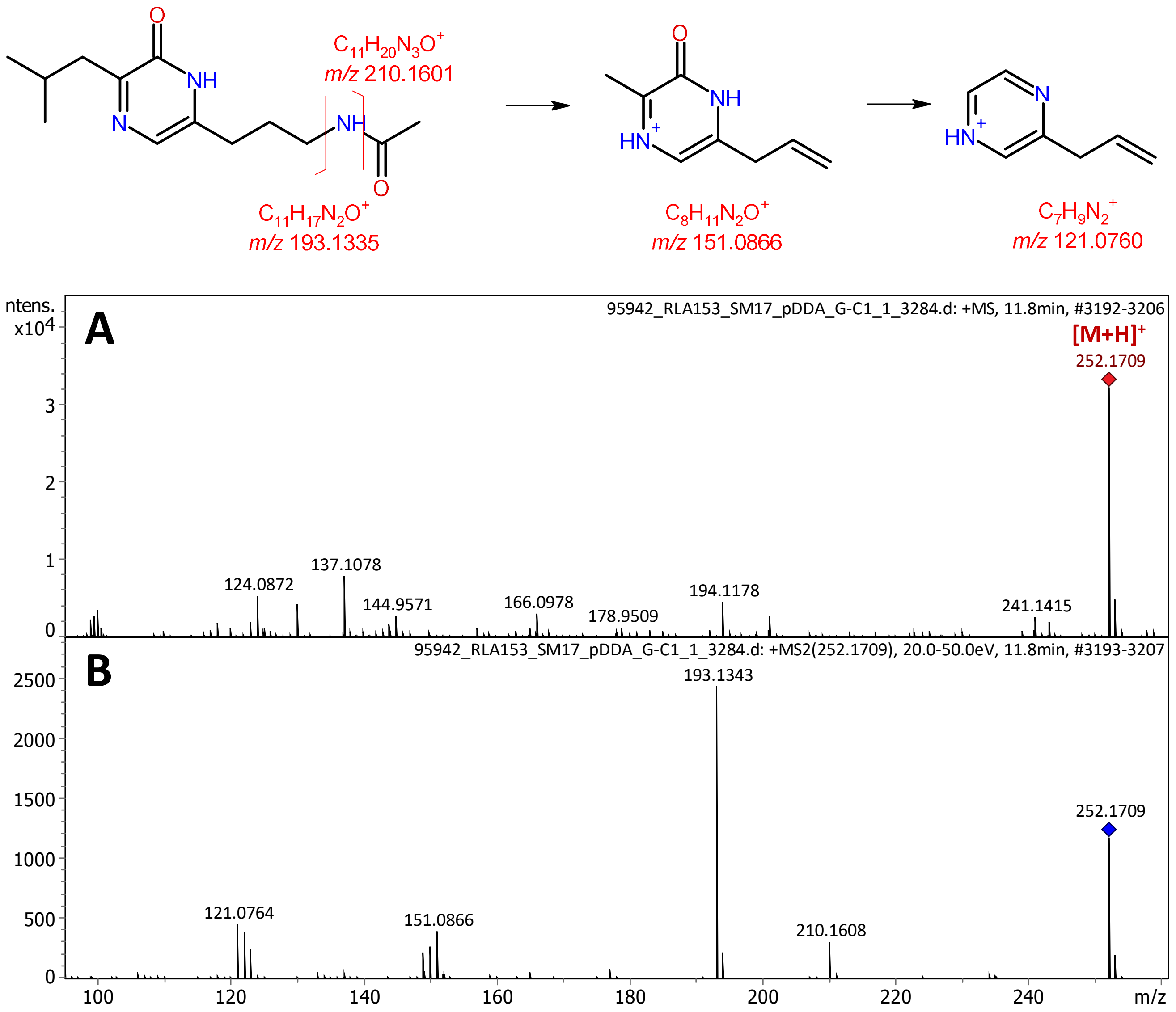

Figure S83. High resolution ESI-Qq-TOF mass spectrum of Streptopyrazinone A (**83**) in strain RLA153 grown in SM17 (A) and high resolution MS/MS spectrum of its $[M+H]^+$ ion (B).

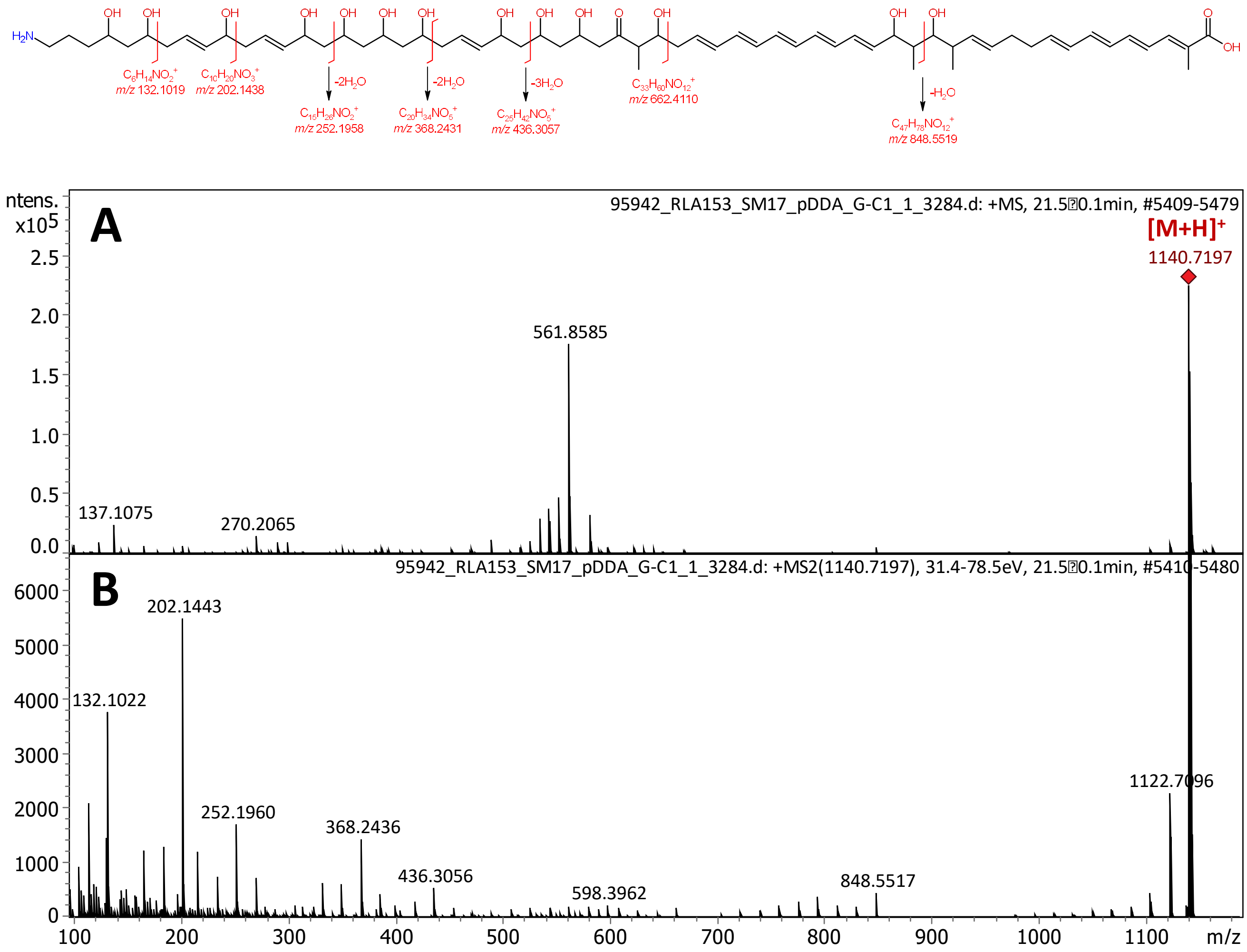

Figure S84. High resolution ESI-Qq-TOF mass spectrum of Linearmycin A (**84**) in strain RLA153 grown in SM17 (A) and high resolution MS/MS spectrum of its $[M+H]^+$ ion (B).

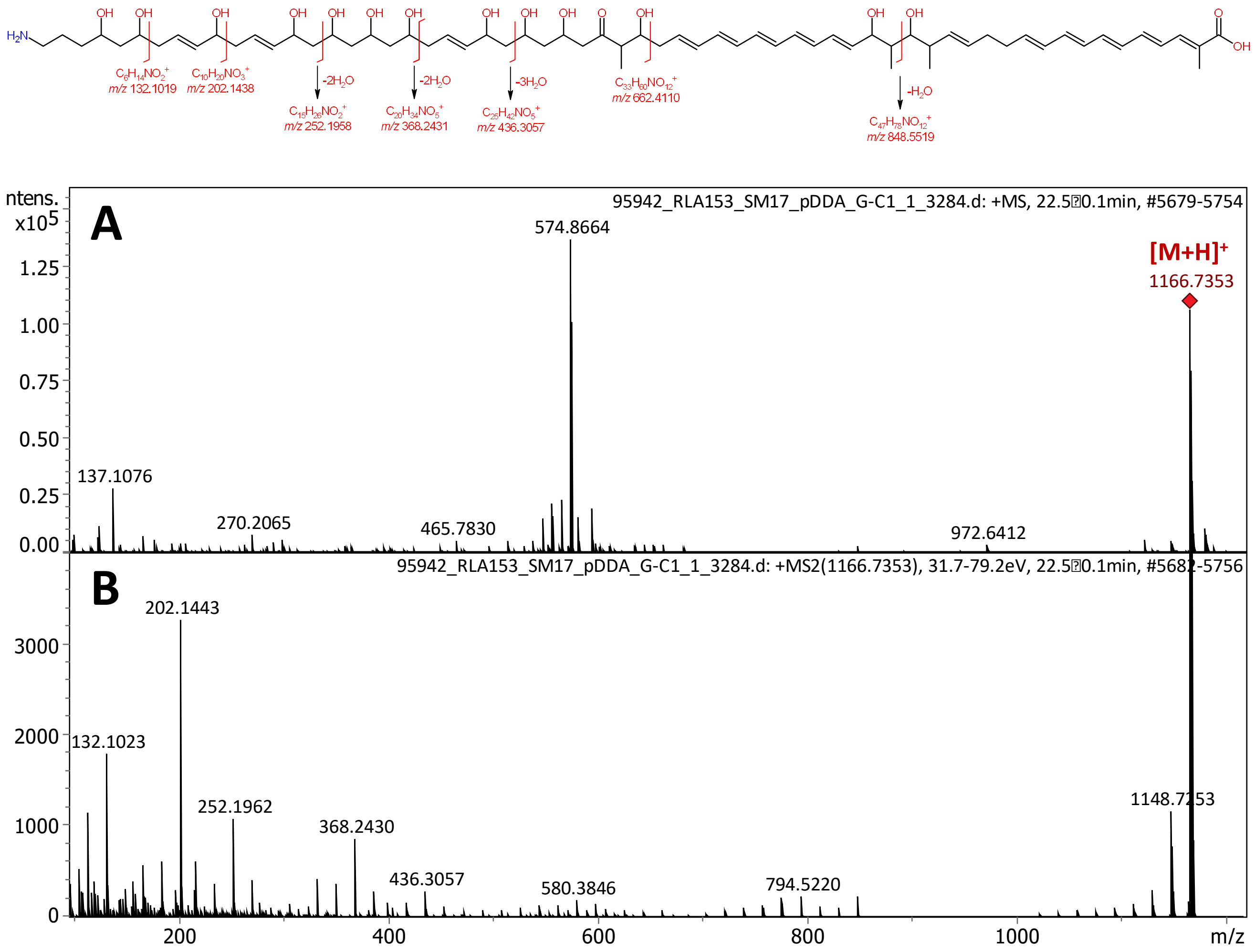

Figure S85. High resolution ESI-Qq-TOF mass spectrum of Linearmycin B (**85**) in strain RLA153 grown in SM17 (A) and high resolution MS/MS spectrum of its $[M+H]^+$ ion (B).

Figure S86. Biosynthetic gene clusters detected using antiSMASH7.0 in the genomes of *Streptomyces* spp. Isolated from the rhyzosphere of Edelweiss

RLA012

Region	No	Cluster type	Presence in another bacterium	Putative product
Region 1.1	1	Ectoine	showdomycin biosynthetic gene cluster from *Streptomyces showdoensis* (17% of genes show similarity)	ectoine (showdomycin-like)
Region 2.1	2	NRPS	diisonitrile antibiotic SF2768 biosynthetic gene cluster from *Streptomyces thioluteus* (66% of genes show similarity)	diisonitrile antibiotic SF2768
Region 2.2	3	Nucleoside	-	unknown
Region 2.3	4	2dos, T1PKS, NRPS-like hybrid cluster	hygrocin A biosynthetic gene cluster from *Streptomyces sp. LZ35* (51% of genes show similarity)	hygrocin A
Region 2.4	5	T3PKS	alkylresorcinol biosynthetic gene cluster from *Streptomyces griseus* subsp. *griseus* NBRC 13350	alkylresorcinol
Region 2.5	6	Melanin	melanin biosynthetic gene cluster from *Streptomyces coelicolor* A3(2)	melanin
Region 2.6	7	NRPS	-	NR peptide
Region 2.7	8	Lanthipeptide Class II, NRP-metallophore, NRPS	scabichelin biosynthetic gene cluster from *Streptomyces scabiei* 87.22	scabichelin
Region 2.8	9	T2PKS	spore pigment biosynthetic gene cluster from Streptomyces avermitilis (83% of genes show similarity)	spore pigment
Region 2.8	10	Indole	-	unknown
Region 2.8	11	T1PKS	-	polyketide
Region 2.9	12	Terpene	*Streptomyces olivochromogenes* strain DSM 40451 (100% of genes show similarity)	hopene
Region 2.10	13	Lanthipeptide Class IV	-	lanthipeptide
Region 2.11	14	Redox-cofactor	-	unknown
Region 2.12	15	NI-siderophore	*Streptomyces* sp. RLB1-33 chromosome (100% of genes show similarity)	siderophore
Region 2.13	16	hglE-KS (Heterocyst glycolipid synthase-like PKS)	-	unknown
Region 2.14	17	NRPS	-	NR peptide
Region 2.15	18	Terpene	geosmin biosynthetic gene cluster from *Streptomyces coelicolor* A3(2)	geosmin
Region 2.16	19	Terpene	-	terpenoid
Region 2.17	20	NI-siderophore, RiPP-like	-	unknown
Region 2.18	21	T2PKS, oligosaccharide, PKS-like	antibiotic HKI 10311129 biosynthetic gene cluster from *Streptomyces* sp. CS113 (87% of genes show similarity)	antibiotic HKI 10311129
Region 2.19	22	Terpene	albaflavenone biosynthetic gene cluster from *Streptomyces coelicolor A3(2)*	albaflavenone
Region 2.20	23	Terpene	-	terpenoid
Region 2.21	24	Lassopeptide	-	lassopeptide
Region 2.22	25	NRPS	-	NR peptide
Region 2.22	26	NRPS-like, T1PKS	-	NR peptide polyketide hybrid
Region 2.23	27	Ladderane, NRPS	*Streptomyces* sp. BK141 Ga0307717 102 (77% of genes show similarity)	modified NR peptide
Region 2.24	28	T1PKS, NRPS-like	*Streptomyces* sp. S1A1-7 chromosome (69% of genes show similarity)	unknown
Region 2.25	29	NI-siderophore	desferrioxamin B biosynthetic gene cluster from *Streptomyces coelicolor A3(2)* (83% of genes show similarity)	desferrioxamin B
Region 2.26	30	Melanin	*Streptomyces* sp. Tue6028 Scaffold13 (93% of genes show similarity)	unknown
Region 2.27	31	Linaridin, Lanthipeptide Class I	-	unknown
Region 2.28	32	Ectoine	ectoine biosynthetic gene cluster from *Streptomyces anulatus*	ectoine
Region 2.29	33	NAPAA	*Streptomyces* sp. RLB1-33 (64% of genes show similarity)	Non-alpha poly-amino acid like e-Polylysin
Region 2.30	34	NAPAA	*Streptomyces* sp. S1D4-11 chromosome (22% of genes show similarity)	Non-alpha poly-amino acid like e-Polylysin
Region 2.31	35	T3PKS	flaviolin biosynthetic gene cluster from *Streptomyces coelicolor A3(2)*	flaviolin
Region 2.32	36	T1PKS, NRPS-like, NRPS	-	unknown
Region 2.33	37	Terpene	-	terpenoid
Region 2.34	38	RiPP-like	*Streptomyces avermitilis* MA-4680 = NBRC 14893 (43% of genes show similarity)	modified peptide
Region 2.35	39	T3PKS, guanidinotides	*Streptomyces jietaisiensis* strain CGMCC 4.1859 (44% of genes show similarity)	unknown
Region 2.36	40	NAPAA	-	Non-alpha poly-amino acid like e-Polylysin
Region 2.37	41	NRPS, RiPP-like	-	peptide
Region 2.38	42	T3PKS, guanidinotides	-	unknown
Region 4.1	43	Butyrolactone	-	unknown butyrolactone
Region 6.1	44	T1PKS, NRPS-like, PKS-like	-	unknown
Region 7.1	45	T1PKS	-	polyketide

RLA016

Region	No	Cluster type	Presence in another bacterium	Putative product
Region 1.1	1	NI-siderophore	*Streptomyces olivochromogenes* strain DSM 40451 (100% of genes show similarity)	siderophore
Region 1.2	2	hglE-KS, T1PKS	*Streptomyces* sp. S1A1-7 chromosome (70% of genes show similarity)	polyketide
Region 1.3	3	RiPP-like	-	peptide
Region 1.4	4	Terpene	geosmin biosynthetic gene cluster from *Streptomyces coelicolor A3(2)*	geosmin
Region 1.5	5	NI-siderophore	*Streptomyces olivochromogenes* strain DSM 40451 PRJ... (100% of genes show similarity)	siderophore
Region 1.6	6	NRPS	-	NR peptide
Region 1.7	7	NAPAA	-	non-alpha poly-amino acid like e-Polylysin
Region 1.8	8	Terpene	albaflavenone biosynthetic gene cluster from *Streptomyces coelicolor A3(2)*	albaflavenone
Region 1.9	9	Lassopeptide	-	lassopeptide
Region 1.10	10	T2PKS, butyrolactone	*Streptomyces* sp. RLB1-33 chromosome (89% of genes show similarity)	unknown
Region 1.11	11	Lassopeptide	siamycin I biosynthetic gene cluster from *Streptomyces* sp.	siamycin I
Region 1.12	12	T1PKS, NRPS-like	*Streptomyces olivochromogenes* strain DSM 40451 (92% of genes show similarity)	unknown
Region 1.13	13	NI-siderophore	*Streptomyces olivochromogenes* strain DSM 40451 (100% of genes show similarity)	siderophore
Region 1.14	14	Melanin	*Streptomyces olivochromogenes* strain DSM 40451 (100% of genes show similarity)	melanine-like compound
Region 1.15	15	RRE-containing	-	unknown
Region 1.16	16	Ectoine	ectoine biosynthetic gene cluster from *Streptomyces anulatus*	ectoine
Region 1.17	17	NAPAA	*Streptomyces olivochromogenes* strain DSM 40451 (76% of genes show similarity)	non-alpha poly-amino acid like e-Polylysin
Region 1.18	18	T3PKS	*Streptomyces olivochromogenes* strain DSM 40451 (100% of genes show similarity)	polyketide
Region 1.19	19	NRPS, nucleoside	-	modified NR peptide
Region 1.20	20	T1PKS	*Streptomyces olivochromogenes* strain DSM 40451 (92% of genes show similarity)	polyketide
Region 1.21	21	T1PKS	*Streptomyces mirabilis* strain OK461 (82% of genes show similarity)	polyketide
Region 4.1	22	Terpene	-	terpenoid
Region 8.1	23	NRPS, NRPS-like, PKS-like, T3PKS	*Streptomyces venezuelae* strain ATCC 21018 (56% of genes show similarity)	peptide polyketide hybrid
Region 8.2	24	RiPP-like	*Streptomyces* sp. S1D4-11 chromosome (90% of genes show similarity)	unknown
Region 8.3	25	Butyrolactone	-	unknown butyrolactone
Region 8.4	26	NRPS	diisonitrile antibiotic SF2768 biosynthetic gene cluster from *Streptomyces thioluteus* (66% of genes show similarity)	NR peptide
Region 8.5	27	Lanthipeptide Class II, Terpene	*Streptomyces* sp. S1A1-7 chromosome (84% of genes show similarity)	unknown
Region 8.6	28	NAPAA	*Streptomyces olivochromogenes* strain DSM 40451 (81% of genes show similarity)	unknown
Region 8.7	29	RiPP-like	*Streptomyces* sp. RLB1-33 chromosome (75% of genes show similarity)	peptide
Region 8.8	30	T1PKS, NRPS-like	*Streptomyces* sp. RLB1-8 chromosome (93% of genes show similarity)	peptide polyketide hybrid
Region 10.1	31	Thioamitides	-	unknown
Region 11.1	32	Redox-cofactor	-	unknown
Region 11.2	33	Terpene	hopene biosynthetic gene cluster from *Streptomyces coelicolor A3(2)* (92% of genes show similarity)	hopene
Region 11.3	34	T1PKS, indole, other, T2PKS	*Streptomyces olivochromogenes* strain DSM 40451 (91% of genes show similarity)	unknown
Region 11.4	35	NRP-metallophore, NRPS	scabichelin biosynthetic gene cluster from *Streptomyces scabiei 87.22*	scabichelin
Region 11.5	36	RiPP-like	*Streptomyces olivochromogenes* strain DSM 40451 (100% of genes show similarity)	peptide
Region 13.1	37	T3PKS	alkylresorcinol biosynthetic gene cluster from *Streptomyces griseus subsp. griseus NBRC 13350*	alkylresorcinol

RLA039

Region	No	Cluster type	Presence in another bacterium	Putative product
Region 1.1	1	NRPS-like, T1PKS	-	unknown
Region 1.2	2	Lanthipeptide Class III	-	lanthipeptide
Region 1.3	3	Butyrolactone	*Streptomyces* sp. BK308 Ga0307715 120 (60% of genes show similarity)	unknown butyrolactone
Region 1.4	4	T1PKS, T3PKS	filipin biosynthetic gene cluster from *Streptomyces avermitilis MA-4680 = NBRC 14893*	filipin
Region 1.5	5	Melanin	melanin biosynthetic gene cluster from *Streptomyces avermitilis* (71% of genes show similarity)	melanin
Region 1.6	6	Butyrolactone	-	unknown butyrolactone
Region 1.7	7	NRPS-like, betalactone, NRPS	-	unknown
Region 1.8	8	NAPAA	-	non-alpha poly-amino acid like e-Polylysin
Region 1.9	9	NRPS, T1PKS	-	unknown
Region 1.10	10	NAPAA	ε-Poly-L-lysine biosynthetic gene cluster from *Epichloe festucae*	ε-Poly-L-lysine
Region 1.11	11	Ectoine	ectoine biosynthetic gene cluster from *Streptomyces anulatus*	ectoine
Region 1.12	12	Melanin	*Streptomyces* sp. QMT-28 chromosome (92% of genes show similarity)	unknown
Region 1.13	13	NI-Siderophore	desferrioxamin B biosynthetic gene cluster from *Streptomyces coelicolor* A3(2) (66% of genes show similarity)	desferrioxamin B
Region 1.14	14	Terpene	albaflavenone biosynthetic gene cluster from *Streptomyces coelicolor* A3(2)	albaflavenone
Region 1.15	15	NI-Siderophore	Streptomyces turgidiscabies strain T45, whole genome (87% of genes show similarity)	siderophore
Region 1.16	16	Arylpolyene,Ladderane	-	unknown
Region 1.17	17	Terpene	*Streptomyces* sp. S1A1-7 chromosome (80% of genes show similarity)	terpenoid
Region 1.18	18	RiPP-like	-	unknown
Region 1.19	19	NI-siderophore	-	siderophore
Region 1.20	20	Terpene	hopene biosynthetic gene cluster from *Streptomyces coelicolor A3(2)*, (92% of genes show similarity)	hopene
Region 1.21	21	Betalactone	-	unknown
Region 1.22	22	T2PKS, RRE-containing, Thiopeptide	spore pigment biosynthetic gene cluster from *Streptomyces avermitilis* (83% of genes show similarity)	spore pigment
Region 1.22	23	T1PKS, NRPS, Nucleoside	-	unknown
Region 1.23	24	NRPS	diisonitrile antibiotic SF2768 biosynthetic gene cluster from *Streptomyces thioluteus* (72% of genes show similarity)	NR peptide
Region 1.24	25	RiPP-like	-	unknown
Region 1.25	26	Redox-cofactor,Terpene	-	unknown
Region 1.26	27	NRPS,NRPS-like,T1PKS	-	unknown
Region 1.27	28	Butyrolactone	-	unknown butyrolactone
Region 1.28	29	Butyrolactone	-	unknown butyrolactone
Region 2.1	30	T1PKS,T2PKS,T3PKS,Butyrolactone	-	unknown
Region 3.1	31	NRPS-like,T1PKS	-	unknown

RLA041

Region	No	Cluster type	Presence in another bacterium	Putative product
Region 1.1	1	RiPP-like	-	modified peptide
Region 1.2	2	Terpene	*Streptomyces scabiei* 87.22, complete genome (100% of genes show similarity)	terpenoid
Region 1.3	3	RiPP-like	*Streptomyces* sp. 136MFCol5.1 (100% of genes show similarity)	modified peptide
Region 1.4	4	Arylpolyene, Ladderane	-	unknown
Region 1.5	5	NI-siderophore	*Streptomyces* sp. ok210 (100% of genes show similarity)	siderophore
Region 1.6	6	Terpene	*Streptomyces* sp. 136MFCol5.1 (100% of genes show similarity)	terpenoid
Region 1.7	7	NRPS-like,T1PKS	*Streptomyces* sp. 136MFCol5.1 (94% of genes show similarity)	unknown
Region 1.8	8	T3PKS	naringenin biosynthetic gene cluster from *Streptomyces clavuligerus* ATCC 27064	naringenin
Region 1.9	9	NI-siderophore	desferrioxamin B biosynthetic gene cluster from *Streptomyces griseus* subsp. *griseus* NBRC 13350	desferrioxamin B
				unknown
Region 1.10	10	Butyrolactone	Streptomyces sp. 136MFCol5.1 (100% of genes show similarity)	butyrolactone
Region 1.11	11	Ectoine	ectoine biosynthetic gene cluster from Streptomyces anulatus	ectoine
Region 1.12	12	Terpene	*Streptomyces* sp. 136MFCol5.1 (87% of genes show similarity)	terpenoid
Region 1.13	13	T3PKS	alkylresorcinol biosynthetic gene cluster from *Streptomyces griseus* subsp. *griseus* NBRC 13350	alkylresorcinol
Region 1.13	14	RiPP-like	-	modified peptide
Region 1.14	15	NAPAA,NRPS	-	unknown
Region 1.15	16	T2PKS, Terpene	spore pigment biosynthetic gene cluster from *Streptomyces avermitilis* (83% of genes show similarity)	spore pigment
Region 1.16	17	Terpene	-	unknown

RLA046

Region	No	Cluster type	Presence in another bacterium	Putative product
Region 1.1	1	Arylpolyene, Ladderane	*Streptomyces* sp. 136MFCol5.1 (64% of genes show similarity)	unknown
Region 1.2	2	RiPP-like	*Streptomyces* sp. 136MFCol5.1 (100% of genes show similarity)	modified peptide
Region 3.1	3	RiPP-like	*Streptomyces* sp. ok210 (85% of genes show similarity)	modified peptide
Region 3.2	4	Terpene	*Streptomyces scabiei* 87.22, complete genome (100% of genes show similarity)	terpenoid
Region 3.3	5	NI-siderophore	*Streptomyces* sp. ok210 (100% of genes show similarity)	siderophore
Region 3.4	6	Terpene	*Streptomyces* sp. 136MFCol5.1 (100% of genes show similarity)	terpenoid
Region 3.5	7	NRPS-like,T1PKS	*Streptomyces* sp. ok210 (92% of genes show similarity)	unknown
Region 3.6	8	RiPP-like	-	unknown
Region 3.7	9	T3PKS	naringenin biosynthetic gene cluster from *Streptomyces clavuligerus ATCC 27064*	naringenin
Region 3.8	10	NI-siderophore	desferrioxamin B biosynthetic gene cluster from *Streptomyces griseus subsp. griseus NBRC 13350*	desferrioxamin B
Region 3.9	11	Butyrolactone	*Streptomyces* sp. 136MFCol5.1 (100% of genes show similarity)	unknown butyrolactone
Region 3.10	12	Ectoine	ectoine biosynthetic gene cluster from *Streptomyces anulatus*	ectoine
Region 3.11	13	Terpene	*Streptomyces* sp. 136MFCol5.1 (100% of genes show similarity)	terpenoid
Region 3.12	14	T3PKS	alkylresorcinol biosynthetic gene cluster from *Streptomyces griseus subsp. griseus NBRC 13350*	alkylresorcinol
Region 3.12	15	RiPP-like	-	modified peptide
Region 3.13	16	NAPAA,NRPS	-	unknown
Region 3.14	17	T2PKS, Terpene	spore pigment biosynthetic gene cluster from Streptomyces avermitilis (83% of genes show similarity)	spore pigment
Region 3.15	18	Terpene	-	terpenoid

RLA051

Region	No	Cluster type	Presence in another bacterium	Putative product
Region 1.1	1	NRPS,Terpene	-	unknown
Region 1.2	2	T2PKS, Terpene	spore pigment biosynthetic gene cluster from *Streptomyces avermitilis (83% of genes show similarity)*	spore pigment
Region 1.3	3	NRP-metallophore,NRPS,T3PKS	coelichelin biosynthetic gene cluster from *Streptomyces coelicolor A3(2)*	coelichelin
Region 1.4	4	NRP-metallophore,NRPS	-	unknown
Region 1.5	5	Terpene	-	unknown
Region 1.6	6	RiPP-like	*Streptomyces* sp. QL37 (75% of genes show similarity)	modified peptide
Region 1.7	7	NRPS-like, Terpene	*Streptomyces* sp. ADI92-24 scaffold004 (93% of genes show similarity)	unknown
Region 1.8	8	NRPS,T1PKS	-	unknown
Region 1.9	9	Ectoine	ectoine biosynthetic gene cluster from *Streptomyces anulatus*	ectoine
Region 1.10	10	Butyrolactone	-	unknown butyrolactone
Region 1.11	11	Other	-	unknown
Region 1.12	12	Terpene	-	terpenoid
Region 1.13	13	T1PKS, T2PKS	-	polyketide
Region 1.14	14	NRPS,NRPS-like,T1PKS	-	unknown
Region 1.15	15	NI-siderophore	*Streptomyces* sp. CB02058 scaffold3 (100% of genes show similarity)	siderophore
Region 1.16	16	T2PKS, Indole	-	unknown
Region 1.17	17	RiPP-like	*Streptomyces* sp. 136MFCol5.1 (100% of genes show similarity)	modified peptide
Region 1.18	18	NRPS, Lanthipeptide-class-II	-	unknown
Region 1.19	19	Terpene	hopene biosynthetic gene cluster from *Streptomyces coelicolor A3(2)* (76% of genes show similarity)	hopene
Region 1.20	20	T1PKS, Terpene	-	unknown
Region 1.21	21	RiPP-like	-	modified peptide
Region 1.22	22	Other	-	unknown
Region 3.1	23	Thioamitides	-	unknown

RLA063

Region	No	Cluster type	Presence in another bacterium	Putative product
Region 1.1	1	Terpene	isorenieratene biosynthetic gene cluster from *Streptomyces griseus* subsp. *griseus* NBRC 13350	isorenieratene
Region 1.2	2	Terpene	-	terpenoid
Region 1.3	3	NRPS	-	NR peptide
Region 1.4	4	Other	-	unknown
Region 1.5	5	Ectoine	ectoine biosynthetic gene cluster from *Streptomyces anulatus*	ectoine
Region 1.6	6	Lanthipeptide class III	SapB biosynthetic gene cluster from *Streptomyces coelicolor A3(2)*	SapB
Region 1.7	7	NRP-metallophore, NRPS	coelichelin biosynthetic gene cluster from *Streptomyces coelicolor A3(2*)	coelichelin
Region 1.8	8	NRPS-like, T1PKS	-	unknown
Region 1.9	9	CDPS	*Streptomyces lavendulae* subsp. *lavendulae* (90% of genes show similarity)	cyclodipeptide
Region 1.10	10	NI-siderophore	*Streptomyces lavendulae* subsp. *lavendulae* (100% of genes show similarity)	siderophore
Region 1.11	11	RiPP-like	*Streptomyces subrutilus* strain ATCC 27467 (100% of genes show similarity)	peptide
Region 1.12	12	NRPS, terpene	-	unknown
Region 1.13	13	Terpene	*Streptomyces lavendulae* subsp. *lavendulae* (100% of genes show similarity)	terpenoid
Region 1.14	14	T1PKS, hglE-KS	-	polyketide
Region 1.15	15	NRP-metallophore,NRPS,NRPS-like,T1PKS	-	unknown
Region 1.16	16	Melanin	melanin biosynthetic gene cluster from *Streptomyces coelicolor* A3(2)	melanin
Region 1.17	17	NI-siderophore	*Streptomyces* sp. ADI91-18 scaffold003 (72% of genes show similarity)	siderophore
Region 1.18	18	T3PKS	alkylresorcinol biosynthetic gene cluster from Streptomyces griseus subsp. griseus NBRC 13350	alkylresorcinol
Region 1.18	19	LAP,RRE-containing,thiopeptide	-	unknown
Region 1.19	20	NRPS-like,T1PKS	-	unknown
Region 1.20	21	NAPAA	ε-Poly-L-lysine biosynthetic gene cluster from *Epichloe festucae*	ε-Poly-L-lysine
Region 1.21	22	Terpene	-	terpenoid
Region 1.22	23	T2PKS	spore pigment biosynthetic gene cluster from *Streptomyces avermitilis*	spore pigment (polyketide)
Region 1.22	24	NAPAA	-	unknown
Region 1.23	25	Terpene	avermitilol biosynthetic gene cluster from *Streptomyces avermitilis MA-4680 = NBRC 14893*	avermitilol
Region 1.24	26	Lanthipeptide class IV	venezuelin biosynthetic gene cluster from *Streptomyces venezuelae ATCC 10712*	venezuelin
Region 1.25	27	RiPP-like	-	unknown
Region 2.1	28	NRPS	-	NR peptide
Region 2.2	29	NRPS,NRPS-like,arylpolyene,ladderane,lassopeptide	-	unknown
Region 2.3	30	NRPS, NRPS-like	antipain biosynthetic gene cluster from Streptomyces sp.	antipain
Region 2.3	31	PKS-like,butyrolactone,terpene	-	unknown
Region 2.4	32	LAP,T2PKS,butyrolactone,thiopeptide	-	unknown
Region 2.5	33	LAP	*Streptomyces subrutilus* strain ATCC 27467 (92% of genes show similarity)	unknown
Region 2.6	34	T1PKS,butyrolactone	coelimycin P1 biosynthetic gene cluster from *Streptomyces coelicolor A3(2) (79% of genes show similarity)*	coelimycin P1

RLA102

Region	No	Cluster type	Presence in another bacterium	Putative product
Region 1.1	1	Terpene	-	terpenoid
Region 1.2	2	T1PKS	-	polyketide
Region 1.3	3	NRPS, Thioamitides	-	unknown
Region 2.1	4	T1PKS	-	polyketide
Region 3.1	5	T1PKS	-	polyketide
Region 5.1	6	Terpene	isorenieratene biosynthetic gene cluster from *Streptomyces griseus* subsp. *griseus NBRC 13350*	isorenieratene
Region 5.2	7	NI-siderophore	-	siderophore
Region 5.3	8	Terpene	-	terpenoid
Region 5.4	9	RiPP-like, Terpene	-	unknown
Region 5.5	10	Terpene	*Streptomyces* sp. QL37 (100% of genes show similarity)	terpenoid
Region 5.6	11	NAPAA	-	unknown
Region 5.7	12	RiPP-like	*Streptomyces* sp. S501 chromosome, complete genome (100% of genes show similarity)	modified peptide
Region 5.8	13	NI-Siderophore	*Streptomyces* sp. LaPpAH-165 B070DRAFT (100% of genes show similarity)	siderophore
Region 5.9	14	RiPP-like	-	modified peptide
Region 5.10	15	Terpene	-	terpenoid
Region 5.11	16	PKS-like, RiPP-like, T2PKS, Betalactone	-	unknown
				unknown
Region 5.12	17	Butyrolactone	-	butyrolactone
Region 5.13	18	Betalactone	*Streptomyces microflavus* strain NA06532 chromo... (78% of genes show similarity)	unknown
Region 5.14	19	NRPS	Streptomyces nitrosporeus strain ATCC 12769 (84% of genes show similarity)	peptide
Region 5.15	20	Ectoine	ectoine biosynthetic gene cluster from *Streptomyces anulatus*	ectoine
Region 5.16	21	Terpene	*Streptomyces* sp. WY228 chromosome (100% of genes show similarity)	terpenoid
Region 5.17	22	T1PKS	-	polyketide
Region 5.18	23	Terpene	-	terpenoid
Region 5.19	24	RiPP-like	*Streptomyces pratensis* strain S10 chromosome (100% of genes show similarity)	modified peptide
Region 5.20	25	NRP-metallophore,NRPS,T3PKS, Amglyccycl,Arylpolyene		
Region 5.21	26	Melanin	melanin biosynthetic gene cluster from *Streptomyces griseus* subsp. *griseus NBRC 13350*	melanin
Region 5.22	27	T2PKS, Terpene	spore pigment biosynthetic gene cluster from *Streptomyces avermitilis* (83% of genes show similarity)	spore pigment
Region 5.23	28	β-lactam	-	unknown

RLA103

Region	No	Cluster type	Presence in another bacterium	Putative product
Region 1.1	1	Terpene	isorenieratene biosynthetic gene cluster from *Streptomyces griseus* subsp. *griseus NBRC 13350*	isorenieratene
Region 1.1	2	NRPS	-	NR peptide
Region 1.2	3	NRPS,T1PKS	*Streptomyces anulatus* strain ATCC 11523 (100% of genes show similarity)	NR peptide polyketide hybrid
Region 1.3	4	CDPS, NRPS-like, Lanthipeptide-class-IV	-	unknown
Region 1.3	5	Trans-AT PKS	cycloheximide biosynthetic gene cluster from *Streptomyces* sp. *YIM 56141*	cycloheximide
Region 1.4	6	T3PKS	alkylresorcinol biosynthetic gene cluster from *Streptomyces griseus* subsp. *griseus NBRC 13350*	alkylresorcinol
Region 1.5	7	Melanin	melanin biosynthetic gene cluster from *Streptomyces griseus* subsp. *griseus NBRC 13350*	melanin
Region 1.6	8	RiPP-like	streptamidine biosynthetic gene cluster from *Streptomyces albidoflavus J1074* (66% of genes show similarity)	streptamidine
Region 1.7	9	T1PKS	*Streptomyces* sp. TSRI0261 scaffold4 (88% of genes show similarity)	polyketide
Region 1.8	10	RiPP-like	-	modified peptide
Region 1.9	11	NRPS,T1PKS	clifednamide A biosynthetic gene cluster from *Streptomyces* sp. *JV178*	clifednamide A
Region 1.10	12	Linaridin	*Streptomyces anulatus* strain ATCC 11523 (68% of genes show similarity)	unknown
Region 1.11	13	Terpene	*Streptomyces griseus* subsp. *griseus* NBRC 13350 (100% of genes show similarity)	terpenoid
Region 1.12	14	Terpene	*Streptomyces* sp. TSRI0395 scaffold4 (100% of genes show similarity)	terpenoid
Region 1.13	15	RiPP-like	*Streptomyces* sp. TSRI0261 scaffold5 (100% of genes show similarity)	modified peptide
Region 1.14	16	NRPS, T2PKS, Oligosaccharide	warkmycin CS1 biosynthetic gene cluster from *Streptomyces* sp. CS057 (97% of genes show similarity)	warkmycin CS1
Region 1.15	17	NI-siderophore	schizokinen biosynthetic gene cluster from *Nostoc* sp. *PCC 7120 = FACHB-418*	schizokinen
Region 1.16	18	Terpene	*Streptomyces baarnensis* strain NRRL B-2842 P144 (93% of genes show similarity)	terpenoid
Region 1.17	19	Lanthipeptide-Class-III	AmfS biosynthetic gene cluster from *Streptomyces griseus* subsp. *griseus NBRC 13350*	AmfS
Region 1.18	20	Melanin	melanin biosynthetic gene cluster from *Streptomyces griseus* subsp. *griseus NBRC 13350*	melanin
Region 1.19	21	LAP, Thiopeptide	*Streptomyces* sp. CB02115 scaffold1 (100% of genes show similarity)	modified peptide
Region 1.20	22	NRPS-like,T1PKS	*Streptomyces* sp. CB02115 scaffold1 (83% of genes show similarity)	unknown
Region 1.21	23	Arylpolyene	*Streptomyces* sp. CB02115 scaffold1 (100% of genes show similarity)	unknown
Region 1.22	24	NRPS, Arylpolyene, Ladderane	skyllamycin A biosynthetic gene cluster from *Streptomyces* sp. *Acta 2897* (89% of genes show similarity)	skyllamycin A
Region 1.23	25	Melanin	Streptomyces sp. CB02115 scaffold8 (100% of genes show similarity)	melanin
Region 1.24	26	NI-siderophore	*Streptomyces* sp. Root1295 contig 7 (100% of genes show similarity)	dehydroxynocardamine
Region 1.25	27	Lanthipeptide-Class-II, Lanthipeptide-Class-III	*Streptomyces anulatus* strain ATCC 11523 (100% of genes show similarity)	lanthipeptide
Region 1.26	28	Ectoine	ectoine biosynthetic gene cluster from *Streptomyces anulatus*	ectoine
Region 1.27	29	Terpene	*Streptomyces* sp. CB00072 scaffold6 (100% of genes show similarity)	terpenoid
Region 1.28	30	NRPS	*Streptomyces* sp. Root1295 contig 12 (91% of genes show similarity)	NR peptide
Region 1.29	31	T3PKS	Streptomyces sp. CB02115 scaffold5 (93% of genes show similarity)	polyketide
Region 1.30	32	NRP-metallophore	-	unknown
Region 1.30	33	NRPS	griseobactin biosynthetic gene cluster from *Streptomyces* sp. *ATCC 700974*	griseobactin
Region 1.31	34	Terpene	geosmin biosynthetic gene cluster from *Streptomyces coelicolor A3(2)*	geosmin
Region 1.32	35	Butyrolactone	*Streptomyces anulatus* strain ATCC 11523 (100% of genes show similarity)	unknown butyrolactone

RLA120

Region	No	Cluster type	Presence in another bacterium	Putative product
Region 1.1	1	NRPS-like, PKS-like, T1PKS, Butyrolactone, Ectoine, Oligosaccharide	-	unknown
Region 1.1	2	NRPS, Arylpolyene	-	unknown
Region 1.2	3	Butyrolactone	-	unknown butyrolactone
Region 2.1	4	RiPP-like	*Streptomyces* sp. RPA4-2 chromosome (100% of genes show similarity)	modified peptide
Region 2.2	5	PKS-like	-	unknown polyketide
Region 2.3	6	RiPP-like	*Streptomyces* sp. RPA4-2 chromosome (100% of genes show similarity)	modified peptide
Region 2.4	7	T2PKS, Indole	spore pigment biosynthetic gene cluster from *Streptomyces avermitilis* (83% of genes show similarity)	spore pigment
Region 2.5	8	Terpene	hopene biosynthetic gene cluster from *Streptomyces coelicolor A3(2)* (92% of genes show similarity)	hopene
Region 2.6	9	NI-siderophore	*Streptomyces* sp. QMT-28 chromosome (100% of genes show similarity)	siderophore
Region 2.7	10	T1PKS	*Streptomyces* sp. RPA4-2 chromosome (93% of genes show similarity)	polyketide
Region 2.8	11	Terpene	geosmin biosynthetic gene cluster from *Streptomyces coelicolor A3(2)*	geosmin
Region 2.9	12	RiPP-like	*Streptomyces* sp. RPA4-2 chromosome (100% of genes show similarity)	modified peptide
Region 2.10	13	NI-siderophore	*Streptomyces* sp. QMT-28 chromosome (100% of genes show similarity)	siderophore
Region 2.11	14	Terpene	albaflavenone biosynthetic gene cluster from *Streptomyces coelicolor A3(2)*	albaflavenone
Region 2.12	15	T2PKS, Butyrolactone	*Streptomyces* sp. RPA4-2 chromosome (89% of genes show similarity)	unknown
Region 2.13	16	NRPS	*Streptomyces* sp. CB02261 scaffold3 (91% of genes show similarity)	NR peptide
Region 2.14	17	NI-siderophore	desferrioxamin B biosynthetic gene cluster from *Streptomyces griseus* subsp. *griseus NBRC 13350*	desferrioxamin B
Region 2.15	18	Melanin	melanin biosynthetic gene cluster from *Streptomyces coelicolor A3(2)*	melanin
Region 2.16	19	RRE-containing	-	unknown
Region 2.17	20	Ectoine	ectoine biosynthetic gene cluster from *Streptomyces anulatus*	ectoine
Region 2.18	21	NAPAA	ε-Poly-L-lysine biosynthetic gene cluster from *Epichloe festucae*	ε-Poly-L-lysine
Region 2.19	22	T3PKS	flaviolin biosynthetic gene cluster from *Streptomyces coelicolor A3(2)*	flaviolin/1,3,6,8-tetrahydroxynaphthalene
Region 2.20	23	NRPS	-	NR peptide
Region 2.21	24	T1PKS	*Streptomyces* sp. QMT-28 chromosome (81% of genes show similarity)	polyketide
Region 2.22	25	LAP, Thiopeptide	-	unknown
Region 2.23	26	NAPAA, NRPS, Lanthipeptide-Class-III	*Streptomyces* sp. QMT-28 chromosome (97% of genes show similarity)	unknown
Region 2.24	27	Betalacone	*Streptomyces* sp. RPA4-2 chromosome (92% of genes show similarity)	unknown
Region 2.25	28	Melanin	melanin biosynthetic gene cluster from *Streptomyces avermitilis* (71% of genes show similarity)	melanin
Region 2.26	29	Terpene	2-methylisoborneol biosynthetic gene cluster from *Streptomyces griseus* subsp. *griseus NBRC 13350*	2-methylisoborneol
Region 2.27	30	T3PKS	alkylresorcinol biosynthetic gene cluster from *Streptomyces griseus* subsp. *griseus NBRC 13350*	alkylresorcinol

RLA123

Region	No	Cluster type	Presence in another bacterium	Putative product
Region 3.1	1	NRPS	-	NR peptide
Region 3.2	2	NRPS, T3PKS	-	NR peptide polyketide hybrid
Region 3.3	3	NRPS	-	NR peptide
Region 3.4	4	NRPS	-	NR peptide
Region 3.5	5	T3PKS	naringenin biosynthetic gene cluster from *Streptomyces clavuligerus ATCC 27064*	naringenin
Region 3.5	6	LAP, Thiopeptide	-	unknown
Region 3.6	7	Terpene	-	terpenoid
Region 3.7	8	Terpene	hopene biosynthetic gene cluster from *Streptomyces coelicolor A3(2)* (84% of genes show similarity)	hopene
Region 3.8	9	RiPP-like	*Streptomyces glauciniger* strain CGMCC 4.1858 (100% of genes show similarity)	modified peptide
Region 3.9	10	NI-Siderophore	*Streptomyces glauciniger* strain CGMCC 4.1858 (100% of genes show similarity)	siderophore
Region 3.10	11	T2PKS	*Streptomyces glauciniger* strain CGMCC 4.1858 (62% of genes show similarity)	polyketide
Region 3.11	12	Terpene	-	terpenoid
Region 3.12	13	NAPAA	ε-Poly-L-lysine biosynthetic gene cluster from *Epichloe festucae*	ε-Poly-L-lysine
Region 3.13	14	Terpene	*Streptomyces* sp. SID8377 SID8377.c32 (71% of genes show similarity)	terpenoid
Region 3.14	15	Terpene	*Streptomyces* sp. SID8377 SID8377.c28 (77% of genes show similarity)	terpenoid
Region 3.15	16	NI-Siderophore	-	siderophore
Region 3.16	17	RiPP-like, T2PKS	*Streptomyces glauciniger* strain CGMCC 4.1858 (71% of genes show similarity)	modified peptide polyketide hybrid
Region 3.17	18	RiPP-like	-	modified peptide
Region 3.18	19	Terpene	-	terpenoid
Region 3.19	20	NAPAA	-	unknown
Region 3.20	21	NRP-metallophore,NRPS	scabichelin biosynthetic gene cluster from *Streptomyces scabiei 87.22*	scabichelin
Region 3.21	22	Terpene	isorenieratene biosynthetic gene cluster from *Streptomyces griseus* subsp. *griseus NBRC 13350*	isorenieratene
Region 4.1	23	Butyrolactone	-	unknown butyrolactone

RLA131

Region	No	Cluster type	Presence in another bacterium	Putative product
Region 2.1	1	NRPS	-	NR peptide
Region 2.2	2	NRPS, Terpene	-	NR peptide terpenoid hybrid
Region 2.3	3	Terpene	avermitilol biosynthetic gene cluster from *Streptomyces avermitilis MA-4680 = NBRC 14893*	avermitilol
Region 2.4	4	RiPP-like	-	modified peptide
Region 2.5	5	Terpene	isorenieratene biosynthetic gene cluster from *Streptomyces griseus* subsp. *griseus NBRC 13350*	isorenieratene
Region 2.6	6	Lanthipeptide-Class-I	-	lanthipeptide
Region 2.7	7	Ectoine	ectoine biosynthetic gene cluster from *Streptomyces anulatus*	ectoine
Region 2.8	8	T2PKS	-	polyketide
Region 2.9	9	Terpene	-	terpenoid
Region 2.10	10	Terpene	-	terpenoid
Region 2.11	11	Terpene	geosmin biosynthetic gene cluster from *Streptomyces coelicolor A3(2)*	geosmin
Region 2.12	12	Lanthipeptide-Class-I	-	lanthipeptide
Region 2.13	13	NRPS-like, T1PKS, Butyrolactone	*Streptomyces purpureus* KA281 StrpuDRAFT (65% of genes show similarity)	unknown
Region 2.14	14	2dos, T2PKS	spore pigment biosynthetic gene cluster from *Streptomyces avermitilis* (66% of genes show similarity)	spore pigment
Region 2.15	15	NRPS-like	-	NR peptide
Region 2.16	16	NRPS, T1PKS, Butyrolactone	-	unknown
Region 2.17	17	NI-siderophore	*Streptomyces* sp. CB02058 scaffold3 (83% of genes show similarity)	siderophore
Region 2.18	18	NRPS	-	NR peptide
Region 2.19	19	RiPP-like	-	modified peptide
Region 2.20	20	RiPP-like, Hydrogen-cyanide	-	unknown
Region 2.21	21	NAPAA	ε-Poly-L-lysine biosynthetic gene cluster from *Epichloe festucae*	ε-Poly-L-lysine
Region 2.22	22	Terpene	hopene biosynthetic gene cluster from *Streptomyces coelicolor A3(2)* (76% of genes show similarity)	hopene
Region 2.23	23	T1PKS	-	polyketide
Region 2.24	24	NI-siderophore	legonoxamine A biosynthetic gene cluster from *Streptomyces* sp.	legonoxamine A, desferrioxamine B, legonoxamine B
Region 2.25	25	NI-siderophore	-	siderophore
Region 2.26	26	T1PKS, hglE-KS	-	polyketide
Region 2.27	27	Redox-cofactor	-	unknown
Region 2.27	28	Betalactone	-	unknown betalactone
Region 2.28	29	Lassopeptide	-	lassopeptide
Region 2.28	30	T3PKS	-	polyketide

RLA150

Region	No	Cluster type	Presence in another bacterium	Putative product
Region 1.1	1	T1PKS,Butyrolactone	coelimycin P1 biosynthetic gene cluster from *Streptomyces coelicolor A3(2)*, (79% of genes show similarity)	coelimycin P1
Region 1.2	2	LAP	*Streptomyces subrutilus* strain ATCC 27467 (86% of genes show similarity)	unknown
Region 1.3	3	LAP,T2PKS,Butyrolactone,Thiopeptide	-	unknown
Region 1.4	4	NRPS, NRPS-like	antipain biosynthetic gene cluster from Streptomyces sp.	antipain
Region 1.4	5	PKS-like,Butyrolactone,Terpene	-	unknown
Region 1.5	6	NRPS,NRPS-like,Arylpolyene,Lassopeptide	*Streptomyces peucetius* strain NA0869 (68% of genes show similarity)	unknown
Region 1.6	7	NRPS	-	NR peptide
Region 2.1	8	RiPP-like	-	unknown
Region 2.2	9	Lanthipeptide class IV	venezuelin biosynthetic gene cluster from *Streptomyces venezuelae ATCC 10712*	venezuelin
Region 2.3	10	Terpene	avermitilol biosynthetic gene cluster from *Streptomyces avermitilis MA-4680 = NBRC 14893*	avermitilol
Region 2.4	11	T2PKS	spore pigment biosynthetic gene cluster from *Streptomyces avermitilis*, (66% of genes show similarity)	spore pigment (polyketide)
Region 2.4	12	NAPAA	-	unknown
Region 2.5	13	Terpene	-	terpenoid
Region 2.6	14	NAPAA	ε-Poly-L-lysine biosynthetic gene cluster from *Epichloe festucae*	ε-Poly-L-lysine
Region 2.7	15	T1PKS	-	unknown
Region 2.8	16	LAP,RRE-containing,Thiopeptide	-	unknown
Region 2.8	17	T3PKS	alkylresorcinol biosynthetic gene cluster from Streptomyces griseus subsp. griseus NBRC 13350	alkylresorcinol
Region 2.8	18	NI-Siderophore	-	siderophore
Region 2.9	19	Melanin	melanin biosynthetic gene cluster from *Streptomyces coelicolor A3(2)*	melanin
Region 2.10	20	NRP-metallophore,NRPS,NRPS-like,T1PKS	-	unknown
Region 2.11	21	Lassopeptide	-	unknown
Region 2.12	22	T1PKS,hglE-KS	*Streptomyces* sp. H27-S2 (77% of genes show similarity)	polyketide
Region 2.13	23	Terpene	*Streptomyces lavendulae* subsp. *lavendulae* (100% of genes show similarity)	terpenoid
Region 2.14	24	NRPS,terpene	-	unknown
Region 2.15	25	RiPP-like	*Streptomyces subrutilus* strain ATCC 27467 (100% of genes show similarity)	peptide
Region 2.16	26	Hydrogen-cyanide	*Streptomyces* sp. ADI95-16 (100% of genes show similarity)	unknown
Region 2.17	27	NI-siderophore	*Streptomyces flavotricini* strain NGL1 (95% of genes show similarity)	siderophore
Region 2.18	28	CDPS	*Streptomyces* sp. H27-S2 (100% of genes show similarity)	peptide
Region 2.19	29	NRPS-like,T1PKS	-	unknown
Region 2.20	30	NRP-metallophore,NRPS	coelichelin biosynthetic gene cluster from *Streptomyces coelicolor A3(2)*	coelichelin
Region 2.21	31	Lanthipeptide-class-iii	SapB biosynthetic gene cluster from *Streptomyces coelicolor A3(2)*	SapB
Region 2.22	32	Ectoine	ectoine biosynthetic gene cluster from *Streptomyces anulatus*	ectoine
Region 2.23	33	Other	-	unknown
Region 2.24	34	NRPS	-	NR peptide
Region 2.25	35	Terpene	-	terpenoid
Region 2.26	36	Terpene	isorenieratene biosynthetic gene cluster from *Streptomyces griseus* subsp. *griseus* NBRC 13350	isorenieratene

RLA153

Region	No	Cluster type	Presence in another bacterium	Putative product
Region 1.1	1	Terpene	-	terpenoid
Region 1.2	2	Butyrolactone	*Streptomyces* sp. Mg1 plasmid pSMg1-1 (100% of genes show similarity)	unknown butyrolactone
Region 1.3	3	Butyrolactone	*Streptomyces* sp. ADI95-16 plasmid pADI95-16a (100% of genes show similarity)	unknown butyrolactone
Region 1.4	4	T1PKS	*Streptomyces* sp. ADI95-16 plasmid pADI95-16a (66% of genes show similarity)	polyketide
Region 1.5	5	PKS-like, Butyrolactone	-	unknown
Region 2.1	6	Terpene	-	terpenoid
Region 2.2	7	NRPS-like, T1PKS	*Streptomyces* sp. ADI95-16 chromosome (73% of genes show similarity)	unknown
Region 2.3	8	Terpene	*Streptomyces* sp. ADI95-16 chromosome (100% of genes show similarity)	terpenoid
Region 2.4	9	NRPS	*Streptomyces* sp. ADI95-16 chromosome (97% of genes show similarity)	NR peptide
Region 2.5	10	Lanthipeptide-Class-III, Terpene	*Streptomyces* sp. ADI95-16 chromosome (100% of genes show similarity)	lanthipeptide terpenoid hybrid
Region 2.6	11	CDPS	*Streptomyces* sp. ADI95-16 chromosome (100% of genes show similarity)	cyclodipeptide
Region 2.7	12	T1PKS	-	polyketide
Region 2.7	13	NAPAA, NRPS, transAT-PKS	weishanmycin biosynthetic gene cluster from *Streptomyces* sp. CB02120-2	weishanmycin
Region 2.7	14	Terpene	-	terpenoid
Region 2.8	15	T1PKS	linearmycin A biosynthetic gene cluster from *Streptomyces sp. Mg1*	linearmycin A, linearmycin B
Region 2.8	16	LAP, RRE-containing, Thiopeptide	-	unknown
Region 2.8	17	NRPS	-	Desferri-peucechelin, salinichelin C
Region 2.9	18	T1PKS, hglE-KS	*Streptomyces* sp. CGMCC 4.1796 chromosome (82% of genes show similarity)	polyketide
Region 2.10	19	NI-Siderophore	desferrioxamin B biosynthetic gene cluster from *Streptomyces coelicolor A3(2)*	desferrioxamin B
Region 2.11	20	Lanthipeptide-Class-I	*Streptomyces* sp. CGMCC 4.1796 chromosome (82% of genes show similarity)	lanthipeptide
Region 2.12	21	Lanthipeptide-Class-I	*Streptomyces* sp. ADI95-16 chromosome (90% of genes show similarity)	lanthipeptide
Region 2.13	22	NI-Siderophore	*Streptomyces* sp. ADI95-16 chromosome (100% of genes show similarity)	siderophore
Region 2.14	23	Hydrogen-Cyanide	*Streptomyces* sp. ADI95-16 chromosome (100% of genes show similarity)	unknown
Region 2.15	24	RiPP-like	*Streptomyces* sp. ADI95-16 chromosome (100% of genes show similarity)	modified peptide
Region 2.16	25	NRPS, RiPP-like, T1PKS, Terpene	-	unknown
Region 2.17	26	Terpene	*Streptomyces* sp. APSN-46.1 NODE 1 (100% of genes show similarity)	terpenoid
Region 2.18	27	Lanthipeptide-Class-II, Lanthipeptide-Class-III	*Streptomyces* sp. ADI95-16 chromosome (100% of genes show similarity)	lanthipeptide
Region 2.19	28	NRPS, NRPS-like, T1PKS	*Streptomyces* sp. ADI95-16 chromosome (96% of genes show similarity)	NR peptide polyketide hybrid
Region 2.20	29	Lanthipeptide-Class-III	SapB biosynthetic gene cluster from Streptomyces coelicolor A3(2)	SapB
Region 2.21	30	Melanin	melanin biosynthetic gene cluster from *Streptomyces coelicolor A3(2)*	melanin
Region 2.22	31	T2PKS	spore pigment biosynthetic gene cluster from *Streptomyces avermitilis*	spore pigment
Region 2.22	32	NI-Siderophore, T3PKS	*Streptomyces* sp. ADI95-16 chromosome (100% of genes show similarity)	unknown
Region 2.23	33	Terpene	*Streptomyces* sp. ADI95-16 chromosome (100% of genes show similarity)	terpenoid
Region 2.24	34	NRPS	*Streptomyces* sp. ADI95-16 chromosome (100% of genes show similarity)	NR peptide
Region 2.25	35	RiPP-like	*Streptomyces* sp. ADI95-16 chromosome (66% of genes show similarity)	modified peptide
Region 2.26	36	PKS-like, T1PKS	-	polyketide
Region 2.27	37	other	-	unknown
Region 2.28	38	Nucleoside	-	nucleoside

RLA156

Region	No	Cluster type	Presence in another bacterium	Putative product
Region 1.1	1	NAPAA	ε-Poly-L-lysine biosynthetic gene cluster from *Epichloe festucae*	ε-Poly-L-lysine
Region 1.1	2	CDPS	-	cyclodipeptide
Region 1.2	3	T3PKS	alkylresorcinol biosynthetic gene cluster from *Streptomyces griseus* subsp. *griseus NBRC 13350*	alkylresorcinol
Region 1.3	4	NI-Siderophore	*Streptomyces* sp. *Sge12* chromosome, complete genome (72% of genes show similarity)	siderophore
Region 1.4	5	Melanin	*Streptomyces venezuelae* strain ATCC 21018 (100% of genes show similarity)	unknown
Region 1.5	6	Terpene	*Streptomyces nojiriensis* strain JCM 3382 (60% of genes show similarity)	terpenoid
Region 1.6	7	Terpene	*Streptomyces venezuelae* strain ATCC 21018 (94% of genes show similarity)	terpenoid
Region 1.7	8	Terpene	-	terpenoid
Region 1.7	9	Lanthipeptide-Class-III	SapB biosynthetic gene cluster from *Streptomyces coelicolor A3(2)*	SapB
Region 1.8	10	NRPS-like	lipstatin biosynthetic gene cluster from *Streptomyces toxytricini* (42% of genes show similarity)	lipstatin
Region 1.9	11	T1PKS	-	polyketide
Region 1.10	12	NRPS-like	-	NR peptide
Region 1.11	13	NRPS-like, T1PKS	candicidin biosynthetic gene cluster from *Streptomyces* sp. *FR-008* (80% of genes show similarity)	candicidin
Region 1.12	14	NRPS	-	NR peptide
Region 1.13	15	T1PKS, HglE-KS	*Streptomyces venezuelae* strain ATCC 21018 (86% of genes show similarity)	polyketide
Region 1.14	16	Terpene	*Streptomyces nojiriensis* strain JCM 3382 (91% of genes show similarity)	terpenoid
Region 1.15	17	Lanthipeptide-Class-IV	-	lanthipeptide
Region 1.16	18	Terpene	geosmin biosynthetic gene cluster from *Streptomyces coelicolor A3(2)*	geosmin
Region 1.17	19	RiPP-like	-	modified peptide
Region 1.18	20	RiPP-like	*Streptomyces* sp. ADI92-24 scaffold002, whole genome (81% of genes show similarity)	modified peptide
Region 1.19	21	Hydrogen-cyanide	*Streptomyces* sp. NBC 00162 chromosome (100% of genes show similarity)	unknown
Region 1.20	22	NI-Siderophore	*Streptomyces nojiriensis* strain JCM 3382 (96% of genes show similarity)	siderophore
Region 1.21	23	CDPS	*Streptomyces nojiriensis* strain JCM 3382 (100% of genes show similarity)	cyclodipeptide
Region 1.22	24	Lassopeptide	albusnodin biosynthetic gene cluster from *Streptomyces albus*	albusnodin
Region 1.23	25	LAP	-	unknown peptide
Region 1.24	26	Lanthipeptide-Class-I	-	lanthipeptide
Region 1.25	27	NI-Siderophore	desferrioxamin B biosynthetic gene cluster from *Streptomyces coelicolor A3(2)*	desferrioxamin B
Region 1.26	28	Lanthipeptide-Class-V	-	lanthipeptide
Region 1.27	29	NRPS, T1PKS	clipibicyclene biosynthetic gene cluster from *Streptomyces cattleya NRRL 8057 = DSM 46488* (94% of genes show similarity)	clipibicyclene
Region 1.28	30	Butyrolactone	*Streptomyces* sp. *Sge12* chromosome, complete genome (88% of genes show similarity)	unknown butyrolcatone
Region 1.29	31	NRPS	coelichelin biosynthetic gene cluster from *Streptomyces coelicolor A3(2)*	coelichelin
Region 1.29	32	T1PKS	*Streptomyces nojiriensis* strain JCM 3382 (95% of genes show similarity)	polyketide
Region 1.30	33	NRPS, NRPS-like	JBIR-126 biosynthetic gene cluster from *Streptomyces* sp. NRRL F-4474 (92% of genes show similarity)	JBIR-126
Region 1.31	34	T2PKS	*Streptomyces nojiriensis* strain JCM 3382 (89% of genes show similarity)	polyketide
Region 1.32	35	NRP-metallophore, NRPS	-	unknown
Region 1.33	36	NRPS, NRPS-like	antipain biosynthetic gene cluster from Streptomyces sp.	antipain
Region 2.1	37	T2PKS, Butyrolactone, Ectoine	-	unknown
Region 2.2	38	Butyrolactone, PKS-like	-	unknown
Region 2.2	39	LAP, Thiopeptide	-	unknown

RLA186

Region	No	Cluster type	Presence in another bacterium	Putative product
Region 1.1	1	RiPP-like	-	modified peptide
Region 1.2	2	RiPP-like	-	modified peptide
Region 1.3	3	Terpene	hopene biosynthetic gene cluster from *Streptomyces coelicolor A3(2)* (84% of genes show similarity)	hopene
Region 1.4	4	RiPP-like	*Streptomyces* sp. *S501* chromosome, complete genome (100% of genes show similarity)	modified peptide
Region 1.5	5	Arylpolyene	-	unknown
Region 1.6	6	NI-Siderophore	*Streptomyces* sp. *ADI92-24* scaffold002, whole genome (96% of genes show similarity)	siderophore
Region 1.7	7	Terpene	-	terpenoid
Region 1.8	8	NRPS-like, T1PKS	*Streptomyces* sp. *CNS654* CD02DRAFT (89% of genes show similarity)	NR peptide polyketide hybrid
Region 1.9	9	T3PKS	naringenin biosynthetic gene cluster from *Streptomyces clavuligerus ATCC 27064*	naringenin
Region 1.10	10	NI-Siderophore	desferrioxamin B biosynthetic gene cluster from *Streptomyces coelicolor A3(2)*	desferrioxamin B
Region 1.11	11	Butyrolcatone	-	unknown butyrolactone
Region 1.12	12	Ectoine	ectoine biosynthetic gene cluster from *Streptomyces anulatus*	ectoine
Region 1.13	13	Terpene	*Streptomyces* sp. CB00271 chromosome (93% of genes show similarity)	terpenoid
Region 1.14	14	RiPP-like, T3PKS	-	modified peptide polyketide hybrid
Region 1.15	15	NAPAA,NRPS	-	unknown
Region 1.16	16	T2PKS	spore pigment biosynthetic gene cluster from *Streptomyces avermitilis*	spore pigment
Region 1.16	17	Terpene	-	terpenoid
Region 1.17	18	Terpene	-	terpenoid

RLA191

Region	No	Cluster type	Presence in another bacterium	Putative product
Region 1.1	1	Terpene	-	terpenoid
Region 1.2	2	NRPS, T1PKS	-	NR peptide polyketide hybrid
Region 1.3	3	RiPP-like	streptamidine biosynthetic gene cluster from *Streptomyces albidoflavus J1074* (75% of genes show similarity)	streptamidine
Region 1.4	4	Ectoine	ectoine biosynthetic gene cluster from *Streptomyces anulatus*	ectoine
Region 1.5	5	Terpene	geosmin biosynthetic gene cluster from *Streptomyces coelicolor A3(2)*	geosmin
Region 1.6	6	Terpene	*Streptomyces lateritius* strain Z1-26 (88% of genes show similarity)	terpenoid
Region 1.7	7	Lanthipeptide-Class-III	-	lanthipeptide
Region 1.8	8	Lanthipeptide-Class-II, Terpene	*Streptomyces lateritius* strain Z1-26 (70% of genes show similarity)	unknown
Region 1.9	9	Terpene	*Streptomyces lateritius* strain Z1-26 (90% of genes show similarity)	terpenoid
Region 1.10	10	NRPS, Betalactone	*Streptomyces lateritius* strain Z1-26 (95% of genes show similarity)	NR peptide betalactone hybrid
Region 1.11	11	NRPS	pyrroloformamide A biosynthetic gene cluster from *Streptomyces* sp. *CB02980* (70% of genes show similarity)	pyrroloformamide A
Region 1.12	12	RiPP-like, T2PKS	-	modifiied peptide polyketide hybrid
Region 1.13	13	NI-Siderophore	desferrioxamin B biosynthetic gene cluster from *Streptomyces griseus* subsp. *griseus NBRC 13350*	desferrioxamin B
Region 1.14	14	NRPS	*Streptomyces lateritius* strain Z1-26 (100% of genes show similarity)	NR peptide
Region 1.15	15	Melanin	*Streptomyces lateritius* strain Z1-26 (100% of genes show similarity)	unknown
Region 1.16	16	T3PKS	*Streptomyces* sp. *CB02261* scaffold11, whole genome (96% of genes show similarity)	polyketide
Region 1.17	17	NI-Siderophore	*Streptomyces* sp. *CB02261* scaffold11, whole genome (92% of genes show similarity)	siderophore
Region 1.18	18	NI-Siderophore	*Streptomyces lateritius* strain Z1-26 (95% of genes show similarity)	siderophore
Region 1.19	19	Indole	*Streptomyces lateritius* strain Z1-26 (87% of genes show similarity)	unknown
Region 1.20	20	NRPS, NRPS-like, RiPP-like	*Streptomyces lateritius* strain Z1-26 (98% of genes show similarity)	unknown
Region 1.20	21	HR-T2PKS, thioamide-NRP, NRPS, Hydrogen-cyanide	*Streptomyces lateritius* strain Z1-26 (98% of genes show similarity)	unknown
Region 1.21	22	RiPP-like	*Streptomyces lateritius* strain Z1-26 (100% of genes show similarity)	modifiied peptide
Region 1.22	23	Butyrolactone	*Streptomyces lateritius* strain Z1-26 (83% of genes show similarity)	unknown butyrolactone
Region 1.23	24	Terpene	hopene biosynthetic gene cluster from *Streptomyces coelicolor A3(2)* (76% of genes show similarity)	hopene
Region 1.24	25	Lanthipeptide-Class-III	SapB biosynthetic gene cluster from *Streptomyces coelicolor A3(2)*	SapB
Region 1.25	26	Melanin	*Streptomyces lateritius* strain Z1-26 (100% of genes show similarity)	unknown
Region 1.26	27	Arylpolyene	*Streptomyces lateritius* strain Z1-26 (82% of genes show similarity)	unknown
Region 1.27	28	NRP-metallophore, NRPS	*Streptomyces lateritius* strain Z1-26 (100% of genes show similarity)	unknown
Region 1.28	29	Terpene	*Streptomyces lateritius* strain Z1-26 (88% of genes show similarity)	terpenoid
Region 1.29	30	T2PKS	spore pigment biosynthetic gene cluster from *Streptomyces avermitilis* (83% of genes show similarity)	spore pigment
Region 2.1	31	Terpene	-	terpenoid

RLA240

Region	No	Cluster type	Presence in another bacterium	Putative product
Region 2.1	1	Lanthipeptide class I	-	unknown lanthipeptide
Region 2.2	2	Melanin	*Streptomyces niveus* NCIMB 11891 (90% of genes show similarity)	melanin (saccharide)
Region 2.3	3	NRPS, Arylpolyene	-	unknown
Region 2.3	4	Terpene	2-methylisoborneol biosynthetic gene cluster from *Streptomyces griseus* subsp. *griseus* NBRC 13350	2-methylisoborneol
Region 2.4	5	Terpene	Streptomyces sp. S4.7 chromosome, complete genome (80% of genes show similarity)	unknown carotenoid
Region 2.4	6	T1PKS, hglE-KS,	Streptomyces sp. S4.7 chromosome, complete genome (80% of genes show similarity)	glycosylated polyketide
Region 2.5	7	T3PKS	naringenin biosynthetic gene cluste*r Streptomyces clavuligerus* ATCC 27064	naringenin (polyketide Type 3)
Region 2.6	8	NRP-metallophore,NRPS	coelichelin biosynthetic gene cluster from *Streptomyces coelicolor A3(2)*	coelichelin (NR peptide)
Region 2.7	9	Terpene	raimonol biosynthetic gene cluster from *Streptomyces anulatus*	raimonol (terpene)
Region 2.8	10	NRPS,PKS-like,T1PKS,transAT-PKS	alpiniamide biosynthetic gene cluster from *Streptomyces* sp. CBMAI 2042 (55% similarity)	alpiniamide
Region 2.9	11	Butyrolactone	-	unknown butyrolactone
Region 2.10	12	T2PKS	-	aromatic glycosilated polyketide
Region 2.11	13	Terpene	*Streptomyces niveus* NCIMB 11891 (95% similarity)	hopene (terpene)
Region 2.12	14	Terpene	*Streptomyces niveus* strain SCSIO 3406 (87% similarity)	NR peptide
Region 2.13	15	RiPP-like	*Streptomyces niveus* strain SCSIO 3406 (100% similarity)	bacteriocin
Region 2.14	16	T1PKS	-	polyketide
Region 2.15	17	T2PKS, Butyrolactone	-	unknown type II polyketide
Region 2.16	18	NI-siderophore	*Streptomyces* sp. 150FB scaffold00001 (88% of genes show similarity)	unknown siderophore
Region 2.17	19	other	*Streptomyces niveus* NCIMB 11891 (76% of genes show similarity)	unknown secondary metabolite
Region 2.18	20	Terpene	-	unknown terpene
Region 2.19	21	Lanthipeptide class I	*Streptomyces argenteolus* strain 3259 Ga0365441 (80% of genes show similarity)	unknown lanthipeptide
Region 2.20	22	Terpene	geosmin biosynthetic gene cluster from *Streptomyces coelicolor* A3(2)	geosmin
Region 2.21	23	Terpene	*Streptomyces niveus* strain SCSIO 3406 (100% of genes show similarity)	unknown NR peptide
Region 2.22	24	LAP, Thiopeptide	*Streptomyces niveus* strain SCSIO 3406 (100% of genes show similarity)	unknown
Region 2.23	25	Lanthipeptide class V	*Streptomyces niveus* NCIMB 11891 (90% of genes show similarity)	unknown lanthipeptide
Region 2.24	26	Terpene	*Streptomyces* sp. S4.7 (61% of genes show similarity)	unknown terpenoids
Region 2.25	27	NRPS-like	amicetin biosynthetic gene cluster from *Streptomyces vinaceusdrappus* (83% similarity)	amicetin-like compound
Region 2.26	28	NRPS	*Streptomyces niveus* strain SCSIO 3406 (91% of genes show similarity)	unknown NR peptide
Region 2.27	29	Ectoine	*Streptomyces niveus* strain SCSIO 3406 (100% of genes show similarity)	ectoine
Region 2.28	30	T1PKS	*Streptomyces niveus* NCIMB 11891 (87% of genes show similarity	BE-14106 macrolactam
Region 2.29	31	T3PKS	alkylresorcinol biosynthetic gene cluster from *Streptomyces griseus* subsp. *griseus* NBRC 13350	alkylresorcinol (polyketide)
Region 2.30	32	NRPS-like, NRPS, T1PKS, other	*Streptomyces* sp. WAC 01529 (48% of genes show similarity)	peptide polyketide hybrid polyoxipeptin-like
Region 2.31	33	NRPS, T1PKS, NRPS-like	Streptomyces sp. ZFG47 chromosome, complete genome (21% of genes show similarity)	peptide polyketide hybrid
Region 3.1	34	Lassopeptide	-	lassopeptide
Region 3.2	35	Guanidinotides	-	unknown
Region 3.3	36	Lanthipeptide class I	-	unknow lanthipeptide
Region 4.1	37	T1PKS	-	unknown polyketide
Region 4.2	38	Thioamitides	-	unknown RiPP
